# From Mechanistic Study to Chiral Catalyst Optimization: Theoretical Insight into Binaphthophosphepine-catalyzed Asymmetric Intramolecular [3 + 2] Cycloaddition

**DOI:** 10.1038/s41598-017-07863-9

**Published:** 2017-08-08

**Authors:** Meng Duan, Lei Zhu, Xiaotian Qi, Zhaoyuan Yu, Yingzi Li, Ruopeng Bai, Yu Lan

**Affiliations:** School of Chemistry and Chemical Engineering, Chongqing University, Chongqing, 400030 China

## Abstract

Density functional M11 was used to study the mechanism and enantioselectivity of a binaphthophosphepine-catalyzed intramolecular [3 + 2] cycloaddition reaction. The computational results revealed that this reaction proceeds through nucleophilic addition of the phosphine catalyst to the allene, which yields a zwitterionic phosphonium intermediate. The subsequent stepwise [3 + 2] annulation process, which starts with the intramolecular nucleophilic addition of the allenoate moiety to the electron-deficient olefin group, determines the enantioselectivity of the reaction. This step is followed by a ring-closing reaction and water-assisted proton-transfer process to afford the final product with concomitant regeneration of the phosphine catalyst. Theoretical predictions of the enantioselectivity for various phosphine catalysts were consistent with experimental observations, and 2D contour maps played an important role in explaining the origin of the enantioselectivity. Moreover, on the basis of our theoretical study, new binaphthophosphepine catalysts were designed and that are expecting to afford higher enantioselectivity in this cycloaddition reaction.

## Introduction

The development of organocatalysis, which contain no transition metals, is of prime interest because of the extensive applications of these species in organic synthesis^1–7^. Among various organocatalysts, phosphine catalysis has become a powerful tool for the construction of carbocycles, heterocycles, functionalized spirocyclic, and fused ring systems, which have found broad synthesis applications on bioactive natural products and medicinally important substances^8–13^. Organic phosphine catalysts have been studied for more than half a century with pioneering works involving their use in Wittig reaction^14–16^, Morita–Baylis–Hillman reaction^17–19^, Rauhut–Currier reaction^20, 21^, Lu reaction^22–25^, Michael reaction^26–28^, and some other umpolung reactions^29–31^. Although diversified substrates and product structures in these reactions, from a mechanistic point of view, they are all initiated by nucleophilic addition of the lone pair of a phosphine to yield a phosphonium intermediate^32–35^. On the other hand, phosphonium species is also a good leaving group and can be easily regenerated; this is critical for the continuous operation of the catalytic cycle^36, 37^. In particular, the phosphine-catalyzed [3 + 2] cycloaddition of allenes with unsaturated bonds, known as the Lu reaction, provides an efficient approach for the construction of five-membered carbocycles owing to the mild reaction conditions, high product yield, and high atom economy^38–41^.

Triphenylphosphine is the most common catalyst for phosphine-catalyzed [3 + 2] cycloadditions because of its easy availability, low cost, and high catalytic activity^42–45^. In addition, high enantioselectivity and/or diastereoselectivity can be achieved by the introduction of asymmetric trisubstituted phosphines^46–50^. As a result of the broad application of this catalyst in the construction of five-membered carbocycles, the mechanism of this [3 + 2] cycloaddition has attracted considerable attention by both theoretical and experimental chemists^34, 51–54^. In Huisgen-type [3 + 2] cycloadditions, Lewis acids generally act as catalysts to activate 1,3-dipoles so that they could react with dipolarophiles^55–59^. However, Lu^22–25^, Fu^60, 61^, Zhang^62^, and others^47, 63–66^ have independently reported [3 + 2] cycloadditions in which a phosphine catalyst plays the role of a Lewis base. The generally accepted mechanism of phosphine-catalyzed [3 + 2] cycloaddition starts with a zwitterionic phosphonium intermediate, which is formed by the nucleophilic addition of the phosphine catalyst to an allene species. Subsequent [3 + 2] cycloaddition between the zwitterionic phosphonium intermediate and an electron-deficient unsaturated bond then occurs to yield a phosphorus ylide. Previous theoretical investigations have proposed that this is a stepwise process^51, 53^. A solvent-assisted [1,2]-proton transfer leads to the formation of a cycloadduct, and finally the active phosphine catalyst is released.

**Figure 1:**
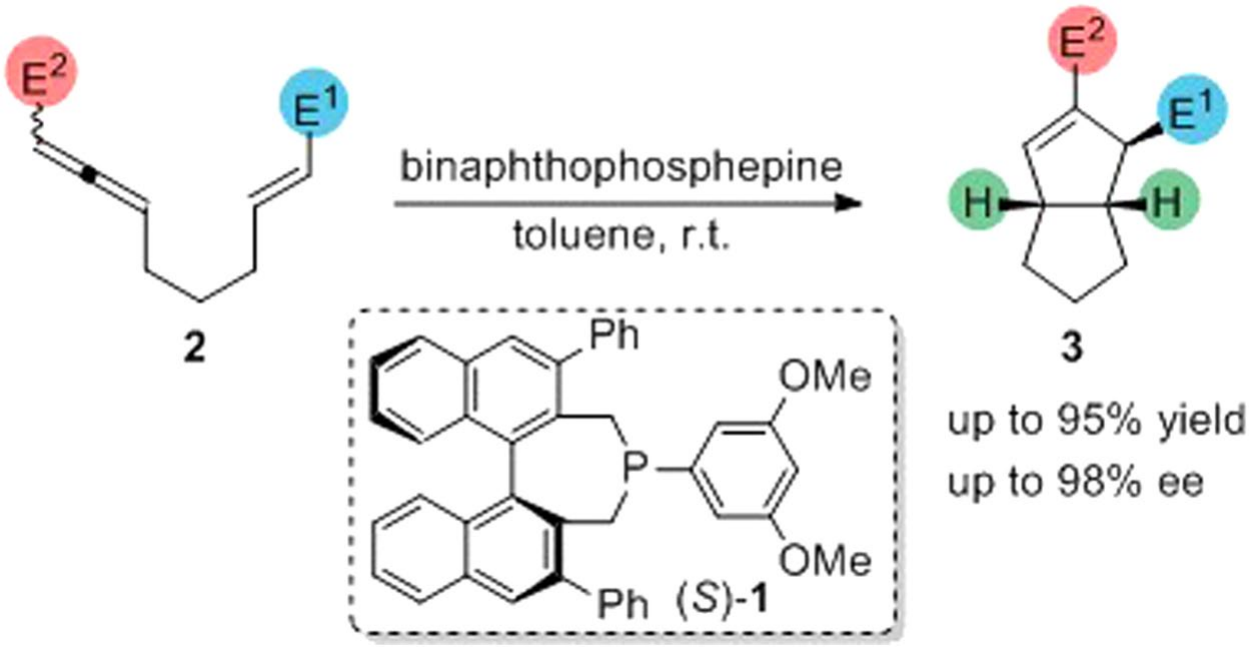
Binaphthophosphepine-catalyzed enantioselective intramolecular [3 + 2] cycloaddition.

It has recently been shown that using a chiral phosphine greatly extends the potential applications of phosphine-catalyzed [3 + 2] cycloadditions to the construction of asymmetric cyclopentenes^52, 67–69^. In contrast to previous theoretical and experimental studies on symmetric phosphine-catalyzed [3 + 2] cycloadditions, the mechanism of chiral phosphine-catalyzed [3 + 2] cycloaddition remains unclear, especially the origin of the chirality in the product. A good example of a chiral phosphine catalyst is binaphthophosphepine, which was first proposed and synthesized by Gladiali *et al*.^70^; this compound is extensively used as a ligand or organic catalyst in asymmetric synthesis owing to its strong chiral induction^71–75^. As shown in Fig. 1, Fu and coworkers have reported the enantioselective intramolecular [3 + 2] cycloaddition of allene **2** to generate chiral [3.3.0] bicyclic products **3** using a series of binaphthophosphepines as catalysts^76^. This reaction occurs under mild conditions with good yields. When chiral binaphthophosphepine (*S*)-**1**, designed by Fu’s group, was used in the intramolecular [3 + 2] annulation of racemic allenes, the observed *ee* was as high as 98%. Owing to the potential for the widespread application of this type of reactions and the rapid development of phosphine organocatalysis, the detailed study of the reaction mechanism is desired, especially on the origin of the enantioselectivity and the conversion pathway of the racemic reactants. We therefore performed density functional theory (DFT) calculations to reveal the reaction mechanism and the origin of the enantioselectivity in this annulation. Moreover, the design of new binaphthophosphepines based on theoretical predictions is also discussed.

### Computational Methods

All calculations were performed with Gaussian 09 program^77^. Geometry optimization of the minimum energy structures and transition states was carried out at the B3-LYP^78, 79^ level of theory with the 6-31G(d) basis set. Harmonic vibrational frequency calculations were performed for all stationary points to identify whether they were local minima or transition structures, and to derive the thermochemical corrections for the enthalpies and free energies. Solvent effect in toluene were considered implicitly by performing single-point energy calculations on the gas-phase optimized geometries using the SMD^80^ polarizable continuum model. Solvation single-point energies were obtained using the M11^81–85^ functional with the 6-311+G(d,p) basis set for all other atoms. Intrinsic reaction coordinate (IRC) calculations of key step were used to confirm that the transition states connected the corresponding reactants and products. The natural bond orbital (NBO) technique was applied to calculate the Wiberg bond indices to analyze the bonding^86^.

## Results and Discussion

**Figure 2:**
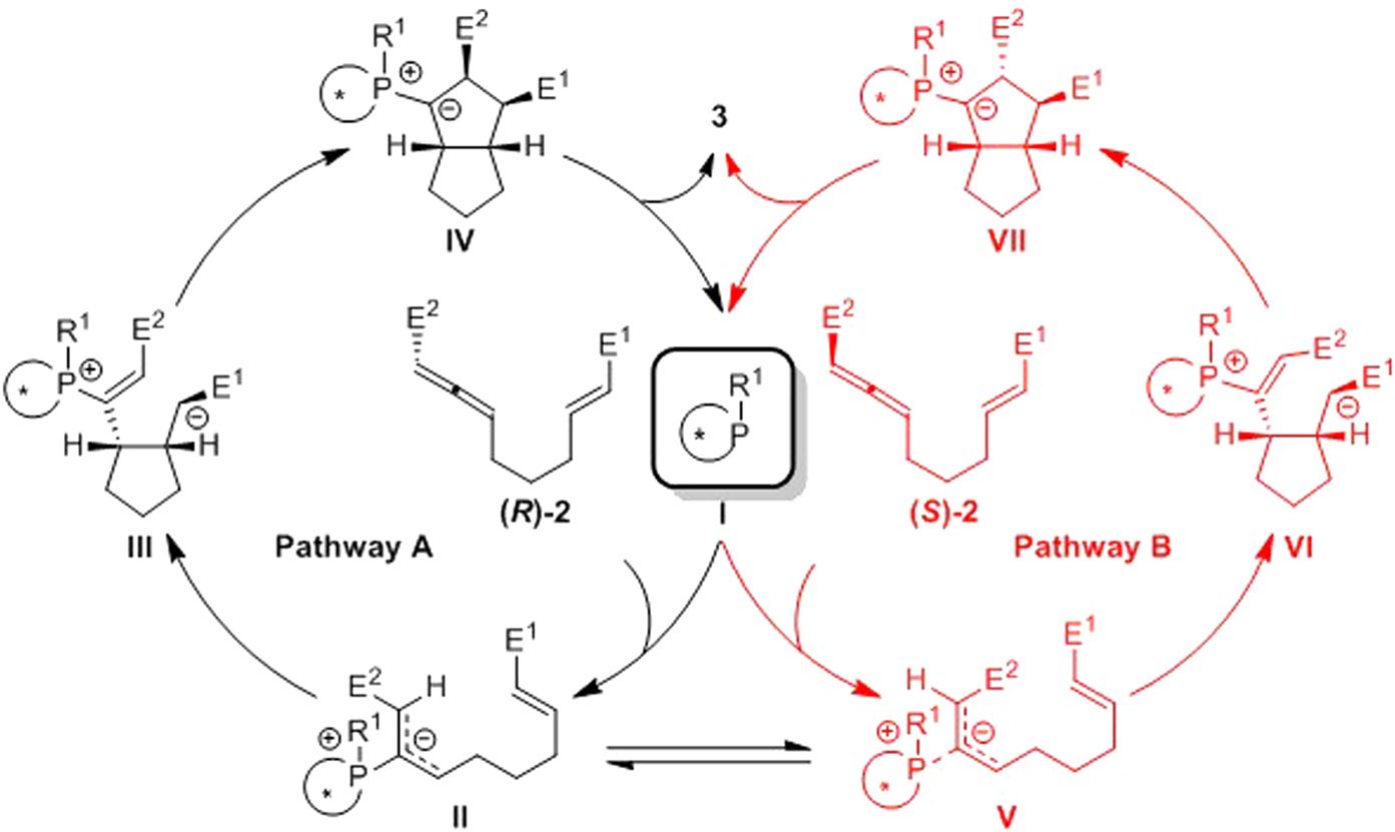
The proposed mechanism of binaphthophosphepine-catalyzed enantioselective intramolecular [3 + 2] cycloaddition of racemic allene (±)-2.

Based on previous theoretical and experimental studies, the proposed reaction pathways for the binaphthophosphepine-catalyzed enantioselective intramolecular [3 + 2] cycloaddition of racemic allene **2** are shown in Fig. 2. The initiation step of this reaction is the nucleophilic addition of binaphthophosphepine catalyst **I** with the (*R*)-**2** enantiomer to generate zwitterionic phosphonium intermediate **II**, which has a *gauche* conformation. Nucleophilic addition is also possible with the other enantiomer (*S*)-**2** to yield regioisomer **V**, and isomerization between **II** and **V** occurs through C–C bond rotation. After intermediate **II** is formed, another nucleophilic addition occurs to form the first new C–C bond in intermediate **III**. It is believed that the enantioselectivity is controlled by this step. Subsequent cyclization generates five-membered carbocycle intermediate **IV**, and finally water-assisted [1,2]-proton transfer leads to the generation of cycloadduct **3** and the regeneration of active phosphine catalyst **I**. Alternatively, intermediate **II** may be isomerized to intermediate **V**, and a similar process from intermediate **V** also yields cycloadduct **3**.

However, in the proposed mechanism, there are still some issues that remain unclear: (1) the consistency of (or differences in) the reaction pathway when using different enantiomers (e.g., (*R*)-**2** and (*S*)-**2**), and (2) the factors controlling the enantioselectivity in the [3 + 2] cycloaddition step. We thus performed DFT calculations to solve these problems and reveal the mechanism of this reaction.

**Figure 3:**
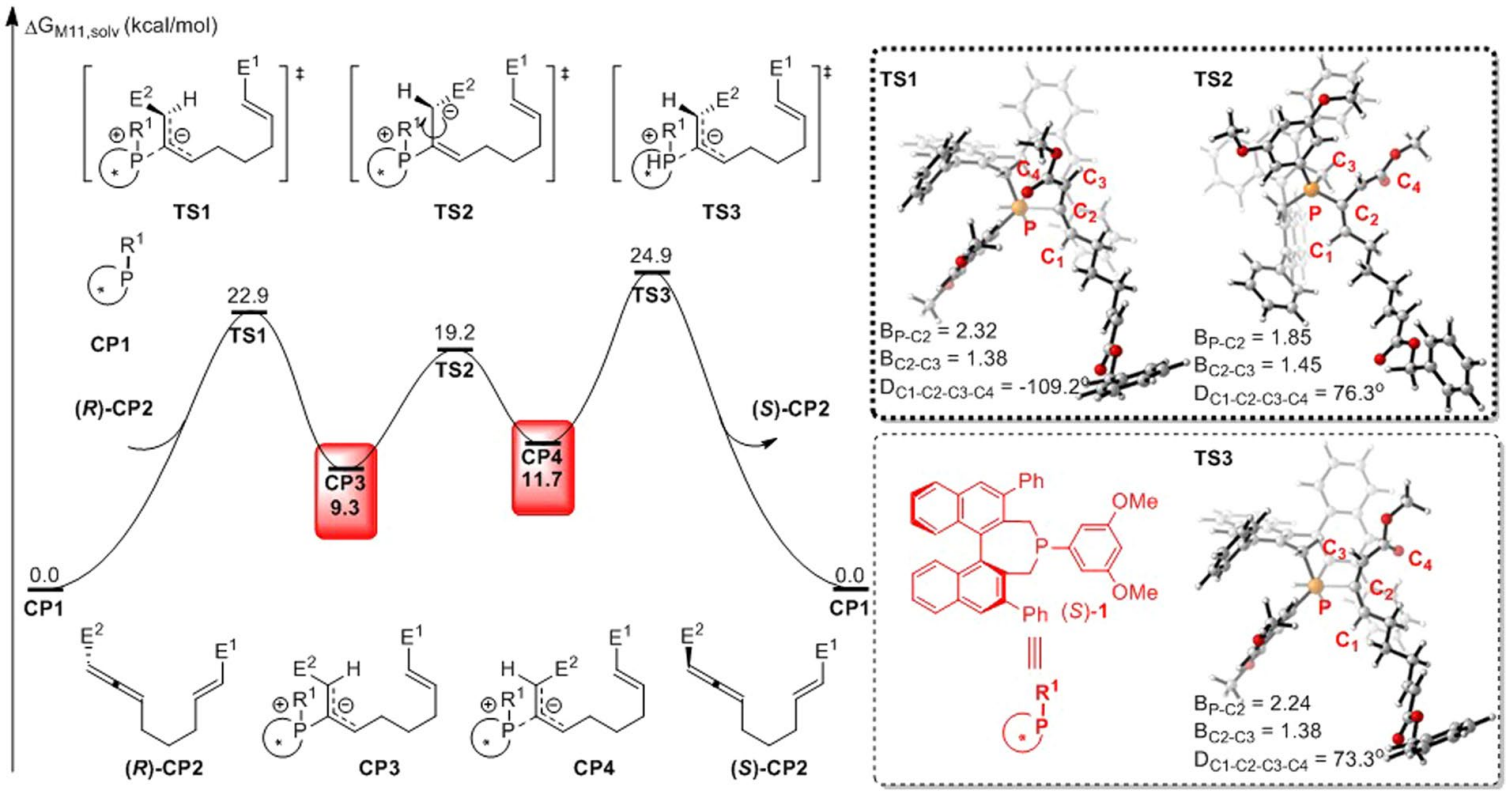
Free energy profiles for the nucleophilic addition step of binaphthophosphepine-catalyzed intramolecular [3 + 2] annulation (E^1^ = CO_2_Bn, E^2^ = CO_2_Me). Values are given by kcal/mol and represent the relative free energies calculated by M11 method in toluene solvent. The values in geometry information are given by angstrom.

**Figure 4:**
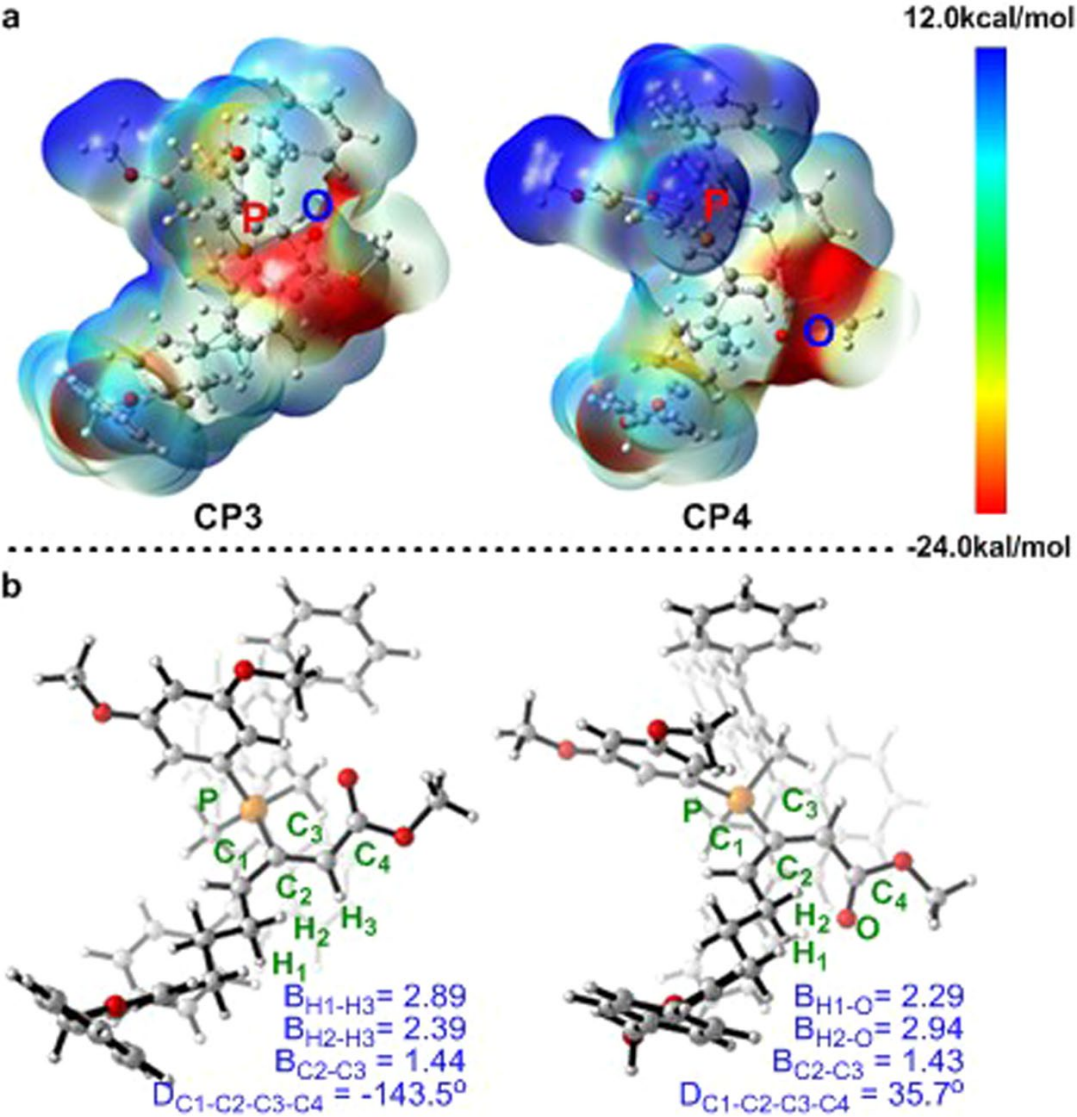
(**a**) Electrostatic potential map for **CP3** (left) and **CP4** (right). (**b**) Geometries of Intermediates **CP3** (left) and **CP4** (right). The values of bond lengths are given in angstroms.

In our theoretical study, binaphthophosphepine (*S*)-**1** was selected as the model catalyst, and racemic complex **CP2** was chosen as the reactant. Our theoretical investigations first focused on the nucleophilic addition step. As shown in Fig. 3, the nucleophilic addition of binaphthophosphepine (*S*)-**1** to the *β* position of (*R*)-**CP2** takes place via transition state **TS1** and has a free energy barrier of 22.9 kcal/mol. This nucleophilic addition reversibly affords intermediate **CP3** with a *gauche* conformation, with 9.3 kcal/mol endothermic. The C2–C3 bond length in **CP3** is 1.44 Å (Fig. 4), which is much longer than a typical C=C double bond. The NBO analysis of intermediate **CP3** also shows that the NBO bond order of the C2–C3 bond is only 1.06. This bond can therefore rotate via transition state **TS2** with a barrier of only 9.9 kcal/mol to reversibly generate intermediate **CP4**.

We found that intermediate **CP3** is 2.4 kcal/mol more stable than **CP4**. An electrostatic potential (ESP) calculation was used to clarify the energy difference between **CP3** and **CP4**. As shown in the ESP maps in Fig. 4, for both intermediates **CP3** and **CP4**, the positive charge (cool tones) is located on the phosphonium moiety, and the center of the negative charge (warm tones) is on the electron-withdrawing group in the terminal position of the allenoate moiety. In **CP4**, the electron-withdrawing group is far away from the phosphonium moiety, and this charge separation probably leads to the higher relative free energy of **CP4**. Meanwhile, the existence of A1,3 strain in **CP4** also results in the instability of **CP4**.

When intermediate **CP4** is formed, P–C2 bond cleavage can occur via transition state **TS3** to afford the reactant with the *S*-configuration (*S*)-**CP2** and release catalyst (*S*)-**1**. The theoretical calculations indicate that the binaphthophosphepine catalyst could promote the inversion of the configuration of reactant **CP2**. Interestingly, based on the principle of microreversibility, nucleophilic addition of binaphthophosphepine (*S*)-**1** with (*S*)-**CP2** could also occur via the same transition state **TS3** with a barrier of 24.9 kcal/mol to reversibly yield the zwitterionic phosphonium intermediate **CP4** common to both steps. The subsequent [3 + 2] cycloaddition could start from either **CP3** or **CP4**. The relative free energy of transition state **TS1** is 2.0 kcal/mol lower than that of transition state **TS3**, which indicates that nucleophilic addition to (*R*)-**CP2** is easier than that to its enantioisomer (*S*)-**CP2**. Therefore, if the reaction were stopped partway through, more unreacted (*S*)-**CP2** would be observed. The computed *ee* value of the unreacted allene is 93%, which is in good agreement with experimental observations.

**Figure 5:**
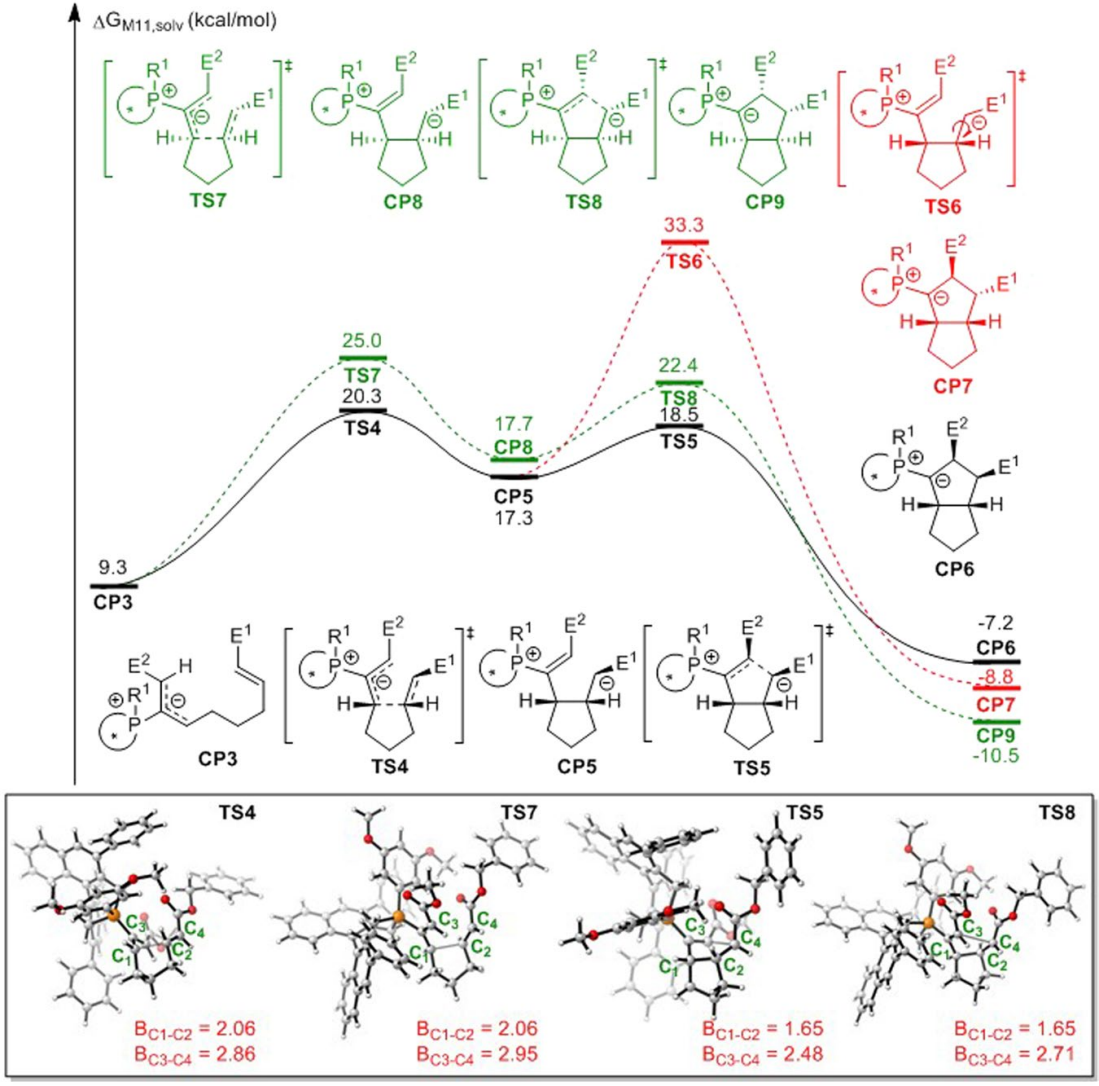
Free energy profiles for the [3 + 2] cycloaddition step of binaphthophosphepine-catalyzed intramolecular annulation from intermediate **CP3** (E^1^ = CO_2_Bn, E^2^ = CO_2_Me). Values are given by kcal/mol and represent the relative free energies calculated by M11 method in toluene solvent. The values in geometry information are given by angstrom.

For the subsequent [3 + 2] cycloaddition from **CP3** or **CP4**, both concerted and stepwise mechanisms have been considered. The computational results for the stepwise [3 + 2] cycloaddition pathway starting from **CP3** are summarized in Fig. 5. The ESP map showed that the positive charge is mainly located on the internal carbon of the electrophile moiety in **CP3** (Fig. 4). Therefore, the first intramolecular C–C bond formation occurs via transition state **TS4** to reversibly yield carbon anionic intermediate **CP5**. The relative free energy of transition state **TS4** is 2.6 or 4.6 kcal/mol lower than those of transition states **TS1** or **TS3**, respectively. Therefore, the previous nucleophilic addition step is expected to be the rate limiting step. After zwitterionic intermediate **CP5** is formed, the following cycloaddition exothermically generates phosphorus ylide **CP6** via transition state **TS5**, which has a barrier of only 1.2 kcal/mol. The rotation of the C–P bond in **CP3** is blocked by the bulky phosphonium moiety when (*S*)-**1** is used as the catalyst; it is therefore favorable for intramolecular electrophilic attack to occur on the *re*-face of the allene moiety via transition state **TS4**. In contrast, additional energy is required to rotate the C–P bond for the subsequent *si*-face attack on the allene moiety via transition state **TS7**. The calculated activation free energy via **TS4** is 4.7 kcal/mol lower than that via **TS7**. This corresponds to a binaphthophosphepine catalyst-induced diastereoselectivity of 1:199, which indicates an *ee* of 99.9% for the final product. This calculated enantioselectivity is consistent with the experimental results. As shown in Fig. 5, the first C–C bond formation is the critical step for the stereospecificity of the reaction. A relatively stable phosphorus ylide (**CP6** or **CP9**) is generated after the second electrophilic addition to the vinylphosphonium moiety, and the new five-membered ring is constructed after this step. Additionally, when the carbon anion is formed in **CP5**, the bond order of the olefin moiety is reduced, which indicates that this bond may be rotated. Unfortunately, the activation barrier for this rotation via transition state **TS6** is 16.0 kcal/mol (red line), which is much higher than that of the ring-closing step. Therefore, *threo*-cycloadduct **CP7** could not be formed. This result is consistent with experimental observations, and also rationalizes our proposed stepwise mechanism.

**Figure 6:**
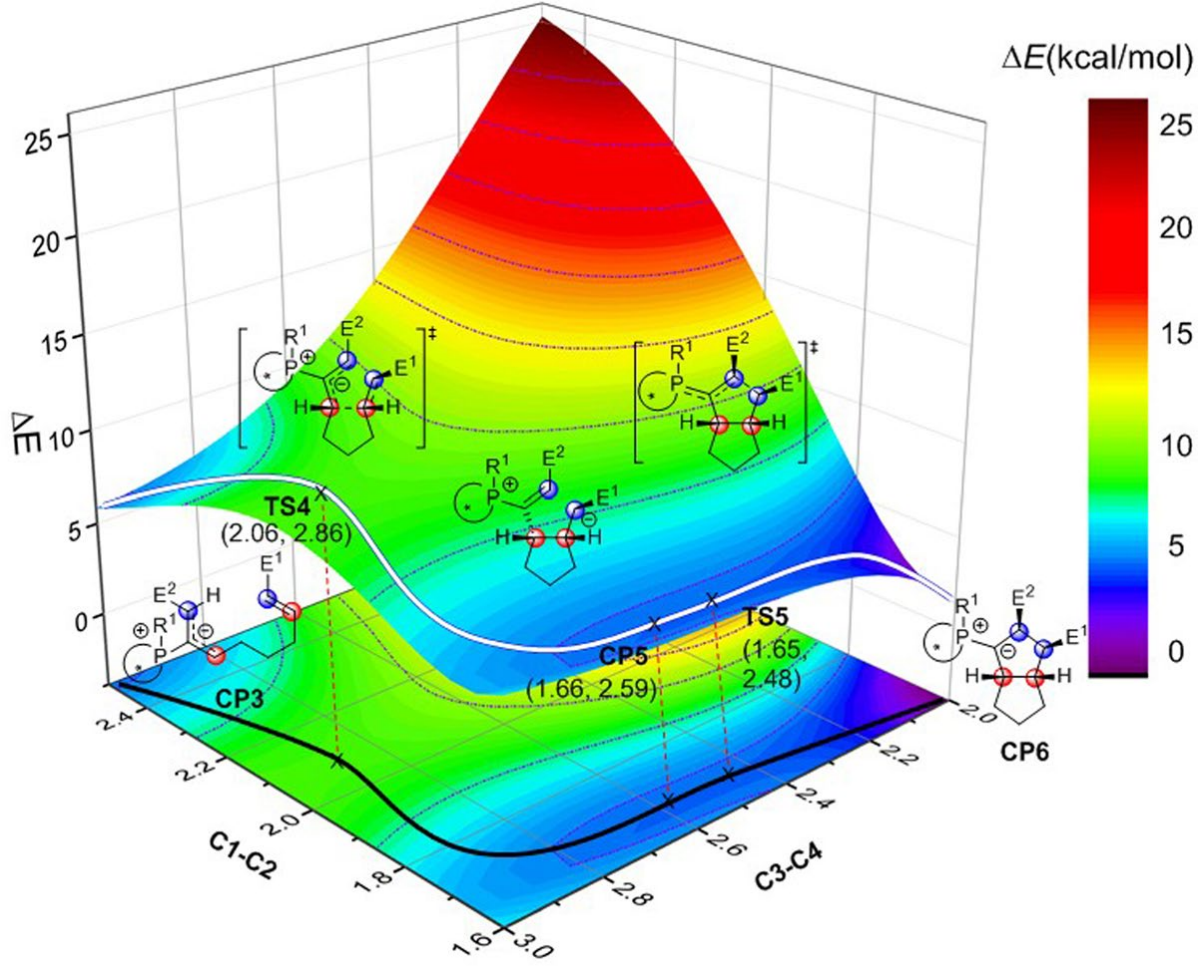
The 2D potential energy surface for the annulation step of binaphthophosphepine-catalyzed intramolecular [3 + 2] cycloaddition reaction calculated by B3-LYP level of theory (E^1^ = CO_2_Bn, E^2^ = CO_2_Me). The relative energies for the surface are given in kcal/mol. The bond axes are given in angstrom. The relative zero is the electronic energy of **CP3**.

Annulation is the key step of binaphthophosphepine-catalyzed intramolecular [3 + 2] cycloaddition reaction. To exclude the concerted mechanism, a 2D potential energy surface calculation is performed at B3-LYP level of theory^87–89^. The reaction pathway is labeled in Fig. 6. The C1 and C2 atoms are closed first. When the distance of C1-C2is reduced to 2.06 Å, the first saddle point, which is transition state **TS4**, is found. Followed the reaction coordination, when the bond length of C1-C2 is shorter than 1.8 Å, the distance of C3 and C4 atoms begin to reduce evidently. When the distance of C3 and C4 atoms is 2.59 Å, a local minimum, which is named **CP5** is formed. When C3 and C4 atoms is further closed to 2.48 Å, another saddle point **TS5** is found. Throw over that saddle point, the relative free energy is reduced rapidly as well as the generation of the annulation intermediate **CP6**. Around the 2D potential energy surface, two saddle points and three local minima are found. Therefore, the concerted pathway could be excluded.

**Figure 7:**
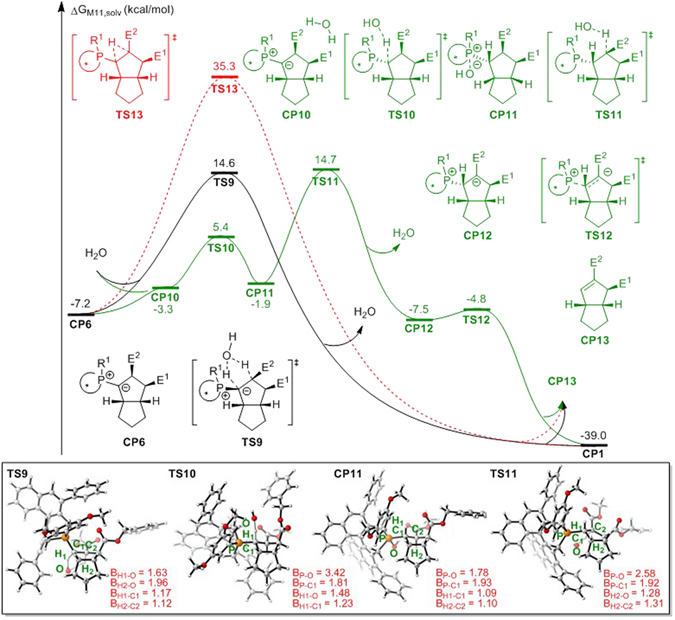
Free energy profiles for the [1,2]-proton shift process (E^1^ = CO_2_Bn, E^2^ = CO_2_Me). Values are given by kcal/mol and represent the relative free energies calculated by M11 method in toluene solvent. The values in geometry information are given by angstrom.

The [1,2]-proton shift in phosphorus ylide to afford cyclopentene product has been previously studied by the Yu group^51, 53^. Their theoretical and experimental investigations suggested a stepwise water-facilitated proton transfer that involved protonation by water to yield a phosphonium intermediate followed by deprotonation. We also considered this step in the binaphthophosphepine-catalyzed intramolecular annulation in our theoretical studies. As shown in Fig. 7, the direct [1,2]-proton shift via transition state **TS13** cannot occur after intermediate **CP6** is formed owing to the very high activation free energy (42.5 kcal/mol). Nevertheless, promotion with water would considerably reduce the barrier of this step. Accordingly, we located a concerted water-molecule-bridged [1,2]-proton transfer transition state, named **TS9**, in the free energy profiles. After this transition state, the phosphonium moiety is decomposed to yield final product **CP13** and regenerate active catalyst **CP1**. Alternatively, stepwise water-facilitated proton transfer may start from the protonation of the phosphorus ylide via transition state **TS10** to reversibly yield hydroxyphosphorane intermediate **CP11** with an energy barrier of only 12.6 kcal/mol. It is followed by deprotonation via transition state **TS11** to generate zwitterionic phosphonium intermediate **CP12**, which is then decomposed to the same product **CP13** and regenerates active catalyst **CP1**. The relative free energy of stepwise water-facilitated proton transfer transition state **TS11** is only 0.1 kcal/mol higher than that of concerted water-molecule-bridged [1,2]-proton transfer transition state **TS9**; therefore, both the water-facilitated concerted and stepwise pathways for the [1,2]-proton transfer step are possible.

**Figure 8:**
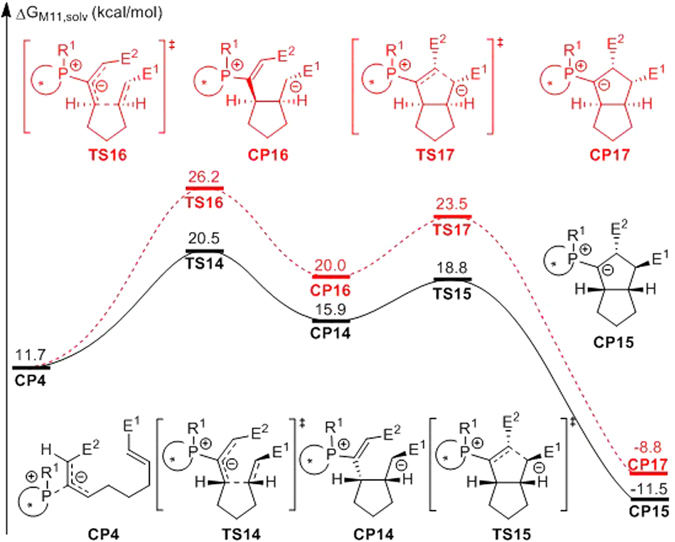
Free energy profiles for the [3 + 2] cycloaddition step from intermediate **CP4** (E^1^ = CO_2_Bn, E^2^ = CO_2_Me). Values are given by kcal/mol and represent the relative free energies calculated by M11 method in toluene solvent.

Stepwise [3 + 2] cycloaddition starting with intermediate **CP4** was also considered (Fig. 8). Although the relative free energy of intermediate **CP4** is 2.4 kcal/mol higher than that of intermediate **CP3**, the relative free energy of the transition state for nucleophilic addition (**TS14**) is nearly the same as that of **TS4**. Thus, if the initial nucleophilic addition occurs via transition state **TS1** to yield intermediate **CP3**, an alternative pathway, besides the direct stepwise [3 + 2] cycloaddition via transition state **TS4**, is isomerization to another zwitterionic phosphonium intermediate **CP4** followed by [3 + 2] annulation via transition state **TS14**. On this pathway, ring closing via transition state **TS15** would yield another phosphorus ylide **CP15**, which is the epimer of **CP6**. Subsequent proton transfer and release of the binaphthophosphepine catalyst from **CP15** would generate **CP13** as the final product, which is the same product as that obtained from intermediate **CP6**. The detailed free energy profiles for the protonation of **CP15** are summarized in the Supporting Information (Figure S1).

**Figure 9:**
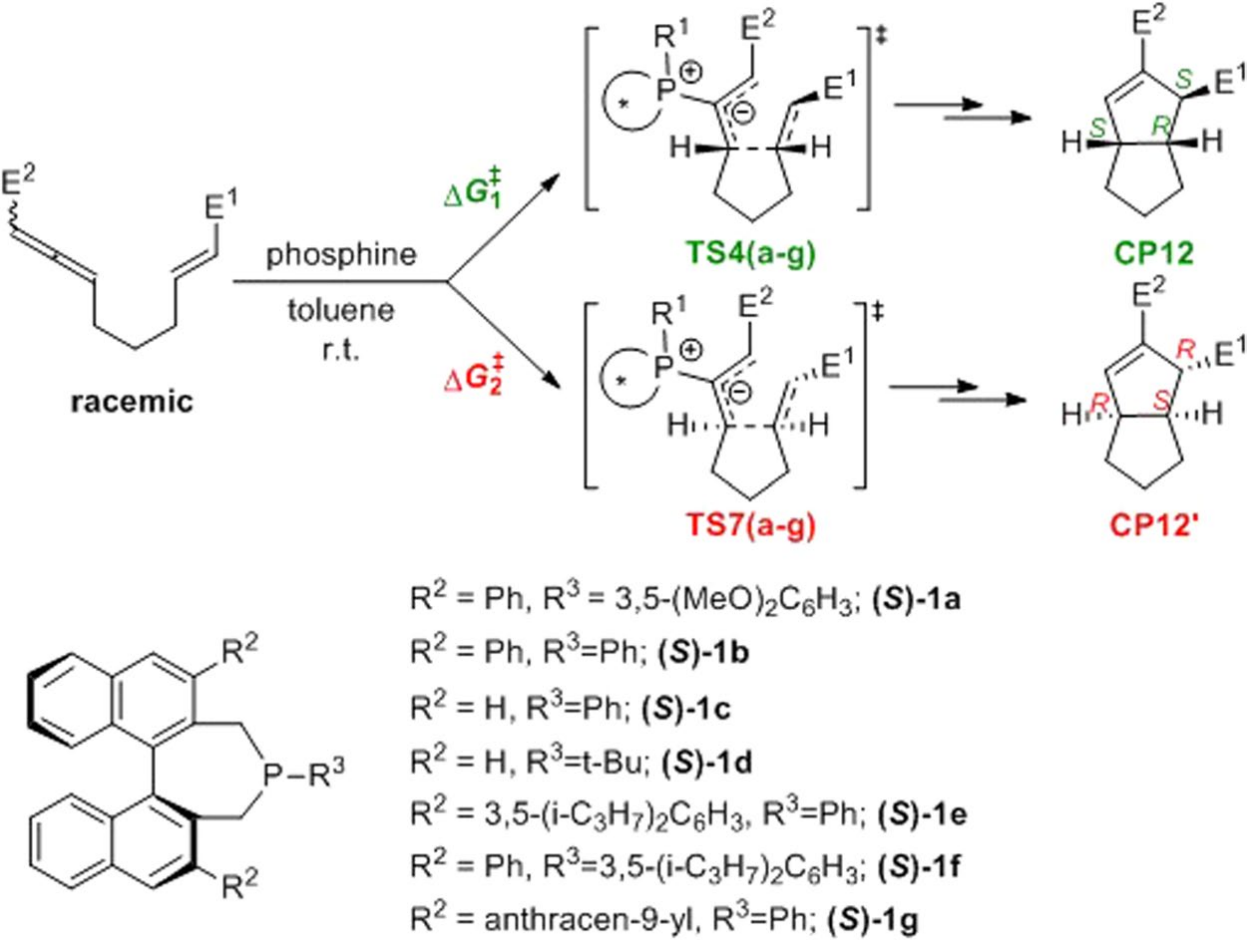
The phosphine catalysts with a series of various substituents.

More than just a tool for mechanistic investigation, quantum chemical calculations can also be used for the evaluation of new reactions and catalyst designs^90, 91^. To understand and improve the enantioselectivity of the binaphthophosphepine-catalyzed intramolecular annulation reaction, we carried out a computational experiment to investigate the effects on stereo induction of phosphine catalysts with various substituents (Fig. 9). Our DFT studies of the reaction mechanism revealed that the enantioselectivity determining step of this reaction is the intramolecular nucleophilic addition of the electron-deficient olefin moiety, which forms the first C–C bond. Based on this idea, the enantioselectivity was evaluated from the relative free energy differences between the transition states for *re*- and *si*-face attack, as shown in Table 1. In our theoretical studies of enantioselectivity, we first considered binaphthophosphepine (*S*)-**1** with substituent groups that have already been used experimentally. The R^2^ and R^3^ groups in (*S*)-**1a** are phenyl and 3,5-dimethoxyphenyl, respectively, and the calculated ΔΔ*G* ^≠^ was 4.7 kcal/mol (entry 1), which indicates an *ee* value of 99.9%.

**Table 1:** Theoretically predicated and experimentally observed enantioselectivities with a series of binaphthophosphepine catalysts.

Entry	Catalyst	*ee*_exp_^a^	*ee*_calc_^b^	ΔΔ*G* ^‡^_exp_^c^	ΔΔ*G* ^‡^_calc_^d^
1	**(** ***S*** **)-1a**	97.0	99.9	2.5	4.7
2	**(** ***S*** **)-1b**	90.0	99.8	1.7	4.0
3	**(** ***S*** **)-1c**	70.0	97.9	1.0	2.7
4	**(** ***S*** **)-1d**	−85.0	−96.6	−1.5	−2.4
5	**(** ***S*** **)-1e**	—	>99.9	—	5.4
6	**(** ***S*** **)-1f**	—	>99.9	—	5.9
7	**(** ***S*** **)-1g**	—	>99.9	—	7.3

^a^The *ee* value obtained in experiment. ^b^The *ee* value obtained in theoretical calculation. ^c^ΔΔ*G* ^‡^_exp_ is extrapolated by experimentally observed *ee* (ΔΔ*G* ^‡^ = −RTln(1 − *ee*)/ (1 + *ee*)). ^*d*^ΔΔ*G* ^‡^_calc_ = Δ*G* ^‡^_2_ − Δ*G* ^‡^_1_, in which Δ*G* ^‡^_1_ and Δ*G* ^‡^_2_ are obtained by theoretical calculation. ^*e*^The data in parentheses are extrapolated by the fitting curve in Fig. 10.

**Figure 10:**
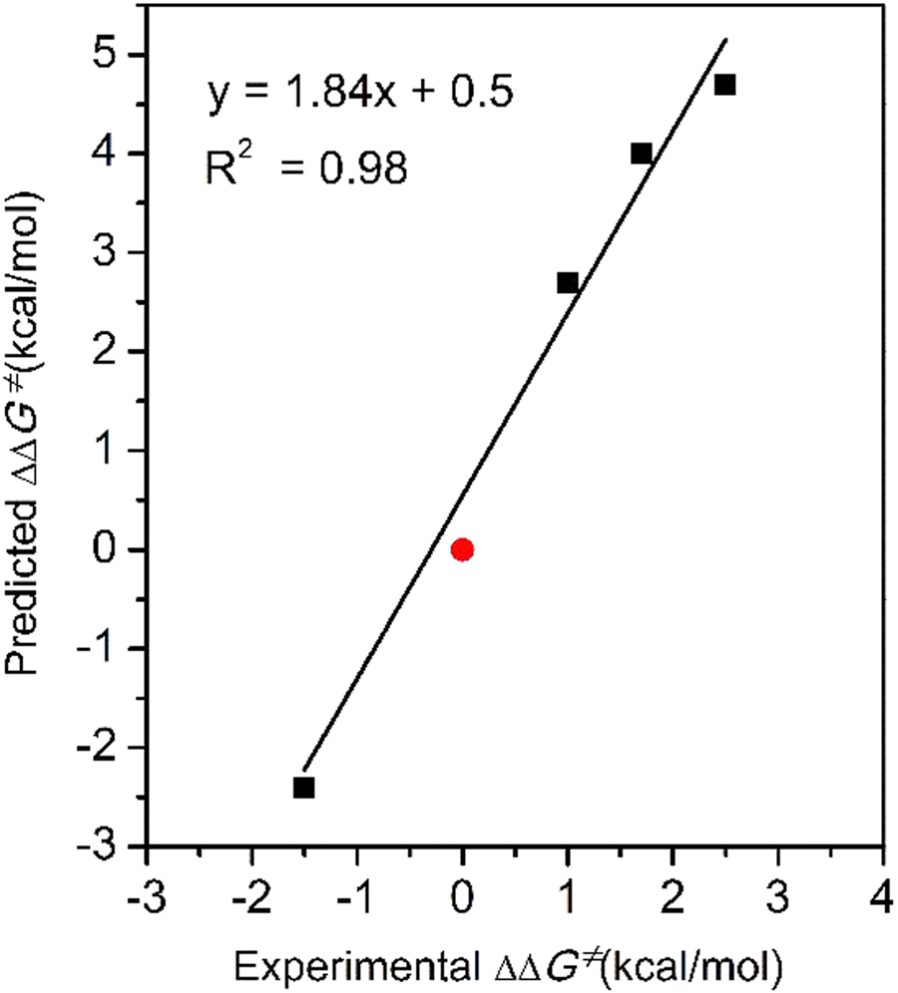
The correlation of calculated activation free energy difference (ΔΔ*G* ^‡^_calc_) and corresponding energy values derived from experimentally observed *ee* (ΔΔ*G* ^‡^_exp_). Plot (0, 0) was added for reference and was not adopted when fitting (red dot).

When the R^3^ group was changed to a phenyl group in catalyst (*S*)-**1b**, the calculated ΔΔ*G* ^≠^ was reduced to 4.0 kcal/mol (entry 2). Furthermore, when the R^2^ group was replaced by a hydrogen atom (entry 3, catalyst (*S*)-**1c**), the calculated ΔΔ*G* ^≠^ was 2.7 kcal/mol. To further verify the accuracy of our computational results, we also calculated the enantioselectivity using (*S*)-**1d** as the catalyst, in which the R^3^ group is a *tert*-butyl group. The calculated *ee* was opposite to that obtained for (*S*)-**1a** and equal to −96.6%, which fully agrees with the experimental observations. A comparison of the experimentally observed and theoretically calculated enantioselectivities of catalysts (*S*)-**1a**, (*S*)-**1b**, (*S*)-**1c**, and (*S*)-**1d** is given in Fig. 10, and shows a good linear correlation between the calculated activation free energy difference and the corresponding energy values derived from the experimentally observed *ee* (correlation coefficient R^2^ = 0.98). The results clearly show that DFT calculations demonstrate the same trend in enantioselectivity as do the experimental results, although every calculated value is slightly higher than the experimentally reported data as a result of systematic error. Both the theoretical calculations and experimental observations confirmed that increasing the size of the R^2^ and R^3^ groups would lead to higher enantioselectivity.

**Figure 11:**
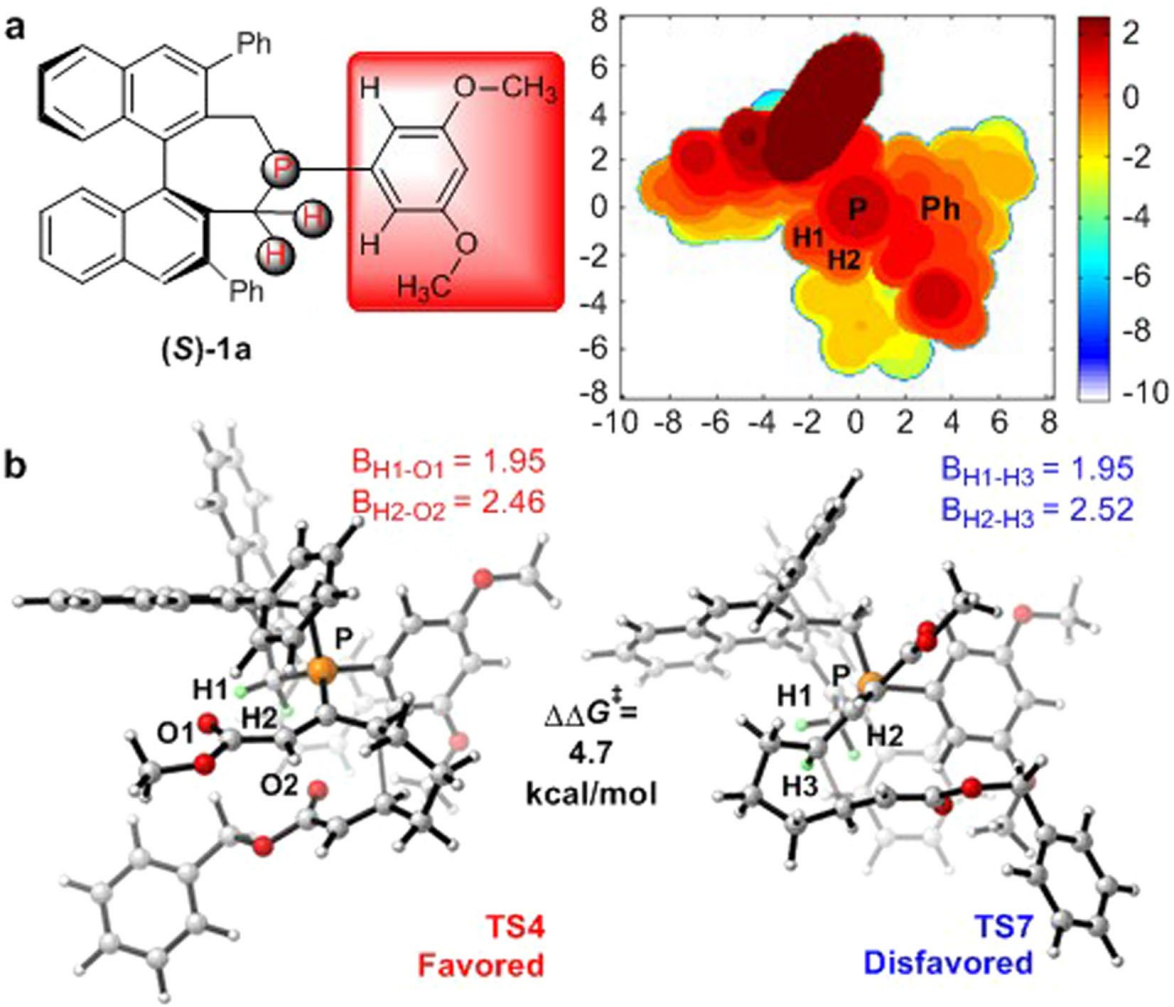
(**a**) 2D contour map of the Van der Waals surface of catalyst **(** ***S*** **)-1a**. Distances are given in angstroms. P atom is located at the origin of the coordinate system in the contour map. The contour line of zero is defined as in the same plane of the P atom. Negative distance (blue) indicates that the atoms on the ligand are farther away from the substrate; positive distance (red) indicates that the atoms on the ligand are closer to the substrate. (**b**) Geometries of transition states **TS-4** and **TS-7** with catalyst **(** ***S*** **)-1a**. The values of bond lengths are given in angstroms.

To better illustrate the steric repulsion in different regions of the binaphthophosphepine catalysts, a 2D contour map along the *z*-axis of the van der Waals surface of catalyst (*S*)**-1a** was plotted^92–95^, which is shown in Fig. 11a. The optimized structures of transition states **TS4** and **TS7** for the C–C bond formation are also shown (Fig. 11b). The 2D contour map clearly shows that the axial phenyl group on the binaphthalene moiety provides the greatest steric hindrance; this group points toward the reactant and would be parallel with the reacting allenoate moiety. The 3,5-dimethoxyphenyl group is approximately parallel with the horizontal plane of the molecule and far from the reactant. However, the two hydrogen atoms in one of the methylene groups of the phosphepine moiety point toward the reactant. Therefore, the changes in enantioselectivity can mainly be attributed to the different steric repulsion between these two hydrogen atoms and the reactant. The geometries of transition states **TS4** and **TS7** further confirmed this proposal. In transition state **TS7**, the H3 atom in the reactant was found to be very close to the H1 and H2 atoms in the phosphepine moiety of the catalyst, with H1···H3 and H2···H3 distances of 1.95 and 2.52 Å, respectively. In contrast, the H1–O1 and H2–O2 bond lengths in the catalyst are 1.95 and 2.46 Å, respectively, which clearly indicates a weak hydrogen-bonding interaction.

**Figure 12:**
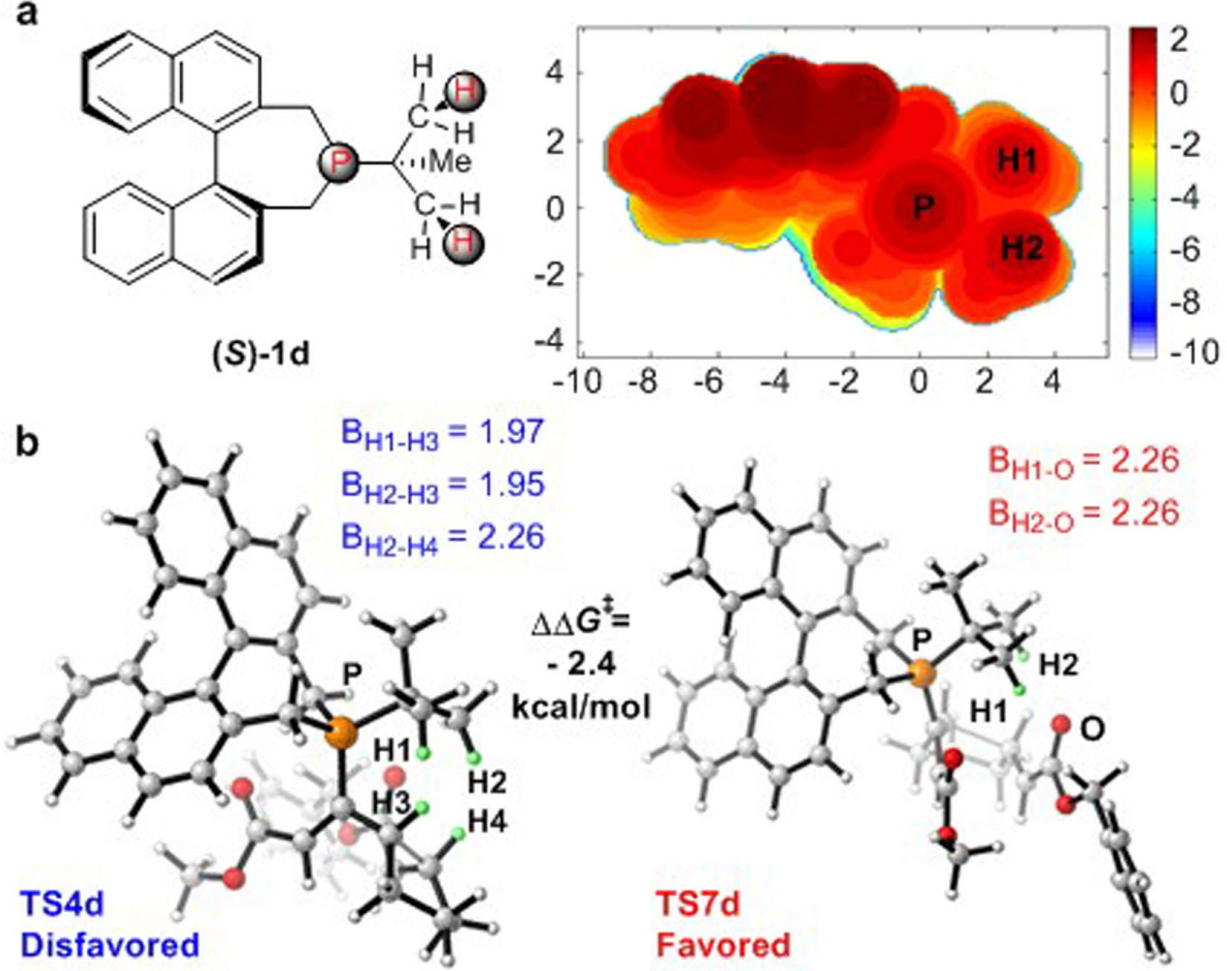
(**a**) 2D contour map of the Van der Waals surface of ligand **(** ***S*** **)-1d**. Distances are given in angstroms. P atom is located at the origin of the coordinate system in the contour map. The contour line of zero is defined as in the same plane of the P atom. Negative distance (blue) indicates that the atoms on the ligand are farther away from the substrate; positive distance (red) indicates that the atoms on the ligand are closer to the substrate. (**b**) Geometries of transition states **TS-4d** and **TS-7d** with catalyst **(** ***S*** **)-1d**. The values of bond lengths are given in angstroms.

We also considered the 2D contour map of catalyst (*S*)-**1d** (Fig. 12a). Without the steric hindrance of the axial phenyl group, the allenoate could rotate to avoid repulsive interactions with the methylene group of the phosphepine moiety. In contrast, the *tert*-butyl group in (*S*)-**1d** is much larger than 3,5-dimethoxyphenyl group in (*S*)-**1a**, which suggests that the *tert*-butyl will offer greater steric hindrance. The arrangement of the steric hindrance around the phosphine is thus changed, which leads to the inverse enantioselectivity. In transition state **TS4d** (Fig. 12b), the H3 atom in the allenoate moiety is close to the H1 and H2 atoms in the *tert*-butyl group of binaphthophosphepine catalyst (*S*)-**1d**, which implies that there is steric repulsion between the reactant and the catalyst. However, in transition state **TS7d**, the atom closest to the H1 and H2 atoms in the catalyst is an oxygen atom. Therefore, transition state **TS4d** is energetically disfavored. These 2D contour maps provide a straightforward explanation for the enantioselectivities observed in the asymmetric syntheses of fused ring systems through phosphine-catalyzed intramolecular [3 + 2] annulation reactions.

Based on our theoretical calculations and previous experimental studies, we found that enlarging the size of substituent groups R^2^ and R^3^ in binaphthophosphepine catalyst (*S*)-**1** resulted in higher enantioselectivities. We therefore designed some new binaphthophosphepine catalysts to further test this hypothesis. As shown in Table 1 (entry 5), changing R^2^ to a 3,5-diisopropylphenyl group increased the calculated ΔΔ*G* ^≠^ to 5.4 kcal/mol. In another example, the R^3^ group was changed to the 3,5-diisopropylphenyl group (entry 6), and in this case the ΔΔ*G* ^≠^ was calculated to be 5.9 kcal/mol. The regression equation shown in Fig. 10 showed that the expected experimental *ee* of these two catalysts was 97.9% and 98.5%, respectively. Interestingly, when we changed R^2^ to an anthracenyl group, the calculated ΔΔ*G* ^≠^ was as high as 7.3 kcal/mol, which gives an extrapolated experimental *ee* of 99.6%. We hope that our predictions will be validated in future experiments.

## Conclusion

In summary, a theoretical study was conducted to reveal the mechanism of phosphine-catalyzed diastereoselective intramolecular [3 + 2] annulations that generate fused ring systems. DFT calculations showed that the reaction proceeds through a nucleophilic addition step that yields a zwitterionic intermediate. There is a low potential energy barrier for the conversion of this zwitterionic intermediate to its geometric isomer, which means that the racemic reactant is converted to the stable *gauche* isomer. The subsequent stepwise [3 + 2] annulation starts with intramolecular nucleophilic addition of the allenoate moiety to the electron-deficient olefin group. The ring-closing reaction then irreversibly yields a phosphorus ylide. The enantioselectivity originates from the first step of the [3 + 2] cycloaddition and is a result of the noticeable disparity in activation free energy for the different attack orientations. Proton transfer is accomplished through a water-assisted direct [1,2]-proton transfer process, which has been confirmed to be either a concert or a stepwise pathway. In addition, based on the structural and thermodynamic studies described above, we performed theoretical calculations on a series of chiral binaphthophosphepine catalysts with the aim of improving the enantioselectivity of [3 + 2] annulations using quantum-chemical methods. The theoretically predicated *ee* values for the experimentally reported binaphthophosphepine catalysts agree well with the experimental observations, which could be explained by steric effects using 2D contour maps. Based on our results, we also designed three new binaphthophosphepine catalysts that are expecting to give higher enantioselectivity in phosphine-catalyzed [3 + 2] cycloadditions. We believe that our study of substituent effects could provide further insight for the design and selection of binaphthophosphepine catalysts, and thus improve the enantioselectivity in this type of reactions.

## Electronic supplementary material

Supplementary Information
