# Supplementary material for: From Mechanistic Study to Chiral Catalyst Optimization: Theoretical Insight into Binaphthophosphepine-catalyzed Asymmetric Intramolecular [3 + 2] Cycloaddition
Source: Sci Rep. 2017 Aug 8;7:7619. doi: 10.1038/s41598-017-07863-9 (PMC5548760; doi:10.1038/s41598-017-07863-9)
Supplement: Supplementary file 1 — Supplementary Information [file 41598_2017_7863_MOESM1_ESM.doc]

**Supplementary Information**

**From Mechanistic Study to Chiral Catalyst Optimization: Theoretical Insight into Binaphthophosphepine-catalyzed Asymmetric Intramolecular [3+2] Cycloaddition**

Meng Duan,^†^ Lei Zhu,^†^ Xiaotian Qi, Zhaoyuan Yu, Yingzi Li, Ruopeng Bai^*^ and Yu Lan^*^

*School of Chemistry and Chemical Engineering, Chongqing University, Chongqing 400030, China.*

E-mail: ruopeng@cqu.edu.cn, lanyu@cqu.edu.cn

**Table of contents**

[1. Complete Reference for Gaussian 09 1](#__RefHeading___Toc483083302)

[2. [1,2]-proton shift process from intermediate **CP15** 2](#__RefHeading___Toc483083303)

[3. Stability and [3+2] cycloaddition pathways from isomers of **CP3/CP4**. 3](#__RefHeading___Toc483083304)

[4. Absolute Calculation Energies, Enthalpies, and Free Energies 5](#__RefHeading___Toc483083305)

[5. B3LYP Geometries for All the Optimized Compounds and Transition State 17](#__RefHeading___Toc483083306)

### 1. Complete Reference for Gaussian 09

Gaussian 09, Revision D.01, Frisch, M. J.; Trucks, G. W.; Schlegel, H. B.; Scuseria, G. E.; Robb, M. A.; Cheeseman, J. R.; Scalmani, G.; Barone, V.; Mennucci, B.; Petersson, G. A.; Nakatsuji, H.; Caricato, M.; Li, X.; Hratchian, H. P.; Izmaylov, A. F.; Bloino, J.; Zheng, G.; Sonnenberg, J. L.; Hada, M.; Ehara, M.; Toyota, K.; Fukuda, R.; Hasegawa, J.; Ishida, M.; Nakajima, T.; Honda, Y.; Kitao, O.; Nakai, H.; Vreven, T.; Montgomery, Jr., J. A.; Peralta, J. E.; Ogliaro, F.; Bearpark, M.; Heyd, J. J.; Brothers, E.; Kudin, K. N.; Staroverov, V. N.; Kobayashi, R.; Normand, J.; Raghavachari, K.; Rendell, A.; Burant, J. C.; Iyengar, S. S.; Tomasi, J.; Cossi, M.; Rega, N.; Millam, N. J.; Klene, M.; Knox, J. E.; Cross, J. B.; Bakken, V.; Adamo, C.; Jaramillo, J.; Gomperts, R.; Stratmann, R. E.; Yazyev, O.; Austin, A. J.; Cammi, R.; Pomelli, C.; Ochterski, J. W.; Martin, R. L.; Morokuma, K.; Zakrzewski, V. G.; Voth, G. A.; Salvador, P.; Dannenberg, J. J.; Dapprich, S.; Daniels, A. D.; Farkas, Ö.; Foresman, J. B.; Ortiz, J. V.; Cioslowski, J.; Fox, D. J. Gaussian, Inc., Wallingford CT, **2013**.

### 2. [1,2]-proton shift process from intermediate CP15

**Figure S1**. Free energy profiles for the [1,2]-proton transfer process. (E^1^ = CO_2_Bn, E^2^ = CO_2_Me).

The Gibbs free energy profiles for the [1,2]-proton transfer of **CP15** were also calculated. As shown in Figure S1, the direct [1,2]-proton shift via transition state **TS20** cannot occur after intermediate **CP15** is formed owing to the very high activation free energy (47.6 kcal/mol). Nevertheless, promotion with water would considerably reduce the barrier of this step. Stepwise proton transfer may start from the protonation of the phosphorus ylide via transition state **TS18** to reversibly yield hydroxyphosphorane intermediate **CP19** with an energy barrier of only 13.2 kcal/mol. This is followed by deprotonation via transition state **TS19** to generate zwitterionic phosphonium intermediate **CP12**, which is then decomposed to the same product **CP13** and regenerates active catalyst **CP1**.

### 3. Stability and [3+2] cycloaddition pathways from isomers of CP3/CP4.

**Figure S2**. Geometries and free energy information of Intermediates of **CP3** (left) and **CP4** (right). The values of bond lengths are given in angstroms. ΔG represent the relative free energies in comparison with **CP1**.

**Figure S3**. Geometries and free energy information of transition states for the nucleophilic addition step and the [3 + 2] cycloaddition step from **CP3-cis** and **CP4-cis**. (E^1^ = CO_2_Bn, E^2^ = CO_2_Me). The values of bond lengths are given in angstroms. ΔG represent the relative free energies in comparison with **CP1**.

We also considered the stability of the phosphoniums with different configuration. As shown in Figure S2, the relative free energy of **CP3-cis** and **CP4-cis** are found to be higher than that of **CP3-trans** and **CP4-trans** respectively. Moreover, the activation free energy of the initial nucleophilic addition steps, leading to the generation of **CP3-cis** and **CP4-cis**, are determined to be 25.6 kcal/mol (**TS21**) and 23.2 kcal/mol(**TS22**), which are 2.7 and 0.3 kcal/mol higher than that of **TS1** (Figure 3) respectively. Despite all this, the subsequent [3+2] pathways from **CP3-cis** and **CP4-cis** are also taken into account in the revised revision. As shown in Figure S3, when **CP3-cis** is formed, the following first intramolecular C–C bond formation could occur via transition state **TS23** and **TS25** to yield corresponding carbon anionic intermediates. The relative free energy of transition state **TS23** is 7.0 kcal/mol higher than that of **TS4** , and the relative free energy of transition state **TS25** are 7.2 kcal/mol higher than that of **TS7** respectively. These energy discrepancies suggest that intramolecular C–C bond formation from **CP3-trans** would occur preferentially. In addition, we also considered the first intramolecular C–C bond formation from intermediate **CP4-cis**. Calculated results also indicate that these pathways are dynamically unfavorable due to the high activation free energy of transition state **TS24** and **TS26**. Based on these results, the possibility of the subsequent [3+2] pathways from **CP3-cis** and **CP4-cis** could be ruled out.

### 4. Absolute Calculation Energies, Enthalpies, and Free Energies

**Table S1. Absolute Calculation Energies, Enthalpies, and Free Energies**

| Geometry | E_(elec-B3LYP)_^1^ | H_(corr-B3LYP)_^2^ | G_(corr-B3LYP)_^3^ | E_(solv-M11)_^4^ | IF^5^ |
| --- | --- | --- | --- | --- | --- |
| **CP1** | -2112.127787 | 0.669416 | 0.56171 | -2111.569704 | - |
| **(*R*)-CP2** | -998.7875966 | 0.368229 | 0.285284 | -998.5553897 | - |
| **TS1** | -3110.898561 | 1.038217 | 0.869736 | -3110.11127 | -157.27 |
| **CP3** | -3110.91382 | 1.039815 | 0.871826 | -3110.135129 | - |
| **TS2** | -3110.894948 | 1.038438 | 0.872518 | -3110.120014 | -27.93 |
| **CP4** | -3110.905452 | 1.039261 | 0.870132 | -3110.129646 | - |
| **TS3** | -3110.893275 | 1.037911 | 0.868581 | -3110.106962 | -144.52 |
| **(*S*)-CP2** | -998.7875966 | 0.368229 | 0.285284 | -998.5553897 | - |
| **TS4** | -3110.900609 | 1.039429 | 0.882271 | -3110.128028 | -350.04 |
| **CP5** | -3110.904649 | 1.041449 | 0.885879 | -3110.136471 | - |
| **TS5** | -3110.904594 | 1.040578 | 0.887262 | -3110.135945 | -48.88 |
| **CP6** | -3110.937006 | 1.04327 | 0.887108 | -3110.176718 | - |
| **TS6** | -3110.872028 | 1.040013 | 0.882535 | -3110.107576 | -37.08 |
| **CP7** | -3110.932842 | 1.043379 | 0.885375 | -3110.177513 | - |
| **TS7** | -3110.895053 | 1.039414 | 0.882501 | -3110.120777 | -331.14 |
| **CP8** | -3110.899695 | 1.041205 | 0.884345 | -3110.134252 | - |
| **TS8** | -3110.898644 | 1.040128 | 0.883566 | -3110.125941 | -14.76 |
| **CP9** | -3110.933956 | 1.043005 | 0.887028 | -3110.181843 | - |
| **H_2_O** | -76.40702399 | 0.024918 | 0.002819 | -76.43937615 | - |
| **TS9** | -3187.324065 | 1.06764 | 0.910427 | -3186.601849 | -67.27 |
| **CP13** | -998.847204 | 0.371345 | 0.296361 | -998.6285733 | - |
| **CP10** | -3187.367051 | 1.071818 | 0.912113 | -3186.631951 | - |
| **TS10** | -3187.343882 | 1.066647 | 0.909666 | -3186.615674 | -185.81 |
| **CP11** | -3187.363929 | 1.0724 | 0.911661 | -3186.629284 | - |
| **TS11** | -3187.33396 | 1.065931 | 0.904567 | -3186.595741 | -922.56 |
| **CP12** | -3110.929486 | 1.04242 | 0.885183 | -3110.175303 | - |
| **TS12** | -3110.928218 | 1.041099 | 0.885378 | -3110.17112 | -133.06 |
| **TS13** | -3110.865657 | 1.038035 | 0.879806 | -3110.101662 | -1507.71 |
| **TS14** | -3110.897687 | 1.039178 | 0.878712 | -3110.124116 | -340.00 |
| **CP14** | -3110.905017 | 1.041003 | 0.880489 | -3110.133212 | - |
| **TS15** | -3110.905117 | 1.040301 | 0.882639 | -3110.13081 | -87.74 |
| **CP15** | -3110.940722 | 1.043491 | 0.885823 | -3110.182268 | - |
| **TS16** | -3110.892695 | 1.039588 | 0.884713 | -3110.121122 | -340.95 |
| **CP16** | -3110.899751 | 1.04149 | 0.88366 | -3110.129932 | - |
| **TS17** | -3110.897704 | 1.040794 | 0.886235 | -3110.126822 | -204.57 |
| **CP17** | -3110.940093 | 1.043238 | 0.884028 | -3110.17615 | - |
| **CP18** | -3187.363809 | 1.071409 | 0.907668 | -3186.626347 |  |
| **TS18** | -3187.344753 | 1.067539 | 0.91127 | -3186.623276 | -117.79 |
| **CP19** | -3187.361398 | 1.071851 | 0.910356 | -3186.62534 | - |
| **TS19** | -3187.341512 | 1.065719 | 0.90678 | -3186.607805 | -853.43 |
| **TS20** | -3110.862424 | 1.03802 | 0.879103 | -3110.09964 | -1495.15 |
| **TS4b** | -2881.854838 | 0.96869 | 0.822471 | -2881.102678 | -351.44 |
| **TS7b** | -2881.849646 | 0.968164 | 0.8193 | -2881.0931 | -329.15 |
| **TS4c** | -2419.761673 | 0.797352 | 0.673369 | -2419.167687 | -351.37 |
| **TS7c** | -2419.759504 | 0.796993 | 0.670113 | -2419.160166 | -339.27 |
| **CP1d** | -1347.192354 | 0.459674 | 0.385239 | -1346.832546 | - |
| **TS4d** | -2345.949468 | 0.830127 | 0.70599 | -2345.381812 | -348.59 |
| **TS7d** | -2345.957625 | 0.829859 | 0.705242 | -2345.384868 | -344.22 |
| **TS4e** | -3353.626168 | 1.325723 | 1.142623 | -3352.671962 | -349.94 |
| **TS7e** | -3353.623337 | 1.325513 | 1.139871 | -3352.660597 | -331.69 |
| **TS4f** | -3117.740091 | 1.147081 | 0.980609 | -3116.888216 | -351.26 |
| **TS7f** | -3117.735524 | 1.146827 | 0.979771 | -3116.877972 | -326.86 |
| **TS4g** | -3496.3918 | 1.166461 | 0.998325 | -3495.423009 | -351.43 |
| **TS7g** | -3496.384116 | 1.165987 | 0.994945 | -3495.407972 | -325.69 |
| **CP3-cis** | -3110.90213 | 1.04013 | 0.874273 | -3110.132461 | - |
| **CP4-cis** | -3110.906447 | 1.040046 | 0.87455 | -3110.133319 | - |
| **TS21** | -3110.891738 | 1.038231 | 0.874086 | -3110.111422 | -179.71 |
| **TS22** | -3110.897593 | 1.038638 | 0.875014 | -3110.116104 | -174.38 |
| **TS23** | -3110.880798 | 1.039406 | 0.882978 | -3110.117612 | -313.72 |
| **TS24** | -3110.882597 | 1.038957 | 0.881274 | -3110.117439 | -384.63 |
| **TS25** | -3110.877027 | 1.039415 | 0.881837 | -3110.10865 | -312.08 |
| **TS26** | -3110.88089 | 1.039675 | 0.882954 | -3110.10916 | -334.77 |

^1^The electronic energy calculated by B3LYP in gas phase. ^2^The thermal correction to enthalpy calculated by B3LYP in gas phase. ^3^ The thermal correction to Gibbs free energy calculated by B3LYP in gas phase. ^4^The electronic energy calculated by M11 in toluene solvent. ^5^The B3LYP calculated imaginary frequencies for the transition states.

**Table S2. Absolute Calculation Energies of The 2D potential energy surface for the annulation step of binaphthophosphepine-catalyzed intramolecular [3+2] cycloaddition reaction**

| R1^1^ | R2^2^ | E_(elec-B3LYP)_^3^ | ΔE^4^ |
| --- | --- | --- | --- |
| 1.6  1.6  1.6  1.6  1.6  1.6  1.6  1.6  1.6  1.6  1.6  1.6  1.6  1.6  1.6  1.6  1.6  1.6  1.6  1.6  1.6  1.65  1.65  1.65  1.65  1.65  1.65  1.65  1.65  1.65  1.65  1.65  1.65  1.65  1.65  1.65  1.65  1.65  1.65  1.65  1.65  1.65  1.7  1.7  1.7  1.7  1.7  1.7  1.7  1.7  1.7  1.7  1.7  1.7  1.7  1.7  1.7  1.7  1.7  1.7  1.7  1.7  1.7  1.75  1.75  1.75  1.75  1.75  1.75  1.75  1.75  1.75  1.75  1.75  1.75  1.75  1.75  1.75  1.75  1.75  1.75  1.75  1.75  1.75  1.8  1.8  1.8  1.8  1.8  1.8  1.8  1.8  1.8  1.8  1.8  1.8  1.8  1.8  1.8  1.8  1.8  1.8  1.8  1.8  1.8  1.85  1.85  1.85  1.85  1.85  1.85  1.85  1.85  1.85  1.85  1.85  1.85  1.85  1.85  1.85  1.85  1.85  1.85  1.85  1.85  1.85  1.9  1.9  1.9  1.9  1.9  1.9  1.9  1.9  1.9  1.9  1.9  1.9  1.9  1.9  1.9  1.9  1.9  1.9  1.9  1.9  1.9  1.95  1.95  1.95  1.95  1.95  1.95  1.95  1.95  1.95  1.95  1.95  1.95  1.95  1.95  1.95  1.95  1.95  1.95  1.95  1.95  1.95  2  2  2  2  2  2  2  2  2  2  2  2  2  2  2  2  2  2  2  2  2  2.05  2.05  2.05  2.05  2.05  2.05  2.05  2.05  2.05  2.05  2.05  2.05  2.05  2.05  2.05  2.05  2.05  2.05  2.05  2.05  2.05  2.1  2.1  2.1  2.1  2.1  2.1  2.1  2.1  2.1  2.1  2.1  2.1  2.1  2.1  2.1  2.1  2.1  2.1  2.1  2.1  2.1  2.15  2.15  2.15  2.15  2.15  2.15  2.15  2.15  2.15  2.15  2.15  2.15  2.15  2.15  2.15  2.15  2.15  2.15  2.15  2.15  2.15  2.2  2.2  2.2  2.2  2.2  2.2  2.2  2.2  2.2  2.2  2.2  2.2  2.2  2.2  2.2  2.2  2.2  2.2  2.2  2.2  2.2  2.25  2.25  2.25  2.25  2.25  2.25  2.25  2.25  2.25  2.25  2.25  2.25  2.25  2.25  2.25  2.25  2.25  2.25  2.25  2.25  2.25  2.3  2.3  2.3  2.3  2.3  2.3  2.3  2.3  2.3  2.3  2.3  2.3  2.3  2.3  2.3  2.3  2.3  2.3  2.3  2.3  2.3  2.35  2.35  2.35  2.35  2.35  2.35  2.35  2.35  2.35  2.35  2.35  2.35  2.35  2.35  2.35  2.35  2.35  2.35  2.35  2.35  2.35  2.4  2.4  2.4  2.4  2.4  2.4  2.4  2.4  2.4  2.4  2.4  2.4  2.4  2.4  2.4  2.4  2.4  2.4  2.4  2.4  2.4  2.45  2.45  2.45  2.45  2.45  2.45  2.45  2.45  2.45  2.45  2.45  2.45  2.45  2.45  2.45  2.45  2.45  2.45  2.45  2.45  2.45  2.5  2.5  2.5  2.5  2.5  2.5  2.5  2.5  2.5  2.5  2.5  2.5  2.5  2.5  2.5  2.5  2.5  2.5  2.5  2.5  2.5 | 2  2.05  2.1  2.15  2.2  2.25  2.3  2.35  2.4  2.45  2.5  2.55  2.6  2.65  2.7  2.75  2.8  2.85  2.9  2.95  3  2  2.05  2.1  2.15  2.2  2.25  2.3  2.35  2.4  2.45  2.5  2.55  2.6  2.65  2.7  2.75  2.8  2.85  2.9  2.95  3  2  2.05  2.1  2.15  2.2  2.25  2.3  2.35  2.4  2.45  2.5  2.55  2.6  2.65  2.7  2.75  2.8  2.85  2.9  2.95  3  2  2.05  2.1  2.15  2.2  2.25  2.3  2.35  2.4  2.45  2.5  2.55  2.6  2.65  2.7  2.75  2.8  2.85  2.9  2.95  3  2  2.05  2.1  2.15  2.2  2.25  2.3  2.35  2.4  2.45  2.5  2.55  2.6  2.65  2.7  2.75  2.8  2.85  2.9  2.95  3  2  2.05  2.1  2.15  2.2  2.25  2.3  2.35  2.4  2.45  2.5  2.55  2.6  2.65  2.7  2.75  2.8  2.85  2.9  2.95  3  2  2.05  2.1  2.15  2.2  2.25  2.3  2.35  2.4  2.45  2.5  2.55  2.6  2.65  2.7  2.75  2.8  2.85  2.9  2.95  3  2  2.05  2.1  2.15  2.2  2.25  2.3  2.35  2.4  2.45  2.5  2.55  2.6  2.65  2.7  2.75  2.8  2.85  2.9  2.95  3  2  2.05  2.1  2.15  2.2  2.25  2.3  2.35  2.4  2.45  2.5  2.55  2.6  2.65  2.7  2.75  2.8  2.85  2.9  2.95  3  2  2.05  2.1  2.15  2.2  2.25  2.3  2.35  2.4  2.45  2.5  2.55  2.6  2.65  2.7  2.75  2.8  2.85  2.9  2.95  3  2  2.05  2.1  2.15  2.2  2.25  2.3  2.35  2.4  2.45  2.5  2.55  2.6  2.65  2.7  2.75  2.8  2.85  2.9  2.95  3  2  2.05  2.1  2.15  2.2  2.25  2.3  2.35  2.4  2.45  2.5  2.55  2.6  2.65  2.7  2.75  2.8  2.85  2.9  2.95  3  2  2.05  2.1  2.15  2.2  2.25  2.3  2.35  2.4  2.45  2.5  2.55  2.6  2.65  2.7  2.75  2.8  2.85  2.9  2.95  3  2  2.05  2.1  2.15  2.2  2.25  2.3  2.35  2.4  2.45  2.5  2.55  2.6  2.65  2.7  2.75  2.8  2.85  2.9  2.95  3  2  2.05  2.1  2.15  2.2  2.25  2.3  2.35  2.4  2.45  2.5  2.55  2.6  2.65  2.7  2.75  2.8  2.85  2.9  2.95  3  2  2.05  2.1  2.15  2.2  2.25  2.3  2.35  2.4  2.45  2.5  2.55  2.6  2.65  2.7  2.75  2.8  2.85  2.9  2.95  3  2  2.05  2.1  2.15  2.2  2.25  2.3  2.35  2.4  2.45  2.5  2.55  2.6  2.65  2.7  2.75  2.8  2.85  2.9  2.95  3  2  2.05  2.1  2.15  2.2  2.25  2.3  2.35  2.4  2.45  2.5  2.55  2.6  2.65  2.7  2.75  2.8  2.85  2.9  2.95  3  2  2.05  2.1  2.15  2.2  2.25  2.3  2.35  2.4  2.45  2.5  2.55  2.6  2.65  2.7  2.75  2.8  2.85  2.9  2.95  3 | -3110.91159  -3110.90956  -3110.908  -3110.90682  -3110.90592  -3110.90525  -3110.90477  -3110.90442  -3110.90419  -3110.90403  -3110.90392  -3110.90383  -3110.90376  -3110.90367  -3110.90357  -3110.90343  -3110.90325  -3110.90302  -3110.90273  -3110.9024  -3110.90201  -3110.9111  -3110.90922  -3110.90785  -3110.90681  -3110.90605  -3110.9055  -3110.90512  -3110.90488  -3110.90473  -3110.90466  -3110.90462  -3110.90459  -3110.90457  -3110.90452  -3110.90445  -3110.90433  -3110.90417  -3110.90395  -3110.90368  -3110.90335  -3110.90296  -3110.90951  -3110.90781  -3110.90662  -3110.90574  -3110.90512  -3110.90469  -3110.90443  -3110.90429  -3110.90425  -3110.90426  -3110.9043  -3110.90435  -3110.90438  -3110.90438  -3110.90435  -3110.90426  -3110.90412  -3110.90392  -3110.90367  -3110.90335  -3110.90298  -3110.90712  -3110.90566  -3110.90464  -3110.90393  -3110.90346  -3110.90317  -3110.90304  -3110.90303  -3110.90309  -3110.9032  -3110.90333  -3110.90345  -3110.90354  -3110.9036  -3110.90361  -3110.90356  -3110.90346  -3110.90328  -3110.90305  -3110.90275  -3110.9024  -3110.90423  -3110.90301  -3110.90218  -3110.90165  -3110.90134  -3110.90122  -3110.90123  -3110.90135  -3110.90153  -3110.90175  -3110.90197  -3110.90217  -3110.90234  -3110.90246  -3110.90252  -3110.90252  -3110.90245  -3110.90231  -3110.9021  -3110.90183  -3110.9015  -3110.90105  -3110.90008  -3110.89945  -3110.89912  -3110.899  -3110.89904  -3110.89922  -3110.89948  -3110.89979  -3110.90013  -3110.90045  -3110.90074  -3110.90099  -3110.90118  -3110.9013  -3110.90135  -3110.90132  -3110.90122  -3110.90105  -3110.90081  -3110.9005  -3110.89778  -3110.89704  -3110.89664  -3110.89651  -3110.89658  -3110.89681  -3110.89716  -3110.89758  -3110.89804  -3110.8985  -3110.89894  -3110.89933  -3110.89966  -3110.89992  -3110.90011  -3110.90021  -3110.90024  -3110.90018  -3110.90005  -3110.89984  -3110.89957  -3110.89453  -3110.89403  -3110.89386  -3110.89395  -3110.89423  -3110.89466  -3110.89519  -3110.89578  -3110.89639  -3110.89699  -3110.89755  -3110.89805  -3110.89847  -3110.89882  -3110.89907  -3110.89924  -3110.89932  -3110.89931  -3110.89922  -3110.89906  -3110.89882  -3110.8914  -3110.89115  -3110.89122  -3110.89154  -3110.89203  -3110.89267  -3110.8934  -3110.89417  -3110.89494  -3110.89568  -3110.89637  -3110.89698  -3110.8975  -3110.89793  -3110.89826  -3110.89849  -3110.89863  -3110.89867  -3110.89863  -3110.89852  -3110.89833  -3110.88846  -3110.88847  -3110.88879  -3110.88934  -3110.89007  -3110.89092  -3110.89185  -3110.8928  -3110.89374  -3110.89463  -3110.89544  -3110.89616  -3110.89679  -3110.89731  -3110.89772  -3110.89802  -3110.89821  -3110.89831  -3110.89833  -3110.89825  -3110.89811  -3110.88576  -3110.88604  -3110.88661  -3110.88741  -3110.88837  -3110.88945  -3110.89058  -3110.89172  -3110.89282  -3110.89385  -3110.8948  -3110.89563  -3110.89636  -3110.89696  -3110.89745  -3110.89782  -3110.89808  -3110.89823  -3110.89829  -3110.89827  -3110.89817  -3110.88332  -3110.88387  -3110.88471  -3110.88576  -3110.88696  -3110.88826  -3110.88959  -3110.89091  -3110.89217  -3110.89335  -3110.89443  -3110.89537  -3110.89619  -3110.89689  -3110.89745  -3110.89789  -3110.8982  -3110.89841  -3110.89853  -3110.89855  -3110.8985  -3110.88116  -3110.88199  -3110.88309  -3110.88439  -3110.88583  -3110.88734  -3110.88887  -3110.89036  -3110.89179  -3110.89311  -3110.8943  -3110.89536  -3110.89628  -3110.89705  -3110.89769  -3110.8982  -3110.89857  -3110.89883  -3110.89899  -3110.89907  -3110.89906  -3110.87928  -3110.88038  -3110.88175  -3110.88329  -3110.88496  -3110.88668  -3110.88839  -3110.89005  -3110.89162  -3110.89307  -3110.89438  -3110.89555  -3110.89656  -3110.89742  -3110.89813  -3110.8987  -3110.89913  -3110.89944  -3110.89965  -3110.89977  -3110.89981  -3110.87767  -3110.87904  -3110.88066  -3110.88244  -3110.88433  -3110.88624  -3110.88813  -3110.88994  -3110.89164  -3110.89321  -3110.89463  -3110.89589  -3110.89698  -3110.89792  -3110.8987  -3110.89934  -3110.89983  -3110.9002  -3110.90045  -3110.90062  -3110.9007  -3110.87631  -3110.87794  -3110.8798  -3110.88181  -3110.88389  -3110.88598  -3110.88802  -3110.88997  -3110.89179  -3110.89347  -3110.89498  -3110.89633  -3110.89751  -3110.89852  -3110.89937  -3110.90006  -3110.90061  -3110.90103  -3110.90134  -3110.90155  -3110.90167  -3110.87519  -3110.87707  -3110.87915  -3110.88135  -3110.88362  -3110.88586  -3110.88804  -3110.89011  -3110.89204  -3110.89381  -3110.89541  -3110.89683  -3110.89808  -3110.89915  -3110.90006  -3110.90081  -3110.90141  -3110.90189  -3110.90224  -3110.9025  -3110.90267  -3110.87427  -3110.87637  -3110.87865  -3110.88104  -3110.88345  -3110.88584  -3110.88813  -3110.89031  -3110.89233  -3110.89418  -3110.89585  -3110.89734  -3110.89865  -3110.89978  -3110.90074  -3110.90154  -3110.9022  -3110.90272  -3110.90312  -3110.90342  -3110.90363  -3110.87351  -3110.87582  -3110.87827  -3110.88081  -3110.88336  -3110.88587  -3110.88826  -3110.89053  -3110.89263  -3110.89455  -3110.89628  -3110.89783  -3110.89919  -3110.90037  -3110.90138  -3110.90223  -3110.90293  -3110.90349  -3110.90394  -3110.90428  -3110.90452 | 1.399519525  2.673344525  3.652244525  4.392694525  4.957444525  5.377869525  5.679069525  5.898694525  6.043019525  6.143419525  6.212444525  6.268919525  6.312844525  6.369319525  6.432069525  6.519919525  6.632869525  6.777194525  6.959169525  7.166244525  7.410969525  1.706994525  2.886694525  3.746369525  4.398969525  4.875869525  5.220994525  5.459444525  5.610044525  5.704169525  5.748094525  5.773194525  5.792019525  5.804569525  5.835944525  5.879869525  5.955169525  6.055569525  6.193619525  6.363044525  6.570119525  6.814844525  2.704719525  3.771469525  4.518194525  5.070394525  5.459444525  5.729269525  5.892419525  5.980269525  6.005369525  5.999094525  5.973994525  5.942619525  5.923794525  5.923794525  5.942619525  5.999094525  6.086944525  6.212444525  6.369319525  6.570119525  6.802294525  4.204444525  5.120594525  5.760644525  6.206169525  6.501094525  6.683069525  6.764644525  6.770919525  6.733269525  6.664244525  6.582669525  6.507369525  6.450894525  6.413244525  6.406969525  6.438344525  6.501094525  6.614044525  6.758369525  6.946619525  7.166244525  6.017919525  6.783469525  7.304294525  7.636869525  7.831394525  7.906694525  7.900419525  7.825119525  7.712169525  7.574119525  7.436069525  7.310569525  7.203894525  7.128594525  7.090944525  7.090944525  7.134869525  7.222719525  7.354494525  7.523919525  7.730994525  8.013369525  8.622044525  9.017369525  9.224444525  9.299744525  9.274644525  9.161694525  8.998544525  8.804019525  8.590669525  8.389869525  8.207894525  8.051019525  7.931794525  7.856494525  7.825119525  7.843944525  7.906694525  8.013369525  8.163969525  8.358494525  10.06529453  10.52964453  10.78064453  10.86221953  10.81829453  10.67396952  10.45434453  10.19079453  9.902144525  9.613494525  9.337394525  9.092669525  8.885594525  8.722444525  8.603219525  8.540469525  8.521644525  8.559294525  8.640869525  8.772644525  8.942069525  12.10466953  12.41841953  12.52509452  12.46861952  12.29291953  12.02309453  11.69051952  11.32029453  10.93751953  10.56101952  10.20961952  9.895869525  9.632319525  9.412694525  9.255819525  9.149144525  9.098944525  9.105219525  9.161694525  9.262094525  9.412694525  14.06874453  14.22561953  14.18169453  13.98089453  13.67341953  13.27181952  12.81374453  12.33056953  11.84739452  11.38304453  10.95006953  10.56729453  10.24099453  9.971169525  9.764094525  9.619769525  9.531919525  9.506819525  9.531919525  9.600944525  9.720169525  15.91359452  15.90731953  15.70651953  15.36139452  14.90331953  14.36994453  13.78636953  13.19024453  12.60039453  12.04191953  11.53364453  11.08184453  10.68651953  10.36021953  10.10294453  9.914694525  9.795469525  9.732719525  9.720169525  9.770369525  9.858219525  17.60784453  17.43214453  17.07446953  16.57246953  15.97006952  15.29236952  14.58329452  13.86794453  13.17769453  12.53136953  11.93524453  11.41441953  10.95634452  10.57984453  10.27236953  10.04019452  9.877044525  9.782919525  9.745269525  9.757819525  9.820569525  19.13894453  18.79381953  18.26671953  17.60784453  16.85484453  16.03909452  15.20451952  14.37621953  13.58556953  12.84511953  12.16741953  11.57756952  11.06301953  10.62376953  10.27236953  9.996269525  9.801744525  9.669969525  9.594669525  9.582119525  9.613494525  20.49434453  19.97351953  19.28326953  18.46751952  17.56391952  16.61639452  15.65631952  14.72134453  13.82401952  12.99571953  12.24899453  11.58384453  11.00654453  10.52336953  10.12176953  9.801744525  9.569569525  9.406419525  9.306019525  9.255819525  9.262094525  21.67404453  20.98379453  20.12411953  19.15776952  18.10984453  17.03054453  15.95751953  14.91586953  13.93069453  13.02081953  12.19879452  11.46461952  10.83084452  10.29119453  9.845669525  9.487994525  9.218169525  9.023644525  8.891869525  8.816569525  8.791469525  22.68431953  21.82464453  20.80809453  19.69114453  18.50516953  17.30664453  16.12066953  14.98489453  13.91814453  12.93296952  12.04191953  11.25126953  10.56729453  9.977444525  9.487994525  9.086394525  8.778919525  8.546744525  8.389869525  8.283194525  8.232994525  23.53771953  22.51489453  21.34774452  20.08646953  18.78126953  17.46979453  16.18969453  14.96606952  13.82401952  12.76981953  11.82229453  10.97516953  10.23471953  9.600944525  9.067569525  8.634594525  8.289469525  8.025919525  7.831394525  7.699619525  7.624319525  24.24051952  23.06081953  21.75561952  20.37511953  18.95069453  17.54509453  16.17714453  14.87821953  13.66714452  12.55646953  11.55246953  10.66141952  9.877044525  9.205619525  8.634594525  8.163969525  7.787469525  7.486269525  7.266644525  7.103494525  6.996819525  24.81781953  23.50006953  22.06936953  20.56964452  19.05736953  17.55764453  16.12066953  14.75271952  13.48516952  12.32429453  11.27636953  10.34139453  9.519369525  8.810294525  8.207894525  7.705894525  7.291744525  6.965444525  6.714444525  6.526194525  6.394419525  25.29471953  23.84519452  22.30781952  20.71396953  19.11384453  17.53881953  16.03909452  14.61466952  13.29691953  12.09211953  11.00654453  10.03391953  9.180519525  8.440069525  7.806294525  7.272919525  6.833669525  6.482269525  6.199894525  5.986544525  5.835944525 |

^1^The bond length of C1-C2, values are given by angstrom. ^2^The bond length of C3-C4, values are given by angstrom.^3^The electronic energy calculated by B3LYP in gas phase.^4^Relative zero is the electronic energy of **CP3**, values are given by kcal/mol.

### 5. B3LYP Geometries for All the Optimized Compounds and Transition State

**CP1**

C -2.22571300 -0.73680000 0.06609900

C -2.31487200 0.63337700 -0.18154600

C -3.52017200 1.20489000 -0.70784900

C -4.65669400 0.35706900 -0.90445300

C -4.54882300 -1.01736500 -0.58653600

C -3.37020100 -1.58083400 -0.13306100

H -2.77852000 3.22943800 -0.94336100

C -3.63628100 2.57782100 -1.06766200

C -5.86003300 0.90932000 -1.42289900

H -5.41704200 -1.65461700 -0.73534500

C -5.94105300 2.24294500 -1.75051500

C -4.81331500 3.08162800 -1.57557000

H -6.71546500 0.25221400 -1.56180400

H -6.86455200 2.65435300 -2.14895600

H -4.87723000 4.13187600 -1.84750400

C -1.13162100 1.51438300 0.10342200

C -1.19130000 2.45151300 1.18953600

C 0.02726400 1.38661300 -0.66505100

C -2.31429600 2.56501900 2.05814900

C -0.05952600 3.29078800 1.44194100

C 1.13917100 2.26632000 -0.43130400

C -2.32025100 3.46777100 3.09866600

H -3.17280000 1.92258800 1.89848600

C -0.10013200 4.21934800 2.51744400

C 1.07368900 3.18099100 0.60181900

C -1.20612900 4.31009600 3.33052200

H -3.18773800 3.53241300 3.75009500

H 0.76623700 4.85430700 2.68810700

H 1.91042300 3.85549900 0.76668900

H -1.22599400 5.02049300 4.15256200

C 2.35971600 2.27778100 -1.29241500

C 2.27527900 2.55676100 -2.66736700

C 3.63038900 2.08392500 -0.72906000

C 3.42470300 2.63370700 -3.45436300

H 1.30139400 2.73456300 -3.11623200

C 4.78173000 2.16243300 -1.51442100

H 3.70980600 1.84847400 0.32824000

C 4.68344300 2.43743800 -2.88014400

H 3.33755700 2.86008000 -4.51398200

H 5.75499700 2.00005700 -1.05916300

H 5.57982600 2.50402800 -3.49124000

C -3.36860000 -3.04326100 0.17557400

C -4.30651200 -3.55652300 1.08770100

C -2.49486300 -3.94330900 -0.45727900

C -4.36696500 -4.92283400 1.36411000

H -4.98365500 -2.87152700 1.59110600

C -2.55559100 -5.30982700 -0.18064400

H -1.77236800 -3.57258000 -1.17872400

C -3.49040300 -5.80538000 0.73060100

H -5.09682700 -5.29607600 2.07795800

H -1.87309500 -5.98875800 -0.68526400

H -3.53518400 -6.86989200 0.94488400

C 0.11398000 0.26982100 -1.67554400

H -0.78009700 0.23759500 -2.30663100

C -0.89816700 -1.28789500 0.53377500

H -1.01044700 -2.26690700 1.00526700

P 0.24847000 -1.48378300 -0.94810200

C 1.93300100 -1.48559700 -0.18113700

C 3.02489100 -1.53080400 -1.07411700

C 2.16120400 -1.54317000 1.19123700

C 4.32430300 -1.59985000 -0.57525300

H 2.84451800 -1.50979300 -2.14297900

C 3.47642200 -1.63406100 1.68423000

H 1.35592500 -1.52564000 1.91600800

C 4.55929900 -1.65526900 0.81012200

H 5.58493100 -1.72349700 1.15056500

O 5.45079800 -1.62934000 -1.34636200

O 3.58567100 -1.69302900 3.04383900

C 4.88190600 -1.79176900 3.60896300

H 5.50002100 -0.91920700 3.35789800

H 4.73590400 -1.83104200 4.69015300

H 5.39815600 -2.70302000 3.27867000

C 5.29924700 -1.59474400 -2.75739100

H 6.31066100 -1.64075900 -3.16648800

H 4.72322300 -2.45583800 -3.12128600

H 4.81538200 -0.66657200 -3.08601300

H 0.97113900 0.38350600 -2.34126200

H -0.45035100 -0.60387600 1.26117000

**(*R*)-CP2**

C -3.59956100 1.87713700 0.07404600

H -3.74804500 0.82231500 0.33601100

H -3.34556900 1.89918600 -0.99484900

C -4.91164000 2.65453300 0.30560400

H -5.18430900 2.61793900 1.36797400

H -4.73763100 3.71335900 0.05852800

C -2.42912100 2.45026400 0.89290700

H -2.66481700 2.41530200 1.96244600

H -2.29089500 3.50956500 0.63188600

C -0.44686700 1.06895300 1.53767400

C 0.23882200 0.42258300 2.45487200

C -1.13003000 1.71706300 0.63483000

H -0.74918600 1.73600200 -0.38836800

H 0.05841900 -0.63103900 2.65711100

C 1.29459300 1.09087700 3.25917100

O 1.63536600 2.25269500 3.18351000

O 1.84691800 0.20023100 4.12142800

C 2.88494600 0.72925100 4.95763900

H 3.71234600 1.10915300 4.35175900

H 3.21555100 -0.10452600 5.57817800

H 2.50190300 1.54308100 5.57979500

C -6.03938500 2.13865300 -0.53620100

H -5.90514000 2.16511500 -1.61798200

C -7.18988600 1.63679700 -0.06975800

H -7.40380900 1.57099000 0.99367500

C -8.23406400 1.14700800 -0.99853400

O -8.16521100 1.14392500 -2.21330200

O -9.30797000 0.68702700 -0.30981800

C -10.40052600 0.19409700 -1.11844700

H -10.66850700 0.97194500 -1.84235400

H -10.06022300 -0.67621700 -1.68852400

C -11.55185100 -0.15333000 -0.21089400

C -12.06799800 0.79658800 0.68175000

C -12.14278000 -1.41952000 -0.26850200

C -13.15195200 0.48311900 1.49976900

H -11.60835500 1.77963400 0.73951200

C -13.23537200 -1.73338700 0.54367100

H -11.74571500 -2.16593900 -0.95269200

C -13.74108400 -0.78295400 1.43060000

H -13.54060200 1.22705500 2.19024900

H -13.68467000 -2.72125200 0.48667300

H -14.58824000 -1.02556400 2.06657800

**TS1**

C 3.64190500 1.85632800 0.29262100

C 4.56413300 1.17425500 -0.49988800

C 5.27191800 1.85597400 -1.54446200

C 5.04195700 3.25607700 -1.73521200

C 4.13206200 3.92461900 -0.88157100

C 3.43705800 3.26566800 0.11421900

H 6.34704100 0.13304500 -2.30686100

C 6.17539600 1.19701200 -2.42576700

C 5.73352500 3.94392400 -2.76929800

H 3.98909000 4.99485800 -1.00881400

C 6.60771700 3.27814400 -3.59706700

C 6.82402800 1.88947400 -3.42426000

H 5.55310400 5.00877700 -2.89629300

H 7.12881300 3.81235900 -4.38682300

H 7.50695000 1.36540800 -4.08740600

C 4.79963000 -0.29060400 -0.26509900

C 6.03935100 -0.72140100 0.31586400

C 3.79770800 -1.21198700 -0.57300800

C 7.06613000 0.18155500 0.71412000

C 6.24859000 -2.11971000 0.53886500

C 4.03475800 -2.61605300 -0.38254800

C 8.23400200 -0.27688700 1.28292000

H 6.91906500 1.24599500 0.56981900

C 7.46788100 -2.56185900 1.12034200

C 5.23294600 -3.03145300 0.16409100

C 8.44317300 -1.66255800 1.48497400

H 9.00201400 0.43177300 1.58114500

H 7.61238700 -3.62851400 1.27548700

H 5.41343100 -4.09537300 0.29684400

H 9.37071500 -2.01050300 1.93138100

C 3.05038800 -3.66344300 -0.79094200

C 2.69963400 -3.83646800 -2.14016400

C 2.50847500 -4.54127100 0.15974400

C 1.82957000 -4.85609500 -2.52601000

H 3.12543300 -3.17572600 -2.89091200

C 1.63652100 -5.56116200 -0.22520100

H 2.75953900 -4.40798300 1.20816400

C 1.29470800 -5.72246100 -1.56920400

H 1.57611900 -4.97926600 -3.57573600

H 1.21921400 -6.22456900 0.52747700

H 0.61840600 -6.51821500 -1.86996800

C 2.51776700 4.06310400 0.98018600

C 2.72863700 4.16665500 2.36570500

C 1.45375200 4.78136400 0.41198900

C 1.89553700 4.95601600 3.15912300

H 3.56508000 3.64020400 2.81848400

C 0.62176800 5.57400200 1.20488600

H 1.27432300 4.70421200 -0.65648100

C 0.83769800 5.66171100 2.58136200

H 2.07944700 5.02728900 4.22789700

H -0.19654300 6.12225600 0.74516300

H 0.18968100 6.27763400 3.19901000

C 2.44846600 -0.71346300 -1.03294900

H 2.52879400 0.02211200 -1.84281100

C 2.79895100 1.05477000 1.25976400

H 2.30322400 1.68523200 1.99911700

P 1.46727300 0.15247700 0.29582500

C 0.82484800 -1.14386600 1.41589000

C -0.15375700 -2.00270700 0.87776100

C 1.20464400 -1.24222900 2.75463100

C -0.71559700 -2.98184500 1.69703900

H -0.45761100 -1.88848900 -0.15748300

C 0.61351400 -2.22363100 3.57010300

H 1.94788000 -0.59339100 3.20295300

C -0.33837600 -3.09604900 3.04792100

H -0.81406000 -3.86349000 3.64593800

O -1.65316200 -3.87582500 1.28137700

O 1.04715100 -2.24254100 4.86531900

C 0.48933500 -3.20459600 5.74491300

H 0.69451900 -4.22896700 5.40621300

H 0.96981400 -3.04142600 6.71156500

H -0.59562300 -3.07330500 5.85180700

C -2.07893100 -3.80790900 -0.07834500

H -2.82456500 -4.59743400 -0.19116700

H -2.53055300 -2.83652700 -0.31002800

H -1.24460400 -3.98095000 -0.76715600

H 1.80627900 -1.51456800 -1.40213700

H 3.41071100 0.31728500 1.79004000

C -3.39901800 2.00361100 0.31181400

H -3.51042800 0.95172000 0.02266900

H -3.22466000 2.01143200 1.39684200

C -4.70277100 2.76605000 -0.00379700

H -4.89189600 2.74833400 -1.08461400

H -4.56612900 3.82197400 0.27789100

C -2.17983200 2.60305900 -0.41373900

H -2.33593200 2.57372800 -1.49691700

H -2.07992400 3.66168300 -0.12679300

C -0.16541400 1.22257400 -0.95902600

C -0.05256000 0.98119100 -2.31064100

C -0.89766600 1.88318300 -0.06737300

H -0.60878500 1.90862200 0.98174800

H 0.38076100 1.72987200 -2.96884500

C -0.37116200 -0.30358700 -2.86847800

O -0.72370400 -1.31695100 -2.25610400

O -0.23021600 -0.31810200 -4.23309900

C -0.54087000 -1.56050600 -4.86079300

H -1.58964300 -1.83307500 -4.70280400

H -0.34838600 -1.41063400 -5.92525200

H 0.08634500 -2.36904000 -4.47117700

C -5.88369400 2.21632900 0.73672700

H -5.83646800 2.22595300 1.82611300

C -6.98306400 1.69993800 0.17191900

H -7.10816200 1.64683500 -0.90620300

C -8.08701500 1.17279600 1.00406300

O -8.12151900 1.15168400 2.22028600

O -9.09039400 0.69841200 0.22213100

C -10.22945400 0.16389100 0.93059300

H -10.56623000 0.91369500 1.65573500

H -9.91761400 -0.71798600 1.49967900

C -11.30560500 -0.17566200 -0.06836900

C -11.72832900 0.76936800 -1.01357600

C -11.92552800 -1.42905600 -0.04073400

C -12.74991400 0.46409300 -1.91133400

H -11.24366000 1.74114400 -1.04915300

C -12.95598900 -1.73424600 -0.93328100

H -11.59991400 -2.17259800 0.68324100

C -13.36959200 -0.78848200 -1.87152600

H -13.06534700 1.20418800 -2.64214900

H -13.42821600 -2.71246400 -0.89874600

H -14.16772900 -1.02479900 -2.57021200

**CP3**

C 2.72004300 1.90407600 0.00218100

C 3.95192100 1.47129300 -0.48789800

C 4.75529200 2.33243600 -1.30700800

C 4.29835900 3.66395000 -1.56151700

C 3.08984400 4.09750000 -0.96802500

C 2.30144900 3.26368000 -0.19716800

H 6.32591900 0.90006700 -1.74237400

C 5.97453700 1.91200700 -1.90980500

C 5.07614200 4.52596600 -2.38163000

H 2.78606800 5.13180800 -1.10686600

C 6.25255600 4.08926700 -2.94526400

C 6.70071900 2.76698200 -2.70912000

H 4.71820500 5.53708300 -2.55999100

H 6.83728200 4.75347000 -3.57568900

H 7.62502300 2.42345400 -3.16545800

C 4.44689400 0.09237000 -0.16001900

C 5.54767700 -0.05880900 0.74911400

C 3.81875700 -1.02369300 -0.70992200

C 6.17811600 1.04051300 1.39857100

C 6.01678100 -1.37772300 1.04683700

C 4.32098200 -2.34173100 -0.43664100

C 7.22095500 0.84273600 2.27667900

H 5.82547300 2.04626800 1.19994700

C 7.10093800 -1.54794700 1.95038900

C 5.39230200 -2.48451400 0.42428900

C 7.69350700 -0.46283200 2.55365100

H 7.68491800 1.69669700 2.76273300

H 7.44991900 -2.55652400 2.15880300

H 5.78311500 -3.47969700 0.62063000

H 8.51957400 -0.60366800 3.24527800

C 3.76376700 -3.57518800 -1.06733400

C 3.74984900 -3.73861800 -2.46217400

C 3.31327200 -4.63543600 -0.26504300

C 3.29656200 -4.92553900 -3.03685700

H 4.11104000 -2.93637500 -3.09978900

C 2.86122200 -5.82432500 -0.83891800

H 3.30521800 -4.51666700 0.81485900

C 2.85227900 -5.97355800 -2.22745000

H 3.29594400 -5.03266200 -4.11805200

H 2.51069800 -6.63100800 -0.20064800

H 2.50307900 -6.89992200 -2.67583500

C 1.09079700 3.85502200 0.44433000

C 0.91073700 3.81555600 1.83783600

C 0.14753000 4.55122300 -0.32875300

C -0.18661400 4.43651200 2.43620800

H 1.65093500 3.31850600 2.45989800

C -0.94502200 5.17982900 0.26949800

H 0.26928000 4.58446800 -1.40762000

C -1.11987500 5.12029100 1.65410200

H -0.30411500 4.39755300 3.51589300

H -1.66307100 5.71293600 -0.34806300

H -1.97145100 5.60811300 2.12036400

C 2.57129500 -0.82684500 -1.53900500

H 2.67302000 -0.01458100 -2.26757000

C 1.82800300 0.89217700 0.69601200

H 1.00408700 1.36373100 1.22972200

P 1.11647200 -0.33636300 -0.51008400

C 0.50899100 -1.74351000 0.48168500

C -0.02322400 -2.84561000 -0.21119500

C 0.53148500 -1.70678100 1.87605100

C -0.52235000 -3.91966800 0.52673900

H -0.03392900 -2.83150300 -1.29605500

C 0.01762500 -2.79684700 2.60055000

H 0.92097100 -0.86881600 2.44047100

C -0.50435700 -3.90168200 1.93307400

H -0.90890300 -4.75840000 2.45796800

O -1.05485000 -5.03898400 -0.02989400

O 0.07854300 -2.67204600 3.95769300

C -0.44281900 -3.72548600 4.75262200

H 0.09475300 -4.66685800 4.57780000

H -0.30086500 -3.41598700 5.78965500

H -1.51278800 -3.88148300 4.56269700

C -1.11732500 -5.10898600 -1.45391500

H -1.58991400 -6.06703000 -1.67863200

H -1.72084000 -4.29289600 -1.86771700

H -0.11680300 -5.07321400 -1.89898600

H 2.25565400 -1.70931200 -2.09290200

H 2.40686100 0.30851800 1.41863300

C -3.43104800 1.98007200 -0.33388300

H -3.67955200 0.98814100 0.06457500

H -3.08283800 2.58147300 0.51759300

C -4.70084800 2.62493400 -0.92623300

H -5.07227100 2.02099100 -1.76367300

H -4.42808700 3.60814500 -1.34157400

C -2.29780200 1.85197700 -1.36546500

H -2.63056200 1.25279700 -2.22449600

H -2.07911500 2.85661100 -1.76738700

C -0.20741100 0.44214200 -1.51516100

C -0.23304800 0.26818800 -2.94051100

C -1.06134900 1.22338100 -0.78693400

H -0.87815700 1.42726200 0.26506800

H -0.63651200 1.08211500 -3.53242300

C 0.05412500 -0.93222600 -3.60832600

O 0.41110600 -2.02976300 -3.11496500

O -0.08587500 -0.82493800 -4.98122500

C 0.15485200 -2.01846700 -5.71171400

H -0.52166200 -2.82467800 -5.40537300

H -0.02061100 -1.76552200 -6.76066200

H 1.18408600 -2.37302400 -5.58259000

C -5.78019700 2.81624300 0.09429600

H -5.53584500 3.42323400 0.96682200

C -7.01301000 2.29468800 0.03471200

H -7.33876800 1.67512500 -0.79641000

C -7.99520800 2.54747000 1.11075200

O -7.80517300 3.20535400 2.11710800

O -9.17675400 1.93728100 0.83332200

C -10.21945100 2.12726400 1.81276100

H -10.29693100 3.19880100 2.02978900

H -9.93274700 1.62940500 2.74509200

C -11.50845500 1.56985800 1.26512400

C -11.95724400 1.92792500 -0.01369700

C -12.29705000 0.71794800 2.04517400

C -13.17028700 1.44229900 -0.49921800

H -11.34449600 2.57926200 -0.63066400

C -13.51746300 0.23721600 1.56446000

H -11.95413600 0.42740500 3.03568400

C -13.95624400 0.59778800 0.29038400

H -13.50493500 1.72381300 -1.49427000

H -14.11892400 -0.42378600 2.18288000

H -14.90288200 0.22160500 -0.08836100

**TS2**

C 0.58423600 1.77766200 1.29140600

C 0.83176000 2.43877900 0.08940100

C -0.20571300 3.19010300 -0.55784100

C -1.47944900 3.30347300 0.08416300

C -1.66027500 2.70577200 1.35337700

C -0.67144300 1.96115700 1.96892700

H 0.91414400 3.71560800 -2.34145600

C -0.03795000 3.80529100 -1.83069200

C -2.52146500 4.03350500 -0.54943200

H -2.60276900 2.86385400 1.87128800

C -2.32447600 4.61823100 -1.77896100

C -1.07063400 4.49626500 -2.42516400

H -3.48082900 4.11544100 -0.04423100

H -3.12858400 5.16932100 -2.25863800

H -0.92293900 4.95037600 -3.40113000

C 2.19648700 2.39924700 -0.53826900

C 3.00078900 3.58857300 -0.50244200

C 2.67005900 1.23659300 -1.14487500

C 2.60581600 4.77557200 0.17761400

C 4.27537800 3.57612300 -1.15186800

C 3.91916400 1.25472400 -1.85643800

C 3.42326400 5.88420800 0.20290700

H 1.65009500 4.80038100 0.68874700

C 5.09127200 4.73967300 -1.11736500

C 4.68156000 2.40802300 -1.83794700

C 4.67724300 5.87159300 -0.45423300

H 3.10310000 6.77719600 0.73281100

H 6.05340100 4.71508100 -1.62348800

H 5.61551900 2.43323800 -2.39323500

H 5.30960400 6.75480600 -0.42988900

C 4.41186100 0.11827500 -2.68863600

C 3.60653700 -0.46132200 -3.68464400

C 5.74028200 -0.32277000 -2.56708800

C 4.11418600 -1.44795300 -4.53050300

H 2.58275400 -0.12310200 -3.81584400

C 6.25119700 -1.30305100 -3.41851600

H 6.37171100 0.10387400 -1.79289500

C 5.43969000 -1.86971400 -4.40434300

H 3.47402500 -1.87696700 -5.29631500

H 7.28276800 -1.62685400 -3.30742100

H 5.83674800 -2.63227700 -5.06893400

C -0.94080300 1.44980300 3.34555800

C -0.07633900 1.74476000 4.41484000

C -2.11888500 0.73656300 3.62442200

C -0.37087400 1.32448600 5.71204200

H 0.81878400 2.33347300 4.23166400

C -2.41525500 0.31723000 4.92224400

H -2.80275800 0.50517300 2.81221600

C -1.53970900 0.60548500 5.97051900

H 0.30931700 1.56936700 6.52345600

H -3.32987700 -0.23752200 5.11347300

H -1.76854600 0.27651000 6.98022700

C 1.87154300 -0.03687600 -0.98533000

H 0.80939500 0.10095700 -1.20899800

C 1.64280500 0.84076800 1.83122000

H 1.39573700 0.47835500 2.83008500

P 1.90924800 -0.68153600 0.75673300

C 3.57492400 -1.28741500 1.18243500

C 4.20179800 -2.22477500 0.33921700

C 4.17426000 -0.89924800 2.38306700

C 5.41145400 -2.79755400 0.74189500

H 3.72601300 -2.53100500 -0.57975000

C 5.41154100 -1.45271200 2.75236600

H 3.71977800 -0.19288000 3.06664000

C 6.02847900 -2.40213800 1.94078600

H 6.97071200 -2.86663200 2.20386100

O 6.06889900 -3.75720600 0.04136300

O 5.91944400 -1.00191000 3.93554200

C 7.15686600 -1.53445700 4.38139400

H 7.96709200 -1.31891500 3.67242400

H 7.36872700 -1.04260900 5.33248300

H 7.09529400 -2.61925700 4.53887800

C 5.44269000 -4.29572000 -1.12506300

H 6.08131100 -5.12307500 -1.44046000

H 4.43370200 -4.66708300 -0.91230100

H 5.39057200 -3.54950000 -1.92533900

H 2.20265700 -0.85069500 -1.62854700

H 2.61546400 1.34101800 1.88511600

C -2.27564100 -3.19766100 2.47964800

H -1.51498500 -3.65599100 3.12396500

H -2.59387600 -2.27641200 2.98720400

C -3.47931700 -4.15364400 2.34976600

H -3.16798100 -5.08059800 1.85225000

H -4.22887700 -3.68070000 1.69590400

C -1.65151300 -2.85938900 1.11307800

H -1.26761700 -3.76436500 0.63200400

H -2.43203900 -2.45363300 0.45086300

C 0.71230400 -2.09122800 0.76331100

C 1.25159100 -3.22221100 0.03803900

C -0.53535800 -1.85915700 1.21555700

H -0.78497100 -0.90415300 1.67623800

H 1.97746600 -3.86473000 0.52245800

C 0.99766600 -3.39968400 -1.33877100

O 0.30899100 -2.70680100 -2.10178800

O 1.69481700 -4.50990000 -1.84301900

C 1.49174200 -4.75150100 -3.22743400

H 1.88674100 -3.93479400 -3.84472800

H 2.02869700 -5.67750300 -3.45365300

H 0.42922300 -4.86830600 -3.46593700

C -4.11486100 -4.46223700 3.67058300

H -4.48691500 -3.61850300 4.25296700

C -4.25317300 -5.68397300 4.20245200

H -3.91146900 -6.57887100 3.68958100

C -4.89435700 -5.86844600 5.52243500

O -5.32106100 -4.98551200 6.24378000

O -4.95604000 -7.18595000 5.84359600

C -5.55987400 -7.48717700 7.11971600

H -6.59932800 -7.14367800 7.11717600

H -5.03311100 -6.91584500 7.89313100

C -5.46960900 -8.97266500 7.35868100

C -6.60070800 -9.69379700 7.75450400

C -4.24789600 -9.64678200 7.22403500

C -6.51602900 -11.06254000 8.02092700

H -7.55527900 -9.18191500 7.85358600

C -4.16281100 -11.01431500 7.48018500

H -3.36744200 -9.09614600 6.90464200

C -5.29672500 -11.72597300 7.88292800

H -7.40389600 -11.60904200 8.32773500

H -3.21083100 -11.52643900 7.36718800

H -5.22884700 -12.79177500 8.08425300

**CP4**

C 3.49657000 -1.57229900 -0.72055400

C 4.57696900 -1.07240500 0.00515100

C 5.44575600 -1.96987200 0.70963400

C 5.20768600 -3.37883300 0.61067900

C 4.14803800 -3.84557800 -0.20567500

C 3.30131400 -2.98332900 -0.87061000

H 6.70104300 -0.45933900 1.63275100

C 6.51757700 -1.52364500 1.53373000

C 6.05138900 -4.27912800 1.31617200

H 4.00474400 -4.91739500 -0.31483300

C 7.08235400 -3.81440500 2.09970300

C 7.31238800 -2.42182700 2.21083100

H 5.86023100 -5.34579900 1.22831700

H 7.71784800 -4.51180900 2.63850400

H 8.12157300 -2.05903800 2.83886600

C 4.82684800 0.40937900 0.05202700

C 5.95391200 0.96045500 -0.64589100

C 3.94597000 1.24725600 0.73519500

C 6.84747700 0.16806200 -1.42115400

C 6.17799300 2.37384300 -0.59150600

C 4.19297900 2.65882400 0.81197600

C 7.90828100 0.74142100 -2.08687000

H 6.68309800 -0.90126500 -1.48802000

C 7.28723600 2.93347600 -1.28237900

C 5.28855500 3.18364300 0.15519600

C 8.13706600 2.13679400 -2.01417100

H 8.57584500 0.11732800 -2.67463500

H 7.44780200 4.00737700 -1.22415500

H 5.48575500 4.25072400 0.22141000

H 8.98050200 2.57453000 -2.54097700

C 3.32354500 3.58285900 1.60118800

C 3.19596200 3.44522100 2.99408000

C 2.65263100 4.64104300 0.96962800

C 2.41238400 4.33391100 3.73074100

H 3.72921900 2.64630500 3.50323600

C 1.86812600 5.53046900 1.70606600

H 2.73409600 4.75177800 -0.10781600

C 1.74390800 5.37911400 3.08836900

H 2.33152600 4.21435300 4.80786300

H 1.34914600 6.33819000 1.19704800

H 1.13509900 6.07259400 3.66223300

C 2.25649100 -3.54196800 -1.78662200

C 2.46080000 -3.49322500 -3.17645900

C 1.09431900 -4.14795100 -1.28777200

C 1.52524400 -4.04330300 -4.05344100

H 3.36565900 -3.03332700 -3.56760300

C 0.16161600 -4.69786100 -2.17189300

H 0.89110800 -4.16411700 -0.21968900

C 0.37184200 -4.65053400 -3.55084700

H 1.70098200 -4.00377800 -5.12555900

H -0.73644700 -5.15995500 -1.77067500

H -0.35792900 -5.08149700 -4.23166500

C 2.69348800 0.64623900 1.32927700

H 2.90322500 -0.28592400 1.86235500

C 2.49362200 -0.60213000 -1.30608300

H 1.81207400 -1.08959000 -2.00556700

P 1.46324200 0.17113100 0.03244700

C 0.70325900 1.68417300 -0.64638400

C -0.30577700 2.29086800 0.12745100

C 1.10021800 2.23705800 -1.86143000

C -0.89304100 3.47075200 -0.33245200

H -0.62844300 1.82807000 1.05065900

C 0.48476800 3.41611100 -2.32137800

H 1.86420300 1.79480900 -2.48805100

C -0.50353700 4.03463300 -1.56032800

H -0.99970800 4.94249100 -1.88025900

O -1.86399500 4.15485700 0.32804600

O 0.93130700 3.87136400 -3.52371400

C 0.33572400 5.04119900 -4.06635000

H 0.49555000 5.91199300 -3.41713500

H 0.82925500 5.20959900 -5.02485000

H -0.74093500 4.90438800 -4.22948300

C -2.29822000 3.66173100 1.59021600

H -3.06079100 4.36188500 1.93502500

H -2.73802600 2.66084000 1.49953700

H -1.47201300 3.63484500 2.31167800

H 2.18659400 1.32177400 2.01999100

H 3.00087900 0.21468900 -1.82812800

C -3.39859000 -1.46405900 -0.33535900

H -3.60113400 -0.39004100 -0.22197800

H -3.24454200 -1.63559900 -1.41069600

C -4.63315000 -2.26232800 0.13223800

H -4.81101800 -2.08215800 1.20008000

H -4.41037900 -3.33490900 0.02538600

C -2.12635500 -1.84678600 0.43364100

H -2.26743800 -1.68682200 1.51500900

H -1.92974600 -2.92631800 0.35124200

C 0.20440100 -0.92427700 0.76649300

C 0.57625500 -1.56363400 1.99291700

C -0.92452900 -1.06309300 0.00207700

H -0.97485000 -0.58774600 -0.97772900

H 1.12659400 -1.00940800 2.74447100

C 0.26813000 -2.90898400 2.32019000

O -0.22317300 -3.80045500 1.60844000

O 0.63967600 -3.20138600 3.62844900

C 0.45550400 -4.55679400 4.01160100

H 1.05106700 -5.23733700 3.39175200

H 0.78417500 -4.61990000 5.05262300

H -0.59432400 -4.86009000 3.93228700

C -5.86153700 -1.93786400 -0.66031700

H -5.82423900 -2.12795300 -1.73359900

C -6.99310800 -1.41528100 -0.16757400

H -7.11269800 -1.19158200 0.88910200

C -8.13979600 -1.11800600 -1.05135200

O -8.18992700 -1.30300400 -2.25326300

O -9.17051000 -0.59010500 -0.33905100

C -10.35155500 -0.27346600 -1.10322600

H -10.65011000 -1.16759800 -1.66337100

H -10.10941800 0.49961600 -1.84006600

C -11.43501600 0.18419200 -0.15988500

C -11.71815000 -0.53085600 1.01191300

C -12.20538800 1.31032900 -0.46814900

C -12.75122400 -0.12523600 1.85567700

H -11.11599700 -1.39877200 1.26493300

C -13.24699900 1.71304700 0.37088000

H -11.98893300 1.87755100 -1.37069200

C -13.52160100 0.99642300 1.53592000

H -12.95719900 -0.68576800 2.76385000

H -13.83646400 2.59017700 0.11708900

H -14.32791300 1.31038300 2.19348400

**TS3**

C 3.67548300 1.81697000 0.22683600

C 4.53773400 1.12426500 -0.62156800

C 5.16075900 1.79420700 -1.72521800

C 4.90117400 3.18934600 -1.91840000

C 4.06102000 3.86822400 -1.00369300

C 3.45477200 3.22236000 0.05426900

H 6.19113400 0.06864600 -2.54349300

C 5.99747900 1.12865900 -2.66579000

C 5.49429400 3.86315600 -3.02094700

H 3.88764100 4.93159400 -1.14391700

C 6.30323800 3.19072400 -3.90794500

C 6.55198900 1.80841600 -3.72760400

H 5.29017700 4.92319700 -3.15110200

H 6.74840100 3.71504400 -4.74919600

H 7.18367700 1.27887900 -4.43581300

C 4.78703400 -0.33860900 -0.38791100

C 6.06362500 -0.77042800 0.10543500

C 3.76337800 -1.26026900 -0.61133200

C 7.11965600 0.13285000 0.41652900

C 6.28409100 -2.16727600 0.32732700

C 4.00557600 -2.66318600 -0.41925200

C 8.32480700 -0.32458200 0.90213600

H 6.96472000 1.19589300 0.27084200

C 7.54167400 -2.60856700 0.82129900

C 5.23970800 -3.07855300 0.04132400

C 8.54382200 -1.70902800 1.10293700

H 9.11531200 0.38379200 1.13487800

H 7.69423200 -3.67408800 0.97659200

H 5.42570200 -4.14164400 0.17321100

H 9.50093000 -2.05586900 1.48283700

C 2.98704400 -3.71131000 -0.72994800

C 2.49567500 -3.88414500 -2.03533400

C 2.54916900 -4.59335600 0.27011900

C 1.59218300 -4.90607800 -2.32956600

H 2.84163400 -3.22506400 -2.82749900

C 1.64694900 -5.61736400 -0.02294200

H 2.91026800 -4.46224900 1.28620300

C 1.16538500 -5.77775200 -1.32401200

H 1.23220900 -5.02927600 -3.34768200

H 1.31694600 -6.28635900 0.76726900

H 0.46823700 -6.57897200 -1.55477000

C 2.60740300 4.01889100 0.99435000

C 3.00878300 4.21577400 2.32638100

C 1.42443000 4.62585400 0.54286300

C 2.24629000 5.00159700 3.19167400

H 3.93396000 3.76478800 2.67819900

C 0.66686100 5.41426000 1.41281700

H 1.09820500 4.44785200 -0.47852400

C 1.07207100 5.60497600 2.73543200

H 2.57466500 5.15016200 4.21727900

H -0.24677200 5.88081100 1.05221700

H 0.47904500 6.22072300 3.40692500

C 2.38955500 -0.75614500 -0.98408000

H 2.41700500 -0.02475000 -1.80258300

C 2.89186100 1.03444800 1.25708500

H 2.45037800 1.68408600 2.01430300

P 1.49916700 0.13541500 0.38449000

C 0.89113200 -1.13048300 1.55717500

C -0.08476900 -2.03635200 1.09094100

C 1.28231900 -1.14473000 2.89515900

C -0.63306800 -2.96397900 1.97585000

H -0.40168400 -2.00470400 0.05576900

C 0.71315000 -2.07969700 3.77930200

H 2.02070900 -0.45899700 3.29316000

C -0.23741200 -2.98994800 3.32566900

H -0.70016900 -3.72235000 3.97520000

O -1.57099700 -3.89190900 1.63906000

O 1.16394400 -2.01373500 5.06358000

C 0.62426600 -2.91937600 6.01389300

H 0.83221100 -3.96213600 5.74006300

H 1.11753700 -2.68898000 6.95983400

H -0.45954300 -2.78589800 6.12669700

C -2.05399600 -3.90922300 0.30215100

H -2.80047700 -4.70491500 0.26744200

H -2.52881700 -2.95610200 0.03584800

H -1.25029000 -4.13122600 -0.41060500

H 1.73131000 -1.56348100 -1.31033100

H 3.52203600 0.29103900 1.75715600

C -3.44602900 1.80573100 0.37717000

H -3.55674800 0.77019900 0.03110800

H -3.31792800 1.75574500 1.46778100

C -4.73077800 2.59459300 0.04935400

H -4.87763800 2.63336000 -1.03739500

H -4.59561800 3.63354600 0.38832800

C -2.19462900 2.43170900 -0.26423500

H -2.29574600 2.46128300 -1.35355100

H -2.10485100 3.47525400 0.07548800

C -0.08151600 1.19572800 -0.79540600

C 0.12729000 1.16957900 -2.16222200

C -0.92633100 1.70287400 0.10310500

H -0.72452100 1.60759200 1.16971500

H 0.01504600 0.24650600 -2.72483700

C 0.62333700 2.31651900 -2.88017600

O 0.84141400 3.44844500 -2.45149700

O 0.82965600 2.01119000 -4.20729400

C 1.30506900 3.09287200 -5.00540400

H 2.28543400 3.43778400 -4.65996800

H 1.38241800 2.69946300 -6.02162900

H 0.61228600 3.94029800 -4.98011600

C -5.94362600 2.02187700 0.71675800

H -5.93338000 1.97000900 1.80599300

C -7.03131400 1.55624400 0.08839900

H -7.12267200 1.56818300 -0.99436000

C -8.17063600 1.00447100 0.85388200

O -8.24143000 0.90648300 2.06478200

O -9.16181000 0.60575900 0.01585000

C -10.33643500 0.06520600 0.65705800

H -10.67960600 0.78514300 1.40924200

H -10.06662300 -0.85248100 1.18994400

C -11.38698800 -0.19504500 -0.39218800

C -11.71095400 0.78389600 -1.34157900

C -12.08414400 -1.40760300 -0.40501300

C -12.71201500 0.55163700 -2.28353100

H -11.16521600 1.72318200 -1.34601300

C -13.09427700 -1.63879500 -1.34181600

H -11.83488600 -2.17738300 0.32199500

C -13.40947800 -0.65983100 -2.28433200

H -12.95035100 1.31705500 -3.01745500

H -13.62720000 -2.58595100 -1.33858100

H -14.19126300 -0.83902600 -3.01763200

**(*S*)-CP2**

C -3.59956100 1.87713700 -0.07404600

H -3.74804500 0.82231500 -0.33601100

H -3.34556900 1.89918600 0.99484900

C -4.91164000 2.65453300 -0.30560400

H -5.18430900 2.61793900 -1.36797400

H -4.73763100 3.71335900 -0.05852800

C -2.42912100 2.45026400 -0.89290700

H -2.66481700 2.41530200 -1.96244600

H -2.29089500 3.50956500 -0.63188600

C -0.44686700 1.06895300 -1.53767400

C 0.23882200 0.42258300 -2.45487200

C -1.13003000 1.71706300 -0.63483000

H -0.74918600 1.73600200 0.38836800

H 0.05841900 -0.63103900 -2.65711100

C 1.29459300 1.09087700 -3.25917100

O 1.63536600 2.25269500 -3.18351000

O 1.84691800 0.20023100 -4.12142800

C 2.88494600 0.72925100 -4.95763900

H 3.71234600 1.10915300 -4.35175900

H 3.21555100 -0.10452600 -5.57817800

H 2.50190300 1.54308100 -5.57979500

C -6.03938500 2.13865300 0.53620100

H -5.90514000 2.16511500 1.61798200

C -7.18988600 1.63679700 0.06975800

H -7.40380900 1.57099000 -0.99367500

C -8.23406400 1.14700800 0.99853400

O -8.16521100 1.14392500 2.21330200

O -9.30797000 0.68702700 0.30981800

C -10.40052600 0.19409700 1.11844700

H -10.66850700 0.97194500 1.84235400

H -10.06022300 -0.67621700 1.68852400

C -11.55185100 -0.15333000 0.21089400

C -12.06799800 0.79658800 -0.68175000

C -12.14278000 -1.41952000 0.26850200

C -13.15195200 0.48311900 -1.49976900

H -11.60835500 1.77963400 -0.73951200

C -13.23537200 -1.73338700 -0.54367100

H -11.74571500 -2.16593900 0.95269200

C -13.74108400 -0.78295400 -1.43060000

H -13.54060200 1.22705500 -2.19024900

H -13.68467000 -2.72125200 -0.48667300

H -14.58824000 -1.02556400 -2.06657800

**TS4**

C 1.41311000 -1.37382900 5.28861400

H 0.99270400 -0.46242800 5.73706100

H 1.49578000 -2.11673000 6.09148100

C 2.77597800 -1.06747900 4.64443600

H 3.32852700 -2.00652200 4.51159200

H 3.38880100 -0.43027300 5.29578500

C 0.50439100 -1.84142200 4.14651500

H 0.85806300 -2.80596700 3.76595200

H -0.52934900 -1.99392900 4.48776700

C 0.35971900 -1.01015200 1.67746000

C 0.95146600 -2.12835000 1.09457000

C 0.56413600 -0.76174700 3.07470700

C 1.10729400 -2.54472600 -0.28076200

O 0.87041500 -1.93337000 -1.32261400

O 1.61846100 -3.81478600 -0.32527500

C 1.94895100 -4.29446600 -1.63151400

H 1.07603500 -4.27524200 -2.29028900

H 2.74237800 -3.68711900 -2.07826800

H 2.29789500 -5.31867100 -1.48701200

C 2.58164300 -0.41415900 3.27854700

H 2.44291200 0.66585200 3.28892400

C 3.30524900 -0.92664200 2.18657400

C 3.33046100 -0.24437400 0.93661500

O 2.71938300 0.80431100 0.66112000

O 4.11825100 -0.86748700 -0.00388200

C 4.08955400 -0.30734900 -1.31973800

H 3.09712200 -0.45536700 -1.75886600

C -2.43763300 -1.28107100 -0.58249600

C -2.34033300 -0.71556400 -1.85147900

C -2.25152100 -1.55169600 -3.01235400

C -2.31185700 -2.97177100 -2.84505300

C -2.48029800 -3.50492100 -1.54482900

C -2.54507700 -2.70247200 -0.42275800

H -2.00569700 0.03679500 -4.46868700

C -2.07430200 -1.03601300 -4.32678700

C -2.21913800 -3.81415200 -3.98618200

H -2.57567100 -4.58192700 -1.43133600

C -2.05495400 -3.28206500 -5.24439000

C -1.97646300 -1.87854100 -5.41205700

H -2.27600800 -4.89075000 -3.84346000

H -1.98035900 -3.93501800 -6.10974200

H -1.83522000 -1.46296700 -6.40612500

C -2.32777300 0.77921200 -2.00792000

C -3.48532400 1.42586300 -2.55891700

C -1.22291600 1.52621700 -1.59472300

C -4.67060400 0.72259800 -2.91833300

C -3.46770400 2.84657400 -2.72828100

C -1.19281700 2.94547900 -1.82282100

C -5.76472400 1.38820500 -3.42572600

H -4.70903500 -0.35238900 -2.78395500

C -4.60902700 3.50337600 -3.26377800

C -2.30086000 3.56229900 -2.37243900

C -5.73567000 2.79222800 -3.60645400

H -6.65937400 0.83071000 -3.68962700

H -4.57447100 4.58257400 -3.39318100

H -2.27082100 4.63246200 -2.56112600

H -6.60445700 3.30359000 -4.01190800

C 0.00591000 3.79389900 -1.55600700

C 1.24616700 3.50783100 -2.15084100

C -0.10754800 4.95657400 -0.77665200

C 2.33790900 4.35726500 -1.97090300

H 1.34918000 2.62568100 -2.77668700

C 0.98326500 5.80785200 -0.59676900

H -1.05606300 5.18206700 -0.29741000

C 2.21005900 5.51219700 -1.19547500

H 3.28650500 4.12039200 -2.44504500

H 0.87525900 6.69883500 0.01595100

H 3.05949000 6.17648100 -1.06032100

C -2.76896800 -3.35379000 0.90247000

C -3.91222300 -3.06307400 1.66616200

C -1.87195100 -4.32184900 1.38174900

C -4.14747400 -3.71349300 2.87806800

H -4.63257300 -2.33799000 1.29482400

C -2.10954300 -4.97427000 2.59264100

H -0.97385900 -4.54237900 0.81237700

C -3.24552400 -4.67152700 3.34589800

H -5.04091100 -3.47891100 3.45096500

H -1.40043600 -5.71623100 2.95020900

H -3.42809300 -5.17931400 4.28918400

C -0.09343500 0.82184600 -0.87402100

H 0.22142700 -0.09617600 -1.37729100

C -2.33811000 -0.36360500 0.61637300

H -2.60592600 -0.86388700 1.54789500

P -0.59919700 0.26977500 0.82101100

C -0.69995000 1.77379500 1.85560100

C -1.89533700 2.15840900 2.45966900

C 0.47274100 2.53739600 2.00615800

C -1.93106400 3.33687300 3.22763400

H -2.80943600 1.58385200 2.37526400

C 0.41911600 3.70033900 2.77730400

H 1.39659400 2.19658900 1.55226400

C -0.78256000 4.10760200 3.38518200

H -0.77027200 5.01772400 3.97193200

O -3.14194500 3.63394200 3.77902600

O 1.48295800 4.51248000 3.00570900

C 2.74315800 4.14251700 2.44624800

H 2.70191500 4.12097300 1.35222000

H 3.44452800 4.91175400 2.77462100

H 3.07146600 3.16297300 2.81228600

C -3.24871900 4.80231600 4.57764500

H -4.28585200 4.83933900 4.91558500

H -2.58328600 4.75635600 5.44963700

H -3.02244900 5.70776000 3.99931500

H 0.79386200 1.43671800 -0.72746100

H -2.98287900 0.51127800 0.49221700

C 5.14962100 -0.97895800 -2.16054500

C 6.39446900 -1.32693900 -1.61960900

C 4.91207900 -1.22428900 -3.51840600

C 7.37980100 -1.90218200 -2.42208400

H 6.57579300 -1.15819100 -0.56272500

C 5.90027200 -1.79283300 -4.32546700

H 3.94411000 -0.97175600 -3.94621600

C 7.13822800 -2.13391000 -3.77889900

H 8.33912300 -2.17176100 -1.98718600

H 5.69814500 -1.97746700 -5.37759900

H 7.90711600 -2.58235600 -4.40280500

H 4.26215100 0.77474800 -1.25615400

H 0.13032600 0.16186300 3.45209100

H 1.33736000 -2.86178300 1.79021400

H 3.82121400 -1.87845800 2.25322200

**CP5**

C 1.02241100 -4.66648900 3.17323000

H 0.94138100 -5.31237900 4.05550700

H 0.81499300 -5.29363000 2.29591100

C 2.40346400 -4.01528400 3.02596600

H 3.18396700 -4.72251800 2.72508100

H 2.70799500 -3.58125200 3.98841700

C 0.05110500 -3.47163700 3.22356400

H -0.94334000 -3.72170300 2.84161600

H -0.08358000 -3.14887500 4.26316300

C -0.02960900 -1.85793200 1.18985500

C -0.22029700 -2.88905200 0.28739000

C 0.70738900 -2.29875900 2.41624400

C -0.62817800 -2.89716700 -1.11938800

O -0.65760700 -1.97186600 -1.91782500

O -0.98000800 -4.16086800 -1.48892800

C -1.30281900 -4.32220800 -2.87526300

H -2.12829600 -3.66482000 -3.16188400

H -0.43479600 -4.09217600 -3.49997500

H -1.58529500 -5.36983000 -2.98980100

C 2.20017900 -2.89168200 1.98790800

H 2.88390700 -2.05240700 2.15370500

C 2.30830700 -3.34919000 0.58643100

C 2.54572900 -2.39691900 -0.41764500

O 2.53278800 -1.15682500 -0.25985500

O 2.76122000 -2.94585400 -1.68051100

C 2.92427300 -2.02649500 -2.75169600

H 2.57957400 -2.56526700 -3.64308700

C -2.93200800 -0.08110200 -0.01609000

C -2.79669200 0.73671200 -1.13636900

C -3.38826000 0.35897200 -2.38648100

C -4.15429600 -0.84847200 -2.45023300

C -4.32601200 -1.61650000 -1.27392900

C -3.74149300 -1.26501700 -0.07257900

H -2.62872200 2.02056100 -3.55552300

C -3.22288500 1.11397300 -3.58158600

C -4.74016800 -1.24062400 -3.68464000

H -4.95533900 -2.50195200 -1.31730900

C -4.56519900 -0.48379800 -4.82023000

C -3.79386200 0.70221000 -4.76547500

H -5.32878900 -2.15467400 -3.71363200

H -5.01348000 -0.79429100 -5.76009800

H -3.64915500 1.29121800 -5.66707000

C -2.02352300 2.02151700 -1.03517000

C -2.74057700 3.26471500 -1.07044000

C -0.63706900 2.00135400 -0.87139600

C -4.16060800 3.34135400 -1.14898900

C -2.00336300 4.48851200 -0.99138300

C 0.09853300 3.23681900 -0.83687900

C -4.80628200 4.55812300 -1.16418800

H -4.73821800 2.42496200 -1.19153100

C -2.69793100 5.72853500 -1.01982300

C -0.59324400 4.43204200 -0.89711100

C -4.07047200 5.76637800 -1.10507600

H -5.89096700 4.59157100 -1.22092700

H -2.12054700 6.64859400 -0.96740300

H -0.03612800 5.36545400 -0.89005300

H -4.59260200 6.71915200 -1.12250800

C 1.58888400 3.30360600 -0.78193500

C 2.38946400 2.66407800 -1.74306800

C 2.22174200 4.08383900 0.19976400

C 3.77845200 2.79262100 -1.71911900

H 1.91977600 2.07359700 -2.52485300

C 3.60995400 4.22017800 0.22137400

H 1.61727100 4.57090700 0.95988200

C 4.39306900 3.57472200 -0.73812300

H 4.37812100 2.27664000 -2.46355900

H 4.07959200 4.82274700 0.99428400

H 5.47482500 3.67779400 -0.72118000

C -4.02695300 -2.11242300 1.12364100

C -4.65935600 -1.57465600 2.25757100

C -3.73654000 -3.48625700 1.10946800

C -4.98248100 -2.38297200 3.34801800

H -4.91994800 -0.51916900 2.27297400

C -4.06243200 -4.29527200 2.19962200

H -3.23390900 -3.91496800 0.24729600

C -4.68429700 -3.74694800 3.32312100

H -5.47689000 -1.94805100 4.21272800

H -3.82486500 -5.35546300 2.17105300

H -4.93608400 -4.37731700 4.17163700

C 0.05831000 0.67004300 -0.69094800

H -0.25310300 -0.07033300 -1.43079400

C -2.13042000 0.26159600 1.21929400

H -2.44948000 -0.30544400 2.09450800

P -0.31992900 -0.11217000 0.95151700

C 0.60373000 0.83291600 2.21222100

C -0.05146500 1.51581700 3.23457000

C 2.00673300 0.85113600 2.10213400

C 0.70580000 2.24423100 4.17027900

H -1.12796500 1.50756200 3.35213800

C 2.74131000 1.57607700 3.04248900

H 2.48675500 0.28245300 1.31299300

C 2.09414000 2.27901500 4.07575200

H 2.70984000 2.82570500 4.77929600

O -0.01859400 2.88016100 5.13502300

O 4.09597000 1.66319000 3.04923400

C 4.82166000 0.93066100 2.06142700

H 4.57103900 1.26589800 1.04981200

H 5.87503900 1.13018600 2.26626900

H 4.62769300 -0.14515900 2.14182800

C 0.68180300 3.62242300 6.12087500

H -0.08155000 4.03442400 6.78334400

H 1.35990500 2.98252900 6.70064900

H 1.25687300 4.44405500 5.67386700

H 1.14452900 0.72703700 -0.72845400

H -2.20410600 1.32853700 1.44859500

C 4.35714600 -1.56765400 -2.95755100

C 4.62523000 -0.54027700 -3.87271300

C 5.42668100 -2.16693500 -2.28675000

C 5.93375900 -0.11804400 -4.11101700

H 3.80133200 -0.06981600 -4.40747000

C 6.73755400 -1.74266400 -2.51941200

H 5.22268600 -2.96423600 -1.57943900

C 6.99725700 -0.71843200 -3.43094100

H 6.12392200 0.67736100 -4.82785700

H 7.55846800 -2.21702000 -1.98693400

H 8.01763900 -0.39082800 -3.61280800

H 2.27287000 -1.16058400 -2.60467700

H 0.88037200 -1.46815300 3.10067300

H -0.20272500 -3.88855000 0.70157200

H 2.35585400 -4.40437600 0.34014500

**TS5**

C 1.02921000 -4.72617900 3.06421800

H 0.97118800 -5.38875000 3.93588600

H 0.78232700 -5.33406100 2.18346600

C 2.41415100 -4.09203400 2.88052800

H 3.17087700 -4.80292300 2.53096800

H 2.76292700 -3.68982200 3.84172700

C 0.07666800 -3.51975300 3.17202500

H -0.93094900 -3.74684300 2.81067400

H -0.02581000 -3.22208400 4.22269300

C -0.03541500 -1.86024800 1.17341800

C -0.19464400 -2.87978000 0.24259400

C 0.72588900 -2.33490500 2.37493200

C -0.60569800 -2.86413300 -1.16541800

O -0.63013200 -1.92668700 -1.94909100

O -0.96286200 -4.12012300 -1.55445800

C -1.28657700 -4.25829300 -2.94324000

H -2.10822500 -3.59172700 -3.21935800

H -0.41698100 -4.02373600 -3.56404000

H -1.57521700 -5.30234300 -3.07388000

C 2.18844600 -2.93448000 1.88537200

H 2.89734600 -2.11552100 2.04566400

C 2.23074000 -3.34977100 0.46334300

C 2.51867700 -2.37534900 -0.51414300

O 2.55732700 -1.14469800 -0.31931000

O 2.70974900 -2.89817900 -1.78906300

C 2.89302700 -1.95571800 -2.83867100

H 2.53600100 -2.46658500 -3.74128700

C -2.94518400 -0.06714500 0.01552100

C -2.81588900 0.77188600 -1.08979700

C -3.40992400 0.41604900 -2.34521100

C -4.17024500 -0.79371600 -2.43034300

C -4.33380900 -1.58583500 -1.26894800

C -3.74810900 -1.25442500 -0.06242100

H -2.66237800 2.10400200 -3.48373400

C -3.25226500 1.19525200 -3.52578600

C -4.75825000 -1.16441200 -3.67038400

H -4.95783700 -2.47412500 -1.32812300

C -4.59128800 -0.38419700 -4.79125500

C -3.82568400 0.80441300 -4.71557000

H -5.34235900 -2.08070900 -3.71552100

H -5.04141800 -0.67818700 -5.73554900

H -3.68725200 1.41202800 -5.60574200

C -2.04396500 2.05548500 -0.96720000

C -2.76082700 3.29909000 -0.97716900

C -0.65703500 2.03198200 -0.80809600

C -4.18107100 3.37724900 -1.04977600

C -2.02323000 4.52122300 -0.87747900

C 0.07876100 3.26659800 -0.75410700

C -4.82672300 4.59410400 -1.04034900

H -4.75880600 2.46174500 -1.10752900

C -2.71783300 5.76153900 -0.88049700

C -0.61270500 4.46293800 -0.78926100

C -4.09065100 5.80103200 -0.96086500

H -5.91158500 4.62863100 -1.09308800

H -2.14024600 6.68047600 -0.81262800

H -0.05536500 5.39600600 -0.76686500

H -4.61275300 6.75398700 -0.95879500

C 1.56952600 3.33092100 -0.70613800

C 2.36294600 2.71267900 -1.68699600

C 2.20982600 4.08686200 0.28950700

C 3.75228200 2.83805700 -1.66861700

H 1.88692900 2.14143800 -2.47919800

C 3.59849100 4.21996000 0.30578700

H 1.61107600 4.55720000 1.06455700

C 4.37449700 3.59569100 -0.67327500

H 4.34651300 2.33895100 -2.42884300

H 4.07410400 4.80317400 1.08980700

H 5.45654400 3.69632500 -0.66047400

C -4.02461000 -2.12751700 1.11731700

C -4.65899000 -1.61742900 2.26283800

C -3.72312000 -3.49833500 1.07538400

C -4.97297800 -2.44967300 3.33781200

H -4.92786700 -0.56458800 2.29964200

C -4.03973500 -4.33132700 2.15008500

H -3.21951600 -3.90588600 0.20354000

C -4.66348200 -3.81035900 3.28551700

H -5.46896500 -2.03596500 4.21198000

H -3.79355700 -5.38877000 2.10015100

H -4.90804800 -4.45936000 4.12202200

C 0.03827100 0.69804100 -0.65388000

H -0.27330500 -0.02711000 -1.40861200

C -2.14197400 0.25564700 1.25488800

H -2.45894200 -0.32596400 2.12116200

P -0.33296500 -0.12046700 0.97495200

C 0.59974400 0.80177200 2.24583400

C -0.04394700 1.47018100 3.28441200

C 2.00208000 0.81526300 2.12498000

C 0.72424800 2.17900800 4.22642000

H -1.11972300 1.46490600 3.40940600

C 2.74773000 1.52076300 3.07117200

H 2.47342700 0.25959100 1.32151300

C 2.11191600 2.20889300 4.12142400

H 2.73574000 2.74078100 4.82915100

O 0.01075800 2.80205300 5.20761600

O 4.10298100 1.60215200 3.06855400

C 4.81731700 0.88291200 2.06321600

H 4.55908200 1.23445400 1.05908500

H 5.87321800 1.07543400 2.26175600

H 4.62024300 -0.19344300 2.12854000

C 0.72274500 3.52485300 6.19953100

H -0.03310500 3.92946400 6.87504300

H 1.40267500 2.87254300 6.76310300

H 1.29796600 4.35103000 5.76114100

H 1.12440200 0.75774900 -0.69483800

H -2.21498800 1.31881700 1.50134100

C 4.33490200 -1.52249000 -3.03430700

C 4.62179200 -0.47233000 -3.91730800

C 5.39351100 -2.16579100 -2.38749800

C 5.93826500 -0.07113400 -4.14762100

H 3.80640600 0.03298700 -4.43288400

C 6.71240600 -1.76267000 -2.61209000

H 5.17553600 -2.98064200 -1.70474500

C 6.99088500 -0.71568600 -3.49163400

H 6.14293400 0.74291000 -4.83901900

H 7.52466600 -2.27125100 -2.09831300

H 8.01743200 -0.40432300 -3.66708100

H 2.25959700 -1.08065000 -2.66972100

H 0.92504200 -1.52227900 3.07381100

H -0.21827200 -3.88405100 0.64580300

H 2.26786900 -4.39790200 0.18631100

**CP6**

C 0.43866100 -3.13759900 4.53986300

H 0.84812500 -2.60786400 5.41134600

H -0.17611800 -3.96317000 4.91763700

C 1.58384000 -3.60771300 3.62655800

H 1.19475800 -4.34940200 2.91405200

H 2.40890600 -4.08557700 4.16760600

C -0.33059400 -2.16194800 3.63626300

H -0.94525900 -2.72812800 2.92494000

H -1.00492300 -1.49792300 4.19016200

C 0.53259200 -1.04704800 1.39940400

C 1.05926800 -2.25022600 0.64157100

C 0.77440700 -1.36836300 2.87448600

C 1.24392000 -2.18829000 -0.86196200

O 1.77716200 -1.29605700 -1.49487500

O 0.76070100 -3.30706400 -1.44959000

C 0.93181400 -3.38932100 -2.87426100

H 0.41191600 -2.56645400 -3.37151900

H 1.99350000 -3.35971900 -3.13276000

H 0.49493500 -4.34537200 -3.16464400

C 2.02603700 -2.32915200 2.87805300

H 2.85588000 -1.85315600 3.40787200

C 2.41600400 -2.55018100 1.40180100

C 3.53153600 -1.61768900 0.96282000

O 3.79272500 -0.53242800 1.44481300

O 4.23676400 -2.15973700 -0.05670900

C 5.23987000 -1.29942000 -0.65110100

H 4.79578900 -0.31037000 -0.79261100

C -2.62372900 -0.51732700 -0.65403700

C -2.47863800 0.27971800 -1.79208400

C -2.58993900 -0.28420000 -3.10562300

C -2.88403600 -1.67923200 -3.23532200

C -3.06526800 -2.45075800 -2.06286900

C -2.94418900 -1.91177500 -0.79589800

H -2.13966300 1.52373900 -4.21785700

C -2.38454800 0.47047500 -4.29610500

C -2.98908800 -2.25606000 -4.53060400

H -3.32441000 -3.50141800 -2.16757800

C -2.79531300 -1.49476600 -5.66045200

C -2.48438200 -0.11870700 -5.53724600

H -3.22397400 -3.31512300 -4.61004800

H -2.87576500 -1.94557600 -6.64592300

H -2.32091500 0.47746700 -6.43101800

C -2.16372100 1.73785300 -1.63673500

C -3.16754400 2.71443200 -1.94880100

C -0.91154500 2.12696200 -1.15940100

C -4.48654500 2.36763500 -2.35828700

C -2.84783000 4.10226700 -1.80974900

C -0.58300700 3.52415300 -1.07580500

C -5.42432900 3.33954600 -2.63021600

H -4.75288300 1.32047500 -2.44888700

C -3.83449900 5.08150300 -2.10598900

C -1.54455000 4.46414400 -1.39376200

C -5.09706800 4.71169100 -2.50911100

H -6.42580100 3.05093500 -2.93809100

H -3.57161300 6.13180100 -2.00317900

H -1.28913400 5.51981300 -1.34547800

H -5.84548900 5.46795900 -2.72991100

C 0.77875000 4.02173600 -0.71761900

C 1.90593000 3.65766300 -1.47393200

C 0.94838500 4.93653900 0.33328100

C 3.16296500 4.18883700 -1.18483600

H 1.79122900 2.96835100 -2.30619300

C 2.20539900 5.46894000 0.62364000

H 0.08913200 5.21523500 0.93658000

C 3.31756700 5.09765700 -0.13490600

H 4.02024100 3.89972000 -1.78712800

H 2.31586600 6.16803100 1.44820100

H 4.29615300 5.51476600 0.08771700

C -3.18147200 -2.81757000 0.36700100

C -4.18882600 -2.55463100 1.31073900

C -2.44418600 -4.00681500 0.49122500

C -4.44489600 -3.44865100 2.35042000

H -4.78951500 -1.65403400 1.21375900

C -2.70231400 -4.90261600 1.53079800

H -1.65433400 -4.21491400 -0.22524700

C -3.70185900 -4.62596500 2.46529000

H -5.23288000 -3.22954700 3.06626000

H -2.11748300 -5.81528400 1.61184700

H -3.90219300 -5.32197000 3.27537300

C 0.05607600 1.06555300 -0.69332200

H 0.15638300 0.26567500 -1.42740300

C -2.32251700 0.09527600 0.68658300

H -2.67646500 -0.51406600 1.51837100

P -0.42805100 0.25910800 0.93095400

C -0.23133500 1.56287400 2.20978300

C -1.29936400 2.16279600 2.86952000

C 1.09441200 1.94222400 2.49530500

C -1.04831600 3.15750000 3.83364900

H -2.33064000 1.88855800 2.68542400

C 1.32936600 2.92639100 3.45482800

H 1.90960700 1.43524600 1.99301700

C 0.25703900 3.54219700 4.12677000

H 0.48826700 4.29933900 4.86592600

O -2.15849800 3.68144400 4.43098500

O 2.56570800 3.35959500 3.82093300

C 3.69641500 2.78183800 3.17059800

H 3.69381300 3.01020100 2.09837100

H 4.56905100 3.23989200 3.64040700

H 3.73186100 1.69480400 3.30528800

C -1.97847200 4.67919300 5.42205300

H -2.98023900 4.94849300 5.76212100

H -1.39480700 4.30268000 6.27259300

H -1.48247800 5.56969900 5.01312100

H 1.05857800 1.45588100 -0.52101000

H -2.76864800 1.09013800 0.76462300

C 5.68013100 -1.90873500 -1.95562100

C 6.94340700 -2.49451800 -2.08501800

C 4.82263300 -1.88544100 -3.06590800

C 7.35108400 -3.04765600 -3.30159100

H 7.61367200 -2.51730900 -1.22859500

C 5.22693200 -2.44003300 -4.27986200

H 3.83690500 -1.43920500 -2.96428000

C 6.49285400 -3.02162200 -4.40120200

H 8.33675400 -3.49730900 -3.38849000

H 4.55742700 -2.41304700 -5.13602600

H 6.80810500 -3.44931400 -5.34942400

H 6.07519800 -1.19706900 0.04943900

H 1.00397400 -0.46638100 3.44909800

H 0.44281700 -3.14474300 0.82375600

H 2.73775500 -3.57719100 1.19744000

**TS6**

C 3.85058200 0.19200000 4.02851600

H 4.29971800 0.18497700 5.02863000

H 4.37082400 -0.56835000 3.43202300

C 3.94899200 1.54852600 3.32192600

H 4.94778400 1.79484500 2.95943100

H 3.63190900 2.34358200 4.01290900

C 2.33685200 -0.08101300 4.06311600

H 2.08006500 -1.13906800 4.17624400

H 1.89546200 0.44428200 4.91924400

C 1.21068500 -0.47988200 1.76153700

C 2.00614700 -1.56938700 1.51369600

C 1.74676400 0.53850500 2.74970500

C 1.83156100 -2.71293400 0.61897600

O 0.84619600 -3.01238300 -0.04617100

O 2.94603800 -3.48623400 0.63580100

C 2.91140200 -4.63654200 -0.21759500

H 2.08065700 -5.29526700 0.05135900

H 2.79932800 -4.33721300 -1.26357900

H 3.86686700 -5.14008300 -0.06710000

C 2.94154200 1.44226600 2.14712800

H 2.47732100 2.42808500 1.98554900

C 3.52010400 0.90568200 0.87428400

C 4.71963800 1.39694900 0.31419800

O 5.49618600 2.25941500 0.74718300

O 4.97428000 0.77183600 -0.92815800

C 6.23319600 1.02930600 -1.52475200

H 6.06662900 1.51286500 -2.49879100

C -2.06909800 -2.19021700 1.26063700

C -2.52355100 -2.35867000 -0.04492900

C -2.63254900 -3.67239900 -0.60884900

C -2.30972200 -4.80185500 0.20875700

C -1.90730600 -4.58963100 1.54875000

C -1.77950300 -3.32492200 2.08679800

H -3.24784900 -3.06354800 -2.59918500

C -3.01939700 -3.90777700 -1.95836300

C -2.40897000 -6.11061200 -0.33610900

H -1.70314900 -5.45483800 2.17456300

C -2.79358900 -6.30320800 -1.64283500

C -3.09526200 -5.18791100 -2.46096200

H -2.16780300 -6.95834600 0.30079500

H -2.86170700 -7.30760700 -2.05161000

H -3.38720800 -5.34399300 -3.49588500

C -2.89182900 -1.16293300 -0.87860100

C -4.27545900 -0.92149400 -1.17593500

C -1.90542100 -0.29149400 -1.34346400

C -5.33074400 -1.72580300 -0.65894500

C -4.62019900 0.19499400 -2.00232700

C -2.25436900 0.79339200 -2.21932200

C -6.64645700 -1.44491600 -0.95471400

H -5.08950200 -2.56683100 -0.01887700

C -5.98667300 0.45237300 -2.29760500

C -3.58551400 1.00809800 -2.52147700

C -6.98116100 -0.34878300 -1.78615800

H -7.43570800 -2.06978300 -0.54575400

H -6.23028500 1.29980300 -2.93387500

H -3.84997900 1.81692100 -3.19777100

H -8.02304100 -0.14195900 -2.01462300

C -1.24034300 1.67391700 -2.87062600

C -0.21984000 1.13747200 -3.67466500

C -1.33381800 3.06986200 -2.75349900

C 0.68526800 1.97163800 -4.33132900

H -0.14911100 0.06051400 -3.80340800

C -0.43115800 3.90528200 -3.41258900

H -2.10843000 3.49803400 -2.12367200

C 0.58275300 3.35883100 -4.20249500

H 1.46554400 1.53711500 -4.95003200

H -0.51443600 4.98270300 -3.29984200

H 1.28648100 4.00815800 -4.71603900

C -1.36736300 -3.19937600 3.51678700

C -2.21672100 -2.61427800 4.47131400

C -0.13842600 -3.72305400 3.94799200

C -1.84425100 -2.54791900 5.81451900

H -3.18555300 -2.23059900 4.16055200

C 0.23428700 -3.65772400 5.29175900

H 0.53037300 -4.17217700 3.21951600

C -0.61637100 -3.06835800 6.22894700

H -2.51822900 -2.09789500 6.53867900

H 1.19190600 -4.06501400 5.60478300

H -0.32578500 -3.01647100 7.27457100

C -0.48004800 -0.47970000 -0.86284400

H -0.14778500 -1.51731000 -0.93458100

C -1.81767400 -0.78334700 1.75398600

H -1.64351200 -0.74042500 2.83030700

P -0.32766800 -0.02569300 0.92970700

C -0.52591800 1.79138600 1.01930300

C -1.60935000 2.37104400 1.67752800

C 0.44278800 2.58606300 0.37900300

C -1.73567100 3.77239800 1.69634900

H -2.36628300 1.79467500 2.19383200

C 0.31229200 3.97727900 0.41902700

H 1.30757900 2.12400200 -0.08434700

C -0.78483700 4.57232100 1.06943000

H -0.84144200 5.65375000 1.07544200

O -2.82522300 4.24559100 2.36248200

O 1.18886400 4.84531100 -0.13665300

C 2.39482700 4.33431300 -0.71810800

H 2.17495900 3.68521000 -1.57287100

H 2.94192500 5.21361200 -1.06130800

H 3.00019800 3.79187700 0.01515900

C -3.00609700 5.65201300 2.44416800

H -3.92369000 5.80180300 3.01553400

H -2.16935100 6.13486300 2.96504900

H -3.11971500 6.10101900 1.44890400

H 0.22990100 0.15209800 -1.39845500

H -2.66407600 -0.13407500 1.51456800

C 7.02590400 -0.24917500 -1.71936100

C 6.70934200 -1.41810800 -1.01826000

C 8.11684500 -0.26536700 -2.59894000

C 7.47478400 -2.57421700 -1.18771700

H 5.85494900 -1.41361200 -0.34902000

C 8.88309900 -1.41871900 -2.76791500

H 8.36882700 0.63516100 -3.15678600

C 8.56427700 -2.58065600 -2.06045900

H 7.21733300 -3.47464000 -0.63439800

H 9.72643200 -1.41191400 -3.45446800

H 9.15828900 -3.48175800 -2.19140800

H 6.78243900 1.73687400 -0.89171900

H 0.94384300 1.22362300 3.02999800

H 2.92564000 -1.62005900 2.08440800

H 2.96246900 0.22587000 0.25099000

**CP7**

C 2.97793900 2.13472100 3.20076400

H 3.28822500 2.36248500 4.22742800

H 3.41711600 1.16637700 2.93523000

C 3.42422400 3.20021500 2.19016600

H 4.49836800 3.18044700 1.97553000

H 3.20071300 4.20062700 2.58603300

C 1.44942400 2.10618700 3.02573400

H 1.01136200 1.15220900 3.33410300

H 0.99524700 2.88914200 3.64900800

C 0.81141800 1.25013200 0.59849800

C 2.12787500 0.61087800 0.15959800

C 1.16907700 2.42038200 1.51111000

C 2.08342900 -0.21677800 -1.11636100

O 1.97103500 0.21680500 -2.24542800

O 2.19582100 -1.54136000 -0.85013500

C 2.21747200 -2.40610300 -1.99735900

H 1.29368300 -2.31002800 -2.57454800

H 3.06507100 -2.15949400 -2.64264300

H 2.31005500 -3.41691000 -1.60050600

C 2.53798700 2.95648800 0.95014300

H 2.40396700 3.88816500 0.39237000

C 3.02328500 1.85774500 -0.06254300

C 4.50210500 1.54672100 0.03373100

O 4.99352400 0.62399200 0.65679600

O 5.23243500 2.45895400 -0.65041500

C 6.67851500 2.31531200 -0.60133000

H 7.04939600 3.34002900 -0.67841800

C -0.89552600 -2.12868100 1.41225200

C -1.49884900 -2.75705300 0.32059000

C -0.98833900 -3.99600700 -0.19103400

C 0.12984800 -4.60413700 0.46333000

C 0.68664200 -3.96640300 1.59704800

C 0.21045600 -2.76173600 2.07656200

H -2.34872100 -4.18133200 -1.87290100

C -1.51558200 -4.63666000 -1.34907000

C 0.65538100 -5.82713300 -0.03640500

H 1.51338300 -4.44996600 2.11138800

C 0.11466500 -6.42465800 -1.15227700

C -0.97842300 -5.81653500 -1.81574300

H 1.49829400 -6.27994300 0.48075200

H 0.52545600 -7.35774400 -1.52829600

H -1.39627200 -6.28446500 -2.70315400

C -2.67188600 -2.11022200 -0.35288600

C -3.97459500 -2.69753800 -0.21873200

C -2.49218300 -0.93273200 -1.08020500

C -4.22733800 -3.84647900 0.58329100

C -5.08099900 -2.08631400 -0.88889200

C -3.59988200 -0.35608800 -1.79276700

C -5.49794100 -4.36637600 0.69838600

H -3.40365800 -4.31042400 1.11418000

C -6.37845700 -2.65177000 -0.75827700

C -4.84901000 -0.93615800 -1.67993600

C -6.58614400 -3.76886000 0.01775600

H -5.66762900 -5.24183600 1.31943400

H -7.20656300 -2.17929500 -1.28153000

H -5.68225500 -0.50941400 -2.23271100

H -7.58258500 -4.19136200 0.11455000

C -3.46454900 0.82098300 -2.70170400

C -2.58347200 0.80195100 -3.79649800

C -4.28415800 1.94743000 -2.52613500

C -2.52017100 1.87749500 -4.68278800

H -1.95758500 -0.07022100 -3.96494500

C -4.22518300 3.02238900 -3.41395300

H -4.95838700 1.98311600 -1.67528400

C -3.34210400 2.99181500 -4.49572400

H -1.83493900 1.83914600 -5.52535800

H -4.86370100 3.88714400 -3.25448900

H -3.29763400 3.82662800 -5.19026000

C 0.86972200 -2.19250200 3.28869800

C 0.15379300 -1.96714900 4.47686100

C 2.25274300 -1.94880800 3.28418300

C 0.80097000 -1.50495700 5.62314100

H -0.91209900 -2.17826900 4.50671000

C 2.90046700 -1.48852700 4.43220900

H 2.81547200 -2.10882400 2.36862700

C 2.17680700 -1.26335100 5.60449300

H 0.23110900 -1.34297100 6.53446600

H 3.97026200 -1.30019300 4.40536300

H 2.68031200 -0.90323700 6.49760700

C -1.14099300 -0.25878300 -1.05410500

H -0.34533500 -0.99159200 -1.20543900

C -1.37508600 -0.75647300 1.80017200

H -1.01979900 -0.45052500 2.78474000

P -0.74408100 0.58559200 0.57408400

C -1.93890600 1.96739400 0.79078200

C -2.93429600 1.96778200 1.76354700

C -1.80439200 3.05838300 -0.09026900

C -3.81067000 3.06445200 1.86603400

H -3.06271900 1.15819700 2.47042500

C -2.67762400 4.13990300 0.02219300

H -1.01090400 3.04558900 -0.82678200

C -3.68889300 4.14669300 0.99957000

H -4.34114500 5.00968400 1.05005400

O -4.74456100 2.97061300 2.85558700

O -2.63940700 5.24824900 -0.76929400

C -1.60053100 5.34846500 -1.73594800

H -1.67494700 4.55457000 -2.48824800

H -1.73717600 6.31900000 -2.21690700

H -0.61080800 5.31316700 -1.26304000

C -5.65282200 4.04663500 3.02641700

H -6.29635600 3.76561900 3.86206300

H -5.12965600 4.98119300 3.26846800

H -6.26835100 4.20116400 2.13015900

H -1.03020200 0.50795000 -1.81905800

H -2.46779000 -0.72210900 1.79859400

C 7.20519600 1.46583600 -1.73246300

C 7.58906600 2.05853500 -2.94188300

C 7.31561700 0.07505000 -1.59197100

C 8.07471900 1.28063500 -3.99378700

H 7.50824900 3.13705700 -3.05874100

C 7.80190900 -0.70393000 -2.64238500

H 7.00696600 -0.38762000 -0.65952100

C 8.18265900 -0.10350900 -3.84496600

H 8.37096200 1.75421200 -4.92609900

H 7.88604500 -1.78085100 -2.52121200

H 8.56365700 -0.71129900 -4.66158300

H 6.95253900 1.89717700 0.36943300

H 0.40332800 3.20145500 1.45972100

H 2.58246000 -0.02031700 0.93399000

H 2.82922600 2.22281200 -1.07403600

**TS7**

C -2.99451700 -1.06584500 0.39799200

C -3.66426400 -0.13792800 -0.39640400

C -4.65252600 -0.56715000 -1.34357700

C -4.96977500 -1.96096200 -1.42238900

C -4.32747900 -2.86294000 -0.53972400

C -3.36580100 -2.45253300 0.36400000

H -5.07083800 1.38198100 -2.20032200

C -5.31045300 0.32502400 -2.23673200

C -5.93595800 -2.40487800 -2.36587500

H -4.62208100 -3.90914200 -0.56051400

C -6.55690000 -1.51483400 -3.21160200

C -6.23530700 -0.13748400 -3.14691800

H -6.17153400 -3.46556000 -2.40874600

H -7.29088700 -1.86520200 -3.93204200

H -6.72213200 0.56035000 -3.82272800

C -3.34212300 1.32340100 -0.26898400

C -4.29935200 2.19906300 0.34395500

C -2.11507400 1.81198000 -0.71656100

C -5.52906600 1.74021900 0.89616800

C -3.99463100 3.59392700 0.43878100

C -1.84530000 3.22152000 -0.67877600

C -6.40729300 2.61594000 1.49540500

H -5.76892500 0.68395200 0.84931200

C -4.92651000 4.47373900 1.05396500

C -2.77622700 4.06584500 -0.10336400

C -6.10863300 3.99795700 1.57223700

H -7.33765600 2.24305200 1.91500100

H -4.68285700 5.53194000 1.11097900

H -2.57956800 5.13477100 -0.08671000

H -6.81257600 4.67767100 2.04445800

C -0.63681200 3.84323100 -1.29607800

C -0.36550200 3.68998900 -2.66637200

C 0.19914800 4.67787500 -0.53834300

C 0.71115300 4.35000800 -3.25962900

H -1.02009900 3.07176600 -3.27558300

C 1.27501200 5.33971400 -1.13088300

H 0.01260000 4.78927600 0.52570100

C 1.53396300 5.17940300 -2.49384600

H 0.89972900 4.22504900 -4.32256300

H 1.91715000 5.97234200 -0.52446000

H 2.37033500 5.69648600 -2.95625300

C -2.80491000 -3.46242300 1.30992000

C -2.94280200 -3.30101500 2.69875800

C -2.19293100 -4.63110900 0.83101300

C -2.47294500 -4.27359300 3.58148200

H -3.43919800 -2.41569800 3.08775600

C -1.72091200 -5.60354600 1.71336500

H -2.07253300 -4.76526000 -0.23979600

C -1.85793800 -5.42791300 3.09149800

H -2.59563700 -4.13231100 4.65207300

H -1.23917900 -6.49579800 1.32284700

H -1.49061700 -6.18551300 3.77842500

C -1.08163200 0.81597100 -1.20374900

H -1.54410600 0.11106700 -1.89695200

C -1.83500900 -0.58747600 1.24196100

H -1.49813000 -1.31460700 1.97689100

P -0.35256600 -0.19591800 0.18911800

C 0.72188900 0.99063600 1.08392700

C 0.37820800 1.41799300 2.36488800

C 1.84535100 1.50743300 0.41760000

C 1.17002200 2.38745700 3.00012800

H -0.45247300 1.00264900 2.91894800

C 2.62525300 2.47098300 1.06704400

H 2.14628800 1.11253000 -0.54412200

C 2.28455800 2.91830100 2.35613200

H 2.91950100 3.66600500 2.81525300

O 0.76070800 2.74427000 4.25308700

O 3.73696000 3.04696400 0.54242400

C 4.10557800 2.72607200 -0.80536300

H 5.02192500 3.28884800 -0.99331600

H 4.28805300 1.65604400 -0.92826600

H 3.32547800 3.05337400 -1.50315600

C 1.53331700 3.69681200 4.96396600

H 1.04105200 3.82099400 5.93041800

H 2.56160000 3.34582400 5.12230600

H 1.56079200 4.66420500 4.44443400

H -0.24916800 1.30023000 -1.71715300

H -2.09199600 0.33789300 1.76450800

C 0.12633900 -3.37731700 -3.96028400

H -0.20397500 -4.30798800 -4.43757400

H -0.23494100 -2.54976700 -4.58772300

C 1.65338700 -3.31279500 -3.81600200

C -0.42593500 -3.21472300 -2.54302800

H -1.52447300 -3.15624100 -2.54105400

H -0.14970800 -4.08439300 -1.93409500

C 0.30430200 -1.73921300 -0.55152700

C 0.91635500 -2.69472600 0.25061600

C 0.18725500 -1.94272200 -1.96612600

H -0.13650600 -1.07980300 -2.54366800

H 1.23701900 -3.62123000 -0.20385300

C 1.08658200 -2.58240500 1.67405700

O 0.68158600 -1.64662400 2.37303200

O 1.75054400 -3.63822800 2.20914900

C 1.93179600 -3.59237900 3.62609900

H 0.96578600 -3.60957600 4.14073500

H 2.51084900 -4.48282600 3.87681100

H 2.47307600 -2.68959900 3.92369600

C 2.05651300 -2.27303600 -2.77189400

H 2.14634200 -1.24977000 -3.13280800

C 3.01997300 -2.63790900 -1.81747800

H 3.25546100 -3.67853600 -1.61972500

C 3.72478200 -1.63709800 -1.08758700

O 3.55415100 -0.41569900 -1.18347200

O 4.67101400 -2.16385200 -0.22872600

C 5.37531900 -1.22659000 0.58440400

H 5.69036800 -1.80875900 1.45880300

H 4.69687100 -0.43673400 0.92014400

C 6.58836000 -0.62091500 -0.09485600

C 7.03707700 0.65146400 0.27918600

C 7.31141500 -1.33803200 -1.05495400

C 8.19158400 1.19487600 -0.28782100

H 6.47140300 1.22598900 1.01010300

C 8.46096700 -0.79323100 -1.62909000

H 6.95861100 -2.31975700 -1.35643200

C 8.90794600 0.47316100 -1.24485500

H 8.52574900 2.18539700 0.01141100

H 9.00945000 -1.35937700 -2.37800600

H 9.80407800 0.89627600 -1.69162700

H 2.02376100 -4.29554100 -3.49650600

H 2.13848100 -3.09205000 -4.77582600

**CP8**

C -3.02991900 -1.11787100 0.27783500

C -3.52501100 -0.08049700 -0.51128500

C -4.55335300 -0.32950200 -1.48001300

C -5.10807900 -1.64602700 -1.57292100

C -4.65417100 -2.64602600 -0.67953600

C -3.64508100 -2.41576100 0.23742200

H -4.60967100 1.66578600 -2.32947700

C -5.02984900 0.66725800 -2.37781500

C -6.11945000 -1.91137300 -2.53611600

H -5.13725800 -3.61959000 -0.70167500

C -6.55879500 -0.92397300 -3.38748500

C -6.00294500 0.37594200 -3.30819100

H -6.53595000 -2.91439000 -2.58993000

H -7.32855800 -1.13856100 -4.12364000

H -6.34680100 1.14992100 -3.98888700

C -3.00387500 1.31833100 -0.32967800

C -3.85517800 2.29003000 0.29763900

C -1.71424000 1.66048800 -0.73632200

C -5.14741200 1.97872500 0.80897000

C -3.37521000 3.62948400 0.44921400

C -1.26416100 3.02076500 -0.63701300

C -5.91929200 2.94168200 1.42086400

H -5.52253200 0.96557000 0.71997900

C -4.19990400 4.60301200 1.07607100

C -2.09324400 3.95765200 -0.04963900

C -5.44644200 4.26973200 1.55263400

H -6.90042200 2.67961800 1.80745800

H -3.82179200 5.61768400 1.17533800

H -1.76120900 4.99080000 0.01150700

H -6.06800200 5.01989800 2.03377000

C 0.03186200 3.50227400 -1.19855000

C 0.33993400 3.33476800 -2.55883700

C 0.92688700 4.22610600 -0.39476500

C 1.50839800 3.87275800 -3.09813200

H -0.35282800 2.80056300 -3.20371500

C 2.09417300 4.76730300 -0.93380900

H 0.71168500 4.34626200 0.66308700

C 2.38831000 4.59366600 -2.28801900

H 1.72659500 3.73459100 -4.15352400

H 2.77893900 5.31493500 -0.29213400

H 3.29735800 5.01495100 -2.70870200

C -3.30129600 -3.50716800 1.19657000

C -3.35265600 -3.29163000 2.58476200

C -3.00538200 -4.80038300 0.73641700

C -3.10025300 -4.32993600 3.48094700

H -3.61541400 -2.30764100 2.96387700

C -2.75277100 -5.83979600 1.63243100

H -2.96171900 -4.98608100 -0.33219200

C -2.79606100 -5.60812600 3.00808000

H -3.14942300 -4.14055300 4.54987300

H -2.51774300 -6.83079900 1.25312100

H -2.59777400 -6.41688600 3.70614200

C -0.80397300 0.56063800 -1.24130000

H -1.33117900 -0.04606600 -1.98036800

C -1.81750100 -0.84441300 1.13840800

H -1.58252300 -1.65682600 1.82483000

P -0.27587300 -0.58633800 0.12162100

C 0.87200800 0.38169500 1.19487900

C 0.49896500 0.68309200 2.50668900

C 2.06684200 0.88151400 0.65208900

C 1.32354900 1.50801800 3.28647500

H -0.38168500 0.27579000 2.98212400

C 2.87985000 1.69449500 1.44852900

H 2.35171800 0.59722100 -0.35641300

C 2.50917300 2.01923600 2.76470800

H 3.17236500 2.65449100 3.33912300

O 0.87333800 1.74544400 4.55511000

O 4.05686300 2.22965000 1.03420900

C 4.51895000 1.91059100 -0.28329900

H 5.49108600 2.40010100 -0.37139400

H 4.62733500 0.83140900 -0.42181600

H 3.83655400 2.30678300 -1.04308400

C 1.66711900 2.55460600 5.40561000

H 1.13128900 2.60726800 6.35542300

H 2.65930800 2.11445100 5.57278000

H 1.78927400 3.56886700 5.00197000

H 0.11626900 0.92061300 -1.70256500

H -1.96441000 0.06831000 1.72101400

C -0.67205700 -4.25058300 -3.67447600

H -1.09149700 -5.24795900 -3.85200400

H -1.03960200 -3.59455000 -4.47603100

C 0.86106000 -4.23008700 -3.63707700

C -1.05766700 -3.67826400 -2.30495500

H -2.10727300 -3.37019100 -2.24274700

H -0.89513100 -4.43993500 -1.53278600

C 0.18834400 -2.18815300 -0.62533000

C 0.88761900 -3.13394700 0.06829100

C -0.08982800 -2.48530300 -2.07233000

H -0.51131500 -1.61592000 -2.57210500

H 1.16881900 -4.04415600 -0.44619500

C 1.23783200 -3.08030700 1.48513700

O 0.74638700 -2.32213000 2.31245700

O 2.17015900 -4.00758600 1.80662300

C 2.57949800 -4.02198500 3.18110900

H 1.71794400 -4.15951700 3.84012900

H 3.27171800 -4.85984200 3.27067400

H 3.08263100 -3.08604200 3.43969500

C 1.23919100 -2.89093000 -2.97367400

H 1.23091200 -2.09827500 -3.73323400

C 2.54343300 -2.87853400 -2.27633100

H 3.14863900 -3.77464900 -2.22367300

C 2.92279200 -1.65442200 -1.74723600

O 2.20296600 -0.62050700 -1.83554700

O 4.10418400 -1.48536300 -1.02181900

C 4.85235300 -2.64325800 -0.66941200

H 5.34893800 -3.06923400 -1.55361100

H 4.16693400 -3.40760900 -0.27530500

C 5.87531600 -2.28314400 0.38341200

C 7.17437900 -2.79830900 0.31946700

C 5.52294200 -1.47208500 1.47178100

C 8.10429500 -2.51989200 1.32425700

H 7.46252100 -3.42025400 -0.52549100

C 6.45252800 -1.18512500 2.47133500

H 4.52074700 -1.05651400 1.52012500

C 7.74581800 -1.71122600 2.40345800

H 9.11006600 -2.92719700 1.25752700

H 6.16726000 -0.54662000 3.30368500

H 8.46944900 -1.48704700 3.18305200

H 1.22358700 -5.06752300 -3.02337200

H 1.31734100 -4.33707500 -4.62793400

**TS8**

C 5.01123100 -0.53926900 4.93272800

H 5.16212000 -0.91301000 3.91367200

H 4.89332200 -1.41789600 5.57823300

C 6.19012300 0.31471700 5.42818600

H 7.04822300 -0.28471900 5.74766900

H 6.53424200 0.97532100 4.61958900

C 3.76920100 0.38150000 5.04761100

H 2.93385900 -0.13736300 5.52718300

H 3.40998500 0.69956300 4.06167300

C 3.21334000 2.11173000 6.89279900

C 2.87626400 1.20353700 7.87897200

C 4.19632600 1.63367700 5.88036500

C 2.11845700 1.54122000 9.07712600

O 1.64472900 2.64937300 9.32048100

O 1.96759200 0.48737900 9.90518700

C 1.22916400 0.74748700 11.10460000

H 0.19610700 1.01893800 10.86737300

H 1.68881900 1.56003100 11.67368100

H 1.25868200 -0.18278000 11.67325600

C 5.61224600 1.16909700 6.57970100

H 6.19186500 2.08254900 6.74192400

C 5.48349400 0.45983700 7.86124100

C 5.78925000 1.12807000 9.06315500

O 6.05460400 2.33583300 9.19651500

O 5.76624400 0.28582600 10.17514100

C 6.08510800 0.89500600 11.42031400

H 5.57149600 0.28231600 12.17219100

C 7.57391300 0.92577600 11.71514400

C 8.09370700 1.87749800 12.60069900

C 8.44143400 -0.01902400 11.15575100

C 9.45079000 1.88344900 12.92725400

H 7.43124300 2.62485200 13.03346400

C 9.80030700 -0.01142400 11.47525700

H 8.04290200 -0.74993100 10.45892500

C 10.31013600 0.93736500 12.36429500

H 9.83832900 2.63223000 13.61400900

H 10.46294400 -0.74869000 11.02817100

H 11.36847300 0.94280700 12.61271500

H 5.67802100 1.90981900 11.45809900

H 4.47096400 2.41777200 5.17255600

H 3.05561600 0.15169700 7.71988500

H 5.32257700 -0.61290900 7.89652900

C 0.35928000 3.48556500 5.33219600

C 0.61865100 4.34704700 4.26730100

C 0.37483200 3.92950500 2.91603100

C -0.18606400 2.63244500 2.68660100

C -0.50612100 1.81606400 3.79786200

C -0.25472100 2.20870600 5.09848800

H 1.14097200 5.70961100 1.93997700

C 0.69833400 4.73202500 1.78532300

C -0.42367600 2.19928200 1.35363900

H -0.98353900 0.85621400 3.61748700

C -0.10589600 3.00349000 0.28358500

C 0.46568500 4.27982100 0.50515600

H -0.85770600 1.21471500 1.19671000

H -0.28737200 2.66134300 -0.73155200

H 0.72455200 4.90732300 -0.34331800

C 1.18509800 5.71432400 4.52778900

C 0.36396500 6.87246300 4.32171900

C 2.49588100 5.84907500 4.98342700

C -1.00509200 6.79406000 3.93835900

C 0.93206700 8.16795400 4.54177700

C 3.07632600 7.15115000 5.14696600

C -1.76038600 7.93362200 3.77057200

H -1.45637400 5.82024900 3.78431000

C 0.12849700 9.32495100 4.35048300

C 2.28989800 8.26578800 4.92943900

C -1.18958300 9.21359000 3.97259200

H -2.80462200 7.85040400 3.48189600

H 0.57688400 10.30191000 4.51433700

H 2.72967500 9.25351900 5.04218600

H -1.79633100 10.10400800 3.83278500

C 4.51541800 7.35509000 5.49061500

C 5.53202400 6.89137200 4.63800400

C 4.88042400 8.07099000 6.64012800

C 6.87461700 7.13002000 4.93230600

H 5.26503000 6.35745700 3.72922800

C 6.22363900 8.30866200 6.93604300

H 4.10586000 8.41792800 7.31732400

C 7.22484400 7.83892300 6.08410100

H 7.64649000 6.76817700 4.25825400

H 6.48671200 8.85010300 7.84044400

H 8.27046300 8.02390300 6.31463600

C -0.68645600 1.30410800 6.20525000

C -1.61782200 1.73136100 7.16634700

C -0.22473900 -0.02007400 6.26593600

C -2.06359900 0.86444400 8.16450500

H -2.00964600 2.74409500 7.11849100

C -0.66756900 -0.88724100 7.26567500

H 0.49649100 -0.36263600 5.52955300

C -1.58723800 -0.44745500 8.21953800

H -2.79018000 1.21272700 8.89383500

H -0.28941300 -1.90527100 7.30156000

H -1.93320600 -1.12284800 8.99724400

C 3.26467000 4.59597000 5.34226500

H 3.15511500 3.84529000 4.55594700

C 0.78914400 3.90118700 6.72133900

H 0.37167400 3.27990700 7.51011700

P 2.63846300 3.81511500 6.91722400

C 3.14474600 4.97011400 8.25316400

C 2.22510000 5.88302900 8.76819800

C 4.48520900 4.96695100 8.67485000

C 2.64375800 6.81324000 9.73271400

H 1.18068900 5.89490400 8.48874400

C 4.89171300 5.92337700 9.61430100

H 5.17010500 4.19266900 8.34883800

C 3.97105700 6.84387600 10.14776100

H 4.33542000 7.55320300 10.88059500

O 1.66916300 7.65025100 10.19626900

O 6.15515200 6.06169600 10.08385400

C 7.19386900 5.24665000 9.52670600

H 8.10567700 5.55541000 10.04206000

H 7.00350100 4.18324500 9.68878900

H 7.29831500 5.44402000 8.45195200

C 2.01558600 8.58750200 11.20241800

H 1.09590800 9.12541700 11.44036800

H 2.39023400 8.08911300 12.10605100

H 2.77126000 9.30152700 10.84781800

H 4.32941900 4.79099900 5.48091100

H 0.49813900 4.93625000 6.91393100

**CP9**

C 4.39268300 -0.11414800 4.47942100

H 4.46690100 -0.69437800 3.55221300

H 3.42029500 -0.34850900 4.92994300

C 5.53622700 -0.42198100 5.46635300

H 5.35506800 -1.32988300 6.05444900

H 6.46401200 -0.59185000 4.90579000

C 4.51090900 1.39937500 4.25833100

H 3.60250700 1.83728300 3.83286800

H 5.33752800 1.60074100 3.55938300

C 3.66960100 2.23088800 6.60302800

C 3.61101700 1.06532300 7.57123800

C 4.85732900 1.98702800 5.65748800

C 2.80183400 1.22773900 8.83761100

O 2.24064100 2.24517400 9.20063500

O 2.71417400 0.06377100 9.51287100

C 1.97713400 0.11561400 10.74026300

H 0.94078800 0.40907700 10.55364000

H 2.43451300 0.82905600 11.43145600

H 2.02144500 -0.89373500 11.15179700

C 5.69919000 0.84406100 6.35637600

H 6.75179800 1.12433600 6.42884300

C 5.11905500 0.69719100 7.77605300

C 5.83080500 1.62103800 8.76139000

O 6.83136800 2.27114500 8.51990800

O 5.23199100 1.59664900 9.96933900

C 5.74824700 2.46749400 11.01419300

H 4.85542200 2.83045700 11.52721500

C 6.65515300 1.71783400 11.95808400

C 7.98078600 1.43160000 11.59919200

C 6.18473900 1.29416100 13.20644200

C 8.81327300 0.73229800 12.47255000

H 8.34892700 1.75685200 10.63040800

C 7.01803400 0.59776200 14.08411700

H 5.15902200 1.51451200 13.49432500

C 8.33451700 0.31449500 13.71745000

H 9.83879800 0.51676800 12.18403800

H 6.63959200 0.27793400 15.05148900

H 8.98587100 -0.22689200 14.39857200

H 6.25831100 3.30894800 10.54428100

H 5.47851000 2.88088200 5.52357400

H 3.16896800 0.16866900 7.10208900

H 5.22476300 -0.32140300 8.17249400

C 0.51552200 3.36081900 5.39357000

C 0.62451100 4.28457000 4.35556400

C 0.31147200 3.91248000 3.00556500

C -0.15290100 2.58347100 2.74680500

C -0.31059500 1.69040000 3.83312300

C 0.00397600 2.04542300 5.13101600

H 0.83729900 5.80015400 2.07404700

C 0.47121000 4.79464800 1.89926600

C -0.45780900 2.19559100 1.41363400

H -0.71112800 0.69890600 3.63649300

C -0.29876000 3.07594400 0.36840700

C 0.17538700 4.38674900 0.61716300

H -0.81587500 1.18415600 1.23593100

H -0.53122000 2.76833400 -0.64753500

H 0.30858500 5.07656600 -0.21194000

C 1.09799400 5.68050400 4.64441700

C 0.16384800 6.76733600 4.57300300

C 2.42844300 5.90608600 4.99735700

C -1.22115600 6.58269100 4.29819500

C 0.63084100 8.09723000 4.81985700

C 2.89969600 7.24999200 5.18554500

C -2.08579000 7.65425200 4.25808600

H -1.59632300 5.58011700 4.12576900

C -0.28445400 9.18339200 4.76184500

C 2.00288300 8.29788900 5.09971300

C -1.61534000 8.97018900 4.48646400

H -3.13960500 7.48865400 4.05069400

H 0.08931400 10.18811700 4.94494400

H 2.36383000 9.31508800 5.22975300

H -2.30796200 9.80659900 4.44794200

C 4.33669800 7.58799900 5.41274900

C 5.32005200 7.24297100 4.46991200

C 4.72228400 8.33800700 6.53455000

C 6.64801100 7.63299700 4.64591500

H 5.03530100 6.68462400 3.58181500

C 6.04982900 8.73063700 6.71091500

H 3.97705600 8.59428600 7.28184400

C 7.01766300 8.38041700 5.76707600

H 7.39156700 7.36298900 3.90051400

H 6.32857200 9.30278700 7.59166700

H 8.05096600 8.68899200 5.90205500

C -0.25884300 1.04739500 6.20966000

C -1.14960800 1.32927700 7.25903500

C 0.31581500 -0.23244200 6.14993800

C -1.44952000 0.36492600 8.22148400

H -1.62503600 2.30529700 7.30729100

C 0.01913900 -1.19730600 7.11394400

H 1.00710000 -0.46308200 5.34409900

C -0.86512700 -0.90227800 8.15340400

H -2.14945900 0.60113600 9.01885400

H 0.48084500 -2.17922100 7.05285500

H -1.10088000 -1.65467200 8.90132100

C 3.33534800 4.71500000 5.22101700

H 3.26889900 4.01354100 4.38555400

C 1.03173700 3.74861200 6.75822700

H 0.71799200 3.06865600 7.54789400

P 2.90505400 3.73507800 6.75567100

C 3.37710300 4.92418900 8.11988200

C 2.46881900 5.58467400 8.94084600

C 4.76039100 5.10293300 8.30910000

C 2.93611300 6.43392000 9.96009800

H 1.39778500 5.45401900 8.85077200

C 5.21496500 5.95822100 9.31305600

H 5.46187200 4.55090700 7.69501600

C 4.30245400 6.62803900 10.14786800

H 4.70001500 7.27511300 10.91998000

O 1.96515400 7.02722000 10.71727700

O 6.52874900 6.20968100 9.58065000

C 7.51239000 5.66923500 8.69899900

H 8.47142900 6.03020600 9.07702400

H 7.50277100 4.57445100 8.70446700

H 7.36325100 6.03517200 7.67581200

C 2.36749900 7.89694200 11.76073200

H 1.44686800 8.25300300 12.22744500

H 2.97624800 7.37503100 12.51137000

H 2.93437600 8.75563000 11.37599300

H 4.37846900 5.01732000 5.32010000

H 0.70074900 4.75692700 7.02049400

**H_2_O**

O -0.01674200 1.96617400 -0.02604500

H 0.95067100 2.01367800 -0.02604500

H -0.29489500 2.89395900 -0.02604500

**TS9**

C 1.14846100 -3.57223300 4.15967200

H 1.23141600 -2.90250900 5.02803400

H 1.01330500 -4.59000100 4.54275900

C 2.39137600 -3.43608200 3.26452800

H 2.43036200 -4.29009500 2.57621000

H 3.33662300 -3.42275400 3.81906500

C 0.01158500 -3.12181900 3.23096000

H -0.22709300 -3.89634600 2.49447800

H -0.91517900 -2.87967100 3.76408000

C 0.10284100 -1.66123900 1.04984800

C 1.24554700 -2.31228300 0.19898300

C 0.60115700 -1.87991600 2.51165000

C 1.39539000 -2.14561000 -1.28848200

O 2.45748500 -2.00475200 -1.86969700

O 0.22463100 -2.29328900 -1.95349700

C 0.32548900 -2.34049700 -3.38838200

H 0.73785000 -1.40622200 -3.77975800

H 0.97099800 -3.16806500 -3.69343900

H -0.69242600 -2.48838400 -3.74661300

C 2.15671700 -2.13009600 2.46229900

H 2.67780700 -1.30033000 2.95336200

C 2.57707100 -2.21342800 0.96357100

C 3.41973200 -1.03301600 0.53189700

O 3.00453200 0.10302500 0.35801800

O 4.71459700 -1.37369000 0.40137300

C 5.63454300 -0.37154800 -0.09276200

H 5.04631600 0.45673500 -0.49760700

C -2.95214500 -0.58119500 -0.53400100

C -2.73536700 0.09072200 -1.73471200

C -3.04020300 -0.55462600 -2.97864400

C -3.62963500 -1.86057100 -2.95057200

C -3.97592300 -2.43563500 -1.70143600

C -3.67032600 -1.82214400 -0.50441500

H -2.29272400 1.01320000 -4.27826000

C -2.74534200 0.02767700 -4.24369000

C -3.89811700 -2.52639300 -4.17780000

H -4.51843600 -3.37728500 -1.68967100

C -3.59366500 -1.93769500 -5.38481800

C -3.01015500 -0.64780200 -5.41514300

H -4.34893900 -3.51527100 -4.14343400

H -3.80039300 -2.45946700 -6.31528800

H -2.76867100 -0.18849100 -6.36993300

C -2.25561900 1.51630600 -1.72471800

C -3.19330400 2.55164400 -2.06451800

C -0.95069800 1.84031600 -1.35551000

C -4.56074600 2.29420400 -2.36729600

C -2.74801700 3.91169900 -2.06880200

C -0.49617500 3.20170900 -1.41459600

C -5.42624000 3.32199200 -2.67106000

H -4.92401000 1.27340800 -2.35004900

C -3.66284200 4.94923300 -2.39650600

C -1.39600400 4.19143400 -1.76173500

C -4.97534200 4.66374400 -2.69281300

H -6.46629900 3.10085700 -2.89465800

H -3.30366500 5.97560300 -2.40355000

H -1.05112600 5.22006400 -1.82842600

H -5.66839000 5.46362000 -2.93863400

C 0.92218000 3.60862100 -1.18932600

C 1.96889200 3.05173100 -1.94374200

C 1.22762000 4.64132100 -0.28764400

C 3.27807300 3.51159100 -1.80055400

H 1.75210000 2.27151500 -2.66816300

C 2.53547600 5.10733200 -0.14843000

H 0.43119000 5.07499200 0.31055300

C 3.56599800 4.54445700 -0.90542000

H 4.06972600 3.07245800 -2.40158700

H 2.74887700 5.90862800 0.55412000

H 4.58404300 4.91049600 -0.80196300

C -4.24345200 -2.36564900 0.77207400

C -5.37364600 -1.72610300 1.31306300

C -3.72591800 -3.50761800 1.40258200

C -5.98726200 -2.22089500 2.46432300

H -5.78051500 -0.84609300 0.81891600

C -4.35037100 -3.99307300 2.55656900

H -2.78950300 -3.95281800 1.02621500

C -5.47668600 -3.36223200 3.08773700

H -6.86391000 -1.72000400 2.86793600

H -3.94233600 -4.87466400 3.04570800

H -5.95361400 -3.75214300 3.98405300

C -0.04950300 0.72731100 -0.87028000

H -0.11206200 -0.13023300 -1.54301500

C -2.40757400 0.00643200 0.74595200

H -2.74468900 -0.56706400 1.61256500

P -0.54458100 0.03442200 0.77289900

C -0.14892100 1.23815400 2.10326600

C -1.13126200 1.58933200 3.03069200

C 1.15617600 1.75517800 2.21226500

C -0.82046100 2.48041500 4.07358900

H -2.14045900 1.20017300 2.99733400

C 1.45018700 2.63033800 3.26112700

H 1.91735000 1.45680100 1.50595500

C 0.46362300 3.00364000 4.19016400

H 0.74488500 3.68511900 4.98340300

O -1.84944900 2.76321300 4.91899700

O 2.67462500 3.17904800 3.47903200

C 3.75876500 2.75992900 2.65464300

H 3.60230900 3.04526200 1.60910500

H 4.63935400 3.27171500 3.04780400

H 3.90542400 1.67504500 2.71277400

C -1.60579400 3.64289200 6.00651800

H -2.55157200 3.71962200 6.54544700

H -0.83451300 3.24617300 6.67935400

H -1.30358400 4.63915500 5.65802000

H 1.00031000 1.00902000 -0.78430700

H -2.72241600 1.04673300 0.87611200

C 6.53850600 -0.98735800 -1.13133700

C 7.92265700 -0.80002600 -1.06230600

C 5.99736900 -1.72038000 -2.19751700

C 8.76129600 -1.32597200 -2.04796600

H 8.34949800 -0.24190200 -0.23144100

C 6.83661700 -2.25306200 -3.17536400

H 4.92398500 -1.88073700 -2.24873400

C 8.21897300 -2.05519300 -3.10637600

H 9.83541200 -1.17374100 -1.98179400

H 6.41015600 -2.82490900 -3.99544200

H 8.86884600 -2.47181600 -3.87147700

H 6.21237200 -0.00054800 0.76079200

H 0.40964700 -1.00171800 3.13500600

H 0.84812000 -3.35966300 0.27778600

H 3.17813100 -3.10880300 0.78646700

O -0.98242100 -4.02886000 0.49397000

H -1.13211200 -4.05724300 -0.46657000

H -0.73503400 -2.44970800 0.81339000

**CP13**

C -5.15555400 -0.55903900 -0.44622100

H -5.41034500 -1.59206300 -0.17368600

H -6.03869400 -0.11953000 -0.92280900

C -3.91299500 -0.55156700 -1.35058600

H -3.79459700 0.44211800 -1.80434400

H -3.96252100 -1.27987000 -2.16755900

C -4.69762400 0.21938600 0.80263700

H -4.79839100 1.29725300 0.62407200

H -5.28893900 -0.01629400 1.69322500

C -2.25663600 1.00945500 1.10564300

C -1.30251100 1.02899600 0.16388200

C -3.19245600 -0.15578400 0.97099900

C -0.26916800 2.08404700 0.08923000

O -0.21100200 3.06882300 0.79961700

O 0.63170100 1.81672900 -0.88959700

C 1.68484500 2.78005100 -1.03321900

H 1.27426400 3.76549500 -1.27007800

H 2.26688300 2.85298800 -0.11037100

H 2.30563900 2.41557600 -1.85231700

C -2.74758300 -0.82491700 -0.37734400

H -2.62788100 -1.90491200 -0.24623700

C -1.39044100 -0.16060200 -0.77850000

C -0.22821700 -1.12398200 -0.56346800

O 0.10676800 -1.57332600 0.51227700

O 0.36511000 -1.45406500 -1.73083800

C 1.46387100 -2.39341300 -1.62597300

H 2.24941800 -1.95271100 -1.00496800

C 1.95992900 -2.70499700 -3.01321500

C 3.29358300 -2.46828900 -3.36142400

C 1.09713000 -3.25863000 -3.96965200

C 3.76301800 -2.78379200 -4.63892200

H 3.96984100 -2.03436000 -2.62843400

C 1.56011300 -3.56627700 -5.24757300

H 0.05703700 -3.43817500 -3.70997100

C 2.89665300 -3.33188300 -5.58480700

H 4.80254400 -2.59615300 -4.89422100

H 0.88048000 -3.99225600 -5.98092300

H 3.25844000 -3.57530000 -6.58021300

H 1.10042900 -3.28790400 -1.10808600

H -3.06566000 -0.83924200 1.82053500

H -1.38328400 0.14615900 -1.82890600

H -2.34386100 1.76638500 1.87984700

**CP10**

C 0.11212300 -2.80203100 4.58419400

H 0.60994200 -2.36516400 5.46105400

H -0.65884000 -3.48912600 4.95190000

C 1.14821000 -3.48685000 3.67854500

H 0.62417400 -4.12674300 2.95412600

H 1.85432900 -4.12588600 4.22177700

C -0.44137500 -1.69464000 3.67426400

H -1.15104500 -2.13576700 2.96300200

H -0.97288800 -0.90714700 4.22110600

C 0.63979500 -0.80849000 1.41810700

C 1.02294600 -2.06878100 0.66536800

C 0.80069100 -1.13949900 2.90843400

C 1.33011200 -1.97014200 -0.81742500

O 1.94568800 -1.07726100 -1.38033400

O 0.84822200 -3.03589600 -1.48133800

C 1.12193800 -3.08445200 -2.89370000

H 0.69188900 -2.21442700 -3.39577200

H 2.19989400 -3.11624900 -3.06935800

H 0.64773900 -3.99995400 -3.24784700

C 1.84758900 -2.31479700 2.95427300

H 2.72701800 -2.00225100 3.52358600

C 2.25836600 -2.60875800 1.49851200

C 3.55216000 -1.90780600 1.12757700

O 4.01005500 -0.92567700 1.68717500

O 4.15084800 -2.50772700 0.08202300

C 5.33647400 -1.84114900 -0.44718600

H 5.09573300 -0.78443500 -0.58698800

C -2.56033300 -0.46424900 -0.58695200

C -2.44144800 0.30259200 -1.74868700

C -2.51732200 -0.30527200 -3.04499900

C -2.75757600 -1.71338900 -3.13424800

C -2.92805500 -2.45296600 -1.94026500

C -2.83720000 -1.87259200 -0.68879600

H -2.12521100 1.48491700 -4.20660400

C -2.32842100 0.42100400 -4.25571300

C -2.82729600 -2.33302600 -4.41199700

H -3.15685800 -3.51306700 -2.01562100

C -2.64915900 -1.59946000 -5.56260500

C -2.39134700 -0.20938300 -5.47901100

H -3.02263900 -3.40190500 -4.46128900

H -2.70205600 -2.08256000 -6.53453700

H -2.24070500 0.36491900 -6.38916200

C -2.21602700 1.78099500 -1.63736100

C -3.28249200 2.67698300 -1.98276000

C -0.99601500 2.26564000 -1.16458300

C -4.57261000 2.23117900 -2.38909200

C -3.05739300 4.08613200 -1.88269000

C -0.76485000 3.68469700 -1.11026000

C -5.57078500 3.13007500 -2.69500700

H -4.76969800 1.16673800 -2.44973900

C -4.10437200 4.98861000 -2.21426400

C -1.78665800 4.54562800 -1.46471600

C -5.33633600 4.52399800 -2.61348200

H -6.54852700 2.76629600 -2.99952000

H -3.91228100 6.05651700 -2.14081500

H -1.60475400 5.61710500 -1.44115400

H -6.13157500 5.22188400 -2.86121800

C 0.54504400 4.29333500 -0.73224900

C 1.72840900 3.97056400 -1.41535800

C 0.60121500 5.27484200 0.27156500

C 2.93174600 4.60076700 -1.09979700

H 1.70714000 3.22743300 -2.20715800

C 1.80228000 5.91148500 0.58365200

H -0.30244400 5.52215800 0.82155200

C 2.97236600 5.57675700 -0.10224100

H 3.83617500 4.32273000 -1.63257600

H 1.82569600 6.66174000 1.36954800

H 3.90930800 6.07090100 0.14137600

C -3.06711600 -2.75013000 0.49650800

C -4.05932600 -2.45867200 1.44866300

C -2.34069800 -3.94490200 0.63756600

C -4.30541300 -3.32498900 2.51389800

H -4.65752500 -1.55818700 1.33828100

C -2.58765000 -4.81262700 1.70335600

H -1.57012300 -4.18263000 -0.09062100

C -3.56834000 -4.50421300 2.64773000

H -5.08155000 -3.08278900 3.23512600

H -2.01073200 -5.72923400 1.79534400

H -3.76075500 -5.17823400 3.47798900

C 0.04174400 1.27899500 -0.67848800

H 0.19684300 0.47982200 -1.40538500

C -2.27926200 0.20271200 0.73362200

H -2.59911600 -0.39545400 1.58674600

P -0.39137800 0.45754700 0.95024200

C -0.22957400 1.76733600 2.22133000

C -1.30664100 2.26154400 2.95081600

C 1.07144500 2.26027400 2.43146800

C -1.08764700 3.26757000 3.91069500

H -2.31833100 1.89732700 2.81991600

C 1.27416100 3.25708300 3.38546700

H 1.89197900 1.85603700 1.85213800

C 0.19360900 3.76658200 4.12897900

H 0.40041700 4.53812000 4.86035800

O -2.20175700 3.68511400 4.58015000

O 2.48483500 3.80435900 3.67419600

C 3.62919800 3.28635500 2.99859900

H 3.55414700 3.42671800 1.91463500

H 4.47869500 3.85275100 3.38505300

H 3.77002000 2.22056300 3.21890200

C -2.05260300 4.69102900 5.56828000

H -3.05193400 4.86576500 5.97144400

H -1.38583000 4.36592900 6.37793800

H -1.66623200 5.62498700 5.13874400

H 1.01319300 1.74216700 -0.50531800

H -2.76683100 1.18009100 0.78802400

C 5.70564100 -2.50845600 -1.74350100

C 6.70451000 -3.48699800 -1.79192200

C 5.04274400 -2.15311900 -2.92723900

C 7.03909200 -4.10316000 -2.99967000

H 7.22513600 -3.76619600 -0.87864800

C 5.37422900 -2.76831800 -4.13480200

H 4.26443100 -1.39538500 -2.89119000

C 6.37382600 -3.74474800 -4.17323700

H 7.81945800 -4.85919300 -3.02394400

H 4.85999000 -2.48002000 -5.04807100

H 6.63583500 -4.21980600 -5.11503200

H 6.13483000 -1.92310300 0.29668900

H 1.18930800 -0.27850800 3.46234500

H 0.27139400 -2.86903500 0.75562100

H 2.39141700 -3.67762400 1.29893700

O 3.55955500 1.15578600 -0.28585600

H 2.93846300 0.51065400 -0.67317200

H 3.79271600 0.72626800 0.55566400

**TS10**

C 1.38141000 0.80780800 5.17343900

H 1.25554600 1.82292500 5.57341900

H 1.18403900 0.10346100 5.98963500

C 2.77953800 0.65012600 4.55796300

H 2.98604100 -0.41887300 4.40385800

H 3.58379100 1.04705400 5.18743800

C 0.46168900 0.59855200 3.96024500

H 0.42851800 -0.47734100 3.74122300

H -0.56704800 0.93274500 4.13016000

C 1.12425500 0.76981300 1.36740300

C 2.34664500 -0.17437300 1.36896300

C 1.15797000 1.37346900 2.80732400

C 2.77900400 -0.86351200 0.09121200

O 2.18135800 -0.90413900 -0.96621700

O 3.93099200 -1.53934400 0.29446400

C 4.47139300 -2.19804600 -0.86479500

H 3.76437200 -2.93716000 -1.25069500

H 4.69057200 -1.46441100 -1.64412600

H 5.38756400 -2.68097700 -0.52287900

C 2.68057800 1.38800700 3.20282200

H 3.03803000 2.41233400 3.31597800

C 3.43536500 0.72603500 2.02990600

C 4.05057200 1.81367200 1.13805500

O 4.47371700 2.85992900 1.58237000

O 4.13853700 1.46700100 -0.15815800

C 4.43137600 2.58070300 -1.05092500

H 3.55019200 3.23250500 -0.97773400

C -0.73064600 -2.50431800 0.52838000

C -1.13791800 -2.58764800 -0.80235800

C -0.54160500 -3.54601700 -1.68735300

C 0.43842600 -4.45012000 -1.16796700

C 0.76437700 -4.39038500 0.20743000

C 0.20712600 -3.45502000 1.05921200

H -1.58385900 -2.93107900 -3.48768500

C -0.85740600 -3.62085600 -3.07320500

C 1.04416300 -5.40032300 -2.03469900

H 1.45854000 -5.12416300 0.60927200

C 0.71156300 -5.44887800 -3.36876100

C -0.24511300 -4.54495600 -3.89069300

H 1.77902400 -6.08723300 -1.62147200

H 1.18281500 -6.17563000 -4.02471800

H -0.49685500 -4.58007500 -4.94710300

C -2.20944600 -1.67274500 -1.32603400

C -3.50669100 -2.21618100 -1.61398000

C -1.94787500 -0.31558400 -1.51299000

C -3.85451000 -3.57465200 -1.36585200

C -4.51322000 -1.34854700 -2.14549100

C -2.94610500 0.53486400 -2.09981800

C -5.12017700 -4.04456500 -1.63977900

H -3.11060200 -4.24430000 -0.94894500

C -5.80680800 -1.86626600 -2.42682100

C -4.18791900 0.00556500 -2.39511400

C -6.10760600 -3.18587900 -2.18036700

H -5.36397700 -5.08417800 -1.43823600

H -6.55680200 -1.19444000 -2.83739500

H -4.93858200 0.64419700 -2.85370300

H -7.10061500 -3.57110700 -2.39507600

C -2.69068400 1.95954300 -2.46372900

C -1.65248600 2.30794100 -3.34402000

C -3.54077500 2.97426400 -1.99706300

C -1.46787300 3.63195800 -3.74171600

H -0.99922700 1.53249600 -3.73539900

C -3.35831800 4.29870600 -2.39602200

H -4.33562000 2.72157500 -1.30108100

C -2.32158900 4.63198000 -3.27018100

H -0.66085000 3.88074700 -4.42539300

H -4.02083500 5.07138500 -2.01557300

H -2.17881900 5.66327000 -3.58139300

C 0.57226000 -3.52377500 2.50512900

C -0.41142700 -3.66237000 3.50029200

C 1.92169400 -3.53164400 2.89896000

C -0.05885000 -3.78643500 4.84449000

H -1.45934300 -3.69950500 3.21398800

C 2.27446700 -3.65771200 4.24424500

H 2.69656500 -3.42035600 2.14511900

C 1.28590000 -3.78137500 5.22216100

H -0.83591000 -3.89847700 5.59586900

H 3.32393600 -3.65659700 4.52643800

H 1.56044900 -3.87826100 6.26885200

C -0.62634000 0.23759100 -1.03145100

H 0.22671500 -0.36314100 -1.35293500

C -1.25055900 -1.35285200 1.36434200

H -1.03462400 -1.47089700 2.42516000

P -0.53304300 0.28373200 0.81593800

C -1.66552700 1.60659700 1.36551400

C -2.80222300 1.34613500 2.13157200

C -1.31486100 2.91289200 0.97963800

C -3.61343500 2.42260700 2.53203700

H -3.09011500 0.35088900 2.44863100

C -2.12996000 3.96678100 1.40029300

H -0.39485100 3.07853300 0.39476800

C -3.28245500 3.72727400 2.17215200

H -3.88035300 4.57915900 2.47254700

O -4.70552800 2.08101500 3.27859600

O -1.87908400 5.26830000 1.11509900

C -0.73802200 5.56387700 0.29844500

H -0.87469800 5.17165300 -0.71473300

H -0.67816100 6.65377000 0.27395500

H 0.18396400 5.13719400 0.70444400

C -5.56039100 3.11922800 3.72856000

H -6.35299800 2.62921800 4.29733800

H -5.02948300 3.82594800 4.37997400

H -6.00387500 3.66994800 2.88836300

H -0.43859200 1.26728900 -1.33928500

H -2.33475600 -1.25643000 1.24968700

C 4.65586300 2.05913400 -2.44422100

C 5.78176300 2.46193800 -3.17218400

C 3.72382600 1.20863600 -3.05793700

C 5.97447000 2.03442100 -4.48804400

H 6.51360400 3.11695100 -2.70467000

C 3.92188600 0.76974600 -4.36739500

H 2.85590000 0.87854800 -2.49690600

C 5.04521900 1.18427500 -5.08877100

H 6.85353100 2.35972500 -5.03868400

H 3.19499800 0.10455300 -4.82705800

H 5.19508000 0.84495900 -6.11061100

H 5.30939300 3.11260300 -0.67808000

H 0.76532400 2.39261300 2.77492500

H 2.18998400 -0.99871400 2.08147700

H 4.27080100 0.10970900 2.38335500

O 1.38284000 2.90300400 -0.27319000

H 1.31813100 1.68887000 0.57067500

H 1.40694800 2.62475800 -1.20391800

**CP11**

C 1.24706600 -3.08766400 3.15166200

H 1.38402600 -2.59639300 4.12477200

H 0.78288900 -4.06252500 3.33627900

C 2.59006300 -3.18695900 2.41469700

H 2.49821800 -3.91103400 1.59312600

H 3.41978700 -3.52186600 3.04683400

C 0.41309400 -2.19700400 2.21815700

H 0.05379700 -2.79195200 1.37001700

H -0.46432000 -1.77413700 2.71712200

C 1.28137400 -0.59600600 0.27190400

C 2.17134100 -1.59644700 -0.50513900

C 1.39832400 -1.09860500 1.74343400

C 2.82134200 -1.76654000 1.84280100

H 3.46764700 -1.20288400 2.52297500

C 3.41431300 -1.75610800 0.40202700

C 4.40840400 -0.61671300 0.23583900

O 4.19462800 0.45345800 -0.30361800

O 5.58739700 -0.93107900 0.80862000

C 6.60764500 0.10443700 0.76642800

H 6.78401600 0.37025300 -0.27997000

C -3.09315100 -1.21003000 -0.69775600

C -3.60487000 -0.27614600 -1.58998300

C -4.36187500 -0.70118600 -2.73621800

C -4.62716900 -2.09682000 -2.90991900

C -4.14474200 -3.01219600 -1.94094300

C -3.39560200 -2.60267300 -0.86002700

H -4.63744800 1.25782900 -3.62692500

C -4.84242700 0.19812100 -3.72970300

C -5.37041900 -2.53390200 -4.03874100

H -4.37877600 -4.06770900 -2.05925400

C -5.82653900 -1.63596300 -4.97713700

C -5.55260600 -0.25685800 -4.81984800

H -5.56798900 -3.59784200 -4.14942300

H -6.39115800 -1.98200100 -5.83885400

H -5.90449600 0.45047100 -5.56630600

C -3.38190700 1.19608400 -1.38201100

C -4.50602400 1.99564100 -0.97966600

C -2.13364200 1.78209000 -1.60498900

C -5.76629400 1.43479200 -0.62631700

C -4.35514700 3.41460000 -0.89493200

C -2.02745600 3.21874300 -1.63263900

C -6.81095400 2.23466000 -0.21752800

H -5.89533300 0.35915800 -0.66896100

C -5.45441400 4.21536900 -0.48235200

C -3.11889000 3.98648800 -1.27199500

C -6.65885200 3.64048100 -0.14803700

H -7.76077200 1.78247700 0.05544200

H -5.32197600 5.29372200 -0.43208800

H -3.04229500 5.06986800 -1.31387100

H -7.49242000 4.26017900 0.17162100

C -0.83487600 3.94938800 -2.15332500

C -0.27537800 3.62722400 -3.40255200

C -0.32389800 5.06505400 -1.46835000

C 0.75997500 4.38970000 -3.94424200

H -0.67480400 2.78929500 -3.96679600

C 0.70405500 5.83441000 -2.01391600

H -0.72909300 5.31837800 -0.49349000

C 1.25271400 5.49913900 -3.25409400

H 1.17100800 4.12378600 -4.91470000

H 1.08198400 6.69157900 -1.46271500

H 2.05425300 6.09742900 -3.67930300

C -2.93415900 -3.63471200 0.11673600

C -3.46825800 -3.69530800 1.41385700

C -1.98979800 -4.59997100 -0.26425300

C -3.06948700 -4.69233000 2.30599700

H -4.21091600 -2.96193800 1.71842100

C -1.59269600 -5.59956100 0.62602900

H -1.55697400 -4.54443300 -1.25799700

C -2.13059300 -5.64924200 1.91422100

H -3.49920700 -4.72608700 3.30394000

H -0.86080700 -6.34024400 0.31311600

H -1.82376100 -6.42936800 2.60613800

C -0.91766600 0.89544700 -1.80769000

H -1.08422300 0.19193200 -2.62483900

C -2.18589400 -0.72949300 0.40575100

H -2.00969900 -1.50944200 1.14466000

P -0.51797300 -0.17267200 -0.28894600

C -0.35612900 1.39724100 0.93287700

C -1.02660000 1.51837300 2.15056900

C 0.62675500 2.36687200 0.63031200

C -0.75573700 2.58107200 3.03414900

H -1.76954200 0.80380800 2.48577700

C 0.92699000 3.39410000 1.52472300

H 1.17686900 2.31282300 -0.30238300

C 0.22166800 3.52350300 2.73274900

H 0.48351100 4.33612400 3.39890400

O -1.49705700 2.58388600 4.18496000

O 1.90555900 4.32982100 1.32450700

C 2.88598600 4.06790100 0.32567400

H 2.46997800 4.15010900 -0.68437600

H 3.65298900 4.83522400 0.45714400

H 3.33344100 3.07395100 0.45292100

C -1.28630100 3.63306900 5.11197800

H -1.98245000 3.45407500 5.93419000

H -0.25877600 3.63134000 5.50124300

H -1.49623800 4.61493500 4.66646300

H -0.03134500 1.47857600 -2.05362000

H -2.63594700 0.12497800 0.90690300

C 7.84690400 -0.43134300 1.42910700

C 8.78170100 -1.17162500 0.69421900

C 8.07772800 -0.21072000 2.79234700

C 9.92464500 -1.68286600 1.30906000

H 8.61100800 -1.34703800 -0.36546600

C 9.22037300 -0.71963500 3.41108600

H 7.35853600 0.36462400 3.37076500

C 10.14592000 -1.45693400 2.66972300

H 10.64355900 -2.25335900 0.72720700

H 9.38933200 -0.53838100 4.46910000

H 11.03795100 -1.85096100 3.14924400

H 6.22204600 0.99185600 1.27700800

H 1.32943200 -0.24379100 2.42060800

H 3.95588400 -2.68480400 0.20185400

H 1.80332700 0.35938600 0.23463300

C 2.47838700 -1.23682000 -1.94493900

O 1.73358800 -0.62927400 -2.69468900

O 3.65598300 -1.74666200 -2.35502700

C 4.01026000 -1.47332400 -3.72159500

H 3.24827200 -1.86375100 -4.40080000

H 4.11226700 -0.39645000 -3.87737900

H 4.96377000 -1.97748100 -3.88131700

H 1.65636200 -2.56494600 -0.54934100

O -0.54715600 -1.68372400 -1.23104200

H 0.03886000 -1.57059200 -1.99788100

**TS11**

C -2.00365400 1.45094900 4.49349200

H -1.81860600 0.79193200 5.35268700

H -1.91178100 2.48254500 4.84924900

C -3.38681100 1.14219900 3.87705400

H -3.79754700 2.04450500 3.40867800

H -4.11442800 0.81774700 4.62866600

C -1.00937700 1.11981900 3.36884000

H -1.00328000 1.91025100 2.61117300

H 0.01086000 1.00315400 3.75163300

C -1.28210100 -0.63390500 1.33354100

C -2.37990600 -0.07739200 0.43400000

C -1.58513000 -0.18459000 2.78407500

C -3.12475700 0.05688400 2.79513500

H -3.64622900 -0.87713100 3.02395700

C -3.51237700 0.47390900 1.34366100

C -4.89807000 -0.05964500 1.01565700

O -5.25524000 -1.21180000 1.15230800

O -5.71393700 0.93469000 0.58580300

C -7.03855700 0.56158400 0.16474900

H -7.03795000 -0.50929800 -0.06244400

C 1.99291200 2.03148200 0.16807100

C 2.60446200 1.64953700 -1.01941200

C 2.75555100 2.59095800 -2.09874700

C 2.34706500 3.94581500 -1.89415900

C 1.83554800 4.32115300 -0.62581200

C 1.65321800 3.41004800 0.39085400

H 3.56538300 1.20614800 -3.56069400

C 3.26515600 2.23183200 -3.37790900

C 2.47848300 4.88519400 -2.95095700

H 1.58513800 5.36499800 -0.45357700

C 2.97923000 4.50528600 -4.17604700

C 3.37036500 3.16303500 -4.38925200

H 2.16865800 5.91252900 -2.77405500

H 3.07072600 5.23074100 -4.97987100

H 3.75600300 2.86222200 -5.35971000

C 3.13137700 0.25802500 -1.21787000

C 4.55513000 0.07094100 -1.26098700

C 2.26635900 -0.82092400 -1.39718500

C 5.48232200 1.11419800 -0.98044400

C 5.07222100 -1.22616100 -1.56797800

C 2.79240000 -2.09954600 -1.79859700

C 6.84011700 0.88268600 -1.00769600

H 5.10822100 2.10099200 -0.73225100

C 6.47831900 -1.43113400 -1.60147500

C 4.16321600 -2.26657600 -1.86882100

C 7.34693200 -0.40036800 -1.32603100

H 7.52924900 1.69219100 -0.78280000

H 6.85397800 -2.42206600 -1.84548400

H 4.56282400 -3.22213100 -2.19793200

H 8.42037700 -0.56746100 -1.34903800

C 1.94504800 -3.24014000 -2.25290200

C 0.94773800 -3.06545800 -3.22752400

C 2.21132800 -4.54514300 -1.80365600

C 0.23884700 -4.15578400 -3.73181200

H 0.74353300 -2.07102500 -3.61390200

C 1.51123400 -5.63747100 -2.31592600

H 2.96627400 -4.69831500 -1.03809300

C 0.51983900 -5.44709500 -3.28171000

H -0.52620800 -3.99516300 -4.48639900

H 1.73644600 -6.63709300 -1.95339600

H -0.02751700 -6.29702700 -3.68034000

C 1.16139100 3.92301200 1.70415400

C 1.99936200 3.92812000 2.83056100

C -0.11797100 4.48857200 1.81479600

C 1.57135600 4.48495700 4.03735100

H 3.00088600 3.51117400 2.75350000

C -0.54308900 5.05164400 3.01902600

H -0.78429000 4.44284200 0.96003000

C 0.29941100 5.05266300 4.13375500

H 2.23632300 4.48502200 4.89722900

H -1.53742600 5.48543400 3.08819300

H -0.03295400 5.49273300 5.07030800

C 0.79135600 -0.62581700 -1.10276600

H 0.38793800 0.27004300 -1.57673000

C 1.71110200 0.99134000 1.22868900

H 1.32895000 1.45178500 2.13664100

P 0.51522200 -0.36338700 0.72234900

C 1.24083500 -1.89172700 1.50242900

C 2.21667800 -1.85012800 2.49501700

C 0.67710100 -3.13029700 1.12599900

C 2.65445100 -3.04087500 3.10594700

H 2.66302300 -0.92884600 2.84758100

C 1.10054200 -4.30115600 1.75668400

H -0.08303500 -3.17245100 0.35509000

C 2.10284500 -4.26463500 2.74191400

H 2.39716600 -5.19879500 3.20378300

O 3.61559200 -2.88115700 4.05952700

O 0.59763900 -5.53761800 1.49274600

C -0.59469600 -5.62807700 0.71506900

H -0.42612000 -5.32087300 -0.32190500

H -0.88082100 -6.68140600 0.73911900

H -1.39688200 -5.01977800 1.15041600

C 4.09415800 -4.03602500 4.73090400

H 4.84441900 -3.68073200 5.43955600

H 3.29013500 -4.54608900 5.27756800

H 4.56010500 -4.74370500 4.03266500

H 0.16683700 -1.46923400 -1.39314500

H 2.63548500 0.46298600 1.47596100

C -7.44357000 1.39199400 -1.03003900

C -6.50986900 1.74458000 -2.01371900

C -8.77878700 1.77888500 -1.19048900

C -6.91143600 2.46765700 -3.13768800

H -5.47053500 1.45898400 -1.88401000

C -9.18182700 2.49532600 -2.31922600

H -9.50914100 1.52044500 -0.42633600

C -8.24795100 2.84260900 -3.29661800

H -6.17704700 2.74128500 -3.89114800

H -10.22244100 2.78973800 -2.42865400

H -8.55769000 3.40650300 -4.17262000

H -7.72643000 0.72189800 1.00327300

H -1.32837800 -1.00185900 3.46972000

H -3.55561900 1.56537900 1.26621100

H -1.29939800 -1.72563500 1.31051900

C -2.68860700 -0.95268300 -0.69044600

O -2.02169600 -1.92125900 -1.06054700

O -3.78358400 -0.54887300 -1.40325100

C -4.17358100 -1.40953900 -2.47739300

H -4.40857600 -2.41098800 -2.10566900

H -5.06162400 -0.94922300 -2.91401600

H -3.37766200 -1.48734800 -3.22376100

H -1.71529000 0.97052600 0.01800500

O -0.72775700 1.79333300 0.04064500

H -0.63917200 2.16267000 -0.85204800

**CP12**

C 0.76675200 -1.77927700 4.68227400

H 0.95878300 -1.10464600 5.52813000

H 0.14570500 -2.60414700 5.04978800

C 2.08846500 -2.24784300 4.05418400

H 1.88863800 -3.11702200 3.41429900

H 2.84046600 -2.54869000 4.79246300

C 0.11348200 -1.00996000 3.52446400

H -0.24830100 -1.74186000 2.79197800

H -0.73077200 -0.38485300 3.83889100

C 1.26432600 -0.00572200 1.35955700

C 1.96601600 -1.21913000 0.83647400

C 1.27320200 -0.19369900 2.91204100

C 2.56008600 -1.05951400 3.18077000

H 3.30057400 -0.46165900 3.72071400

C 3.11275800 -1.48788200 1.79118800

C 4.26551300 -0.59424400 1.36363900

O 4.33010400 0.61242600 1.54585900

O 5.20602900 -1.28155700 0.67150200

C 6.23541200 -0.50804500 0.03557900

H 5.78847600 0.41357400 -0.35505800

C -2.12902900 -1.20353900 -0.63895000

C -2.36518800 -0.52272000 -1.82754900

C -2.46497600 -1.24501600 -3.06446500

C -2.38784500 -2.67373900 -3.03698900

C -2.22187200 -3.33035100 -1.79384800

C -2.08258800 -2.63383900 -0.61329600

H -2.64830100 0.47671300 -4.37316900

C -2.61213100 -0.60605400 -4.32787600

C -2.48749200 -3.40290800 -4.25254600

H -2.19807300 -4.41714600 -1.77613100

C -2.63789900 -2.75372600 -5.45628100

C -2.69377900 -1.33995900 -5.49094800

H -2.43358500 -4.48831600 -4.21214700

H -2.70700700 -3.32113100 -6.38045100

H -2.79885400 -0.82881800 -6.44420900

C -2.52337500 0.97187600 -1.84391000

C -3.82371200 1.52256200 -2.11110000

C -1.43435600 1.81401300 -1.61127600

C -4.98937800 0.71753700 -2.25588700

C -3.97109400 2.94250200 -2.20390600

C -1.57728200 3.23575500 -1.77684500

C -6.22190300 1.28765000 -2.48727900

H -4.90061500 -0.35969100 -2.17462200

C -5.25382600 3.50048900 -2.45619600

C -2.82399900 3.75797300 -2.06372200

C -6.35869000 2.69265700 -2.59485000

H -7.09810700 0.65302200 -2.58851900

H -5.34481000 4.58147700 -2.53237300

H -2.92824300 4.83000200 -2.21027400

H -7.33613000 3.12838800 -2.78303200

C -0.42689400 4.18537800 -1.72853100

C 0.70175900 4.00399900 -2.54714900

C -0.48966300 5.33846000 -0.92903700

C 1.73531300 4.94111800 -2.56016000

H 0.75655000 3.13549500 -3.19810200

C 0.53966400 6.28070600 -0.94715000

H -1.34821100 5.48655400 -0.28038000

C 1.65777500 6.08463700 -1.76148800

H 2.59509000 4.78364000 -3.20577000

H 0.46942900 7.16501800 -0.31929400

H 2.45910600 6.81845900 -1.77788000

C -1.91630600 -3.40441700 0.65665200

C -2.94662700 -3.44812500 1.61097600

C -0.74442900 -4.13793600 0.89766200

C -2.81015800 -4.20377400 2.77647300

H -3.86642800 -2.89741000 1.42775500

C -0.61151900 -4.89884100 2.06155300

H 0.06895700 -4.09053800 0.18173500

C -1.64103300 -4.93342100 3.00434700

H -3.62019700 -4.22968000 3.50084200

H 0.30317400 -5.46039300 2.23235900

H -1.53463300 -5.52615100 3.90906100

C -0.13597700 1.21137700 -1.11210100

H 0.18603600 0.31878800 -1.66226100

C -1.89961600 -0.40955500 0.62660500

H -1.88912500 -1.04289100 1.51352200

P -0.33397000 0.59799600 0.62298900

C -0.68031900 2.07337100 1.66891000

C -1.84743900 2.20387500 2.41723800

C 0.32501900 3.05866800 1.74991200

C -2.02567000 3.32628500 3.24823900

H -2.64035100 1.46689000 2.40649000

C 0.14729900 4.15574000 2.59477800

H 1.23032800 2.96957500 1.16451700

C -1.03521700 4.29948000 3.34147100

H -1.12955500 5.16749500 3.98193400

O -3.20358200 3.35767800 3.93038100

O 1.06326400 5.14405400 2.76862300

C 2.34452400 4.99520100 2.16013700

H 2.27265200 5.02867100 1.06706500

H 2.93540200 5.84347500 2.51005400

H 2.82669200 4.06024900 2.47070800

C -3.44897000 4.45131600 4.80196200

H -4.43475800 4.27259800 5.23458200

H -2.70231500 4.50035500 5.60514900

H -3.45775400 5.40472700 4.25788900

H 0.68287400 1.93275200 -1.10427000

H -2.69737600 0.32881400 0.74993400

C 6.86518100 -1.33135200 -1.06281500

C 6.13060000 -2.30596200 -1.75013700

C 8.19178900 -1.08673700 -1.43721300

C 6.71879500 -3.01731000 -2.79730400

H 5.10801600 -2.50920900 -1.44691100

C 8.77613100 -1.79345800 -2.48939600

H 8.77348100 -0.33946400 -0.90070600

C 8.03968000 -2.76270700 -3.17325800

H 6.14163200 -3.77522300 -3.32120300

H 9.80776000 -1.59337700 -2.76777000

H 8.49381400 -3.31949200 -3.98892400

H 6.98033600 -0.20989700 0.78341000

H 1.34487800 0.77568700 3.41595600

H 3.44471200 -2.52945200 1.76714700

H 1.86122000 0.90718000 1.15922800

C 1.67350300 -1.83884600 -0.36184700

O 0.70728300 -1.56239300 -1.12410600

O 2.51336900 -2.90341500 -0.69817100

C 2.23288900 -3.51362500 -1.95263500

H 1.21933200 -3.92699900 -1.99162100

H 2.33760400 -2.80251200 -2.77993100

H 2.96588800 -4.31815500 -2.05970700

**TS12**

C 1.56442800 4.29160700 1.93387600

H 0.96636900 5.21187700 1.92013900

H 1.95768600 4.18356900 2.95043300

C 2.68042900 4.36643000 0.85599400

H 3.63605200 3.99378800 1.24235900

H 2.85328300 5.40056500 0.53878700

C 0.67857700 3.10104900 1.51845300

H 1.13315900 2.16164000 1.86058500

H -0.32691900 3.16291400 1.94400500

C 0.44712600 1.84679100 -0.81071700

C 1.74436400 1.21038900 -0.98502200

C 0.70089500 3.15383000 -0.02707300

C 1.88886500 0.04073400 -1.75039300

O 0.97809800 -0.63043500 -2.26981500

O 3.21066100 -0.37898500 -1.89609200

C 3.39071700 -1.47995100 -2.78422400

H 2.82577900 -2.35583700 -2.45171300

H 3.06893700 -1.22641000 -3.79981900

H 4.46260100 -1.69457200 -2.77868700

C 2.18610000 3.48544400 -0.31920700

H 2.29855800 3.97726400 -1.28995400

C 2.85746500 2.08440000 -0.42577300

C 4.08294100 2.17495400 -1.32634600

O 4.11666000 2.66686400 -2.43387300

O 5.18655900 1.68331700 -0.70184800

C 6.39567800 1.66864900 -1.48558100

H 6.13390800 1.40277600 -2.51499900

C -0.18825700 -1.73696800 0.92199000

C -0.99247600 -2.57183400 0.15046500

C -0.43472800 -3.72448300 -0.49563300

C 0.94836400 -4.02711000 -0.29079000

C 1.71157300 -3.19986000 0.56685100

C 1.18146100 -2.07924300 1.17466100

H -2.23310500 -4.34652400 -1.53759100

C -1.18802300 -4.57131400 -1.35619000

C 1.51810100 -5.16137800 -0.93029700

H 2.74600600 -3.46845000 0.76492500

C 0.76019400 -5.96190100 -1.75371900

C -0.60531000 -5.65820100 -1.97004500

H 2.56904700 -5.38153400 -0.75823200

H 1.20655100 -6.82385400 -2.24211400

H -1.19809000 -6.28680100 -2.62907700

C -2.45824000 -2.28211600 -0.00722300

C -3.40864000 -3.12520100 0.66155100

C -2.88614200 -1.20366300 -0.78103000

C -3.02648100 -4.18619100 1.53122800

C -4.80565000 -2.87575400 0.47630700

C -4.29063700 -0.99737700 -1.00877500

C -3.97349300 -4.95741700 2.16844800

H -1.97274800 -4.38100900 1.69528600

C -5.75845300 -3.69637600 1.13899000

C -5.20361200 -1.82366900 -0.38165600

C -5.35439200 -4.71660700 1.96877200

H -3.65811600 -5.75893900 2.83095400

H -6.81576000 -3.49788400 0.97967300

H -6.26484100 -1.67872200 -0.56802200

H -6.09021400 -5.33632500 2.47390700

C -4.82046200 0.03768100 -1.94481900

C -4.42375600 0.07047700 -3.29322100

C -5.79400400 0.95375200 -1.51467400

C -4.97906800 0.99337400 -4.18024100

H -3.69210500 -0.64818200 -3.65287100

C -6.35344500 1.87460700 -2.40164400

H -6.10004400 0.95011600 -0.47241200

C -5.94760800 1.89852300 -3.73814500

H -4.66436500 0.99521400 -5.22046000

H -7.10271200 2.57699400 -2.04622900

H -6.38679000 2.61134200 -4.43112100

C 2.05330800 -1.30876800 2.10994300

C 1.72109700 -1.17595800 3.46943700

C 3.26757300 -0.76813400 1.65969000

C 2.57722400 -0.51426200 4.35119800

H 0.79841500 -1.61582500 3.84099200

C 4.12450900 -0.10712400 2.54228700

H 3.52191700 -0.84026900 0.60697600

C 3.78175400 0.02234600 3.88996500

H 2.30750000 -0.42838700 5.40080900

H 5.05292500 0.31191300 2.16448500

H 4.44864900 0.53645200 4.57711200

C -1.85324400 -0.22948900 -1.30068200

H -0.99490400 -0.70011500 -1.79499800

C -0.77387200 -0.44047700 1.44235400

H -0.11674700 0.04627600 2.16398500

P -1.09968400 0.77711300 0.06599400

C -2.42040000 1.91029500 0.63041600

C -2.89168300 1.91695300 1.94233000

C -2.88754600 2.86561900 -0.29808400

C -3.85051900 2.86854100 2.33566900

H -2.54927800 1.21150000 2.68980100

C -3.83337600 3.80817000 0.10781300

H -2.52469200 2.85936600 -1.31808300

C -4.32266600 3.81161300 1.42627400

H -5.05540400 4.56271000 1.69326200

O -4.25362500 2.78225200 3.63336700

O -4.35229800 4.77476400 -0.69659700

C -3.86737800 4.88024800 -2.02994500

H -4.09805600 3.98010100 -2.61176300

H -4.38562600 5.73631900 -2.46567900

H -2.78556200 5.06482600 -2.04702900

C -5.21852400 3.71186600 4.10306200

H -5.38795900 3.46185300 5.15174000

H -4.85173300 4.74388400 4.02957900

H -6.16331900 3.62419700 3.55087000

H -2.28266500 0.48617500 -2.00442600

H -1.73992100 -0.62024900 1.92602600

C 7.35544100 0.67822200 -0.87675000

C 6.93371600 -0.62713800 -0.58492100

C 8.68586300 1.03088600 -0.62967600

C 7.82799100 -1.55741200 -0.05711700

H 5.89766700 -0.89871700 -0.76708500

C 9.58590600 0.09735800 -0.10919600

H 9.02112500 2.04317600 -0.84482100

C 9.15861700 -1.19883500 0.17964200

H 7.48806600 -2.56495300 0.16875300

H 10.61699900 0.38674400 0.07685500

H 9.85537700 -1.92537400 0.58949600

H 6.82494200 2.67694900 -1.50801500

H 0.03044000 3.95488600 -0.36285200

H 3.21693100 1.76395800 0.56160500

H -0.13267300 1.99006600 -1.73488300

**TS13**

C 1.50913900 -1.15430800 5.48787800

H 1.88490300 -0.28289400 6.04195300

H 1.20699500 -1.90472700 6.22776900

C 2.58921600 -1.65765400 4.51773600

H 2.30662400 -2.64747200 4.13078000

H 3.58057000 -1.75498400 4.97518800

C 0.36370100 -0.74111500 4.54488200

H -0.22240100 -1.62636700 4.27016700

H -0.33084900 -0.02794900 5.00431800

C 0.54181900 -0.66356900 1.91626200

C 1.66759100 -1.59516200 1.34455800

C 1.05743600 -0.15675200 3.27360900

C 1.51669500 -2.47721600 0.23557300

O 0.45605400 -2.75855800 -0.34502400

O 2.69993400 -3.09152200 -0.12506000

C 2.55931600 -4.12461000 -1.10060200

H 1.96206400 -4.95705100 -0.71258700

H 2.07903500 -3.75186500 -2.00984300

H 3.57436900 -4.46545800 -1.31644100

C 2.56139600 -0.62282700 3.37602300

H 3.19129300 0.23008400 3.65237600

C 2.97974500 -1.17434000 1.99460300

C 3.59017700 -0.08585600 1.13157400

O 3.18688700 1.06664000 1.06191100

O 4.62268700 -0.53707300 0.38578700

C 5.17763200 0.39401800 -0.56396600

H 4.35191100 0.91937900 -1.05807900

C -2.60241300 -1.56531000 -0.01076700

C -2.67328900 -1.22224100 -1.35879000

C -2.81340600 -2.23956000 -2.36187900

C -2.92695500 -3.60448800 -1.94751600

C -2.87543400 -3.90944600 -0.56691800

C -2.70441200 -2.93503900 0.39341700

H -2.70626100 -0.93856900 -4.09635500

C -2.81788300 -1.96264600 -3.75866400

C -3.07572100 -4.62437300 -2.92596100

H -2.96184300 -4.94860400 -0.25940400

C -3.09201800 -4.31981000 -4.26739200

C -2.95365100 -2.97404400 -4.68404200

H -3.16703800 -5.65491500 -2.59079400

H -3.20175900 -5.10758000 -5.00784900

H -2.95118100 -2.73690100 -5.74467200

C -2.56141100 0.21355300 -1.78302300

C -3.70866100 0.87705100 -2.33553800

C -1.34971300 0.89469500 -1.64036300

C -4.99378700 0.26798600 -2.40606700

C -3.57447100 2.22266400 -2.80339200

C -1.20557300 2.21897700 -2.18221900

C -6.07513500 0.94883200 -2.92104400

H -5.11767200 -0.74482400 -2.03949900

C -4.70588400 2.89446500 -3.34035600

C -2.30457500 2.84350200 -2.73969400

C -5.93245200 2.27368600 -3.39938500

H -7.04748600 0.46512800 -2.95964700

H -4.58303000 3.91350900 -3.69978900

H -2.18969300 3.83674400 -3.16650900

H -6.79272400 2.79725800 -3.80752300

C 0.10355300 2.93311600 -2.24619900

C 1.21544200 2.34973400 -2.87838900

C 0.22736000 4.24388500 -1.75885500

C 2.41335800 3.05206800 -3.01285300

H 1.13023000 1.34738900 -3.28963600

C 1.42360900 4.94984500 -1.89737600

H -0.61822500 4.70398000 -1.25569800

C 2.52184400 4.35623300 -2.52347200

H 3.25668300 2.58531200 -3.51527900

H 1.49716500 5.96259300 -1.50982900

H 3.45265200 4.90616500 -2.63392600

C -2.60663100 -3.36289500 1.82154300

C -3.61077600 -3.06338900 2.75551500

C -1.50635400 -4.12982100 2.23689000

C -3.51565900 -3.51456500 4.07326700

H -4.47829500 -2.48772000 2.44163100

C -1.41380500 -4.58394900 3.55417700

H -0.72400300 -4.34813100 1.51583000

C -2.41678800 -4.27644000 4.47656700

H -4.30477900 -3.27804600 4.78244900

H -0.55579500 -5.17711400 3.86037200

H -2.34432500 -4.62962100 5.50182600

C -0.23499000 0.25125200 -0.84537200

H -0.08266500 -0.80239700 -1.10515800

C -2.37224600 -0.46837100 1.00108200

H -2.55761200 -0.80409600 2.02287700

P -0.62712300 0.21331500 0.97210600

C -0.79771400 1.94076300 1.58692400

C -1.98683200 2.45427700 2.10019300

C 0.37191300 2.72607000 1.56195400

C -2.02401300 3.77698800 2.57881600

H -2.90147400 1.87813600 2.15735300

C 0.32060100 4.03496600 2.04492300

H 1.30103300 2.29741300 1.20572400

C -0.87992500 4.56882300 2.54827600

H -0.86740300 5.58727000 2.91648200

O -3.23317300 4.18722000 3.05839100

O 1.38434200 4.88073900 2.08271700

C 2.65892700 4.37567400 1.68593700

H 2.66686900 4.09856000 0.62636700

H 3.36196200 5.19312300 1.85819800

H 2.94773700 3.50314500 2.28162100

C -3.34161100 5.50799000 3.56450400

H -4.37779800 5.62020700 3.88873200

H -2.67327800 5.66752500 4.42096200

H -3.11993100 6.25544900 2.79127700

H 0.71890500 0.77178700 -0.94163300

H -3.03861200 0.37161100 0.78777100

C 6.03228900 -0.36009700 -1.55215700

C 7.17525500 0.24421100 -2.08770300

C 5.67114800 -1.64208000 -1.98625400

C 7.94196100 -0.41345100 -3.05156500

H 7.47036500 1.23471900 -1.74714100

C 6.44229500 -2.30224800 -2.94329300

H 4.79610100 -2.11744200 -1.55394600

C 7.57698100 -1.69010500 -3.48182600

H 8.82805800 0.06759100 -3.45755600

H 6.15675900 -3.29910300 -3.26970500

H 8.17547300 -2.20720300 -4.22721700

H 5.76269700 1.14985600 -0.02728400

H 1.01621700 0.93363200 3.30843700

H 3.68397900 -2.00712300 2.06482800

H 0.69122400 -1.95400100 2.07360800

**TS14**

C 1.50699600 -1.69404200 5.16722800

H 1.19794800 -0.75489900 5.65030900

H 1.59743800 -2.44751100 5.95949200

C 2.83359700 -1.49048400 4.42104000

H 3.26517500 -2.47065200 4.18554400

H 3.56381500 -0.95676900 5.04523700

C 0.48238100 -2.09325700 4.09891200

H 0.73081900 -3.06718000 3.67690500

H -0.53132400 -2.15093400 4.52151800

C 0.27799800 -1.17299900 1.63132600

C 0.77563000 -2.09917900 0.71673700

C 0.53944400 -1.01701000 3.02496800

C 1.40025700 -3.39272700 0.94609600

O 1.48687000 -4.06259500 1.97083300

O 1.88525700 -3.86974200 -0.24772400

C 2.53866800 -5.13532400 -0.17456000

H 3.40336600 -5.09343800 0.49509000

H 1.85754200 -5.91252100 0.18756400

H 2.86234600 -5.35941500 -1.19288000

C 2.60260600 -0.73467700 3.11789500

H 2.48399900 0.34392600 3.21455300

C 3.26604600 -1.17173700 1.95977500

C 3.33133300 -0.37354700 0.78332600

O 2.76610200 0.71897200 0.59059800

O 4.13562900 -0.93276900 -0.18967600

C 4.21966600 -0.24696200 -1.43418800

H 3.42899000 -0.61559000 -2.10407100

C -2.59320500 -1.34273400 -0.41265000

C -2.45754200 -0.88906100 -1.72327400

C -2.39980700 -1.82447200 -2.80898400

C -2.52155800 -3.22219900 -2.52140500

C -2.72957900 -3.63600300 -1.18268300

C -2.77405000 -2.73817200 -0.13521800

H -2.08262900 -0.37596600 -4.39287400

C -2.19180300 -1.42966700 -4.16085500

C -2.44921600 -4.16154800 -3.58566300

H -2.87056000 -4.69446400 -0.97942400

C -2.25249000 -3.74554000 -4.88226500

C -2.11878100 -2.36546800 -5.16885900

H -2.54718600 -5.21897800 -3.35246500

H -2.19411000 -4.47267800 -5.68745900

H -1.95442500 -2.04267700 -6.19330700

C -2.36937300 0.58633500 -1.99747400

C -3.48634900 1.25007600 -2.60741900

C -1.23254600 1.30579200 -1.62623700

C -4.70028600 0.58280500 -2.93787400

C -3.39593100 2.65470600 -2.86748800

C -1.13034900 2.70473500 -1.93507900

C -5.75325600 1.26600100 -3.50513700

H -4.79408600 -0.47760200 -2.73310900

C -4.49685500 3.32926300 -3.46230800

C -2.20175500 3.33734000 -2.53663100

C -5.65238600 2.65211700 -3.77618400

H -6.67109500 0.73673700 -3.74623500

H -4.40792100 4.39471800 -3.66050200

H -2.12022200 4.39235100 -2.78558000

H -6.48968600 3.17709000 -4.22768800

C 0.10317800 3.50851100 -1.68795900

C 1.33107100 3.15015700 -2.26871700

C 0.03929900 4.69343200 -0.93767500

C 2.46079200 3.95128100 -2.10305200

H 1.39475100 2.24935800 -2.87321900

C 1.16825000 5.49688600 -0.77248700

H -0.90043500 4.97505000 -0.47095200

C 2.38275200 5.12906500 -1.35582500

H 3.39966800 3.65980700 -2.56585100

H 1.09941700 6.40704700 -0.18284400

H 3.26171300 5.75573500 -1.23104700

C -3.07164000 -3.24399600 1.24001700

C -4.28077300 -2.90152300 1.86978500

C -2.18634200 -4.10421500 1.90632500

C -4.59670300 -3.40561800 3.13169300

H -4.98388900 -2.25024000 1.35569700

C -2.50373000 -4.60748100 3.16973000

H -1.23384600 -4.35833500 1.45372800

C -3.70771900 -4.26196700 3.78557300

H -5.53977600 -3.13522600 3.59965900

H -1.79885300 -5.26287200 3.67349100

H -3.95155500 -4.65475800 4.76906700

C -0.14533900 0.59611200 -0.84727300

H 0.12104000 -0.34741500 -1.32949000

C -2.45252500 -0.34407000 0.71416000

H -2.76305400 -0.76067600 1.67395400

P -0.67467100 0.17392500 0.88378400

C -0.65531600 1.70448700 1.87356600

C -1.81908300 2.16807400 2.48626200

C 0.56359800 2.39629800 1.99786000

C -1.77366200 3.35596500 3.23792200

H -2.76669200 1.64808600 2.42188900

C 0.58787900 3.57329500 2.74984200

H 1.45958300 1.99622300 1.53553700

C -0.57913200 4.05924100 3.36734300

H -0.50550400 4.97592000 3.93957600

O -2.95626600 3.73293500 3.80050100

O 1.69938300 4.32592800 2.94806500

C 2.92851200 3.87222500 2.37899300

H 2.87041500 3.83184800 1.28630700

H 3.67779000 4.60479500 2.68434600

H 3.20254600 2.88153200 2.75819400

C -2.97393700 4.90239800 4.60517300

H -3.99904400 5.00098000 4.96641500

H -2.29286100 4.81120300 5.46114200

H -2.70564100 5.79481000 4.02454900

H 0.77688700 1.16866300 -0.73935500

H -3.03656700 0.56014000 0.51984100

C 5.57772100 -0.46663000 -2.06222300

C 6.71260600 -0.71852200 -1.28281200

C 5.72152600 -0.36738800 -3.45185200

C 7.96517300 -0.86200800 -1.88227900

H 6.60199000 -0.81487900 -0.20776100

C 6.97383400 -0.50386100 -4.05222400

H 4.84442500 -0.18632200 -4.07068900

C 8.10208400 -0.75205700 -3.26759500

H 8.83681900 -1.06257400 -1.26414600

H 7.06646100 -0.42584200 -5.13264400

H 9.07793200 -0.86538200 -3.73265100

H 4.03061900 0.81982000 -1.26673300

H 0.17064200 -0.07323500 3.42939300

H 0.65365000 -1.90786700 -0.34158800

H 3.74818700 -2.14235900 1.93520500

**CP14**

C 1.02786800 -3.28675600 4.56280400

H 1.13382800 -2.51350000 5.33796800

H 0.82002000 -4.23377900 5.07446300

C 2.29192300 -3.34656900 3.69179000

H 2.29367900 -4.28629400 3.12785700

H 3.21771400 -3.30980100 4.27810000

C -0.07192000 -2.89608500 3.56648600

H -0.32310400 -3.74762400 2.93423400

H -0.98797600 -2.54144200 4.05423300

C 0.09590900 -1.50439500 1.33589700

C 0.39036900 -2.30740500 0.25495300

C 0.58045100 -1.77653900 2.71738800

C 0.68323300 -3.75007900 0.21359000

O 0.45573700 -4.61061000 1.05408100

O 1.16423300 -4.06715900 -1.02433300

C 1.49687000 -5.44303800 -1.21508000

H 2.28967700 -5.75045500 -0.52606300

H 0.62513400 -6.08470700 -1.05376600

H 1.84421100 -5.52103200 -2.24656900

C 2.19492500 -2.16308300 2.69830000

H 2.65321000 -1.26467100 3.13345400

C 2.81021200 -2.43075000 1.38121800

C 3.17208300 -1.37012900 0.53244900

O 2.87091800 -0.16301000 0.68241100

O 3.90898700 -1.77485500 -0.57474600

C 4.21728600 -0.77667300 -1.53705500

H 3.40548900 -0.71373800 -2.27833700

C -2.74302700 -0.57622600 -0.54810400

C -2.48578600 0.09298100 -1.74406400

C -2.63291700 -0.58596000 -2.99859400

C -3.08530200 -1.94486000 -2.99836600

C -3.40477000 -2.56506600 -1.76573600

C -3.25213200 -1.91735200 -0.55568500

H -1.95685200 1.04225600 -4.26361200

C -2.31590700 0.01902000 -4.24781200

C -3.22131100 -2.63464800 -4.23382300

H -3.79714600 -3.57874300 -1.77772800

C -2.91174800 -2.01805300 -5.42410600

C -2.45024400 -0.67945300 -5.42727700

H -3.57056400 -3.66418200 -4.21809600

H -3.01484900 -2.55613900 -6.36233700

H -2.19767100 -0.20032500 -6.36924300

C -2.05335200 1.53244200 -1.71347100

C -2.97570000 2.54757400 -2.13644600

C -0.78849500 1.87751200 -1.23599600

C -4.30370000 2.26163800 -2.56390400

C -2.55897500 3.91658500 -2.09866300

C -0.36401200 3.24940700 -1.23884400

C -5.15874400 3.27170600 -2.94602900

H -4.64358200 1.23240200 -2.58304300

C -3.46234200 4.93515800 -2.50766700

C -1.24851300 4.22299400 -1.66193500

C -4.73559200 4.62273800 -2.92412900

H -6.16855800 3.03001700 -3.26624000

H -3.12659700 5.96904200 -2.48037700

H -0.92408900 5.26037600 -1.67858100

H -5.41971100 5.40866700 -3.23204100

C 1.01047400 3.67856800 -0.84324300

C 2.14426500 3.21101500 -1.52812800

C 1.18896200 4.62521700 0.17770300

C 3.41857900 3.67315000 -1.19876400

H 2.02414200 2.49484100 -2.33668200

C 2.46310200 5.08868100 0.50800000

H 0.32181300 4.98524100 0.72428800

C 3.58215300 4.61491400 -0.17991500

H 4.28255700 3.30285700 -1.74384300

H 2.58104000 5.81547900 1.30712300

H 4.57452400 4.97771200 0.07389200

C -3.68720900 -2.61217500 0.69424100

C -4.76622700 -2.10910700 1.44183900

C -3.07420300 -3.80136400 1.11621600

C -5.21894200 -2.77574500 2.58047000

H -5.26335400 -1.19904400 1.11429800

C -3.52853500 -4.46761900 2.25627600

H -2.21921200 -4.19281600 0.57497900

C -4.60078600 -3.95922600 2.99086200

H -6.05903700 -2.37469200 3.14157100

H -3.03162600 -5.38030500 2.57291800

H -4.95127500 -4.47902800 3.87847800

C 0.09559700 0.79018200 -0.66774600

H 0.14421300 -0.06215600 -1.35025700

C -2.38118400 0.10584200 0.75160900

H -2.79870200 -0.40915700 1.61832700

P -0.52840900 0.12914200 0.95583100

C -0.15722500 1.34844400 2.25777100

C -1.18254900 1.95636400 2.98175100

C 1.19371400 1.66111300 2.49618400

C -0.85735500 2.90700100 3.96604600

H -2.22957000 1.72550200 2.83062900

C 1.49510200 2.60754700 3.47778600

H 1.96889900 1.14770500 1.93669300

C 0.47287200 3.23603300 4.21239300

H 0.76049000 3.96112300 4.96380800

O -1.92138900 3.44851600 4.62405300

O 2.75621800 2.99003100 3.80073400

C 3.84163800 2.36495900 3.11337400

H 3.80203900 2.56880400 2.03815800

H 4.74624800 2.79989800 3.54203400

H 3.84057600 1.28036000 3.26867200

C -1.66611900 4.40349200 5.64273000

H -2.64357900 4.69272500 6.03266600

H -1.06277100 3.97520300 6.45360700

H -1.15658900 5.29064600 5.24417800

H 1.12336300 1.09839600 -0.47277700

H -2.72382700 1.14450500 0.76336100

C 5.52247600 -1.10142100 -2.22894800

C 6.53152100 -1.82514900 -1.58271200

C 5.75727300 -0.62904200 -3.52634200

C 7.75063000 -2.06398400 -2.21938800

H 6.34504400 -2.20967400 -0.58535100

C 6.97775500 -0.86107000 -4.16230800

H 4.97531200 -0.07807600 -4.04613500

C 7.98068300 -1.58052400 -3.50926200

H 8.52286100 -2.63192700 -1.70621200

H 7.14138600 -0.48841000 -5.17052100

H 8.93005300 -1.76864400 -4.00405500

H 4.26910900 0.19840900 -1.03782500

H 0.50018200 -0.86049000 3.30943600

H 0.29954800 -1.90880400 -0.75050200

H 3.17387900 -3.42364500 1.14285300

**TS15**

C -1.46315200 -3.47719100 -4.21400900

H -1.49778800 -2.76858300 -5.05457800

H -1.39409700 -4.48470500 -4.64055800

C -2.69541300 -3.29625600 -3.31382300

H -2.79556900 -4.17383400 -2.66464400

H -3.62876900 -3.19509000 -3.88018000

C -0.29124300 -3.14351100 -3.28112400

H -0.12487100 -3.96296600 -2.58197400

H 0.64523100 -2.95068300 -3.81830200

C -0.24693300 -1.56078600 -1.15888900

C -0.74531100 -2.19732800 -0.02785800

C -0.77035500 -1.88175700 -2.51990900

C -1.11669000 -3.61159900 0.15342300

O -0.94271600 -4.55992000 -0.60013300

O -1.64311000 -3.77825600 1.40192000

C -2.06955200 -5.10578200 1.71275600

H -2.85629000 -5.43334000 1.02610300

H -1.23567200 -5.81183800 1.65052100

H -2.45498800 -5.05914700 2.73254500

C -2.41145800 -2.04920500 -2.44084700

H -2.77506100 -1.14373000 -2.94376700

C -3.00028400 -2.11412900 -1.08161700

C -3.28823800 -0.92560800 -0.36803700

O -2.88151800 0.21887800 -0.64966700

O -4.07268600 -1.14354300 0.75490200

C -4.33243600 -0.02311400 1.59352100

H -3.63608800 -0.04351000 2.44489300

C 2.73191900 -0.91283900 0.64700100

C 2.58858900 -0.11826500 1.78381800

C 2.65242300 -0.70592100 3.09029300

C 2.90327500 -2.11166700 3.20232900

C 3.11637300 -2.86990400 2.02508300

C 3.04454000 -2.30788900 0.76586800

H 2.23366900 1.10081100 4.21663400

C 2.44063800 0.03843700 4.28535500

C 2.95186100 -2.71057100 4.49053000

H 3.36095600 -3.92477500 2.12069400

C 2.74777400 -1.96071600 5.62584500

C 2.48538300 -0.57344000 5.51852000

H 3.14898600 -3.77760000 4.56017500

H 2.78247400 -2.42988000 6.60522300

H 2.31555100 0.01259300 6.41763800

C 2.36451500 1.36022000 1.63356700

C 3.42720300 2.26582300 1.96570800

C 1.15419300 1.83994100 1.13201600

C 4.70834500 1.83046700 2.40969700

C 3.20769000 3.67214100 1.81319600

C 0.92605000 3.25371000 1.02442200

C 5.70415900 2.73689400 2.69973900

H 4.89981800 0.76850900 2.51383400

C 4.25280000 4.58232200 2.12943100

C 1.94564700 4.12382000 1.35996900

C 5.47617900 4.12776400 2.56444500

H 6.67501900 2.38152500 3.03421000

H 4.06616600 5.64755100 2.01590800

H 1.76949200 5.19448000 1.29388400

H 6.26981400 4.83127700 2.80055800

C -0.38440400 3.83835300 0.61173300

C -1.55740900 3.57531300 1.33877600

C -0.45370400 4.73259300 -0.46795800

C -2.76365700 4.18502600 0.99399400

H -1.51841300 2.90461400 2.19301000

C -1.65952800 5.34447400 -0.81322700

H 0.44319800 4.93567900 -1.04647400

C -2.81854600 5.07316100 -0.08297400

H -3.65872600 3.97277000 1.57241200

H -1.69381200 6.02826500 -1.65704200

H -3.75740800 5.55125600 -0.34905300

C 3.36757200 -3.15526800 -0.42240800

C 4.50930800 -2.87771600 -1.19436800

C 2.58295900 -4.26812800 -0.75890300

C 4.85621600 -3.68982200 -2.27410600

H 5.13728600 -2.02929600 -0.93252100

C 2.93167200 -5.08006700 -1.84037000

H 1.67899500 -4.48440300 -0.19938400

C 4.06730300 -4.79552500 -2.59994600

H 5.74645100 -3.46312700 -2.85514100

H 2.30334300 -5.92990700 -2.09186100

H 4.33523300 -5.42853100 -3.44168100

C 0.12041900 0.84421500 0.65628100

H -0.02901300 0.06507100 1.40800200

C 2.45370000 -0.29532800 -0.70370900

H 2.78403800 -0.93356200 -1.52473400

P 0.61782200 -0.03147900 -0.91123400

C 0.41820200 1.13069500 -2.30322600

C 1.51006500 1.52623200 -3.07467600

C -0.87644300 1.61593500 -2.56455600

C 1.31246000 2.43430700 -4.13072200

H 2.51478400 1.15899900 -2.90719100

C -1.05164400 2.51859800 -3.61561700

H -1.71137500 1.26447200 -1.96758700

C 0.04074900 2.93302500 -4.39960600

H -0.14915300 3.63238200 -5.20450000

O 2.43476000 2.76284300 -4.83164000

O -2.24849800 3.05511400 -3.96463000

C -3.40243900 2.65407300 -3.22460300

H -3.31471800 2.93334800 -2.16942700

H -4.24003000 3.18385000 -3.68189700

H -3.56462000 1.57268300 -3.29237700

C 2.30625800 3.66066300 -5.92335600

H 3.31008400 3.77424900 -6.33644100

H 1.63743400 3.26078600 -6.69659700

H 1.93438900 4.64090000 -5.59710200

H -0.85653300 1.28065500 0.44438500

H 2.93773500 0.68069500 -0.80123900

C -5.75958000 -0.05115700 2.09430200

C -6.78676100 -0.62926000 1.33937500

C -6.07925600 0.55475100 3.31583000

C -8.10581500 -0.59476600 1.79462700

H -6.54020600 -1.11816000 0.40244700

C -7.39829500 0.59585200 3.76995100

H -5.28763800 0.99431700 3.92019100

C -8.41792000 0.02026800 3.00909400

H -8.89217700 -1.05232900 1.19930400

H -7.62765300 1.06848900 4.72181300

H -9.44535100 0.04487300 3.36292100

H -4.13119500 0.89421800 1.02818200

H -0.57766800 -1.03463100 -3.18577900

H -0.67217800 -1.70615000 0.93775300

H -3.49755900 -3.02098000 -0.75582900

**CP15**

C 1.90049600 -4.01029500 3.69732300

H 2.31344300 -3.42050800 4.52810900

H 1.66698400 -5.00764700 4.08832600

C 2.89277200 -4.04697700 2.52021700

H 2.60169000 -4.85003900 1.83416800

H 3.92674100 -4.23376800 2.83351700

C 0.68736400 -3.29226000 3.08849100

H 0.16928700 -3.98091200 2.41233800

H -0.03623200 -2.94138200 3.83428000

C 0.64766400 -1.64820400 0.99769500

C 1.25012300 -2.39104900 -0.18650300

C 1.31327100 -2.12084600 2.27815900

C 0.50366600 -3.67965900 -0.51815100

O 0.72266700 -4.78615600 -0.06448100

O -0.50024100 -3.43061600 -1.39291200

C -1.32647900 -4.55785900 -1.72553000

H -0.72019900 -5.36600400 -2.14361600

H -1.84775300 -4.92439600 -0.83719700

H -2.04135300 -4.18801100 -2.46059300

C 2.72039300 -2.67315400 1.82524400

H 3.49995800 -1.98337800 2.15973200

C 2.72140500 -2.70576400 0.27709000

C 3.62894700 -1.65825400 -0.33209200

O 3.99447200 -0.62236300 0.19365800

O 3.97542300 -1.98682800 -1.60035300

C 4.76865600 -1.00967500 -2.30932900

H 4.29013800 -0.02973000 -2.19514600

C -2.90645300 -0.64764800 -0.17367400

C -2.91560100 0.17339000 -1.30382000

C -3.53049800 -0.26151600 -2.52539800

C -4.22367000 -1.51256100 -2.54089100

C -4.28625700 -2.27026000 -1.34779200

C -3.64020400 -1.88471900 -0.18754100

H -2.92585300 1.42402000 -3.75117700

C -3.46272300 0.48192500 -3.73794400

C -4.83433500 -1.96166800 -3.74369900

H -4.84589800 -3.20237400 -1.35418400

C -4.75472800 -1.21502600 -4.89673000

C -4.05583000 0.01636000 -4.89094200

H -5.36349300 -2.91190100 -3.73468000

H -5.22148600 -1.56826800 -5.81220400

H -3.98455700 0.59820800 -5.80600900

C -2.30792000 1.54369500 -1.24473700

C -3.17076200 2.69212300 -1.29498200

C -0.92819300 1.70114700 -1.11696800

C -4.59125200 2.59619200 -1.32034200

C -2.58616600 3.99819700 -1.27537600

C -0.34687200 3.01487500 -1.16272200

C -5.37985000 3.72568300 -1.34320700

H -5.05527000 1.61671400 -1.31196300

C -3.42585600 5.14419000 -1.31123300

C -1.17645400 4.11625600 -1.23630300

C -4.79511000 5.01471700 -1.34549100

H -6.46188800 3.62621700 -1.35654600

H -2.96255400 6.12802600 -1.30480300

H -0.73612900 5.10898200 -1.28602500

H -5.42910900 5.89690600 -1.36777100

C 1.12681900 3.25384300 -1.20006000

C 1.91922100 2.73050000 -2.23634300

C 1.73775100 4.08519500 -0.24875700

C 3.28053900 3.02643800 -2.31550000

H 1.45759600 2.10925400 -2.99964500

C 3.09943100 4.38279800 -0.32705600

H 1.14123500 4.48560200 0.56576900

C 3.87602200 3.85413300 -1.36000100

H 3.87189600 2.62363900 -3.13390600

H 3.55307800 5.02244500 0.42523300

H 4.93529500 4.08814600 -1.42360600

C -3.82359800 -2.74624600 1.01836700

C -5.12728000 -3.06645800 1.43711300

C -2.74251900 -3.29645200 1.72525600

C -5.34441200 -3.91041700 2.52627500

H -5.97373500 -2.63640800 0.90833200

C -2.96075100 -4.14312200 2.81369400

H -1.72722900 -3.06511400 1.41578900

C -4.26027000 -4.45401200 3.21796900

H -6.36093100 -4.13918300 2.83607500

H -2.10859300 -4.56335000 3.34000700

H -4.42668800 -5.11305900 4.06594400

C -0.07426500 0.48383800 -0.86663400

H -0.34646300 -0.33276400 -1.54333000

C -2.16152500 -0.17224700 1.04637700

H -2.41329300 -0.75107500 1.93699900

P -0.26156600 -0.22673400 0.85556200

C 0.30467800 1.02779400 2.08546000

C -0.55103800 1.67292300 2.97547000

C 1.68698100 1.30168700 2.09748900

C -0.03286800 2.61434100 3.88383300

H -1.61429200 1.47581700 3.01715500

C 2.18690600 2.24330900 2.99934300

H 2.34765400 0.76632500 1.42559000

C 1.32752300 2.90665900 3.89440100

H 1.76412300 3.62238100 4.58003700

O -0.95089300 3.18426800 4.71672700

O 3.49664200 2.59500700 3.09979700

C 4.44417100 1.90982300 2.28206100

H 4.26157200 2.09188200 1.21787600

H 5.41813000 2.31497900 2.56407800

H 4.42457600 0.83011200 2.46249300

C -0.49624900 4.13044400 5.67041600

H -1.38206700 4.44620800 6.22463100

H 0.22838600 3.68633700 6.36568200

H -0.04040100 5.00448300 5.18641100

H 0.98949500 0.69034700 -0.98941400

H -2.41517200 0.87397600 1.23837500

C 4.87458800 -1.41700800 -3.75720000

C 3.75337600 -1.87293400 -4.46368100

C 6.09488700 -1.29332800 -4.43043800

C 3.85357400 -2.19628200 -5.81628900

H 2.80598500 -1.98846800 -3.94543000

C 6.19482800 -1.60722500 -5.78770000

H 6.97448000 -0.95148300 -3.88926400

C 5.07386900 -2.06044600 -6.48386900

H 2.97783200 -2.55504600 -6.35068300

H 7.15003600 -1.50660800 -6.29616800

H 5.15022500 -2.31166900 -7.53838300

H 5.75620200 -0.94163700 -1.84094300

H 1.45673900 -1.28585000 2.97543100

H 1.24344500 -1.79934400 -1.10793000

H 3.02818000 -3.67996900 -0.11324600

**TS16**

C -3.10522400 -0.63607800 0.49212700

C -3.56680400 0.42293500 -0.28904100

C -4.65076400 0.22641400 -1.20789300

C -5.30356500 -1.04698000 -1.23975000

C -4.88362300 -2.05646100 -0.34070800

C -3.80769100 -1.88941100 0.51176000

H -4.59730000 2.19837600 -2.11081300

C -5.09104200 1.23285900 -2.11309100

C -6.36818100 -1.26316000 -2.15667100

H -5.41900000 -3.00227700 -0.33503600

C -6.76978400 -0.26812800 -3.01765700

C -6.11996900 0.98983000 -2.99597300

H -6.85540100 -2.23512900 -2.16784500

H -7.58111600 -0.44509400 -3.71826200

H -6.43418100 1.76894500 -3.68503800

C -2.96869700 1.79467300 -0.13639800

C -3.75418700 2.81748000 0.49729800

C -1.67245100 2.06703500 -0.57383000

C -5.04665900 2.57978600 1.04615800

C -3.20367900 4.13321100 0.61754100

C -1.14695100 3.40242200 -0.49680600

C -5.75365600 3.58942700 1.66133200

H -5.47507400 1.58607500 0.98369000

C -3.96314500 5.15657700 1.24763300

C -1.91627200 4.38880600 0.09053200

C -5.21257000 4.89402500 1.75945700

H -6.73646700 3.38258100 2.07610400

H -3.53219900 6.15218900 1.32109300

H -1.52933900 5.40364100 0.13360900

H -5.78391200 5.68187800 2.24256700

C 0.17625000 3.79381300 -1.06465300

C 0.48667200 3.56814400 -2.41555800

C 1.11502300 4.46814500 -0.26682000

C 1.70422700 3.99017700 -2.94827700

H -0.23502900 3.07049500 -3.05741400

C 2.33203700 4.89226800 -0.79988300

H 0.89234700 4.64083000 0.78255800

C 2.63101000 4.65354700 -2.14242000

H 1.92664200 3.79959000 -3.99423900

H 3.05027200 5.40082700 -0.16273200

H 3.58122200 4.97872900 -2.55717200

C -3.51813300 -2.98042600 1.49487800

C -4.55135100 -3.39357500 2.35538700

C -2.28007900 -3.63909400 1.58075900

C -4.35519600 -4.42714400 3.27131200

H -5.51206000 -2.88778100 2.30894000

C -2.08508800 -4.67463200 2.49686200

H -1.47116500 -3.36168500 0.91413800

C -3.11915100 -5.07221500 3.34574700

H -5.16826500 -4.72543400 3.92809600

H -1.12223900 -5.17688900 2.53303100

H -2.96461200 -5.87954800 4.05656500

C -0.82251200 0.92297700 -1.08274700

H -1.37107900 0.32745800 -1.81711100

C -1.86236400 -0.41885700 1.32231800

H -1.69766800 -1.23483600 2.02764400

P -0.33660400 -0.25777100 0.26020300

C 0.90942000 0.51001100 1.34634800

C 0.79011900 0.38048100 2.74641100

C 1.99772400 1.15762700 0.77103400

C 1.79218500 0.91572400 3.55713500

H -0.05029300 -0.13748300 3.18648000

C 2.99729100 1.69175200 1.60624400

H 2.12242300 1.21506400 -0.30529000

C 2.89657700 1.57278900 2.99108500

H 3.65035700 1.96411800 3.66280600

O 1.79673400 0.85446800 4.91877300

O 4.02446600 2.30410500 0.96495900

C 5.15796000 2.70250400 1.72594400

H 5.87689300 3.09029700 1.00400900

H 4.89935200 3.48772300 2.44877800

H 5.60175100 1.84705200 2.24815300

C 0.73069700 0.17975700 5.56906600

H 0.94492900 0.24331900 6.63726600

H -0.23532000 0.66031400 5.36463000

H 0.68073300 -0.87571200 5.27123200

H 0.11005000 1.22494800 -1.56125600

H -1.92662400 0.51692200 1.88775800

C -1.07624500 -3.85782600 -3.36720900

H -1.30200800 -4.88006600 -3.69467300

H -1.88107700 -3.21225800 -3.75004800

C 0.28307900 -3.37719900 -3.89032100

C -0.99801700 -3.74278400 -1.84048600

H -1.97647600 -3.94591600 -1.38242700

H -0.27618200 -4.45608900 -1.44175700

C 0.19402700 -1.85868700 -0.41214100

C 1.40072300 -2.31879200 0.11131600

C -0.55864800 -2.31432500 -1.54104400

H -1.36688700 -1.63723600 -1.82445600

H 1.92445400 -1.69152200 0.82174600

C 2.06879700 -3.59452700 -0.09687000

O 1.68746700 -4.61129500 -0.66332100

O 3.30164300 -3.55771300 0.52040400

C 4.08492000 -4.74168700 0.37028100

H 3.57147100 -5.60991500 0.79530200

H 4.29320400 -4.94341400 -0.68494200

H 5.01605000 -4.54988700 0.90703100

C 0.73694800 -2.12909500 -3.13967600

H 0.28678700 -1.19248300 -3.46814100

C 2.08845700 -2.03456500 -2.76611200

H 2.72842100 -2.90876900 -2.78657400

C 2.68227200 -0.78455100 -2.43023200

O 2.09142900 0.29775200 -2.27665700

O 4.05544000 -0.88361600 -2.30123200

C 4.79729600 0.30717200 -2.05326400

H 4.11595400 1.10153200 -1.73229300

H 5.26007700 0.63197000 -2.99580900

C 5.86666800 0.06333500 -1.01087700

C 5.68500200 -0.86883000 0.01857500

C 7.05007200 0.81257600 -1.04378900

C 6.66420500 -1.03238500 1.00116900

H 4.78758700 -1.47903400 0.03785800

C 8.02805700 0.65126200 -0.06021500

H 7.21053600 1.52466400 -1.85141700

C 7.83668000 -0.27278700 0.96964600

H 6.51092700 -1.76256100 1.79235600

H 8.94349600 1.23637400 -0.10639100

H 8.59967200 -0.40888500 1.73209600

H 1.02573300 -4.16813100 -3.73289400

H 0.24877100 -3.17528400 -4.96980800

**CP16**

C -1.49989100 -0.05515900 2.82360700

C -2.21245500 1.13738700 2.71598100

C -3.60062200 1.18414300 3.07339900

C -4.21574700 0.00600900 3.60675500

C -3.42168100 -1.14709200 3.82159900

C -2.08977000 -1.20186300 3.45685400

H -3.95588600 3.23892300 2.47762200

C -4.40615800 2.34317700 2.89151700

C -5.59592100 0.03296400 3.94539000

H -3.87202400 -2.00441100 4.31545800

C -6.34684000 1.17006500 3.75479500

C -5.74413900 2.33388400 3.21896800

H -6.04867500 -0.86701700 4.35445500

H -7.40258900 1.17772700 4.01128300

H -6.34363400 3.22663900 3.06353700

C -1.50597200 2.39440400 2.29039100

C -1.24221600 3.39111700 3.29206500

C -1.07157800 2.57393900 0.97735100

C -1.58264000 3.22719800 4.66547400

C -0.57358200 4.59544000 2.90511500

C -0.44723700 3.80860000 0.58731000

C -1.29181000 4.20513700 5.59108600

H -2.07196200 2.31510900 4.98679200

C -0.29686500 5.59060300 3.88184400

C -0.21510400 4.77367000 1.54897500

C -0.64780900 5.40311100 5.19845900

H -1.55828200 4.05491000 6.63375900

H 0.20390100 6.50271500 3.56614100

H 0.23910300 5.71547100 1.25225300

H -0.42883300 6.16769500 5.93873200

C -0.08280500 4.14182900 -0.82124000

C -1.04102000 4.12048500 -1.84837900

C 1.21375000 4.58688700 -1.12678200

C -0.71233800 4.53013300 -3.14027700

H -2.05606800 3.80068200 -1.62981000

C 1.54167100 5.00323200 -2.41715500

H 1.96892900 4.59611000 -0.34579500

C 0.57878100 4.97728600 -3.42861800

H -1.46858800 4.50527400 -3.91974600

H 2.55177200 5.34089500 -2.63323400

H 0.83297500 5.30058600 -4.43442600

C -1.29160100 -2.40938900 3.82671600

C -0.13824800 -2.28023300 4.62188900

C -1.70760600 -3.70034600 3.46543200

C 0.58167900 -3.40295300 5.02929700

H 0.18088900 -1.29165900 4.94260400

C -0.98633900 -4.82405400 3.87190100

H -2.58958200 -3.82293900 2.84587700

C 0.16134000 -4.68042600 4.65258800

H 1.46624500 -3.27923900 5.64853100

H -1.32049700 -5.81272200 3.56934100

H 0.72302100 -5.55588000 4.96682300

C -1.24006300 1.44268000 -0.01334400

H -2.26707900 1.06827400 -0.00358200

C -0.11774900 -0.12349200 2.21666500

H 0.38865900 -1.05599000 2.46847000

P -0.16114900 -0.02174600 0.35223200

C 1.57453800 0.32324100 -0.11395500

C 2.59017400 0.14798200 0.83008000

C 1.86008400 0.75928900 -1.42042500

C 3.92005800 0.42250800 0.47038500

H 2.40879800 -0.20183100 1.83770700

C 3.19176800 1.02516700 -1.75903400

H 1.05449500 0.83470100 -2.14522000

C 4.22189700 0.86434400 -0.81375300

H 5.23462800 1.08401100 -1.12872900

O 4.84192200 0.22312800 1.45673200

O 3.60128600 1.44247400 -2.97934100

C 2.61744500 1.59640800 -4.01095200

H 1.91801300 2.40171300 -3.76170700

H 3.18178900 1.86099800 -4.90698700

H 2.06112200 0.67072400 -4.17833200

C 6.20703600 0.46712700 1.15661100

H 6.75931200 0.24110500 2.07060700

H 6.56363300 -0.18191700 0.34610600

H 6.37754700 1.51569700 0.87890900

H -1.01711600 1.70919100 -1.04542700

H 0.50669500 0.70972600 2.55464400

C -4.27962900 -3.06703100 -0.78835400

H -4.78309200 -4.01857100 -0.58054400

H -5.02259800 -2.26809100 -0.64854500

C -3.68625600 -2.99884600 -2.20130200

H -4.44720100 -2.84536200 -2.97591600

H -3.16818800 -3.93857100 -2.42119600

C -3.06797200 -2.83046600 0.12502000

H -3.36004100 -2.53898100 1.13924800

H -2.45705400 -3.73176100 0.17582600

C -0.80984000 -1.55755100 -0.34883500

C 0.14096800 -2.39537300 -0.86989100

C -2.28247600 -1.68627900 -0.57089000

H -2.77212700 -0.76245200 -0.25368700

H 1.17849100 -2.07543900 -0.88174200

C -0.01651400 -3.77225200 -1.36887300

O -0.93018400 -4.56377500 -1.20021000

O 1.13709400 -4.14468300 -2.01160100

C 1.15333800 -5.49377300 -2.48590300

H 2.13680800 -5.63520500 -2.93733700

H 1.00467500 -6.19840200 -1.66238100

H 0.36738500 -5.65703400 -3.22981300

C -2.66051000 -1.84473700 -2.17559000

H -3.16450700 -0.89090200 -2.37808300

C -1.53863000 -1.99588900 -3.12841100

H -1.44684900 -2.91201800 -3.69650600

C -0.72841100 -0.88223100 -3.35204300

O -0.81736000 0.18921700 -2.69815400

O 0.29399300 -0.89619900 -4.30213000

C 0.51406100 -2.09422300 -5.05075100

H -0.37970700 -2.33476600 -5.64275400

H 0.70264800 -2.92603100 -4.36151900

C 1.70489900 -1.88378100 -5.95308300

C 3.00288900 -2.08241500 -5.46382900

C 1.54050200 -1.46327100 -7.27801700

C 4.11293600 -1.86547000 -6.28087300

H 3.13678400 -2.40779700 -4.43468200

C 2.64794100 -1.24818200 -8.10065500

H 0.53685800 -1.30407600 -7.66575700

C 3.93717200 -1.44853800 -7.60291200

H 5.11404800 -2.02455300 -5.88834200

H 2.50467200 -0.92562800 -9.12883300

H 4.80061800 -1.28345300 -8.24232900

**TS17**

C -1.47805600 -0.03520200 2.85250200

C -2.19302000 1.15760900 2.76673900

C -3.57760700 1.19977100 3.13905800

C -4.18906100 0.01292200 3.65714000

C -3.39562300 -1.14656100 3.83856500

C -2.06717200 -1.19471100 3.46178300

H -3.93759500 3.26463500 2.58227100

C -4.38432100 2.36216500 2.98522100

C -5.56560200 0.03539800 4.01053900

H -3.84409700 -2.01541400 4.31376500

C -6.31728500 1.17646700 3.84859700

C -5.71876700 2.34874800 3.32681500

H -6.01507900 -0.87147100 4.40799700

H -7.37022400 1.18066400 4.11665300

H -6.31862100 3.24490700 3.19356000

C -1.49394600 2.41830100 2.34138800

C -1.21773300 3.40779100 3.34646000

C -1.08045500 2.60805300 1.02251200

C -1.53312200 3.22953900 4.72402100

C -0.56249700 4.61903700 2.95914200

C -0.47483300 3.85246200 0.63240800

C -1.23097600 4.20046200 5.65343600

H -2.01148400 2.31149000 5.04492900

C -0.27382700 5.60691700 3.93972500

C -0.23201600 4.81135900 1.59796000

C -0.60033700 5.40549900 5.26064900

H -1.47795100 4.03915500 6.69928000

H 0.21626400 6.52472600 3.62360400

H 0.20572100 5.76071800 1.30054000

H -0.37226900 6.16446600 6.00399100

C -0.15026800 4.20998900 -0.77989600

C -1.13064000 4.17637200 -1.78596100

C 1.12409700 4.70176500 -1.10765400

C -0.84579700 4.62041200 -3.07707000

H -2.13034700 3.82256200 -1.55001100

C 1.40742100 5.15440300 -2.39641700

H 1.89662900 4.72209700 -0.34403700

C 0.42242100 5.11647300 -3.38609800

H -1.61933100 4.58600100 -3.83906900

H 2.40023200 5.53096500 -2.62824700

H 0.64177300 5.46819200 -4.39058100

C -1.27016500 -2.41523400 3.78936600

C -0.13203100 -2.31864000 4.61000900

C -1.67190600 -3.68773200 3.35549800

C 0.58654200 -3.45666900 4.97539800

H 0.17693400 -1.34445300 4.98099900

C -0.95157900 -4.82659500 3.71955200

H -2.53916800 -3.78159000 2.71076900

C 0.17973600 -4.71605600 4.52898800

H 1.45954900 -3.35950900 5.61553500

H -1.27293500 -5.80046500 3.36045600

H 0.74047600 -5.60331100 4.81040500

C -1.25527300 1.47937800 0.03055000

H -2.28620000 1.11512800 0.04421400

C -0.10423800 -0.09556100 2.23066500

H 0.40953600 -1.02500000 2.47777400

P -0.19749400 -0.01747700 0.36409100

C 1.53134100 0.31845000 -0.14401100

C 2.57471900 0.06600300 0.74865800

C 1.78658500 0.80804100 -1.43869800

C 3.90039000 0.30905200 0.35075300

H 2.41514600 -0.32146900 1.74644300

C 3.11332700 1.03542900 -1.82035300

H 0.96725200 0.94993500 -2.13506300

C 4.17164500 0.79353800 -0.92481800

H 5.17950500 0.99006500 -1.26923400

O 4.84945800 0.03678000 1.29227100

O 3.49404000 1.48628900 -3.04025800

C 2.47813500 1.78698200 -4.00586600

H 1.83579500 2.60037100 -3.65259700

H 3.01921700 2.10640100 -4.89850900

H 1.86748800 0.90979400 -4.23543200

C 6.20996900 0.25713700 0.95560100

H 6.78593600 -0.02069700 1.84020400

H 6.52193600 -0.36702400 0.10791600

H 6.39945800 1.31151800 0.71460200

H -1.03774400 1.75538000 -1.00117600

H 0.52136000 0.74351800 2.55195400

C -4.32127000 -3.00799600 -0.90497100

H -4.84406500 -3.95168600 -0.70982700

H -5.06907600 -2.20338300 -0.85138800

C -3.61679400 -2.98604100 -2.26964800

H -4.31168400 -2.86059500 -3.10859300

H -3.08652400 -3.93415800 -2.41473000

C -3.17999000 -2.74318400 0.08740600

H -3.54081300 -2.42760800 1.07233600

H -2.57320900 -3.64131800 0.20388900

C -0.86604800 -1.49855900 -0.33702000

C 0.01185100 -2.28593300 -1.09138700

C -2.34295600 -1.61968200 -0.58168000

H -2.83944700 -0.68367200 -0.30647900

H 1.02556100 -1.92523100 -1.24824900

C -0.06263300 -3.74445500 -1.31709200

O -0.91256600 -4.54241900 -0.96099600

O 1.05674700 -4.15671800 -1.99368100

C 1.12055200 -5.56089000 -2.26072000

H 2.06695600 -5.71853700 -2.78061500

H 1.09488000 -6.13503600 -1.33007900

H 0.28311900 -5.87907500 -2.88934500

C -2.59422300 -1.82926600 -2.18728000

H -3.06168800 -0.88919000 -2.50199200

C -1.35917400 -2.03236400 -2.99676600

H -1.26008200 -2.95833900 -3.55073500

C -0.66289500 -0.86438100 -3.42306700

O -0.85495000 0.26151400 -2.93646500

O 0.34386900 -0.93583900 -4.37221800

C 0.66853600 -2.19458500 -4.98078400

H -0.19637400 -2.57457700 -5.54014300

H 0.92004100 -2.92044300 -4.19908200

C 1.84084900 -1.98814900 -5.90876100

C 3.10370300 -1.66873600 -5.38936700

C 1.69286900 -2.11081300 -7.29410200

C 4.19183400 -1.47338500 -6.23885400

H 3.22831100 -1.56512400 -4.31445000

C 2.78260200 -1.92369900 -8.14831100

H 0.71647600 -2.35285100 -7.70783900

C 4.03434800 -1.60329900 -7.62205400

H 5.16396900 -1.22291400 -5.82220700

H 2.65137300 -2.02435900 -9.22261700

H 4.88350100 -1.45513300 -8.28421800

**CP17**

C 2.02130800 1.53432400 -0.34630000

C 2.60983200 0.59171800 -1.19185400

C 2.78758100 0.87018500 -2.58728800

C 2.38917100 2.14941900 -3.09181800

C 1.87276300 3.11102200 -2.19017800

C 1.69303200 2.84174600 -0.84693800

H 3.59471700 -1.06428000 -3.14768200

C 3.30713500 -0.08294300 -3.50859700

C 2.54317800 2.43438800 -4.47562600

H 1.63541900 4.10273400 -2.56705300

C 3.05603400 1.49243800 -5.33760800

C 3.43607200 0.22018600 -4.84616100

H 2.24208400 3.41343600 -4.84090800

H 3.16511300 1.71875600 -6.39476100

H 3.82980600 -0.52520200 -5.53198600

C 3.05935300 -0.72679200 -0.63133200

C 4.46413100 -0.99119500 -0.49402100

C 2.11841500 -1.66206700 -0.20406400

C 5.46968400 -0.04528000 -0.84328900

C 4.88170500 -2.24753400 0.05067800

C 2.54650200 -2.93378900 0.30826700

C 6.80626700 -0.33612700 -0.67963600

H 5.17274600 0.91921600 -1.23917700

C 6.26868300 -2.52089200 0.19914500

C 3.89776900 -3.19368200 0.42508500

C 7.21412400 -1.58783200 -0.15887300

H 7.55521000 0.40296600 -0.95115600

H 6.56755500 -3.48376300 0.60695700

H 4.22180100 -4.16116100 0.80084200

H 8.27196000 -1.80539700 -0.03885000

C 1.58864000 -4.01405200 0.69353800

C 0.71466700 -4.58117400 -0.25003300

C 1.58886700 -4.53055800 1.99838500

C -0.13618300 -5.62863600 0.10392600

H 0.71517100 -4.20758600 -1.27064500

C 0.73865700 -5.57938100 2.35352700

H 2.24913200 -4.09175200 2.74101000

C -0.12709700 -6.13220500 1.40757400

H -0.79734900 -6.06003200 -0.64326800

H 0.74935400 -5.95951300 3.37151600

H -0.78505000 -6.95287400 1.68135400

C 1.23535800 3.95397100 0.03858200

C 2.02034200 4.37270200 1.12759300

C 0.05669200 4.66123100 -0.24628400

C 1.63386600 5.45843000 1.91323400

H 2.95173000 3.85549600 1.34409500

C -0.32984800 5.74743300 0.54223200

H -0.57761600 4.34999100 -1.07039200

C 0.45489600 6.14978900 1.62391900

H 2.25940100 5.76985500 2.74596600

H -1.25261400 6.27131200 0.30797300

H 0.15255400 6.99514700 2.23644000

C 0.65485700 -1.29690000 -0.23810600

H 0.36929100 -0.87578800 -1.20719400

C 1.66010700 1.11863600 1.05547800

H 1.39835000 1.96855900 1.68755400

P 0.13778100 -0.04002100 1.03575900

C 0.18601000 -0.81654000 2.70467900

C 1.14723200 -0.50797700 3.66416300

C -0.80568600 -1.77879100 2.97483000

C 1.12397500 -1.16025600 4.91094700

H 1.91788400 0.23454700 3.50278300

C -0.80733900 -2.43190100 4.20724300

H -1.55865100 -1.97910500 2.22343800

C 0.15664900 -2.12371200 5.18318800

H 0.10902100 -2.65057800 6.12818000

O 2.09654500 -0.77624200 5.78660600

O -1.70604200 -3.38878700 4.57289900

C -2.70437900 -3.76514500 3.63313400

H -2.25697300 -4.18422000 2.72333400

H -3.30462200 -4.53009200 4.12967400

H -3.34620900 -2.91567100 3.36736100

C 2.12101400 -1.37910900 7.07040600

H 2.95914000 -0.92235400 7.59984300

H 1.19243900 -1.18625100 7.62365300

H 2.28138100 -2.46353200 7.00418800

H 0.01310100 -2.16177200 -0.07405100

H 2.48712200 0.57842500 1.52536300

C -4.31737500 1.91918700 2.77119500

H -5.34792000 1.64992600 3.03015100

H -3.89852500 2.44709000 3.63834100

C -4.23334800 2.82885800 1.51398600

H -4.23400400 3.89040900 1.78295100

H -5.10553600 2.67308600 0.86653800

C -3.43464200 0.70562800 2.43600100

H -3.11482100 0.14600400 3.32193300

H -3.96479000 0.00553500 1.77724900

C -1.44724200 0.46397300 0.70032200

C -1.97069800 0.67220100 -0.72830200

C -2.24078600 1.33329400 1.66962100

H -1.60030000 1.82427800 2.41695200

H -1.21905300 1.07362700 -1.42084900

C -2.43709700 -0.61978900 -1.39077600

O -1.85379900 -1.17149400 -2.30806200

O -3.55815500 -1.13475000 -0.83713600

C -4.02137900 -2.36303800 -1.41262700

H -4.93455900 -2.61480800 -0.87148900

H -4.23020100 -2.23503200 -2.47821400

H -3.27361500 -3.15226700 -1.29042800

C -2.92528600 2.41655400 0.78466200

H -2.26017200 3.26869200 0.62522800

C -3.14739400 1.70013200 -0.55954500

H -4.08059500 1.13144900 -0.52504900

C -3.21636000 2.60655200 -1.77030900

O -2.83641400 3.75904000 -1.84057100

O -3.73257400 1.93449800 -2.82711200

C -3.71921100 2.62462300 -4.09726900

H -2.73031600 3.07799600 -4.22900800

H -4.44990200 3.43979600 -4.06965400

C -4.03635400 1.63593800 -5.19046100

C -4.95128800 1.97193100 -6.19397500

C -3.39181600 0.39131800 -5.24297600

C -5.21524600 1.08774400 -7.24262800

H -5.46243700 2.93156500 -6.15527500

C -3.66341400 -0.49510900 -6.28487700

H -2.69107100 0.10959800 -4.46218300

C -4.57189200 -0.14927600 -7.28945400

H -5.92815300 1.36352700 -8.01532000

H -3.16054400 -1.45826000 -6.31302800

H -4.77852000 -0.84181200 -8.10130800

**CP18**

C 1.88996000 -3.98996800 3.54489400

H 2.31558400 -3.40405300 4.37189500

H 1.66024800 -4.98835100 3.93522700

C 2.86566900 -4.02504600 2.35368900

H 2.56578100 -4.82998700 1.67307600

H 3.90434200 -4.21062500 2.65095400

C 0.67113500 -3.26749100 2.95385700

H 0.14983200 -3.94982200 2.27414900

H -0.04633100 -2.92402800 3.70881200

C 0.61114600 -1.57270200 0.89839900

C 1.16462900 -2.29667100 -0.31899400

C 1.29331800 -2.08874000 2.15229200

C 0.36831900 -3.54694300 -0.68175000

O 0.52701700 -4.66321500 -0.22653400

O -0.59815700 -3.25177800 -1.58270900

C -1.45269500 -4.34698200 -1.95199200

H -0.86292000 -5.16184000 -2.38074800

H -1.99509400 -4.71955100 -1.07935900

H -2.14609800 -3.93993600 -2.68766900

C 2.67942000 -2.65289200 1.65760600

H 3.47324500 -1.96509200 1.96214100

C 2.62934500 -2.70061700 0.10818600

C 3.58146300 -1.72818000 -0.54158000

O 3.86855800 -0.62321600 -0.09966900

O 4.06004700 -2.19957600 -1.70669900

C 4.93536600 -1.32822000 -2.46488900

H 4.44420100 -0.35651700 -2.58127400

C -2.97969900 -0.60392800 -0.13360100

C -3.03089900 0.25179200 -1.23580300

C -3.66458000 -0.15418200 -2.45698600

C -4.33642100 -1.41613800 -2.49746300

C -4.36422700 -2.20891500 -1.32585800

C -3.69899700 -1.84901500 -0.16799800

H -3.11976000 1.58333500 -3.63683000

C -3.63800700 0.63065100 -3.64451400

C -4.96389300 -1.83796000 -3.70153500

H -4.91301900 -3.14719500 -1.34761600

C -4.92196100 -1.05305400 -4.83103800

C -4.24688700 0.19124600 -4.79959000

H -5.47616100 -2.79736100 -3.71269900

H -5.40118300 -1.38558500 -5.74779300

H -4.20686900 0.80370300 -5.69636000

C -2.46521500 1.63676000 -1.13381200

C -3.37152500 2.75225100 -1.10479500

C -1.08934400 1.83803100 -1.03029800

C -4.78791600 2.60329900 -1.10589900

C -2.83633800 4.07669700 -1.02600500

C -0.56091600 3.17378700 -1.01989300

C -5.61799300 3.70151600 -1.05189500

H -5.21518200 1.60802200 -1.14108800

C -3.71807400 5.19034200 -0.98213300

C -1.43163700 4.24542800 -1.01203300

C -5.08189800 5.01036800 -0.99597000

H -6.69560200 3.56160800 -1.04907300

H -3.29141100 6.18935500 -0.93200700

H -1.02905200 5.25521100 -1.01810700

H -5.74873000 5.86741500 -0.95796200

C 0.89822700 3.48359500 -1.10337300

C 1.64462700 3.11587800 -2.23480000

C 1.52314000 4.25624100 -0.11116000

C 2.97190100 3.52537800 -2.37988700

H 1.16927600 2.53706000 -3.02258000

C 2.85159600 4.65938800 -0.24967400

H 0.96131600 4.53227100 0.77632500

C 3.57898000 4.30381700 -1.38879700

H 3.52557900 3.25352900 -3.27504500

H 3.31852700 5.25348400 0.53096200

H 4.60855300 4.63171200 -1.50453900

C -3.85424600 -2.74274600 1.01821500

C -5.14830500 -3.07817900 1.45471700

C -2.75776800 -3.30642200 1.68910300

C -5.34064700 -3.94966500 2.52661800

H -6.00659200 -2.63815900 0.95400500

C -2.95095200 -4.18098100 2.76001700

H -1.75001400 -3.06514400 1.36454900

C -4.24096600 -4.50632300 3.18259300

H -6.34992500 -4.18983000 2.85103400

H -2.08702400 -4.61171400 3.25782600

H -4.38816800 -5.18688300 4.01698200

C -0.18465400 0.64192500 -0.86031600

H -0.44025000 -0.14904100 -1.57300500

C -2.20974100 -0.15193900 1.08047300

H -2.41660300 -0.77006800 1.95619900

P -0.31574900 -0.15594100 0.83166700

C 0.26823400 1.05578200 2.09667600

C -0.58214200 1.67073300 3.01254600

C 1.65116300 1.33342100 2.10607300

C -0.06045000 2.58529900 3.94604600

H -1.64434900 1.47002500 3.05634300

C 2.15623700 2.24243000 3.03944400

H 2.30609100 0.82505400 1.40741900

C 1.29945100 2.87650100 3.95889700

H 1.74044400 3.56811600 4.66607800

O -0.97441400 3.12898100 4.79980700

O 3.46414300 2.58936500 3.15719500

C 4.42746400 1.93599900 2.33219700

H 4.30557800 2.19001700 1.27532600

H 5.39870300 2.29783200 2.67391000

H 4.37978800 0.84708100 2.44800400

C -0.51581700 4.05015400 5.77639600

H -1.39941600 4.35117000 6.34211700

H 0.21153800 3.58781600 6.45661100

H -0.06191200 4.93621600 5.31303200

H 0.86895100 0.89441400 -0.99232500

H -2.48620900 0.87850200 1.31920300

C 5.23104200 -1.97563500 -3.79339800

C 4.22881400 -2.63046200 -4.52108300

C 6.51607800 -1.88220500 -4.33921500

C 4.50988000 -3.18198800 -5.77076400

H 3.23277600 -2.72025400 -4.09789600

C 6.79581000 -2.42488000 -5.59499400

H 7.30242200 -1.38165800 -3.77889300

C 5.79332200 -3.07783200 -6.31326800

H 3.72570400 -3.69415300 -6.32212100

H 7.79873100 -2.34503400 -6.00578800

H 6.01066800 -3.50722400 -7.28761200

H 5.85097000 -1.15226500 -1.89287000

H 1.46692600 -1.27355400 2.86602000

H 1.16867900 -1.67013200 -1.21845200

H 2.85773800 -3.69812200 -0.27294500

O 5.47600800 1.58463600 -0.94030000

H 5.00594900 0.79637900 -0.60938200

H 4.75652700 2.17969700 -1.20123800

**TS18**

C 0.40013300 -2.99274500 4.51828500

H 0.55725800 -2.45769700 5.46516200

H -0.23403300 -3.86074500 4.73105700

C 1.74921500 -3.37129600 3.88367300

H 1.57838100 -4.12782900 3.11011900

H 2.46553900 -3.78240400 4.60415700

C -0.20920000 -2.04847500 3.46995000

H -0.57634700 -2.64558900 2.62794800

H -1.04927200 -1.45833300 3.85678100

C 1.08012600 -0.69794800 1.56756800

C 1.91909200 -1.72937100 0.75829900

C 0.98430400 -1.16478500 3.03007300

C 1.26000500 -2.92117300 0.08777000

O 0.88110300 -3.94622800 0.62375100

O 1.21605200 -2.73901800 -1.25315300

C 0.84758400 -3.88700600 -2.03613800

H 1.60889100 -4.66557800 -1.92986500

H -0.11977200 -4.27913800 -1.72271600

H 0.80070400 -3.53559600 -3.06655400

C 2.25699400 -2.05797800 3.23468200

H 2.96026300 -1.55488500 3.90199600

C 2.93463600 -2.21581000 1.85286700

C 4.25145500 -1.44176400 1.75458400

O 4.75556000 -0.78812800 2.64490200

O 4.82364300 -1.65161500 0.55054300

C 6.04326400 -0.92434400 0.25378500

H 6.14556900 -0.12579900 0.99197600

C -2.22969800 -1.30189900 -0.59640500

C -2.10727200 -0.67751800 -1.83791600

C -2.15494800 -1.45671200 -3.04320300

C -2.44074200 -2.85620200 -2.95404800

C -2.68492200 -3.42603400 -1.68093000

C -2.58137900 -2.69269800 -0.51462400

H -1.67566100 0.15435500 -4.41369000

C -1.91153600 -0.90078900 -4.33089500

C -2.49451000 -3.63622300 -4.14141800

H -2.98288200 -4.47013100 -1.62442800

C -2.25554000 -3.06618600 -5.37081200

C -1.95458000 -1.68549700 -5.46218500

H -2.72330600 -4.69609000 -4.05772700

H -2.29341100 -3.67193800 -6.27202500

H -1.75481800 -1.24200800 -6.43371300

C -2.01646300 0.82099800 -1.94027500

C -3.15739100 1.52543400 -2.46266600

C -0.88427100 1.51958400 -1.51961600

C -4.37952000 0.88409900 -2.81519100

C -3.08281000 2.94719200 -2.60382300

C -0.80568700 2.94438200 -1.69665600

C -5.44951400 1.60638600 -3.29621200

H -4.47128800 -0.18894800 -2.69501200

C -4.19919900 3.66323100 -3.11578000

C -1.89063300 3.61077900 -2.23349900

C -5.36012800 3.00978200 -3.45745700

H -6.37220900 1.09324100 -3.55332600

H -4.11768200 4.74208000 -3.22453600

H -1.82549600 4.68456300 -2.38832200

H -6.20986700 3.56596500 -3.84364500

C 0.40209200 3.75432900 -1.36059600

C 1.66346100 3.46078600 -1.90243900

C 0.27385400 4.89225600 -0.54681200

C 2.76363100 4.27397100 -1.63238700

H 1.78133400 2.59763300 -2.55183500

C 1.37158300 5.71076400 -0.28201500

H -0.69177200 5.12206400 -0.10539400

C 2.62127200 5.40356800 -0.82452300

H 3.73277900 4.02359400 -2.05419600

H 1.25162600 6.58158000 0.35688700

H 3.47898500 6.03791100 -0.61731700

C -2.95126100 -3.36072400 0.77087100

C -4.04691700 -2.90044000 1.52248000

C -2.27007300 -4.50659000 1.21065000

C -4.44515000 -3.56193600 2.68411800

H -4.60237100 -2.03078600 1.17983600

C -2.67125600 -5.16830600 2.37322300

H -1.39960000 -4.85292700 0.66388100

C -3.75777900 -4.69967100 3.11336700

H -5.29816000 -3.19366700 3.24798600

H -2.12573500 -6.04846700 2.70281200

H -4.06761900 -5.21582100 4.01802000

C 0.21392000 0.76100500 -0.80824800

H 0.47787700 -0.16142700 -1.33913100

C -2.02452800 -0.48466700 0.65877600

H -2.26729300 -1.04915400 1.55984600

P -0.30622900 0.22084400 0.87923800

C -0.53827300 1.66746200 1.97863500

C -1.77209600 1.95596800 2.56696200

C 0.59061600 2.47168300 2.21997700

C -1.88962400 3.07974700 3.40246700

H -2.65765000 1.34957400 2.42742500

C 0.44930600 3.58251600 3.05481700

H 1.54952500 2.20762000 1.75229800

C -0.78769200 3.89600000 3.64503400

H -0.84105400 4.76672200 4.28705500

O -3.13160900 3.27989400 3.93313300

O 1.46668100 4.43076900 3.35648000

C 2.78267900 4.06507800 2.93437900

H 2.85240300 3.97187500 1.84629100

H 3.43384200 4.86745500 3.28661800

H 3.08623600 3.11425200 3.38814900

C -3.32065200 4.38770300 4.79866100

H -4.36828600 4.35383900 5.10349500

H -2.68092300 4.31842800 5.68837300

H -3.12229000 5.33872000 4.28682100

H 1.13731700 1.32507800 -0.65047300

H -2.67153800 0.39803000 0.63229700

C 5.98060600 -0.39390700 -1.15524400

C 4.99241300 0.54048300 -1.50668900

C 6.91191700 -0.81068200 -2.11165400

C 4.95266200 1.03624200 -2.81044200

H 4.25921000 0.86176200 -0.75798000

C 6.87250800 -0.30145200 -3.41219700

H 7.67398300 -1.53811000 -1.83871800

C 5.88890600 0.62292600 -3.76401900

H 4.18420500 1.75417000 -3.08811900

H 7.60354600 -0.63059300 -4.14639000

H 5.85061700 1.01936800 -4.77586600

H 6.87939600 -1.62060300 0.37972200

H 1.03081400 -0.28808300 3.68670800

H 2.45149900 -1.18493400 -0.02191200

H 3.19359200 -3.26291000 1.66091900

O 2.90042600 1.15711500 0.88052300

H 1.89529400 0.20355600 1.46072900

H 3.62265400 1.22261200 1.52565100

**CP19**

C 1.60042200 -4.12775600 2.49190600

H 2.07947000 -4.08686600 3.48066100

H 1.12001800 -5.10881700 2.40334900

C 2.64236500 -3.86849600 1.39288400

H 2.22537100 -4.16886300 0.42365900

H 3.58209600 -4.41603500 1.52956000

C 0.61288100 -2.96031500 2.32038800

H -0.04903300 -3.17031400 1.47910300

H -0.01071500 -2.78185700 3.19801500

C 1.06257400 -0.67961700 0.95886900

C 1.70060300 -1.09641400 -0.39199700

C 1.51011900 -1.73878900 2.01192600

C 0.95697400 -2.12585800 -1.22567300

O 0.11068700 -2.90878500 -0.84461400

O 1.38555300 -2.08249900 -2.50755300

C 0.76341100 -3.02331700 -3.40037100

H 0.98416400 -4.04914400 -3.09177700

H -0.31991200 -2.88001600 -3.41323600

H 1.19165600 -2.81927300 -4.38213500

C 2.84550000 -2.33493200 1.43452900

H 3.68568200 -2.11007000 2.10050800

C 3.08357800 -1.66388500 0.04495800

C 4.10962100 -0.54507600 0.15863800

O 3.87154100 0.64723200 0.22416300

O 5.35761000 -1.05097500 0.21280800

C 6.42627400 -0.08023500 0.39407400

H 6.39700600 0.62569600 -0.44085000

C -2.88963400 -1.33728000 -0.27705500

C -3.17562100 -0.56693000 -1.40007200

C -3.52817000 -1.18701100 -2.64953600

C -3.65980600 -2.60994600 -2.70163200

C -3.46711000 -3.35417200 -1.51387600

C -3.09486600 -2.76266900 -0.32349000

H -3.59601000 0.62223800 -3.84539800

C -3.71509800 -0.45494900 -3.85674000

C -3.99714700 -3.24073500 -3.93025600

H -3.63826400 -4.42733300 -1.54044800

C -4.18071800 -2.50122800 -5.07634100

C -4.03059800 -1.09436700 -5.03610700

H -4.10404100 -4.32309400 -3.94592500

H -4.43534400 -2.99265400 -6.01167300

H -4.16322600 -0.51289100 -5.94467300

C -3.08290300 0.92481200 -1.35216300

C -4.28197400 1.70490500 -1.46978900

C -1.83060800 1.53209800 -1.24654000

C -5.58538900 1.13261200 -1.45550000

C -4.17257400 3.12731400 -1.56605500

C -1.72831300 2.95332500 -1.47744700

C -6.71047900 1.92480100 -1.53481900

H -5.68869500 0.05644300 -1.36765400

C -5.34995600 3.91677500 -1.65817100

C -2.88055800 3.70344800 -1.61522400

C -6.59589700 3.33149000 -1.64183500

H -7.69531800 1.46551400 -1.51322200

H -5.24691700 4.99668000 -1.73804500

H -2.79828800 4.76990600 -1.81006000

H -7.49087700 3.94446900 -1.70726300

C -0.43074000 3.65322900 -1.71064900

C 0.48884400 3.17118200 -2.65892200

C -0.14921100 4.87612000 -1.08031100

C 1.65158300 3.88160500 -2.95807900

H 0.27603000 2.24376500 -3.18304200

C 1.01145000 5.58994900 -1.37998900

H -0.83790000 5.25492700 -0.33139400

C 1.91833000 5.09550200 -2.32025100

H 2.34333300 3.49190800 -3.70057200

H 1.21059600 6.52911200 -0.87059400

H 2.82147700 5.65209500 -2.55684000

C -2.99712900 -3.65599200 0.86939300

C -3.79112500 -3.44402100 2.00920100

C -2.18282200 -4.79951200 0.82881900

C -3.76442100 -4.34067500 3.07752400

H -4.45242200 -2.58240500 2.04729900

C -2.15997000 -5.69965200 1.89518500

H -1.54993400 -4.96011000 -0.03840400

C -2.94857700 -5.47276200 3.02475800

H -4.39056400 -4.15962800 3.94738400

H -1.52020500 -6.57717500 1.84465200

H -2.92921200 -6.17239200 3.85616500

C -0.65046500 0.68994600 -0.85830500

H -0.62652900 -0.22132100 -1.46269700

C -2.38570700 -0.67930800 0.98108900

H -2.36733400 -1.37185900 1.81594800

P -0.65523000 0.13446900 1.05972300

C -0.57449200 1.92054200 1.61984000

C -1.66419400 2.51408300 2.25569500

C 0.63540600 2.62513400 1.50213700

C -1.54941300 3.81070600 2.78842300

H -2.62176800 2.01395100 2.36203400

C 0.74801900 3.90108600 2.05999600

H 1.47930500 2.19144100 0.98116200

C -0.34389600 4.50418000 2.70578500

H -0.20877100 5.49461100 3.12192800

O -2.67706700 4.29806200 3.38258700

O 1.88480400 4.65090700 2.02943000

C 3.04197700 4.09791400 1.41045900

H 2.88143700 3.93201900 0.33937500

H 3.83146200 4.83937400 1.54965000

H 3.33977100 3.15301400 1.88068300

C -2.63227500 5.59972400 3.94252700

H -3.62803100 5.78533700 4.34971700

H -1.89132500 5.66718800 4.75029200

H -2.40359100 6.35839900 3.18220900

H 0.28333300 1.22928500 -1.01973800

H -3.04352600 0.15342300 1.26382100

C 7.73163200 -0.82417200 0.45634600

C 8.51000000 -0.99392500 -0.69484100

C 8.18176100 -1.36794900 1.66632400

C 9.71643500 -1.69338100 -0.63995000

H 8.16911200 -0.57374400 -1.63828800

C 9.38608400 -2.06912000 1.72497300

H 7.58435100 -1.23933600 2.56598600

C 10.15605800 -2.23235500 0.57075300

H 10.31316600 -1.81511300 -1.53988000

H 9.72568900 -2.48398800 2.67012900

H 11.09668400 -2.77465400 0.61596700

H 6.22871200 0.47918900 1.31345500

H 1.70382000 -1.21297000 2.94806500

H 1.86857200 -0.23298200 -1.03624600

H 3.48066100 -2.38916400 -0.67248200

H 1.64533000 0.21080900 1.22260600

O -0.61311300 -0.30284600 2.79963500

H -1.05989300 0.37695200 3.32635600

**TS19**

C -0.78306200 4.17925600 3.30962500

H -0.89346900 4.01436600 4.39041900

H -0.28469400 5.14530600 3.17309700

C -2.15048400 4.09071000 2.61663400

H -2.07449400 4.52007100 1.60857400

H -2.94238400 4.63780800 3.14065500

C -0.00843700 3.01460200 2.67287800

H 0.30566800 3.29925700 1.66417500

H 0.89310400 2.75192800 3.23788600

C -1.05672000 0.92559800 1.37741700

C -1.94993700 1.57427300 0.31075700

C -1.03701000 1.86507300 2.61278700

C -1.27997500 2.51059100 -0.58778000

O -0.07698000 2.48924200 -0.86749300

O -2.12367300 3.38803400 -1.19731300

C -1.53050500 4.23717000 -2.18325600

H -0.77361600 4.88947800 -1.73634400

H -1.05882600 3.64827000 -2.97515000

H -2.35003500 4.83322100 -2.58840400

C -2.44697400 2.57412400 2.52539900

H -3.06775800 2.25637400 3.36732600

C -3.10633000 2.13460200 1.18916500

C -4.12416300 1.02263500 1.39972300

O -4.12140000 0.23746900 2.33078300

O -5.02482600 0.99129800 0.40015100

C -5.78781900 -0.23268400 0.29403000

H -5.05832400 -1.04941500 0.28474900

C 2.76667900 1.30967800 -0.26780000

C 2.98497000 0.46083700 -1.34912700

C 3.42254300 0.98531400 -2.61384900

C 3.70057800 2.38312700 -2.72325500

C 3.56713500 3.19479500 -1.57358300

C 3.11931400 2.69840800 -0.36566200

H 3.34025900 -0.87757600 -3.72594700

C 3.56543300 0.18129000 -3.78024300

C 4.12776900 2.91985800 -3.96799900

H 3.84951300 4.24220300 -1.64186400

C 4.26070000 2.11331500 -5.07447100

C 3.97010700 0.73138300 -4.97679000

H 4.34279200 3.98411900 -4.02865400

H 4.58322100 2.53284100 -6.02352300

H 4.06518200 0.09858100 -5.85503800

C 2.77474900 -1.02246500 -1.24197000

C 3.92351300 -1.88342200 -1.24601800

C 1.48660600 -1.55260300 -1.18490700

C 5.26019900 -1.39518400 -1.20551900

C 3.72089100 -3.29943600 -1.25791600

C 1.29057300 -2.97351900 -1.28615100

C 6.33107600 -2.26160200 -1.18172200

H 5.43130200 -0.32463600 -1.18401700

C 4.84653600 -4.16728800 -1.24520300

C 2.39883900 -3.79945000 -1.31605300

C 6.12580500 -3.66228700 -1.20636800

H 7.34293900 -1.86722200 -1.14397500

H 4.67528400 -5.24102100 -1.26235800

H 2.25478700 -4.87251700 -1.41216300

H 6.97969600 -4.33413600 -1.19200100

C -0.04925100 -3.60742000 -1.44818900

C -0.93188100 -3.18938700 -2.45737800

C -0.41415000 -4.70993600 -0.65878700

C -2.14094900 -3.85109800 -2.66909800

H -0.65678000 -2.35467700 -3.09638900

C -1.62364800 -5.37259300 -0.86913500

H 0.24983100 -5.03562400 0.13674300

C -2.49193700 -4.94568500 -1.87615900

H -2.80634900 -3.51636400 -3.46058800

H -1.88920200 -6.21769800 -0.23963600

H -3.43439400 -5.46070800 -2.04164300

C 3.10281000 3.64087800 0.79005400

C 3.85182900 3.38577600 1.95120500

C 2.41682400 4.86259000 0.69410300

C 3.90695100 4.31966300 2.98702000

H 4.41895600 2.46152800 2.02974500

C 2.47511800 5.79863200 1.72716200

H 1.82142000 5.05994900 -0.19257800

C 3.21941500 5.53030400 2.87817400

H 4.49895700 4.10642900 3.87321900

H 1.93605300 6.73786100 1.63392100

H 3.26521900 6.25932700 3.68257200

C 0.33554600 -0.60468000 -0.92134100

H 0.37591500 0.29500600 -1.54122000

C 2.15986200 0.74726400 1.00619100

H 2.10915300 1.49860900 1.79341100

P 0.45596800 0.00200500 0.81977300

C 0.37991000 -1.47725000 1.90067500

C 1.38226500 -1.84027800 2.79607100

C -0.82335500 -2.20623300 1.81708300

C 1.18817900 -2.95922400 3.62752800

H 2.31092600 -1.29316000 2.90432100

C -1.00948200 -3.29520600 2.67060700

H -1.58750900 -1.93034100 1.09032400

C -0.00000600 -3.68307400 3.57138700

H -0.19212900 -4.53244300 4.21531400

O 2.22355000 -3.23892000 4.47080600

O -2.14167200 -4.04016600 2.70145100

C -3.28584400 -3.53863600 1.99675500

H -3.11472800 -3.51601600 0.91617400

H -4.09380400 -4.23438200 2.23132600

H -3.54595300 -2.53008800 2.33708500

C 2.08998700 -4.33964200 5.35591700

H 3.01967100 -4.37913900 5.92653900

H 1.24593600 -4.20266500 6.04456500

H 1.95875100 -5.28282800 4.80940500

H -0.67736100 -1.02735900 -0.99207000

H 2.77719900 -0.07768000 1.37512200

C -6.57703500 -0.18366900 -0.98792800

C -5.92370800 0.06879900 -2.20287800

C -7.95448400 -0.42330800 -0.99752800

C -6.63654500 0.08222900 -3.40018600

H -4.85497300 0.26231200 -2.19097400

C -8.67044700 -0.41921100 -2.19775500

H -8.47177700 -0.61230800 -0.05939800

C -8.01314900 -0.16462800 -3.40156900

H -6.11979100 0.28395300 -4.33522900

H -9.74108900 -0.60706800 -2.18926300

H -8.56853800 -0.15561600 -4.33593100

H -6.43859400 -0.33993600 1.16737800

H -0.97113400 1.26753500 3.52924700

H -3.60412600 2.96043200 0.67592400

H -1.64279000 0.06243000 1.70494600

O -2.66299600 -0.80921400 -0.45358700

H -2.36284300 0.50931800 -0.30953700

H -2.99269700 -0.95215000 -1.35463400

**TS20**

C -2.10431800 1.59356200 4.55916000

H -2.51013200 0.84273000 5.25158400

H -1.72263500 2.42691500 5.16001200

C -3.17506200 2.01861400 3.54539300

H -2.82811000 2.91353000 3.01120600

H -4.14567400 2.25806600 3.99502000

C -1.03167900 0.97057200 3.65079900

H -0.46322200 1.77928700 3.17729700

H -0.32022300 0.33236100 4.18769600

C -1.29333000 0.31795800 1.13619700

C -2.26065700 1.28134100 0.36691700

C -1.82518300 0.18058600 2.56624700

C -1.86692800 2.27989100 -0.55666800

O -0.72361500 2.47100700 -1.00483200

O -2.93097300 3.05001600 -0.99063100

C -2.60876500 4.01685600 -1.98563200

H -1.89435200 4.75833700 -1.61146400

H -2.17565700 3.54536300 -2.87391200

H -3.55280300 4.50441800 -2.24029500

C -3.25781300 0.83060000 2.56321600

H -3.99134400 0.10836300 2.94132200

C -3.59309500 1.24540800 1.09622200

C -4.53767000 0.21419300 0.49083800

O -4.22483400 -0.91927500 0.16757800

O -5.79818100 0.68563100 0.37633800

C -6.77318900 -0.24053300 -0.15816400

H -6.49830500 -0.49415000 -1.18667400

C 2.24662600 1.83218000 0.12771800

C 2.72099800 1.43966200 -1.12280900

C 2.97775300 2.41289800 -2.14489800

C 2.78158100 3.79778600 -1.84290000

C 2.33373800 4.16022600 -0.55115800

C 2.05800500 3.22164900 0.42116200

H 3.52137000 1.02304900 -3.72133700

C 3.39203000 2.06804500 -3.46313700

C 3.03462400 4.77592000 -2.84230400

H 2.19975900 5.21480400 -0.32339900

C 3.44763800 4.40868600 -4.10212800

C 3.62036100 3.03848300 -4.41365300

H 2.88631200 5.82381900 -2.59183600

H 3.63394100 5.16421300 -4.86068000

H 3.93259900 2.74958900 -5.41379000

C 2.93072200 -0.01481000 -1.42673600

C 4.26632500 -0.51552900 -1.59329600

C 1.83412700 -0.87245000 -1.53360700

C 5.42368000 0.29098800 -1.39946300

C 4.45396000 -1.89287700 -1.93212600

C 2.02997600 -2.24169600 -1.92661100

C 6.68847100 -0.23469800 -1.54705300

H 5.30000600 1.33245600 -1.12495900

C 5.77130800 -2.40309300 -2.08893400

C 3.31509600 -2.71237300 -2.11494800

C 6.86796300 -1.59371700 -1.90114500

H 7.55704000 0.39899800 -1.38919000

H 5.89496800 -3.45023000 -2.35533200

H 3.46095400 -3.74287400 -2.42906600

H 7.87123500 -1.99424700 -2.01886500

C 0.90160500 -3.18322900 -2.19182600

C -0.07785600 -2.89540900 -3.15804400

C 0.84118100 -4.41996600 -1.53031800

C -1.08983900 -3.81141500 -3.44589100

H -0.03231900 -1.95498100 -3.70104600

C -0.16805900 -5.33940600 -1.82061800

H 1.58105400 -4.64955800 -0.76902200

C -1.13868900 -5.03781500 -2.77847200

H -1.83459900 -3.57030400 -4.19966900

H -0.19924800 -6.28800900 -1.29115100

H -1.92420300 -5.75338200 -3.00657000

C 1.57080000 3.71084800 1.74556000

C 2.34419700 3.57868900 2.91045100

C 0.33912300 4.37935600 1.82883900

C 1.89830300 4.09961300 4.12620100

H 3.31005000 3.08205600 2.85656500

C -0.10405800 4.90545300 3.04419900

H -0.26850700 4.46343200 0.93324100

C 0.67277900 4.76647100 4.19670100

H 2.51345400 3.99357700 5.01610100

H -1.05942600 5.42207600 3.09025700

H 0.32807900 5.17725000 5.14203600

C 0.46379100 -0.36090500 -1.14767600

H 0.25313100 0.64411100 -1.53341700

C 1.89004700 0.76799200 1.13317900

H 1.75092200 1.17189400 2.13673700

P 0.30180700 -0.17949900 0.69826300

C 0.50223600 -1.83000600 1.49407800

C 1.63869700 -2.22546700 2.19496600

C -0.58266300 -2.71578800 1.34160400

C 1.69854500 -3.51611400 2.75425700

H 2.49223200 -1.57788500 2.34857900

C -0.51087900 -3.99247200 1.89767700

H -1.46577400 -2.38380900 0.81056000

C 0.63317200 -4.40038800 2.60679000

H 0.64395000 -5.39917600 3.02534600

O 2.84860800 -3.80174000 3.42900800

O -1.50188800 -4.92072700 1.81329100

C -2.68754900 -4.57478900 1.10069800

H -2.47256400 -4.38210000 0.04328800

H -3.34529800 -5.44150700 1.18858500

H -3.17960700 -3.69596700 1.53415900

C 2.97883900 -5.08281500 4.02439700

H 3.96007900 -5.09212300 4.50233000

H 2.20356600 -5.25611200 4.78236300

H 2.93311300 -5.88265100 3.27343000

H -0.33566500 -1.03082200 -1.46651000

H 2.68419300 0.01785200 1.17592200

C -8.13419100 0.40183700 -0.09097700

C -8.62896500 0.89738300 1.12358600

C -8.93658100 0.48614900 -1.23329000

C -9.89966900 1.46570500 1.19121600

H -8.00667600 0.84702300 2.01304600

C -10.21432200 1.04723800 -1.16665000

H -8.55880000 0.11264900 -2.18235500

C -10.69793800 1.53915000 0.04571100

H -10.26975900 1.85055200 2.13790600

H -10.82612700 1.10556500 -2.06296700

H -11.68974100 1.98019000 0.09950700

H -6.72723900 -1.16457500 0.42933100

H -1.88831000 -0.87029200 2.86719000

H -2.06715800 0.02599100 0.13374300

H -4.08731400 2.21769500 1.06058000

**TS4b**

C 1.44615200 -1.31203800 5.30314800

H 1.00662400 -0.40529400 5.74272200

H 1.54959900 -2.04284000 6.11461900

C 2.79898400 -0.98186500 4.64866700

H 3.37636800 -1.90805000 4.53274700

H 3.39590300 -0.31545000 5.28513100

C 0.54323000 -1.81464500 4.17117400

H 0.91619500 -2.77651600 3.80241200

H -0.48604700 -1.98441600 4.51790700

C 0.36375000 -1.01869800 1.69335500

C 0.95720300 -2.14175400 1.12172600

C 0.57896900 -0.74803800 3.08520400

C 1.09993600 -2.57134700 -0.25018500

O 0.83841900 -1.97334500 -1.29413800

O 1.62532400 -3.83568500 -0.28986700

C 1.94323000 -4.32411300 -1.59601300

H 1.06140700 -4.32017200 -2.24308600

H 2.72406400 -3.71274100 -2.05920700

H 2.30500100 -5.34317700 -1.44686000

C 2.58343800 -0.36133900 3.27026400

H 2.43204800 0.71678900 3.25540100

C 3.30827500 -0.88496000 2.18403900

C 3.30146700 -0.22682500 0.92118600

O 2.65105000 0.79458500 0.63567000

O 4.09962400 -0.84115400 -0.01616600

C 4.04226000 -0.30380500 -1.34042500

H 3.04828400 -0.48100900 -1.76493200

C -2.43299600 -1.28939100 -0.59394200

C -2.33241800 -0.72150400 -1.86155100

C -2.24659000 -1.55669500 -3.02354100

C -2.31406500 -2.97677600 -2.85842100

C -2.48633600 -3.51143000 -1.55926800

C -2.54803000 -2.71027300 -0.43641000

H -1.99051300 0.03230700 -4.47765400

C -2.06508400 -1.04025100 -4.33710200

C -2.22449600 -3.81798000 -4.00060600

H -2.58710300 -4.58815400 -1.44760000

C -2.05633500 -3.28507100 -5.25792400

C -1.97033200 -1.88177200 -5.42344000

H -2.28682500 -4.89447300 -3.85939600

H -1.98411100 -3.93719800 -6.12408800

H -1.82563200 -1.46548300 -6.41670300

C -2.31094000 0.77359000 -2.01651700

C -3.46132500 1.42772100 -2.57377300

C -1.20320100 1.51408500 -1.59911900

C -4.65073400 0.73279800 -2.93567400

C -3.43152000 2.84782000 -2.74762300

C -1.16036200 2.93307300 -1.83055200

C -5.73756100 1.40537800 -3.44956100

H -4.69805600 -0.34145900 -2.79814500

C -4.56544200 3.51175200 -3.29016200

C -2.26082600 3.55592100 -2.38876100

C -5.69642700 2.80850700 -3.63494700

H -6.63553300 0.85416200 -3.71536800

H -4.52165400 4.59014800 -3.42340800

H -2.22177700 4.62508700 -2.58138000

H -6.55930700 3.32544500 -4.04590600

C 0.04198500 3.77228000 -1.55081100

C 1.29313200 3.46559600 -2.11230500

C -0.07447400 4.94314200 -0.78439400

C 2.39313200 4.29757700 -1.90522700

H 1.39961600 2.57923400 -2.73146900

C 1.02476800 5.77791900 -0.57981400

H -1.03222600 5.18778300 -0.33359000

C 2.26337200 5.45727600 -1.13817400

H 3.35104500 4.04160900 -2.34957800

H 0.91291700 6.67713800 0.02057800

H 3.12100800 6.10489900 -0.97756100

C -2.77723000 -3.36102500 0.88846400

C -3.92658000 -3.07305400 1.64412200

C -1.87930800 -4.32389400 1.37617000

C -4.16695200 -3.72098800 2.85635300

H -4.64746900 -2.35214600 1.26559300

C -2.12179500 -4.97361500 2.58761300

H -0.97680500 -4.54299500 0.81338300

C -3.26377200 -4.67364500 3.33277100

H -5.06539500 -3.48925200 3.42262400

H -1.41181300 -5.71146400 2.95182300

H -3.45020100 -5.17962500 4.27627100

C -0.08110200 0.80308100 -0.87416100

H 0.22668000 -0.11889500 -1.37438600

C -2.33083400 -0.37635400 0.60747700

H -2.60323400 -0.88065900 1.53562800

P -0.58991800 0.25373000 0.82009400

C -0.71258300 1.75573200 1.85616900

C -1.89926100 2.09747900 2.52334300

C 0.41686100 2.58346900 1.98200300

C -1.96031600 3.25562200 3.30005900

H -2.77875000 1.46620100 2.45297000

C 0.34611800 3.73753300 2.76275100

H 1.34733300 2.30415400 1.49585800

C -0.83856600 4.07740100 3.41936200

H -0.88723300 4.97810000 4.02561600

H 0.81228200 1.40790200 -0.72722000

H -2.97281900 0.50104800 0.48439400

C 5.10602800 -0.96606200 -2.18404800

C 6.36245200 -1.28530000 -1.65237200

C 4.85854100 -1.23183200 -3.53631800

C 7.34928100 -1.85277500 -2.45862700

H 6.55189000 -1.10020900 -0.59968500

C 5.84803600 -1.79252800 -4.34717500

H 3.88180100 -1.00137900 -3.95664200

C 7.09751600 -2.10517800 -3.80987600

H 8.31782800 -2.10001000 -2.03096600

H 5.63819100 -1.99329600 -5.39483800

H 7.86761800 -2.54752000 -4.43664600

H 4.19168100 0.78284200 -1.29674900

H 0.13445100 0.17304400 3.45559500

H 1.35434600 -2.86372200 1.82294100

H 3.84566900 -1.82363200 2.26561400

H 1.22276900 4.37234600 2.85359100

H -2.88387300 3.51098300 3.81214000

**TS7b**

C -3.03557100 -1.13035600 0.43865300

C -3.84285600 -0.21429200 -0.23333800

C -4.90867200 -0.66717800 -1.08083300

C -5.15327700 -2.07386500 -1.18713800

C -4.35548200 -2.96938000 -0.43467200

C -3.32034800 -2.53609400 0.37149100

H -5.53423700 1.28367800 -1.79641900

C -5.71712300 0.21637200 -1.85076200

C -6.19914800 -2.53955500 -2.02977000

H -4.58313000 -4.03145100 -0.47825700

C -6.96724800 -1.65732700 -2.75395600

C -6.71757300 -0.26649800 -2.66525600

H -6.37730900 -3.61030600 -2.09357600

H -7.76208900 -2.02381400 -3.39768400

H -7.31996600 0.42588100 -3.24696000

C -3.57824100 1.25847200 -0.09775100

C -4.49872400 2.07970500 0.63450900

C -2.43282000 1.81357200 -0.66822800

C -5.63901200 1.55654900 1.30824100

C -4.24613700 3.48598700 0.72406700

C -2.21613600 3.23204500 -0.62454500

C -6.48368100 2.38193200 2.01717500

H -5.83654900 0.49125900 1.26529500

C -5.14275900 4.31222000 1.45504100

C -3.11487600 4.02337200 0.06526400

C -6.23895500 3.77483100 2.08917900

H -7.34554200 1.96078400 2.52757000

H -4.94126500 5.37952100 1.50637200

H -2.96154300 5.09927900 0.08842000

H -6.91611700 4.41427600 2.64870600

C -1.09009900 3.90431900 -1.33786500

C -0.94260500 3.78663600 -2.73067200

C -0.20024000 4.73396700 -0.63915500

C 0.07106800 4.47036600 -3.40176600

H -1.64204500 3.17295500 -3.29301400

C 0.81295500 5.41910000 -1.31085600

H -0.29105800 4.81951500 0.43966800

C 0.95319400 5.28901800 -2.69328100

H 0.16732300 4.36848700 -4.47932400

H 1.49877900 6.04839300 -0.75007600

H 1.74517100 5.81864300 -3.21544100

C -2.57928400 -3.54974000 1.18003900

C -2.57018000 -3.48527700 2.58349900

C -1.93466100 -4.62915500 0.55658100

C -1.92609100 -4.46468600 3.33952800

H -3.08754300 -2.67133500 3.08497000

C -1.28990500 -5.60909900 1.31239000

H -1.92595500 -4.68725700 -0.52776500

C -1.28156400 -5.52948000 2.70593000

H -1.93533800 -4.39934200 4.42430500

H -0.78744200 -6.43153500 0.81047200

H -0.77805000 -6.29185700 3.29404100

C -1.41075100 0.87788800 -1.27857500

H -1.90399600 0.13715000 -1.91132400

C -1.82525600 -0.62124000 1.18889800

H -1.36838500 -1.36445000 1.83782600

P -0.47973400 -0.06430100 0.03250000

C 0.57700200 1.18094100 0.85295200

C 0.23843900 1.75258300 2.08911300

C 1.74470400 1.58574100 0.19538200

C 1.06098400 2.72572700 2.65497900

H -0.64495000 1.43401000 2.63051300

C 2.56566900 2.55631000 0.77300500

H 2.05249300 1.14125400 -0.74767500

C 2.22751700 3.12870100 1.99903700

H 2.87110100 3.88045700 2.44892400

H -0.67147600 1.40524800 -1.88376300

H -2.08989100 0.24541100 1.80105000

C 0.07377100 -3.11680000 -4.19024700

H -0.15784300 -4.08482500 -4.65096800

H -0.45574400 -2.34879300 -4.77229800

C 1.57964300 -2.83227600 -4.16809300

C -0.36736600 -3.03944900 -2.72810700

H -1.45940400 -3.12693800 -2.62755100

H 0.07561700 -3.86675600 -2.15965800

C 0.31950200 -1.49280300 -0.77987900

C 1.14636200 -2.33575200 -0.04833400

C 0.12138700 -1.69765200 -2.18803900

H -0.38411300 -0.88757300 -2.71005800

H 1.59301000 -3.18284200 -0.54965400

C 1.38828900 -2.22111800 1.36672800

O 0.86432700 -1.39193000 2.12003200

O 2.27013900 -3.14412800 1.82331900

C 2.55218000 -3.08503200 3.22347300

H 1.64668000 -3.27094400 3.80993600

H 3.29108900 -3.86664400 3.40683700

H 2.95654700 -2.10702600 3.50004700

C 1.91650300 -1.73208600 -3.15818700

H 1.82888000 -0.71253500 -3.53186800

C 3.03759600 -1.94069800 -2.33312600

H 3.41958500 -2.94012500 -2.15128000

C 3.77317800 -0.84325700 -1.79311900

O 3.51688400 0.36036800 -1.92996600

O 4.88619800 -1.25263200 -1.08671200

C 5.68214400 -0.21088000 -0.52798400

H 5.14140600 0.27280400 0.29668000

H 5.85032900 0.56365300 -1.28737400

C 6.99212100 -0.78509200 -0.03942900

C 7.67108700 -0.16336200 1.01580000

C 7.57435400 -1.90273000 -0.64969700

C 8.91036900 -0.63851800 1.44770400

H 7.22390700 0.69886300 1.50694900

C 8.81017500 -2.38385400 -0.21455800

H 7.04356900 -2.39795200 -1.45607000

C 9.48473400 -1.75280900 0.83300500

H 9.42207600 -0.14380500 2.26957900

H 9.24695500 -3.25588700 -0.69517800

H 10.44687400 -2.12926700 1.17085100

H 2.11049400 -3.74855000 -3.87844300

H 1.95520600 -2.55457500 -5.16149300

H 3.47271200 2.84975200 0.25284900

H 0.79550200 3.15797400 3.61603500

**TS4c**

C 1.72590600 -1.15393600 5.36292800

H 1.26716000 -0.24287200 5.77286300

H 1.87977700 -1.84078300 6.20412400

C 3.04553500 -0.80853000 4.65151900

H 3.65101500 -1.71898800 4.55679500

H 3.63778000 -0.09538100 5.23990500

C 0.80611200 -1.74240600 4.28656200

H 1.20100200 -2.70855800 3.95261100

H -0.20368400 -1.92904000 4.68066700

C 0.52870400 -1.08361900 1.77966300

C 1.15495800 -2.20438800 1.24272500

C 0.76593300 -0.73143900 3.14933800

C 1.26906900 -2.65852700 -0.12383000

O 0.85868800 -2.11937400 -1.15211700

O 1.94756200 -3.84072700 -0.17459400

C 2.21153000 -4.34058800 -1.48756900

H 1.28546300 -4.44989600 -2.05947100

H 2.88580700 -3.67069000 -2.03047600

H 2.68846400 -5.31134000 -1.34066100

C 2.76702000 -0.25585600 3.25647200

H 2.56604900 0.81262700 3.20173200

C 3.47783400 -0.79466900 2.16958700

C 3.40562700 -0.18870900 0.88278400

O 2.70660500 0.79669100 0.58123400

O 4.19358500 -0.80807000 -0.05713600

C 4.07674400 -0.31996200 -1.39712100

H 3.06504000 -0.51003200 -1.77082100

C -2.32030200 -1.39478700 -0.61371000

C -2.35029800 -0.78279400 -1.86474500

C -2.38512400 -1.59816900 -3.04674200

C -2.46180300 -3.02457600 -2.91426100

C -2.47765800 -3.59931800 -1.61762800

C -2.39105500 -2.80630900 -0.50098300

H -2.22633300 0.02156900 -4.48050400

C -2.31540900 -1.05219100 -4.35986800

C -2.50515900 -3.83284300 -4.08226500

H -2.54098700 -4.68041400 -1.52113900

C -2.45175400 -3.26915700 -5.33606600

C -2.34679400 -1.86422600 -5.47181100

H -2.57092000 -4.91191900 -3.96375800

H -2.47980100 -3.89811900 -6.22167500

H -2.28601900 -1.42273000 -6.46289900

C -2.31232900 0.71333900 -1.95692100

C -3.43753000 1.44428700 -2.47499300

C -1.19646500 1.40893100 -1.48941600

C -4.64883500 0.81018300 -2.87502800

C -3.36564200 2.87441100 -2.56963600

C -1.15119800 2.82566000 -1.58373800

C -5.70968300 1.54121600 -3.36213900

H -4.73292700 -0.26710300 -2.78880200

C -4.47409700 3.59760500 -3.08747700

C -2.19300000 3.53720500 -2.12451700

C -5.62248000 2.94914400 -3.47919700

H -6.62332900 1.03248400 -3.65765800

H -4.39835500 4.68008100 -3.16004600

H -2.13846700 4.62037400 -2.20224500

H -6.46478000 3.51328900 -3.87023500

C -0.03114800 0.68090500 -0.86325600

H 0.23959800 -0.23365000 -1.39367100

C -2.18698200 -0.56242500 0.63967700

H -2.42022200 -1.14560500 1.53496500

P -0.46331300 0.12390400 0.85258900

H 0.86613200 1.29558200 -0.77395700

H -2.86176300 0.29774500 0.60273900

C 5.10201800 -1.01416900 -2.26229800

C 6.38189800 -1.31311000 -1.77696600

C 4.79336200 -1.32933800 -3.59124800

C 7.33204900 -1.90935900 -2.60612800

H 6.61926900 -1.08899200 -0.74174000

C 5.74607100 -1.91923200 -4.42516900

H 3.79875800 -1.11283900 -3.97542400

C 7.01934900 -2.21130200 -3.93432700

H 8.31963900 -2.14014400 -2.21441900

H 5.48922800 -2.15783800 -5.45422900

H 7.76107300 -2.67575800 -4.57903100

H 4.22823400 0.76727500 -1.40080800

H 0.29654100 0.18998200 3.48565400

H 1.62315500 -2.87225000 1.95392500

H 4.05563300 -1.70702500 2.27026900

H -0.26711800 3.34346300 -1.22240900

H -2.37299900 -3.25618300 0.48809700

C -0.63803400 1.65776900 1.83824700

C -1.82097300 1.96908900 2.52494700

C 0.44651300 2.55218100 1.88743600

C -1.92623600 3.16351300 3.24018100

H -2.66501400 1.28727200 2.51656700

C 0.33137200 3.74347300 2.60439700

H 1.38075100 2.29084800 1.39796700

C -0.85283700 4.05397000 3.27694300

H -2.84781200 3.39435700 3.76711300

H 1.17416800 4.42831300 2.63994400

H -0.93692300 4.98411200 3.83248700

**TS7c**

C -3.08748500 -1.27275400 0.26285300

C -3.65144500 -0.19022900 -0.41128700

C -4.65735600 -0.42579800 -1.41106300

C -5.11016100 -1.76650900 -1.65066600

C -4.53640300 -2.83264200 -0.91114500

C -3.54252600 -2.59239600 0.00574400

H -4.86115400 1.63381000 -2.06146100

C -5.20976600 0.61780300 -2.20751100

C -6.10905600 -2.00136500 -2.63397600

H -4.88592700 -3.84634100 -1.09064500

C -6.63183500 -0.96571400 -3.37322900

C -6.16855900 0.35481200 -3.16060300

H -6.44622500 -3.02226200 -2.79696800

H -7.39180200 -1.15818900 -4.12543600

H -6.57082600 1.16843100 -3.75797300

C -3.16320200 1.19811800 -0.12051600

C -4.02958300 2.17699500 0.47637300

C -1.83772000 1.53128700 -0.40012400

C -5.36379400 1.88146100 0.87806500

C -3.53355700 3.50273800 0.71165100

C -1.36400500 2.84596300 -0.15327900

C -6.16407900 2.84387500 1.45259200

H -5.74881900 0.87858900 0.73185700

C -4.38961200 4.47501300 1.29649800

C -2.19119500 3.80894700 0.37022100

C -5.67845000 4.15759400 1.65806800

H -7.17728400 2.59206400 1.75386500

H -4.00009100 5.47696700 1.46070600

H -1.81993700 4.81563800 0.54548000

H -6.32227300 4.90827000 2.10804400

C -0.88607400 0.48831300 -0.94111300

H -1.37264100 -0.09662500 -1.72312900

C -1.96866200 -1.06692900 1.25677600

H -1.78969500 -1.94739300 1.87645600

P -0.36347500 -0.68748000 0.39991500

H 0.02468700 0.91832500 -1.36708300

H -2.18050100 -0.21887400 1.91409300

C -0.07456700 -4.04044000 -3.64598100

H -0.23284700 -5.06337000 -4.00842100

H -0.73024700 -3.38487900 -4.23699000

C 1.38553100 -3.59305400 -3.78547500

C -0.40536700 -3.88750500 -2.15942300

H -1.47451700 -4.05051200 -1.96285800

H 0.14684000 -4.63307500 -1.57521400

C 0.32326900 -2.19801100 -0.37714300

C 1.21552300 -2.99006200 0.33200500

C 0.00714800 -2.47481700 -1.74699000

H -0.62705500 -1.74368800 -2.24053200

H 1.75982500 -3.76221700 -0.19779500

C 1.32269500 -2.96877700 1.76578700

O 0.56954500 -2.34294400 2.52292400

O 2.32545200 -3.74976600 2.24018200

C 2.45392500 -3.79108300 3.66361700

H 1.54024100 -4.17444700 4.12875800

H 3.29135300 -4.46147000 3.86304300

H 2.66043500 -2.79491700 4.06610500

C 1.63660300 -2.35393900 -2.93050700

H 1.30148800 -1.41514700 -3.37006200

C 2.86719800 -2.25567800 -2.25318800

H 3.50803200 -3.11840600 -2.10603500

C 3.29025000 -0.99791800 -1.75029300

O 2.62251700 0.05310000 -1.76716000

O 4.56785500 -1.03009000 -1.23009800

C 5.10751200 0.19963400 -0.75355900

H 4.81022400 0.35122000 0.29357300

H 4.67797600 1.02557700 -1.33336700

C 6.61476000 0.17690400 -0.87208500

C 7.39701400 0.89873900 0.03724600

C 7.25120400 -0.51469300 -1.91000200

C 8.78628900 0.94198300 -0.09223700

H 6.91534500 1.42893500 0.85675100

C 8.64031800 -0.47872000 -2.03692000

H 6.64957100 -1.09118700 -2.60522700

C 9.41319200 0.25196200 -1.13143200

H 9.37845300 1.50622400 0.62392300

H 9.12086700 -1.02466900 -2.84503900

H 10.49522600 0.27816600 -1.23117000

H 2.04623700 -4.40130400 -3.44483200

H 1.64828000 -3.38944500 -4.83184000

H -0.32989000 3.08210100 -0.38655000

H -3.09436300 -3.41662100 0.55388900

C 0.65669500 0.35055100 1.51736600

C 0.31168100 0.50829900 2.86925100

C 1.74644700 1.06090500 0.99363400

C 1.03176600 1.38458700 3.67836500

H -0.49489500 -0.06878400 3.30346400

C 2.46667000 1.93135900 1.81648500

H 2.05626900 0.91640300 -0.03892000

C 2.10970600 2.10140400 3.15380100

H 0.75396500 1.49922000 4.72244100

H 3.31099800 2.47582900 1.40219400

H 2.67093700 2.78280100 3.78782100

**CP1d**

C -2.53286300 -1.10123100 -0.37134700

C -2.37062000 -0.72526100 -1.70369300

C -2.30125500 -1.73449000 -2.72526300

C -2.46463800 -3.11360900 -2.36388000

C -2.66633900 -3.44955800 -1.00024500

C -2.68197600 -2.47308400 -0.03524400

H -1.88745300 -0.39736700 -4.38205500

C -2.04209000 -1.43063100 -4.09256800

C -2.40252900 -4.11279000 -3.37260600

H -2.79303700 -4.49499400 -0.72894700

C -2.16675900 -3.78007700 -4.68667200

C -1.97640900 -2.42395900 -5.04462600

H -2.53671700 -5.15214100 -3.08139600

H -2.11710000 -4.55365100 -5.44819500

H -1.77327700 -2.16437800 -6.08023100

C -2.22135200 0.72716400 -2.04427400

C -3.21425900 1.40276200 -2.83574500

C -1.12071200 1.43533000 -1.56074900

C -4.41441200 0.76879200 -3.26734000

C -3.02035900 2.77998800 -3.18734700

C -0.94461200 2.79574800 -1.93530100

C -5.35112900 1.44736700 -4.01545700

H -4.59174900 -0.26458600 -2.99150400

C -4.00053100 3.44793600 -3.96986100

C -1.85351800 3.44643300 -2.73265300

C -5.14274100 2.79920400 -4.37933600

H -6.26085200 0.94081900 -4.32675500

H -3.83255800 4.49002300 -4.23242300

H -1.69491700 4.48436200 -3.01605100

H -5.88769300 3.32164700 -4.97334900

C -0.11470100 0.78507400 -0.64407900

H 0.22038600 -0.17041600 -1.06271300

C -2.48485100 -0.06682300 0.72615900

H -2.95116600 -0.45115900 1.63925400

P -0.67571100 0.31400100 1.11582800

H 0.78172400 1.40651800 -0.54536900

H -3.01758300 0.84053500 0.42566600

H -0.05910600 3.32164800 -1.58559800

H -2.81494800 -2.74244000 1.00961700

C -0.77508900 1.93590200 2.12785300

C -1.73391800 3.01881200 1.60602800

H -1.47613500 3.35151200 0.59694600

H -2.77360700 2.67482600 1.58971400

H -1.69575500 3.89647400 2.26701900

C 0.65495400 2.50631500 2.21039500

H 1.37442900 1.75214600 2.55124400

H 0.99851300 2.89027500 1.24315500

H 0.68492100 3.34229400 2.92219100

C -1.21903900 1.50594600 3.54358800

H -0.54383300 0.75559300 3.97008400

H -1.22460300 2.37563500 4.21468000

H -2.23292300 1.08799200 3.54403300

**TS4d**

C 1.67085800 -1.78958900 5.22570800

H 1.38703800 -0.88387200 5.78139600

H 1.74520000 -2.60225200 5.95867800

C 2.99666500 -1.55873700 4.48184700

H 3.42920100 -2.53060300 4.21091900

H 3.72928300 -1.05597000 5.12712100

C 0.62007800 -2.07142800 4.14547100

H 0.83514000 -3.03264000 3.66640300

H -0.38677800 -2.15377900 4.58196400

C 0.36202500 -1.02135100 1.75740100

C 0.86449700 -2.13115600 1.07005100

C 0.68410800 -0.91780300 3.15165700

C 1.00100000 -2.50728700 -0.32125400

O 0.77837100 -1.86674900 -1.34981300

O 1.49550400 -3.77884000 -0.39845300

C 1.80464800 -4.23547600 -1.71714900

H 0.91794200 -4.21795200 -2.35763800

H 2.58173200 -3.61310700 -2.17147100

H 2.16671900 -5.25798400 -1.59389300

C 2.75322600 -0.75387700 3.21053300

H 2.71783500 0.32690300 3.33155000

C 3.32161000 -1.20259600 2.00742200

C 3.30298600 -0.37510000 0.84334700

O 2.73292400 0.72354500 0.74294700

O 3.99169500 -0.92458200 -0.21279000

C 3.95522700 -0.20100800 -1.44718300

H 2.97601700 -0.33554200 -1.91952100

C -2.40302600 -1.17981700 -0.35839900

C -2.31495600 -0.74345200 -1.67638500

C -2.29841600 -1.71305600 -2.73928300

C -2.44621100 -3.10420100 -2.42349100

C -2.58217000 -3.49634900 -1.06732100

C -2.54026000 -2.56136100 -0.06425500

H -1.95651500 -0.31707400 -4.36399800

C -2.10660400 -1.35834000 -4.10441200

C -2.43833100 -4.06510600 -3.47062000

H -2.69868100 -4.55137000 -0.83227100

C -2.26788100 -3.68270400 -4.78121600

C -2.09113800 -2.31455300 -5.09565500

H -2.56149600 -5.11425400 -3.21192000

H -2.25848800 -4.42663400 -5.57318800

H -1.93558000 -2.01578000 -6.12878400

C -2.20636400 0.71898800 -1.98877000

C -3.24779100 1.36785900 -2.74448400

C -1.10209800 1.46047600 -1.56467600

C -4.46955400 0.71699000 -3.07987500

C -3.08080800 2.73046200 -3.15868500

C -0.94686200 2.80076100 -2.01615900

C -5.45240000 1.36662200 -3.79389200

H -4.62769800 -0.30572000 -2.75712900

C -4.10696600 3.36762000 -3.90692700

C -1.88928000 3.41285100 -2.80278800

C -5.26991500 2.70308400 -4.22164600

H -6.37781600 0.84805500 -4.02958600

H -3.95709600 4.39849400 -4.21937500

H -1.74165500 4.43387600 -3.14637800

H -6.05026200 3.20164100 -4.79005300

C -0.02256700 0.87486800 -0.68056000

H 0.36010400 -0.06621300 -1.09701900

C -2.30390600 -0.20073800 0.78466000

H -2.62753000 -0.65663400 1.72409200

P -0.54711700 0.37436700 1.02826800

H 0.83617600 1.54011000 -0.56510500

H -2.91987700 0.68682400 0.60608000

C 5.05418600 -0.71094800 -2.34921100

C 6.31750900 -1.03786100 -1.83871300

C 4.83486100 -0.82021800 -3.72731500

C 7.33857800 -1.45873600 -2.69045500

H 6.48657600 -0.97449800 -0.76818600

C 5.85834000 -1.23375700 -4.58314900

H 3.85370000 -0.58311000 -4.13332800

C 7.11436200 -1.55476800 -4.06656900

H 8.31237900 -1.71338400 -2.27939200

H 5.67011700 -1.31378900 -5.65089200

H 7.91118600 -1.88275800 -4.72917600

H 4.07843400 0.86871400 -1.23855300

H 0.36392900 -0.00652600 3.64206500

H 1.22429700 -2.92091200 1.71574700

H 3.77517000 -2.18426900 1.92623600

H -0.04529100 3.33997800 -1.73702800

H -2.61309200 -2.87198000 0.97422600

C -0.69643500 1.91765300 2.14440900

C -1.29428000 3.05984600 1.29011800

H -0.59378500 3.41655500 0.53361800

H -2.22966900 2.79271200 0.79051300

H -1.50953400 3.90024300 1.96119700

C 0.68011600 2.39908200 2.65926700

H 1.02810300 1.82373400 3.51813500

H 1.45798800 2.33633200 1.89450300

H 0.58063400 3.44283100 2.98279000

C -1.67488800 1.66768400 3.31692600

H -1.39369300 0.82284800 3.94938600

H -1.68862300 2.56487900 3.94819400

H -2.69901200 1.50343500 2.96553000

**TS7d**

C -2.19992400 -0.99112600 0.65570000

C -3.40417700 -0.48515000 0.17192400

C -4.40388800 -1.39143900 -0.33028000

C -4.17563600 -2.80502200 -0.24319000

C -2.96030900 -3.27520900 0.31742300

C -1.99453600 -2.39433500 0.73444200

H -5.78880800 0.11503800 -1.05340700

C -5.60786600 -0.94856800 -0.94882700

C -5.16082600 -3.70448600 -0.73295800

H -2.79442000 -4.34701100 0.39209300

C -6.32007700 -3.23999800 -1.30994400

C -6.53743800 -1.84649300 -1.42563000

H -4.97317600 -4.77245000 -0.65111400

H -7.06370100 -3.93742200 -1.68557900

H -7.44486400 -1.48095700 -1.89865500

C -3.64490500 0.99297800 0.15057300

C -4.70537800 1.56863100 0.93652900

C -2.83973700 1.82337600 -0.62719500

C -5.47906200 0.80518000 1.85656100

C -4.98681000 2.97050900 0.82498100

C -3.15206700 3.20552500 -0.73952000

C -6.48104600 1.38810500 2.60060100

H -5.26535900 -0.25065200 1.97658200

C -6.03658300 3.53835300 1.59607400

C -4.20130800 3.76175200 -0.05178900

C -6.77204600 2.76650900 2.46552700

H -7.05174100 0.78423900 3.30076000

H -6.24091100 4.60125800 1.49135400

H -4.43238200 4.81850500 -0.16040500

H -7.56888100 3.21198000 3.05458300

C -1.66123300 1.27659600 -1.40318300

H -1.96260600 0.40661900 -1.99329200

C -1.08060300 -0.06946000 1.08155000

H -0.32962900 -0.58163100 1.68295900

P -0.20670000 0.64058400 -0.39266600

H -1.28635800 2.02526200 -2.10590700

H -1.46418300 0.78947200 1.64087200

C 0.62087900 -1.07446800 -5.22885600

H 0.34819300 -1.78039700 -6.02266300

H 0.30036300 -0.07715800 -5.56326000

C 2.13097800 -1.08488500 -4.94262000

C -0.05430900 -1.40053500 -3.89488500

H -1.15072100 -1.34732000 -3.97518200

H 0.19142300 -2.42566800 -3.59361400

C 0.51117900 -0.64736400 -1.46430300

C 1.08388500 -1.83051200 -0.99339600

C 0.46462100 -0.39469400 -2.87492900

H 0.14245000 0.61034100 -3.15606700

H 1.19924700 -2.63932600 -1.70146800

C 1.54419100 -2.13467000 0.33779000

O 1.58778100 -1.37490100 1.31100700

O 1.97173100 -3.42556800 0.42506800

C 2.59424500 -3.79721700 1.65757300

H 1.95414900 -3.55804700 2.51157600

H 2.75558700 -4.87450300 1.59075700

H 3.55449700 -3.28614900 1.77629200

C 2.42892600 -0.50856000 -3.55887500

H 2.58719000 0.56740300 -3.51590300

C 3.23069800 -1.26756300 -2.69226200

H 3.40620300 -2.32230900 -2.87620200

C 3.89578800 -0.67342000 -1.57335900

O 3.82448700 0.50838100 -1.21862000

O 4.68244300 -1.57182900 -0.88465400

C 5.36395200 -1.04646100 0.25560600

H 4.63537800 -0.73623200 1.01377700

H 5.92097700 -0.14681400 -0.03698500

C 6.29135500 -2.10260200 0.81118000

C 6.61202900 -2.09243000 2.17506100

C 6.87877300 -3.07112000 -0.01255100

C 7.50841000 -3.02206700 2.70548900

H 6.15546600 -1.35016800 2.82695200

C 7.76953100 -4.00552800 0.51775100

H 6.62034000 -3.09300200 -1.06604600

C 8.09035600 -3.98337200 1.87701300

H 7.74519500 -2.99947100 3.76628800

H 8.21406900 -4.75426300 -0.13329900

H 8.78440900 -4.71203100 2.28790400

H 2.49101800 -2.12098300 -4.97809100

H 2.68696200 -0.53408200 -5.71237400

H -2.55106300 3.82625200 -1.39955000

H -1.05778600 -2.76425000 1.13778700

C 0.84726800 2.12799200 0.14248300

C -0.04086400 3.17998100 0.83879800

H 0.60477200 4.01439000 1.13935900

H -0.82133500 3.58610600 0.19236900

H -0.51469000 2.79739000 1.74897000

C 1.49335600 2.73497300 -1.12053700

H 0.75023700 3.06984600 -1.85491500

H 2.06989700 3.61836100 -0.81879900

H 2.18914000 2.02976100 -1.58163900

C 1.94781600 1.67924000 1.12343300

H 2.64648100 0.98736400 0.65468400

H 2.50554200 2.57584300 1.42478700

H 1.53649500 1.21547900 2.02409000

**TS4e**

C 1.66488700 -1.16011600 5.29394600

H 1.21172900 -0.25528600 5.72325700

H 1.79857300 -1.87220700 6.11747100

C 2.99891000 -0.81394800 4.60948600

H 3.59969200 -1.72738300 4.51428100

H 3.58643900 -0.11235500 5.21595000

C 0.75505100 -1.70535000 4.18767200

H 1.14956700 -2.66239800 3.82780000

H -0.26280400 -1.89678000 4.55677600

C 0.49367700 -0.95098400 1.70399700

C 1.06912900 -2.08305400 1.13319100

C 0.74311000 -0.65661300 3.08489700

C 1.16067100 -2.52989100 -0.23815900

O 0.87886700 -1.93326600 -1.27659500

O 1.65554800 -3.80664900 -0.28299800

C 1.87678200 -4.33107400 -1.59659400

H 0.96285600 -4.28781000 -2.19516000

H 2.66423500 -3.77218100 -2.11131900

H 2.18933600 -5.36653300 -1.44770700

C 2.75118500 -0.24243600 3.21463900

H 2.59236900 0.83326700 3.16532200

C 3.45771200 -0.80125800 2.13495100

C 3.45140600 -0.18284500 0.85269100

O 2.81927600 0.84208700 0.53514000

O 4.24192200 -0.83792600 -0.06281400

C 4.17261400 -0.38019600 -1.41160700

H 3.12973600 -0.41509000 -1.74990900

C -2.33481700 -1.26939900 -0.54594000

C -2.23753600 -0.71192300 -1.81854300

C -2.14405300 -1.55650100 -2.97305300

C -2.20620300 -2.97510600 -2.79595600

C -2.38280900 -3.49860200 -1.49313700

C -2.44832100 -2.68914800 -0.37594000

H -1.88877100 0.02116900 -4.43952000

C -1.96008700 -1.05041500 -4.29021800

C -2.10957500 -3.82573600 -3.93070700

H -2.48506900 -4.57419700 -1.37288600

C -1.93824700 -3.30282700 -5.19180300

C -1.85703900 -1.90067900 -5.36893400

H -2.16878000 -4.90124700 -3.78056100

H -1.85972200 -3.96195800 -6.05211000

H -1.70904100 -1.49231000 -6.36495900

C -2.24211000 0.78143900 -1.99096600

C -3.40678200 1.39929000 -2.55994000

C -1.15128300 1.55198800 -1.58154700

C -4.58105700 0.67315500 -2.91036300

C -3.40571500 2.81524700 -2.76164800

C -1.14356800 2.97148700 -1.82292500

C -5.67935000 1.31296400 -3.44136200

H -4.60814400 -0.39858200 -2.75037600

C -4.55024100 3.44515500 -3.32279400

C -2.25505000 3.55458300 -2.40475100

C -5.66546400 2.71243300 -3.65679800

H -6.56538600 0.73840700 -3.69769400

H -4.52753300 4.52136500 -3.47741300

H -2.24181400 4.62112700 -2.61298200

H -6.53698300 3.20316800 -4.08150700

C 0.01429600 3.86344000 -1.51795000

C 1.31243900 3.57583000 -1.96636700

C -0.20196100 5.07387600 -0.84066400

C 2.37929000 4.44959000 -1.72873200

H 1.49644100 2.66338800 -2.52753500

C 0.83569800 5.98321100 -0.61076400

H -1.20130800 5.30442400 -0.47956600

C 2.12186700 5.64936200 -1.05302600

H 2.94224200 6.33919900 -0.87042800

C -2.68767000 -3.33462000 0.94959700

C -3.82543500 -3.02011300 1.70917500

C -1.81797800 -4.32679800 1.42357700

C -4.09993600 -3.66992100 2.91762700

H -4.52526100 -2.27418300 1.33705000

C -2.06630400 -4.99999100 2.62598800

H -0.92149700 -4.55980700 0.85490400

C -3.21005500 -4.65819000 3.35871600

H -3.41450600 -5.17397600 4.29419700

C -0.01007700 0.86201600 -0.86295700

H 0.31180300 -0.05073000 -1.37040400

C -2.22962700 -0.34658900 0.64857000

H -2.48701300 -0.84828100 1.58237200

P -0.49400700 0.30168500 0.83386800

C -0.60064500 1.80559900 1.86610600

C -1.77513500 2.15799100 2.54775500

C 0.54116200 2.61742100 1.98275500

C -1.81125500 3.31089100 3.33414400

H -2.66324200 1.53817400 2.48165800

C 0.49731900 3.76238000 2.77786000

H 1.46192100 2.33168600 1.48105900

C -0.67527000 4.11296600 3.45111100

H -0.70294200 5.00700100 4.06837600

H 0.87022900 1.48558100 -0.71988500

H -2.88058900 0.52479700 0.52753800

C 5.03895900 -1.26234300 -2.28303100

C 5.43086900 -2.54483000 -1.88172900

C 5.43113900 -0.80222000 -3.54696800

C 6.19721600 -3.34882400 -2.72958400

H 5.14211400 -2.89954200 -0.89833400

C 6.19101100 -1.60619500 -4.39659500

H 5.14050200 0.19637500 -3.86843400

C 6.57835700 -2.88522000 -3.98954800

H 6.49937200 -4.34014000 -2.40054900

H 6.48690300 -1.23115200 -5.37318200

H 7.17547200 -3.51151400 -4.64729900

H 4.50235900 0.66390200 -1.47565900

H 0.29596500 0.26290700 3.45496300

H 1.48118900 -2.79928500 1.83160700

H 3.99068100 -1.74005200 2.24126000

H 1.38632000 4.37932700 2.87085000

H -2.72559900 3.57611800 3.85758800

C 3.78807300 4.08064700 -2.17665000

H 3.70460400 3.16242900 -2.77409400

C 4.42671700 5.15641200 -3.07384600

H 4.57759800 6.09658400 -2.53004900

H 5.40833000 4.82306700 -3.43103600

H 3.80080200 5.37174700 -3.94722000

C 4.68001400 3.75837300 -0.96088800

H 4.24580900 2.95053200 -0.36247500

H 5.68146500 3.45089800 -1.28676900

H 4.79539700 4.63844300 -0.31588500

C 0.55345000 7.31296900 0.07933800

H -0.49707400 7.29230300 0.40021300

C 0.71351100 8.49442400 -0.89793900

H 0.44690000 9.44026200 -0.41073700

H 1.74871500 8.57900700 -1.24955900

H 0.07233500 8.36992200 -1.77745800

C 1.41371600 7.52404200 1.33870900

H 2.48119700 7.57238900 1.09358900

H 1.14602000 8.46461700 1.83500100

H 1.27130600 6.70797700 2.05514500

C -1.11182500 -6.08671300 3.10692800

H -0.26604500 -6.10293700 2.40674800

C -1.77431700 -7.47764900 3.06363700

H -1.05919500 -8.25620100 3.35560200

H -2.62732200 -7.53271200 3.75094300

H -2.14111300 -7.71100900 2.05796200

C -0.54322900 -5.79048300 4.50699100

H -0.05335700 -4.81134900 4.53613600

H -1.33096200 -5.79413500 5.27002400

H 0.19431200 -6.55078300 4.79043800

C -5.34677500 -3.31280600 3.71826100

H -5.86707200 -2.51766900 3.16648300

C -6.31790400 -4.50332800 3.83231900

H -5.86704700 -5.33268100 4.39016400

H -7.23319400 -4.20699300 4.35889700

H -6.59943400 -4.88071400 2.84320600

C -4.99511300 -2.75231000 5.10969200

H -4.49050200 -3.50507100 5.72685300

H -4.32917000 -1.88569400 5.03166500

H -5.90213700 -2.44048500 5.64141000

**TS7e**

C -3.11138200 -1.21246400 0.35673600

C -3.77401200 -0.26925500 -0.42632200

C -4.75076900 -0.68882800 -1.39028600

C -5.06381100 -2.08276400 -1.49309900

C -4.42873400 -2.99907100 -0.61899300

C -3.47582100 -2.60038700 0.29816500

H -5.16427800 1.27164000 -2.22253600

C -5.40136700 0.21489700 -2.27717100

C -6.01919600 -2.51383300 -2.45337300

H -4.71913600 -4.04587900 -0.66050800

C -6.63306600 -1.61263600 -3.29249600

C -6.31515500 -0.23584200 -3.20425300

H -6.25190200 -3.57424200 -2.51475800

H -7.35841500 -1.95390500 -4.02594600

H -6.79571900 0.47094100 -3.87519700

C -3.46983900 1.19499100 -0.26102700

C -4.45248600 2.04109500 0.35408500

C -2.24277700 1.71623500 -0.67394300

C -5.68468800 1.55196800 0.87401200

C -4.17327800 3.43884600 0.48051700

C -1.98693700 3.12816200 -0.58944800

C -6.58969000 2.40167100 1.47100300

H -5.90646500 0.49310300 0.80353400

C -5.13312600 4.29164100 1.09154800

C -2.95034700 3.94189700 -0.02089000

C -6.31699000 3.78709000 1.57742300

H -7.52158400 2.00539500 1.86504400

H -4.90887900 5.35265100 1.17174800

H -2.77318600 5.01308900 0.02606200

H -7.04212900 4.44645700 2.04646900

C -0.75465900 3.77791300 -1.12546200

C -0.32660000 3.56552000 -2.44554600

C -0.04098400 4.68870900 -0.33266700

C 0.79826400 4.21464000 -2.96520100

H -0.89110800 2.89741600 -3.09186800

C 1.07033100 5.37862700 -0.82999700

H -0.35215100 4.85064500 0.69647200

C 1.47966100 5.12278000 -2.14438100

H 2.34959900 5.64109400 -2.54020600

C -2.89951800 -3.60984900 1.23574000

C -3.04209600 -3.45253400 2.62269900

C -2.23255900 -4.74409500 0.75510400

C -2.51562100 -4.38488400 3.52188600

H -3.57896300 -2.58800400 3.00770000

C -1.69137400 -5.69620200 1.62774600

H -2.10684800 -4.87053400 -0.31645300

C -1.84372400 -5.50049200 3.00577000

H -1.42131600 -6.22891300 3.69389200

C -1.19461100 0.74702300 -1.18113200

H -1.64531000 0.07876700 -1.91637200

C -1.95474900 -0.75340700 1.21454400

H -1.61036400 -1.50506700 1.92208800

P -0.48827000 -0.32022900 0.16704000

C 0.60445500 0.78459300 1.13258700

C 0.32091100 1.09969500 2.47137500

C 1.72545500 1.34726900 0.50960300

C 1.14065900 1.98252600 3.17080800

H -0.51541400 0.64119300 2.98477600

C 2.54860000 2.22054500 1.22457400

H 1.98477300 1.09984300 -0.51734300

C 2.25899100 2.54416800 2.54961500

H 2.90288700 3.22489700 3.10055400

H -0.34648200 1.24217600 -1.65506800

H -2.21515700 0.15547800 1.76520300

C -0.13586300 -3.64351600 -3.91424400

H -0.33404700 -4.65855800 -4.27949300

H -0.73519900 -2.95758000 -4.53015300

C 1.34786600 -3.27414200 -3.99912500

C -0.51007500 -3.46792300 -2.44124900

H -1.59283100 -3.57786900 -2.28551900

H -0.01598800 -4.23779000 -1.83649000

C 0.23757100 -1.80697900 -0.63018600

C 1.12144800 -2.60869100 0.07703300

C -0.04677500 -2.07656100 -2.01212500

H -0.62979700 -1.32029400 -2.52896800

H 1.63516600 -3.39633600 -0.45960400

C 1.30765000 -2.57199700 1.50431600

O 0.67836200 -1.86516100 2.29821200

O 2.26432200 -3.44300100 1.92449900

C 2.50341200 -3.46302100 3.33414700

H 1.60837200 -3.78196500 3.87715000

H 3.31307900 -4.17901300 3.48371600

H 2.79960000 -2.47308700 3.69318300

C 1.63510000 -2.03542600 -3.15486000

H 1.36000700 -1.08903000 -3.61888600

C 2.85667000 -1.98805000 -2.45720800

H 3.42676100 -2.88729200 -2.24896400

C 3.39974300 -0.73702300 -2.05896700

O 2.85322500 0.37252400 -2.18961000

O 4.65780500 -0.85712800 -1.50169000

C 5.30819000 0.34527900 -1.09780500

H 5.07577500 0.54612200 -0.04238900

H 4.90470800 1.17977600 -1.68295600

C 6.80238300 0.21547700 -1.28895400

C 7.67775800 0.89674300 -0.43482200

C 7.33688200 -0.53774800 -2.34141400

C 9.05844600 0.84026900 -0.63310800

H 7.27588400 1.47403100 0.39579800

C 8.71692900 -0.60107600 -2.53744300

H 6.66305500 -1.08411200 -2.99356800

C 9.58322700 0.08988200 -1.68687300

H 9.72336800 1.37446500 0.04106400

H 9.11717300 -1.19366300 -3.35643700

H 10.65808500 0.03866900 -1.84022700

H 1.95106300 -4.11058200 -3.62127500

H 1.66473000 -3.10094500 -5.03604800

H 3.42022500 2.64284600 0.73293700

H 0.91245000 2.21881900 4.20651700

C 1.26378700 3.92873900 -4.38756100

H 0.55382000 3.20947800 -4.81864100

C 1.23398000 5.19014700 -5.27105000

H 1.93536100 5.94995800 -4.90580300

H 1.52004800 4.94407900 -6.30058900

H 0.23489600 5.64020200 -5.29207200

C 2.65635300 3.26706400 -4.40243800

H 3.41949100 3.95045300 -4.00926300

H 2.67690400 2.35442600 -3.79741700

H 2.94331700 3.00864700 -5.42924800

C 1.78912600 6.40345800 0.04033400

H 1.35659300 6.32921400 1.04747500

C 1.53495800 7.83713600 -0.46652300

H 2.00511400 8.57295700 0.19723200

H 1.94973100 7.97926300 -1.47176900

H 0.46253700 8.05600500 -0.51503300

C 3.29765800 6.12597700 0.16718200

H 3.76468800 6.84706000 0.84871500

H 3.48130100 5.11908700 0.55601300

H 3.80690200 6.20937500 -0.80010900

C -2.63937000 -4.15651100 5.02301700

H -3.26290000 -3.26266500 5.16194300

C -3.33980400 -5.32260900 5.74377100

H -3.46041400 -5.09923200 6.81053700

H -4.33235100 -5.51230200 5.32042100

H -2.75973000 -6.24959600 5.66376700

C -1.26284400 -3.85833500 5.65061300

H -1.36696100 -3.59479400 6.71024600

H -0.60501800 -4.73412700 5.58873700

H -0.76535400 -3.03033600 5.13344000

C -0.93461000 -6.90017900 1.07971700

H -0.90965800 -6.79297100 -0.01322500

C -1.65634200 -8.22368300 1.39770300

H -1.12525300 -9.07119600 0.94799100

H -1.70663500 -8.39852500 2.47922500

H -2.68203400 -8.21988900 1.01229500

C 0.52502700 -6.93282200 1.57325000

H 1.04470400 -5.99385600 1.35459600

H 0.57288100 -7.09556400 2.65701900

H 1.07447700 -7.75137100 1.09287700

**TS4f**

C 1.41113300 -1.24710800 5.30512500

H 0.97635900 -0.33487800 5.73814700

H 1.50816700 -1.97360500 6.12129300

C 2.76749100 -0.92891200 4.65222800

H 3.33849700 -1.85954800 4.54027100

H 3.36803900 -0.26500700 5.28807500

C 0.50759400 -1.75069000 4.17405900

H 0.87534100 -2.71704000 3.81184100

H -0.52317400 -1.91261600 4.52027300

C 0.34142700 -0.96835500 1.69050900

C 0.93759600 -2.09533700 1.12875800

C 0.55035100 -0.69116600 3.08161600

C 1.08960400 -2.54042100 -0.23736400

O 0.84114500 -1.95503800 -1.29172700

O 1.61042900 -3.80764700 -0.25722100

C 1.93605100 -4.31331300 -1.55467800

H 1.05797200 -4.31877600 -2.20687800

H 2.71900800 -3.70781000 -2.02198200

H 2.29746800 -5.33011400 -1.38978300

C 2.55900600 -0.31164200 3.27167200

H 2.40650700 0.76648900 3.25234200

C 3.28655500 -0.83771800 2.18916000

C 3.28482400 -0.18096600 0.92530300

O 2.63759900 0.84087700 0.63602600

O 4.08649600 -0.79852800 -0.00781800

C 4.03497600 -0.26527400 -1.33338800

H 3.04883100 -0.46101600 -1.76838400

C -2.44126100 -1.27967600 -0.58709100

C -2.33530700 -0.73452400 -1.86376400

C -2.24185100 -1.58966300 -3.01074800

C -2.30588600 -3.00672000 -2.82076400

C -2.48249000 -3.51873000 -1.51309000

C -2.55226700 -2.69802100 -0.40488000

H -1.98438600 -0.02607700 -4.49186200

C -2.05631700 -1.09614800 -4.33250500

C -2.20864700 -3.86786800 -3.94736000

H -2.58023600 -4.59365400 -1.38291300

C -2.03660700 -3.35691000 -5.21325400

C -1.95435200 -1.95653900 -5.40331900

H -2.26835300 -4.94186900 -3.78721100

H -1.95855100 -4.02411600 -6.06735500

H -1.80674200 -1.55747400 -6.40321000

C -2.31554600 0.75722100 -2.04762200

C -3.46494200 1.39685800 -2.62344200

C -1.20857200 1.50782200 -1.64644900

C -4.65537000 0.69346100 -2.96474700

C -3.43325600 2.81122300 -2.83778700

C -1.15919600 2.91752600 -1.92936200

C -5.74224700 1.35284500 -3.49546900

H -4.70373600 -0.37642100 -2.79665700

C -4.56692800 3.46122400 -3.39739300

C -2.25857000 3.52569900 -2.50618000

C -5.69958600 2.75017500 -3.72014200

H -6.64135400 0.79560200 -3.74436100

H -4.52160900 4.53525600 -3.56186700

H -2.21409300 4.58640900 -2.73978200

H -6.56240400 3.25655400 -4.14419500

C 0.05313500 3.75731600 -1.70137200

C 1.30013400 3.39680900 -2.24011400

C -0.04931200 4.98455400 -1.02604500

C 2.40934100 4.23026100 -2.09918100

H 1.39573000 2.46681100 -2.79321600

C 1.05782100 5.82361900 -0.89302400

H -1.00391700 5.27401100 -0.59543700

C 2.29279900 5.44790400 -1.42566600

H 3.36313700 3.93114500 -2.52502100

H 0.95510500 6.77171300 -0.37169800

H 3.15586600 6.09960500 -1.32027500

C -2.78501600 -3.32685500 0.92982500

C -3.93696500 -3.02874900 1.67748600

C -1.88736100 -4.28096700 1.43504900

C -4.18023300 -3.65846400 2.89874300

H -4.65764800 -2.31471500 1.28576500

C -2.13279600 -4.91255100 2.65539800

H -0.98285300 -4.50706700 0.87827000

C -3.27749600 -4.60274300 3.39236400

H -5.08073100 -3.41925900 3.45863400

H -1.42308500 -5.64393400 3.03296400

H -3.46636000 -5.09463300 4.34281700

C -0.09285400 0.81949400 -0.89015300

H 0.22843600 -0.11065800 -1.36735200

C -2.34982400 -0.34372500 0.59678800

H -2.62547700 -0.83157400 1.53269000

P -0.61375100 0.29876400 0.80884700

C -0.74865000 1.80523100 1.83498100

C -1.93558000 2.12375500 2.51258500

C 0.36682400 2.64615700 1.95308400

C -2.02207700 3.27368600 3.30336200

H -2.80551400 1.47831900 2.44068100

C 0.30696100 3.80081900 2.74660400

H 1.29613300 2.37827100 1.45692000

C -0.89119700 4.09547500 3.40744100

H -0.95022700 4.98849100 4.02349600

H 0.79452400 1.43331800 -0.74429800

H -2.99437800 0.52904300 0.45486100

C 5.11938700 -0.90961900 -2.16471100

C 6.36745100 -1.22833300 -1.61369700

C 4.89951200 -1.15792700 -3.52510500

C 7.37317800 -1.77820600 -2.40890000

H 6.53542700 -1.05676400 -0.55516700

C 5.90780700 -1.70079200 -4.32463700

H 3.92934700 -0.92815200 -3.96085000

C 7.14900700 -2.01314400 -3.76801000

H 8.33496300 -2.02539300 -1.96617200

H 5.71911900 -1.88829000 -5.37877200

H 7.93372600 -2.44186700 -4.38606000

H 4.16440100 0.82395700 -1.29062500

H 0.10865300 0.23460100 3.44362800

H 1.33156800 -2.81095000 1.83828600

H 3.82267900 -1.77682900 2.27392500

C 1.55661900 4.66149200 2.90314600

H 2.15793500 4.50966200 1.99799900

C 1.26351300 6.16636100 3.01526500

H 0.75737900 6.41760700 3.95518100

H 2.20133000 6.73302200 2.99403600

H 0.63639000 6.51608300 2.18816500

C 2.39682200 4.18062600 4.10477500

H 2.67524000 3.12683900 3.99904300

H 3.31868000 4.76823800 4.19070500

H 1.83652600 4.28994700 5.04173900

C -3.31884800 3.61597100 4.02743400

H -4.03339300 2.81123800 3.80638800

C -3.13196400 3.65791700 5.55604300

H -2.44098100 4.45606200 5.85237800

H -4.08965400 3.84609400 6.05555500

H -2.73015700 2.71110300 5.93302000

C -3.93110800 4.93080500 3.50731900

H -4.10381100 4.88751800 2.42637100

H -4.89052100 5.13084200 3.99904500

H -3.27076300 5.78318700 3.70683500

**TS7f**

C -2.94120900 -1.14414500 0.47137500

C -3.79935700 -0.24687700 -0.16140900

C -4.87876000 -0.72236600 -0.97959500

C -5.08185600 -2.13449500 -1.09775500

C -4.23000500 -3.01310100 -0.38634100

C -3.18246900 -2.55771900 0.39124100

H -5.59187100 1.21590600 -1.64779300

C -5.74103700 0.14387500 -1.71004000

C -6.14010500 -2.62356900 -1.91116900

H -4.42424100 -4.08138100 -0.43853800

C -6.96045100 -1.75790500 -2.59692100

C -6.75251300 -0.36103500 -2.49716200

H -6.28533300 -3.69873400 -1.98370000

H -7.76450400 -2.14178900 -3.21871000

H -7.39622400 0.31886100 -3.04858800

C -3.57799400 1.23300600 -0.02559000

C -4.49868000 2.02322700 0.73941000

C -2.47913400 1.82877100 -0.64399200

C -5.58613600 1.45969700 1.46560900

C -4.30013500 3.43958800 0.80775600

C -2.32872000 3.25623100 -0.63483600

C -6.43100600 2.25565500 2.20723500

H -5.74081500 0.38673600 1.43768400

C -5.19611800 4.23429300 1.57353200

C -3.22753600 4.01781500 0.08729400

C -6.23979800 3.65775500 2.26000300

H -7.25132500 1.80410300 2.75844900

H -5.03716400 5.30942800 1.60865500

H -3.12560600 5.10008100 0.08471200

H -6.91665700 4.27371800 2.84563500

C -1.28506300 3.96426600 -1.43360000

C -1.22443200 3.81337000 -2.83008600

C -0.39936900 4.86235000 -0.82008600

C -0.30168400 4.53453300 -3.58758000

H -1.92297200 3.14390400 -3.32591100

C 0.52038200 5.58830300 -1.57846000

H -0.42003900 4.97388300 0.25968800

C 0.57406300 5.42585300 -2.96386700

H -0.27369500 4.40691600 -4.66638800

H 1.20050200 6.27593100 -1.08354500

H 1.29225800 5.98890200 -3.55343100

C -2.38351500 -3.56256100 1.15423600

C -2.31669000 -3.51981200 2.55686600

C -1.74432400 -4.61919300 0.48725800

C -1.62321400 -4.49850300 3.26915500

H -2.82854100 -2.72501700 3.09336200

C -1.05026300 -5.59816800 1.19902500

H -1.77996900 -4.66017000 -0.59746200

C -0.98574600 -5.54054600 2.59220700

H -1.58910600 -4.45066000 4.35436700

H -0.55325400 -6.40222500 0.66307300

H -0.44374000 -6.30192600 3.14636700

C -1.43323200 0.93239500 -1.26989400

H -1.90518200 0.16456700 -1.88648400

C -1.72934300 -0.60486700 1.19692000

H -1.22376300 -1.34645300 1.81062900

P -0.43184600 0.04123000 0.02930800

C 0.55750400 1.33476700 0.85946200

C 0.04649700 2.03715000 1.96063600

C 1.82942000 1.64077900 0.36383300

C 0.79797500 3.04043500 2.57986100

H -0.93407200 1.81040900 2.36517200

C 2.60021900 2.64575700 0.96714600

H 2.25097300 1.09447500 -0.47681600

C 2.06906100 3.32999400 2.06657800

H 2.66671400 4.10302300 2.54431700

H -0.73052200 1.48752000 -1.89352300

H -2.00973900 0.22556200 1.84935900

C 0.14526200 -2.81817000 -4.28675100

H -0.08609400 -3.75486200 -4.80825600

H -0.41879500 -2.02199600 -4.79352000

C 1.64942400 -2.51021600 -4.30118300

C -0.24947600 -2.85072800 -2.80962100

H -1.33462500 -2.98311200 -2.68377900

H 0.23822800 -3.69574100 -2.30756600

C 0.40736300 -1.35622700 -0.80164900

C 1.20878900 -2.22779700 -0.07317800

C 0.21142500 -1.52756300 -2.21053700

H -0.28363700 -0.70342100 -2.71956600

H 1.63028400 -3.08537900 -0.57811600

C 1.48218400 -2.10796700 1.33492500

O 0.99751100 -1.26048000 2.09412700

O 2.35289600 -3.04960000 1.77905900

C 2.66467900 -2.98923000 3.17211700

H 1.76953800 -3.16113700 3.77859900

H 3.39685600 -3.77999400 3.34341300

H 3.08703700 -2.01561900 3.43754500

C 2.04010800 -1.54393400 -3.17748100

H 2.01081300 -0.48440900 -3.42627200

C 3.11714500 -1.92160000 -2.36036900

H 3.41754200 -2.96241300 -2.28915500

C 3.92987200 -0.96408700 -1.68056300

O 3.77766800 0.26412200 -1.66404100

O 4.99688400 -1.56040500 -1.03958500

C 5.89397100 -0.68738900 -0.35983300

H 5.42629300 -0.31287600 0.56069900

H 6.09428500 0.18841000 -0.98998800

C 7.17281700 -1.42694600 -0.03965900

C 7.92825900 -1.05569800 1.07940200

C 7.65234500 -2.44868400 -0.86835300

C 9.14326300 -1.68234600 1.36136100

H 7.56125800 -0.27044400 1.73761300

C 8.86340400 -3.08129700 -0.58410400

H 7.06184500 -2.75128800 -1.72695900

C 9.61525700 -2.69926000 0.52925700

H 9.71585900 -1.38191400 2.23536700

H 9.22034600 -3.87635900 -1.23420500

H 10.55814900 -3.19322800 0.74946500

H 2.20254400 -3.44723700 -4.16051600

H 1.96807600 -2.10383000 -5.26973700

C 3.98990100 2.96599000 0.42679000

H 4.20736200 2.22219500 -0.34660600

C 4.03052500 4.35785000 -0.23306400

H 3.82519900 5.15330700 0.49545800

H 5.02047500 4.54918200 -0.66473500

H 3.28853600 4.43509600 -1.03466400

C 5.07380900 2.84392300 1.51435800

H 5.05768600 1.85245700 1.98090300

H 6.06859500 2.99999200 1.08007400

H 4.94221100 3.58872600 2.30936200

C 0.25027400 3.76399600 3.80507700

H -0.76867100 3.38907900 3.97294000

C 0.15037700 5.28597800 3.59318100

H -0.28395800 5.77056200 4.47578000

H 1.13705300 5.73239400 3.42223400

H -0.48123400 5.52696200 2.73052200

C 1.07479600 3.43554400 5.06510500

H 2.10565400 3.79675800 4.96938000

H 0.63485900 3.91051000 5.95032400

H 1.11519900 2.35502900 5.23949100

**TS4g**

C 1.64046300 -1.56363500 5.27859400

H 1.15797700 -0.71660300 5.78704700

H 1.79443600 -2.34383300 6.03421400

C 2.96515000 -1.11271800 4.63817900

H 3.59096500 -1.99521800 4.45269600

H 3.53200100 -0.46162100 5.31673200

C 0.74980500 -2.03283200 4.12275700

H 1.16146900 -2.94788500 3.68305300

H -0.26427200 -2.27831100 4.46927000

C 0.49413800 -1.06775400 1.70983800

C 1.18056900 -2.09173400 1.05811700

C 0.71202300 -0.89044200 3.11679700

C 1.31748100 -2.45441500 -0.33230400

O 0.88977600 -1.89328400 -1.34263400

O 2.04637800 -3.60969500 -0.44154800

C 2.37524200 -4.00301000 -1.77638100

H 1.47645700 -4.10705300 -2.39107400

H 3.04095700 -3.26979900 -2.24272500

H 2.88727000 -4.96234500 -1.68081700

C 2.70143700 -0.40818300 3.30972200

H 2.49333300 0.65864000 3.37295100

C 3.43760400 -0.81603000 2.18240100

C 3.39613200 -0.07034100 0.96811300

O 2.70189400 0.93911800 0.75490500

O 4.21664900 -0.58170800 -0.00999400

C 4.13463200 0.05509200 -1.29035100

H 3.13604400 -0.09196600 -1.71435200

C -2.43290500 -1.31726300 -0.45140400

C -2.31664000 -0.75693100 -1.72137700

C -2.28762200 -1.60294800 -2.88020100

C -2.47901700 -3.01102100 -2.71281100

C -2.72586700 -3.52188600 -1.41560100

C -2.70098400 -2.71959200 -0.29203100

H -1.89156200 -0.04739600 -4.33923700

C -2.05429900 -1.10960600 -4.19424700

C -2.44941200 -3.86217000 -3.85067700

H -2.94817100 -4.58029300 -1.30641200

C -2.22278700 -3.35123500 -5.10778400

C -2.01793900 -1.96074800 -5.27658800

H -2.60419900 -4.92872700 -3.70581000

H -2.19603200 -4.01083400 -5.97084500

H -1.82787800 -1.56201100 -6.26941100

C -2.28573800 0.73566100 -1.90020600

C -3.42308100 1.36300100 -2.51926600

C -1.19840800 1.50333400 -1.47681200

C -4.61291800 0.65761700 -2.86112400

C -3.37904900 2.76779200 -2.78671700

C -1.14621600 2.90680800 -1.79060100

C -5.68216500 1.30394900 -3.44140500

H -4.67683900 -0.40498600 -2.65774900

C -4.49280200 3.40424100 -3.39940200

C -2.21448100 3.49256800 -2.44091500

C -5.62344600 2.69032900 -3.72156800

H -6.58004600 0.74338600 -3.68728600

H -4.43395200 4.47095000 -3.60219100

H -2.16451400 4.54819300 -2.69540500

H -6.47184700 3.18571700 -4.18565000

C -0.06275800 0.83832400 -0.73084200

H 0.28978500 -0.06101100 -1.24684300

C -2.27469500 -0.41913100 0.75314600

H -2.53116500 -0.93531200 1.67950600

P -0.53482800 0.21699900 0.94606000

C -0.67187600 1.63947800 2.08786800

C -1.84419900 1.88239500 2.82185600

C 0.44470300 2.47480000 2.27028100

C -1.89947500 2.94414600 3.72629200

H -2.71436900 1.24411900 2.71285400

C 0.37860100 3.53214300 3.17712200

H 1.36632700 2.26663200 1.73452200

C -0.78870400 3.76903100 3.90562700

H -0.83242800 4.59355700 4.61217300

H 0.80105000 1.48559000 -0.57956900

H -2.91394200 0.46528800 0.66321100

C 5.19006400 -0.53925200 -2.19185900

C 6.49383700 -0.76332100 -1.72839800

C 4.88891000 -0.84003200 -3.52561900

C 7.47289700 -1.27010700 -2.58231300

H 6.72818100 -0.55161800 -0.68944300

C 5.86998200 -1.34142100 -4.38528300

H 3.87656700 -0.68300600 -3.89121500

C 7.16577800 -1.55766600 -3.91556400

H 8.47868000 -1.44270900 -2.20719900

H 5.61797300 -1.57120200 -5.41766300

H 7.93009000 -1.95279100 -4.57986200

H 4.27834700 1.13600100 -1.17125300

H 0.22675600 -0.02408800 3.56046300

H 1.66484600 -2.80312500 1.71207800

H 4.02111300 -1.73041100 2.19375000

H 1.24363300 4.17537300 3.30872800

H -2.81018800 3.11927500 4.29244800

C 1.36010300 2.57724700 -3.28687700

C 1.24334300 3.56783300 -2.25815300

C 2.52583800 2.41166000 -3.98952500

C 0.05392800 3.76151200 -1.51106600

C 2.39050400 4.41136800 -2.01014700

C 3.66444200 3.22263900 -3.71601400

H 2.58365800 1.65629600 -4.76845400

C -0.02829700 4.81702600 -0.56889600

C 2.29849100 5.43446300 -1.06263900

C 3.59202200 4.19587100 -2.75574800

H 4.58254900 3.06723900 -4.27530100

C -1.21125400 5.09282900 0.19191200

C 1.11939500 5.67049900 -0.35069400

H 3.16219100 6.07206200 -0.88530400

H 4.44862400 4.83146500 -2.54453100

C -1.26683900 6.14257000 1.07143800

H -2.07550000 4.45119700 0.06638900

C 1.02244500 6.74776700 0.58560000

C -0.13759700 6.98702600 1.27200400

H -2.17959500 6.32850800 1.63097200

H 1.89500600 7.38015400 0.73207300

H -0.20095400 7.81514400 1.97286600

H 0.50270700 1.95459400 -3.51700200

C -5.22392800 -2.06458400 1.06382800

C -4.28807200 -2.97838400 1.65137700

C -6.39931600 -1.73978800 1.69129000

C -3.05816900 -3.32672400 1.03324600

C -4.64766000 -3.57364200 2.91921700

C -6.73326400 -2.30884100 2.95320400

H -7.08977900 -1.04720900 1.21716000

C -2.21342200 -4.29306400 1.63568900

C -3.79144000 -4.50899000 3.50717900

C -5.88076900 -3.20328600 3.54260400

H -7.66800600 -2.03837200 3.43662400

C -0.97134900 -4.70657300 1.05555200

C -2.59409200 -4.89155400 2.89693800

H -4.06824400 -4.95634700 4.45982600

H -6.12802300 -3.65726000 4.49942800

C -0.17459400 -5.64288300 1.66210400

H -0.65891400 -4.26396700 0.11939400

C -1.73402300 -5.86450900 3.49727400

C -0.55938700 -6.23447500 2.89883100

H 0.76852600 -5.91826000 1.19932000

H -2.03875100 -6.30516200 4.44367800

H 0.08489100 -6.97472600 3.36555700

H -4.99797700 -1.63231900 0.09535200

**TS7g**

C -2.64282700 -1.42127800 0.67188100

C -3.58094900 -0.50118200 0.20557700

C -4.74722000 -0.95377800 -0.50133500

C -4.97633700 -2.36134900 -0.62399000

C -4.06622900 -3.26116500 -0.01759100

C -2.91560900 -2.83167600 0.61409400

H -5.51613900 1.00247800 -1.04294800

C -5.67769900 -0.06734700 -1.11418600

C -6.12057900 -2.82652000 -1.32745300

H -4.28803800 -4.32481400 -0.04852200

C -7.00017100 -1.94158200 -1.90700700

C -6.76993000 -0.54873800 -1.80197400

H -6.28241800 -3.89896800 -1.40420100

H -7.86846400 -2.30703300 -2.44820800

H -7.46144600 0.14708700 -2.26922000

C -3.41097300 0.96855100 0.47060400

C -4.33468300 1.61837600 1.35987100

C -2.38780900 1.69715600 -0.13709400

C -5.32249900 0.91568300 2.10756800

C -4.24769200 3.03664500 1.52883900

C -2.34407000 3.12847400 -0.01127600

C -6.17411000 1.58129500 2.96158800

H -5.39936400 -0.16079700 2.00579200

C -5.15023300 3.69681500 2.40672500

C -3.27007800 3.75482000 0.80050500

C -6.09460700 2.98688900 3.11100300

H -6.91445600 1.02184400 3.52692200

H -5.07317900 4.77597900 2.51497300

H -3.25122300 4.83825100 0.88461900

H -6.77654400 3.49903600 3.78410700

C -1.36603700 0.95749100 -0.97095800

H -1.87648300 0.29474500 -1.67409700

C -1.34919000 -0.91545700 1.26345800

H -0.77017000 -1.69348200 1.75515100

P -0.21960300 -0.13912900 0.00657100

C 0.94712500 0.95289900 0.88994700

C 0.94900900 1.07063300 2.28920500

C 1.89640200 1.65030100 0.13361000

C 1.88511200 1.88950200 2.91716600

H 0.25734300 0.49963500 2.89718500

C 2.83689800 2.46097000 0.77117000

H 1.96810900 1.52756100 -0.94233900

C 2.83101700 2.58551200 2.15986500

H 3.56720300 3.21352100 2.65479400

H -0.74618600 1.64230200 -1.55234600

H -1.54947100 -0.12365900 1.99279600

C -0.03092700 -2.07622400 -4.87708800

H -0.29029300 -2.91085500 -5.54007100

H -0.60470100 -1.20292300 -5.21952600

C 1.47012300 -1.77154400 -4.89089400

C -0.36424200 -2.36327000 -3.41238600

H -1.44675300 -2.49595700 -3.26503800

H 0.11808000 -3.29465800 -3.09357900

C 0.47858100 -1.32607700 -1.19955500

C 1.39740600 -2.28328100 -0.78583200

C 0.15498200 -1.18375900 -2.59414500

H -0.39868100 -0.28587900 -2.85810300

H 1.78086200 -2.97812200 -1.51911200

C 1.82636500 -2.48733100 0.57356700

O 1.41588100 -1.86436700 1.55932500

O 2.76199200 -3.46226300 0.68434100

C 3.24672700 -3.70162100 2.00759800

H 2.43685300 -4.03533100 2.66403600

H 4.00112700 -4.48341500 1.90586000

H 3.69280000 -2.79643800 2.42987700

C 1.86780400 -0.91639500 -3.68636900

H 1.72399000 0.15612900 -3.81257400

C 3.06029600 -1.25462400 -3.02119800

H 3.47510300 -2.25492600 -3.09056800

C 3.83674900 -0.26352400 -2.34426000

O 3.56880900 0.93802300 -2.22540600

O 5.00915400 -0.78001500 -1.82909100

C 5.85203800 0.15175100 -1.15612000

H 5.38634500 0.46525200 -0.21246200

H 5.95401200 1.05650500 -1.76934100

C 7.19902500 -0.48371600 -0.89922200

C 7.96290800 -0.07211000 0.19990100

C 7.72992400 -1.44737100 -1.76545400

C 9.23466000 -0.60133100 0.42568900

H 7.55682800 0.66720900 0.88770900

C 8.99836600 -1.98306000 -1.53757800

H 7.13422700 -1.78230700 -2.60824700

C 9.75728200 -1.56063900 -0.44377100

H 9.81256600 -0.27076700 1.28527500

H 9.39463400 -2.73434600 -2.21640400

H 10.74486000 -1.97921400 -0.26739000

H 2.02631000 -2.71747800 -4.85043400

H 1.77468400 -1.27007900 -5.81883700

H 3.57813300 2.97514100 0.16763900

H 1.88611000 1.96722600 4.00110600

C -2.55855400 3.41950700 -2.93122900

C -1.53942100 4.10726000 -2.19377500

C -2.66372300 3.54313100 -4.29281900

C -1.39267800 3.98997700 -0.78733500

C -0.65013800 4.97171200 -2.93672600

C -1.76235500 4.37003000 -5.02172700

H -3.44821800 3.01023600 -4.82350300

C -0.41341500 4.75907100 -0.10962800

C 0.31535500 5.71519200 -2.25267200

C -0.78959100 5.06681000 -4.35699800

H -1.85749200 4.45096200 -6.10090300

C -0.22814700 4.70832100 1.31009000

C 0.44594900 5.64575600 -0.86332000

H 0.97523400 6.37228600 -2.81519900

H -0.10204400 5.71266200 -4.89770200

C 0.70273200 5.49515600 1.93680500

H -0.83323000 4.02488400 1.89377900

C 1.41078500 6.44256300 -0.17060700

C 1.53179800 6.38069800 1.19126700

H 0.81985700 5.43407500 3.01520200

H 2.04468100 7.10489400 -0.75530100

H 2.26384000 6.99653800 1.70660800

H -3.26693600 2.79765000 -2.39480500

C -2.77932500 -2.98160400 3.54213700

C -2.02058600 -3.88078800 2.72352300

C -2.71838400 -3.03407600 4.91103300

C -2.05468300 -3.84822900 1.30496800

C -1.21190300 -4.87125500 3.39769300

C -1.89714800 -3.99404700 5.56755600

H -3.30880200 -2.34059600 5.50400200

C -1.34320600 -4.81890600 0.55713700

C -0.50151900 -5.80878300 2.64236700

C -1.17083200 -4.88745600 4.82721300

H -1.85864600 -4.01874800 6.65306900

C -1.36250800 -4.86602900 -0.87427000

C -0.55775400 -5.81958500 1.24627400

H 0.09677100 -6.55932100 3.15508100

H -0.54946700 -5.63540900 5.31409400

C -0.68630700 -5.83705300 -1.56654000

H -1.92402600 -4.11344700 -1.41397300

C 0.14151000 -6.80844200 0.48521500

C 0.07613500 -6.82552100 -0.88146200

H -0.72480400 -5.84957200 -2.65240000

H 0.72593800 -7.55133200 1.02268200

H 0.60868400 -7.58397100 -1.44841800

H -3.42365700 -2.25208200 3.06415400

**CP3-cis**

C -3.14028300 1.41123500 -1.22360100

C -4.22388600 0.53384800 -1.22306700

C -5.56500200 1.03900100 -1.31132200

C -5.76031900 2.44770400 -1.47468500

C -4.63042200 3.29980100 -1.51170100

C -3.34404900 2.82303200 -1.36445500

H -6.59377800 -0.85874700 -1.07341600

C -6.71836200 0.20780900 -1.22248000

C -7.08076000 2.96445600 -1.57551200

H -4.78668900 4.36903100 -1.62870700

C -8.17233700 2.13108000 -1.50011800

C -7.98529900 0.74005000 -1.31400500

H -7.20985500 4.03644300 -1.70307600

H -9.17763700 2.53662700 -1.57336200

H -8.85029300 0.08670800 -1.23869700

C -4.00537700 -0.94682900 -1.09982500

C -4.31029600 -1.80865100 -2.20655000

C -3.51374100 -1.48077000 0.09244400

C -4.71580800 -1.31739300 -3.48003100

C -4.16804900 -3.22377100 -2.04138600

C -3.42733100 -2.90311900 0.27063600

C -4.97684600 -2.17963400 -4.52187800

H -4.81072000 -0.24748400 -3.62753600

C -4.45795200 -4.08768300 -3.13251600

C -3.75479100 -3.73053500 -0.78612900

C -4.85409400 -3.57957400 -4.34793400

H -5.27911300 -1.78244400 -5.48696800

H -4.35432600 -5.16036500 -2.98796500

H -3.70593200 -4.80784600 -0.64966200

H -5.06884100 -4.24798400 -5.17713000

C -3.04165500 -3.53012500 1.56980800

C -3.77808300 -3.28038700 2.74094300

C -1.97200500 -4.43641400 1.63407300

C -3.44738900 -3.91023600 3.94088400

H -4.62807300 -2.60369100 2.70424600

C -1.64170200 -5.06756200 2.83430300

H -1.38885200 -4.63123100 0.73898100

C -2.37647700 -4.80538500 3.99185000

H -4.03219700 -3.70694900 4.83388400

H -0.80441100 -5.75972300 2.86503000

H -2.11844400 -5.29555400 4.92663100

C -2.21526600 3.80765700 -1.36108100

C -1.72787900 4.33744500 -2.56682000

C -1.67315700 4.26106500 -0.14944200

C -0.71441300 5.29758000 -2.55934900

H -2.14862500 3.99723300 -3.51006600

C -0.66130000 5.22332100 -0.14351700

H -2.03853100 3.86768000 0.79307800

C -0.17981000 5.74274400 -1.34694700

H -0.34594500 5.69992700 -3.49969700

H -0.26236200 5.54853500 0.81260100

H 0.60688900 6.49302400 -1.34263100

C -2.99156200 -0.53549700 1.15401600

H -3.66539200 0.30776100 1.32775400

C -1.73784500 0.85213600 -1.09362500

H -0.98693000 1.61393800 -1.30975400

P -1.38186700 0.20922000 0.61536500

C -0.16033000 -1.14465500 0.51598500

C 0.63520100 -1.36324600 1.65663600

C -0.04266800 -1.95836500 -0.60873600

C 1.55196600 -2.41749900 1.64808900

H 0.54938600 -0.69748200 2.50504600

C 0.89635200 -3.00605600 -0.60682600

H -0.64785800 -1.82797700 -1.49757400

C 1.69000800 -3.23743700 0.51458900

H 2.42491100 -4.03202400 0.55135800

O 2.37036400 -2.73049700 2.68559700

O 0.94875200 -3.74054300 -1.75199300

C 1.89595400 -4.79585300 -1.83476100

H 1.70705900 -5.56639700 -1.07592600

H 1.77286000 -5.22923900 -2.82859400

H 2.92239900 -4.42351600 -1.72374900

C 2.29119100 -1.94536000 3.87127000

H 3.02207600 -2.37420100 4.55842700

H 2.54628000 -0.89695000 3.67499800

H 1.29119600 -1.99936200 4.31889200

H -2.81399900 -1.03123700 2.10864000

H -1.59158800 0.02301700 -1.79230100

C 2.81274800 1.72526000 0.82753200

H 3.08431800 2.57597100 1.46697500

H 2.89636800 0.82721300 1.45229000

C 3.80575500 1.62528700 -0.34955600

H 3.70414200 2.53268800 -0.96515900

H 3.53922700 0.77534200 -0.99135500

C 1.35326700 1.89019100 0.35910800

H 1.31491400 2.76015400 -0.32089000

H 1.09311200 1.02085200 -0.25987100

C -0.82283200 1.51283700 1.74697900

C -1.81122600 1.88709700 2.71438300

C 0.39721800 2.09845100 1.49956900

H 0.64658300 2.91255400 2.17503500

H -2.59969100 1.20236700 2.99470400

C -1.76647600 3.11737900 3.42353600

O -0.97130500 4.05891900 3.29135400

O -2.79924300 3.20248400 4.35070600

C -2.85085400 4.42665300 5.07073800

H -3.02897200 5.27944900 4.40523600

H -3.68224000 4.32502200 5.77361800

H -1.91907900 4.61302100 5.61595100

C 5.22807800 1.50195000 0.10132600

H 5.62906900 2.31285000 0.71007600

C 6.04147600 0.47122400 -0.16840900

H 5.71835200 -0.37568900 -0.76767200

C 7.43155300 0.44519900 0.33298500

O 7.95747700 1.30488900 1.01783500

O 8.06017000 -0.68840900 -0.07271300

C 9.44183600 -0.85204900 0.34072800

H 9.56030800 -1.93139600 0.46309400

H 9.57548700 -0.35594000 1.30419200

C 10.40642000 -0.31883100 -0.69139200

C 10.77046700 1.03547600 -0.69679000

C 10.94404400 -1.17054600 -1.66426400

C 11.65429600 1.52431200 -1.65916700

H 10.34749800 1.69896900 0.05143100

C 11.82921500 -0.68310200 -2.62712800

H 10.66877300 -2.22310400 -1.66563800

C 12.18565100 0.66680600 -2.62588000

H 11.93101800 2.57534400 -1.65290800

H 12.24135900 -1.35593100 -3.37466300

H 12.87650100 1.04857000 -3.37313400

**CP4-cis**

C -2.84814100 2.07953300 -0.51318400

C -4.08445500 1.46419100 -0.70417000

C -5.29788900 2.13792700 -0.34228000

C -5.21896700 3.47016300 0.17411000

C -3.95082200 4.08796600 0.28569600

C -2.77973700 3.43471100 -0.04605300

H -6.65670400 0.51183300 -0.80832700

C -6.58080900 1.52822300 -0.43873000

C -6.41135200 4.14745000 0.54774900

H -3.90259000 5.11835900 0.62851600

C -7.63624200 3.53169500 0.43409300

C -7.71760000 2.20695900 -0.05925000

H -6.33470500 5.16150400 0.93255500

H -8.54173400 4.05560700 0.72752800

H -8.68625300 1.72062900 -0.13553500

C -4.14573700 0.07565300 -1.27046800

C -4.64746000 -0.12594300 -2.59967100

C -3.68072900 -0.99797500 -0.51248700

C -5.06448400 0.94171300 -3.44474100

C -4.70253300 -1.45897100 -3.11784400

C -3.77227900 -2.33638100 -1.02669400

C -5.52354300 0.69847300 -4.72050500

H -5.01397400 1.95978500 -3.07517000

C -5.19020600 -1.67762700 -4.43508800

C -4.27416500 -2.53113200 -2.29946000

C -5.59354300 -0.62378000 -5.22212000

H -5.83394000 1.52892100 -5.34883200

H -5.23324100 -2.69726600 -4.81055600

H -4.35839500 -3.54418800 -2.68465600

H -5.96160700 -0.80136100 -6.22889200

C -3.37612200 -3.54194600 -0.23961500

C -3.98167400 -3.84262700 0.99132400

C -2.43246600 -4.44269100 -0.75847700

C -3.64804200 -5.00537200 1.68534900

H -4.72856800 -3.16840400 1.40114300

C -2.09841300 -5.60653700 -0.06518500

H -1.94584100 -4.21465200 -1.70263800

C -2.70564800 -5.89202600 1.15939000

H -4.12861400 -5.21946900 2.63593600

H -1.35852300 -6.28629500 -0.47919700

H -2.44782200 -6.79927000 1.69932900

C -1.50339300 4.20521500 0.04357300

C -0.73175100 4.45954100 -1.10284300

C -1.09547300 4.76820900 1.26283800

C 0.41600300 5.25052100 -1.03161900

H -1.04989400 4.05578100 -2.06099900

C 0.05171600 5.55997400 1.33468000

H -1.67691900 4.56882300 2.15825300

C 0.81149700 5.80419900 0.18832900

H 0.99394500 5.44333400 -1.93166900

H 0.35331600 5.98370800 2.28890500

H 1.70259300 6.42360300 0.24435600

C -3.02743400 -0.72069300 0.82161200

H -3.57643500 0.01337400 1.42124400

C -1.58872500 1.26609300 -0.74124800

H -0.70155500 1.89514000 -0.79319300

P -1.34432800 0.01621600 0.62573500

C -0.19239600 -1.26799900 0.03890800

C 0.12506100 -2.27526600 0.96812700

C 0.35996200 -1.25607600 -1.24150100

C 1.00528900 -3.28633400 0.57828200

H -0.33438000 -2.25143100 1.95202100

C 1.25570200 -2.27640300 -1.60879800

H 0.13650600 -0.48952900 -1.97397600

C 1.57728000 -3.28847000 -0.70717800

H 2.26091300 -4.09039200 -0.95685100

O 1.37608500 -4.31882100 1.37787100

O 1.75536800 -2.18324400 -2.87483100

C 2.66846000 -3.17791600 -3.31200800

H 2.20983000 -4.17553900 -3.30815200

H 2.93745800 -2.90787300 -4.33476100

H 3.57324800 -3.19567800 -2.69048300

C 0.81467300 -4.38045500 2.68917000

H 1.23821200 -5.27654100 3.14629500

H 1.08780200 -3.49951600 3.28150600

H -0.27728200 -4.46028100 2.65071700

H -2.89256900 -1.60001300 1.44873700

H -1.66176400 0.69877200 -1.67435400

C 2.80324700 1.44433900 1.46205000

H 3.06953600 2.02565300 2.35557400

H 2.85134100 0.38774400 1.75170500

C 3.82756200 1.72091900 0.34197800

H 3.75280800 2.78225800 0.05763900

H 3.56994100 1.13454200 -0.54948600

C 1.36228200 1.78969200 1.03429100

H 1.33487600 2.84548000 0.72134600

H 1.12723400 1.19710700 0.14322900

C -0.77266100 0.82728700 2.18622200

C -1.66852400 0.74213900 3.31836600

C 0.38320200 1.54476100 2.14857800

H 0.66367500 2.00113900 3.09967800

H -1.78975600 1.62139400 3.94230300

C -2.07495300 -0.50127300 3.82322200

O -1.84221700 -1.63729400 3.33775100

O -2.82126000 -0.40315300 4.98628600

C -3.21250900 -1.64070400 5.56102500

H -2.34715900 -2.27072700 5.79684300

H -3.74795300 -1.38644300 6.47982500

H -3.87177200 -2.20881000 4.89389000

C 5.23570100 1.42742600 0.75984400

H 5.63151100 1.98729100 1.60762700

C 6.04060600 0.52261700 0.18653200

H 5.72042600 -0.07797200 -0.66048200

C 7.41635600 0.30293900 0.68224600

O 7.93844700 0.88897900 1.61410000

O 8.03348600 -0.66086500 -0.04752800

C 9.39804600 -0.98829000 0.32516100

H 9.49084000 -2.05257700 0.09610000

H 9.50955700 -0.83240800 1.40004700

C 10.40578600 -0.17932200 -0.45564300

C 10.81137400 1.08485400 -0.00420300

C 10.94359800 -0.67945500 -1.64800900

C 11.73699700 1.83088400 -0.73433000

H 10.38741000 1.47834600 0.91472600

C 11.86985400 0.06563900 -2.37940900

H 10.63647700 -1.66060900 -2.00389800

C 12.26838200 1.32344400 -1.92271500

H 12.04609500 2.80856000 -0.37385800

H 12.28162200 -0.33618600 -3.30153800

H 12.99186200 1.90456900 -2.48861300

**TS21**

C 3.05011400 -1.10147400 -1.20832500

C 4.01682400 -0.10125500 -1.12076900

C 5.39925600 -0.44072700 -0.95281200

C 5.76711100 -1.82382800 -0.90769200

C 4.76183100 -2.80955300 -1.05197100

C 3.42916400 -2.48361400 -1.20460600

H 6.16066400 1.58618200 -0.80344900

C 6.42425600 0.53429100 -0.79059000

C 7.13203300 -2.17750100 -0.72548600

H 5.05208800 -3.85657900 -1.04206700

C 8.09888000 -1.20947300 -0.58048800

C 7.73704200 0.15928000 -0.60910900

H 7.39506000 -3.23213600 -0.69744800

H 9.13873300 -1.49082000 -0.43811900

H 8.50245900 0.92036000 -0.48318200

C 3.59765700 1.33948000 -1.17284100

C 3.90337800 2.12613400 -2.33311100

C 2.88271300 1.88930500 -0.10838700

C 4.56618800 1.59544200 -3.47634100

C 3.49982700 3.49895500 -2.36400400

C 2.52050000 3.27949200 -0.12978600

C 4.82322500 2.38356600 -4.57648300

H 4.86594600 0.55349900 -3.47649200

C 3.78776000 4.28841400 -3.51035500

C 2.82896600 4.03965900 -1.24138700

C 4.43605700 3.74546500 -4.59559900

H 5.32748000 1.95620900 -5.43900000

H 3.48017700 5.33151200 -3.51381100

H 2.56691700 5.09479700 -1.25066400

H 4.64889600 4.35609600 -5.46879600

C 1.85774200 3.96583200 1.01956800

C 2.47877900 4.03978000 2.27825000

C 0.62863500 4.62043600 0.84240300

C 1.88725500 4.74510400 3.32702300

H 3.44281600 3.56013300 2.42694700

C 0.03640000 5.32752900 1.88989200

H 0.13130000 4.55987700 -0.12146100

C 0.66403300 5.39343200 3.13587400

H 2.38939700 4.79940300 4.28938300

H -0.91777200 5.82330100 1.73294300

H 0.20744600 5.95037200 3.94983200

C 2.44111700 -3.59020700 -1.38966100

C 1.76046000 -3.74411700 -2.60945600

C 2.22899000 -4.52944300 -0.36743500

C 0.88667300 -4.81391900 -2.80802600

H 1.93541000 -3.03211100 -3.41299000

C 1.35592100 -5.60115900 -0.57371500

H 2.72566100 -4.39226100 0.59016400

C 0.68422800 -5.74831600 -1.78947700

H 0.37447900 -4.92346000 -3.76061400

H 1.20165300 -6.32401800 0.22369200

H 0.00892100 -6.58600000 -1.94437800

C 2.44513700 0.98793500 1.02261800

H 3.26137900 0.36097900 1.40312700

C 1.59113800 -0.69879900 -1.19505700

H 0.94236400 -1.51546100 -1.51506400

P 1.11700500 -0.22890100 0.55682100

C -0.44826600 0.71515300 0.44833600

C -0.91851500 1.32555700 1.63008000

C -1.22294700 0.73618600 -0.71124500

C -2.15358200 1.97478600 1.61952600

H -0.32352100 1.28944900 2.53434000

C -2.47369800 1.38312000 -0.70299300

H -0.89764300 0.27406800 -1.63626000

C -2.93761100 2.00633800 0.45228800

H -3.89823900 2.50334500 0.49824200

O -2.70142600 2.60697600 2.69266900

O -3.16172700 1.33776100 -1.87852900

C -4.39302800 2.04745600 -1.97072600

H -4.25199000 3.11462400 -1.75580900

H -4.72528000 1.92694000 -3.00340200

H -5.15102800 1.63225200 -1.29569400

C -2.00728900 2.55906300 3.93320100

H -2.64001900 3.09062600 4.64633800

H -1.86810700 1.52476900 4.27301700

H -1.03356600 3.05905000 3.86625900

H 2.06339700 1.55587900 1.87293600

H 1.40943700 0.16594600 -1.84224600

C -2.21134200 -2.48831000 1.60682900

H -2.45534100 -3.07969800 2.50000100

H -2.27905000 -1.43384500 1.89802800

C -3.23767700 -2.77398500 0.48845800

H -3.24236900 -3.85386000 0.27811900

H -2.91721600 -2.26957600 -0.43155600

C -0.77306400 -2.79125600 1.14682600

H -0.70000000 -3.84426900 0.83892300

H -0.56227600 -2.20995400 0.24278200

C 1.27644300 -1.66577200 2.23883800

C 2.41304300 -1.45565100 3.00382600

C 0.25790400 -2.53154600 2.20740000

H 0.17080400 -3.15586300 3.09886500

H 2.55615700 -0.53476200 3.56215000

C 3.50530700 -2.39352000 3.00799000

O 3.59462000 -3.47328200 2.42531300

O 4.53489300 -1.93534300 3.80423000

C 5.66849500 -2.79565900 3.87104800

H 6.12829500 -2.92172500 2.88479800

H 6.37078400 -2.30889200 4.55203500

H 5.39696400 -3.78513300 4.25331100

C -4.62048400 -2.32531300 0.84902200

H -5.12028800 -2.83112600 1.67532100

C -5.26717800 -1.31722300 0.24759100

H -4.81880800 -0.76773600 -0.57516600

C -6.61303600 -0.89880600 0.68832300

O -7.27751700 -1.43224700 1.55804700

O -7.02476500 0.19476700 -0.01116900

C -8.33982600 0.72104200 0.31568200

H -8.24315800 1.79956400 0.17006800

H -8.54184500 0.50853400 1.36720400

C -9.41290600 0.15020800 -0.57933600

C -10.02066800 -1.07616600 -0.27317800

C -9.81003300 0.83591400 -1.73380800

C -11.00461100 -1.60323200 -1.10993300

H -9.70899000 -1.61255300 0.61801300

C -10.79540800 0.31043500 -2.57113800

H -9.34659100 1.78990800 -1.97642600

C -11.39419600 -0.91169100 -2.25990200

H -11.47051200 -2.55343400 -0.86249100

H -11.09649800 0.85478300 -3.46233900

H -12.16335900 -1.32239000 -2.90871500

**TS22**

C 3.01888900 1.87170400 0.70937200

C 4.05920600 0.98914600 0.99459400

C 5.42139200 1.36060300 0.74445600

C 5.69691900 2.66782700 0.23046900

C 4.61845800 3.55460100 -0.00138500

C 3.30435500 3.19222800 0.22454300

H 6.32350300 -0.52482100 1.32529700

C 6.51659300 0.47527700 0.95366200

C 7.04171300 3.04800400 -0.02940800

H 4.83757200 4.55899600 -0.35517200

C 8.07769800 2.17030300 0.19216500

C 7.80826300 0.86976600 0.68322900

H 7.23490300 4.04696600 -0.41321100

H 9.10194400 2.46896000 -0.01359100

H 8.62882800 0.17629800 0.84607600

C 3.74263500 -0.37030400 1.54670000

C 4.05122100 -0.66473700 2.91695200

C 3.11628900 -1.31673400 0.73503200

C 4.62198900 0.28916800 3.80718900

C 3.74196700 -1.96432500 3.42983800

C 2.85553200 -2.63409900 1.24677000

C 4.88262300 -0.03301600 5.12101500

H 4.84735000 1.28481500 3.44174300

C 4.03084400 -2.26878900 4.78780100

C 3.16386600 -2.92130100 2.56226300

C 4.59010000 -1.32572700 5.61895500

H 5.31574200 0.71244700 5.78257500

H 3.79587400 -3.26382300 5.15844300

H 2.97567300 -3.92216400 2.94296900

H 4.80434200 -1.56719400 6.65649000

C 2.30618900 -3.73892200 0.40448900

C 3.01626700 -4.21713500 -0.70921400

C 1.10651900 -4.37546900 0.75714000

C 2.53919400 -5.29876000 -1.44932100

H 3.95662800 -3.74685200 -0.98459800

C 0.62775900 -5.45760800 0.01674700

H 0.54063700 -4.00593600 1.60762300

C 1.34299400 -5.92358300 -1.08828800

H 3.10654800 -5.65868400 -2.30352200

H -0.30803500 -5.93160100 0.30044400

H 0.97240500 -6.76823300 -1.66300500

C 2.24217700 4.21280400 -0.02152500

C 1.40563900 4.65928200 1.01605800

C 2.10680600 4.80311800 -1.28802000

C 0.45887900 5.65915900 0.79185600

H 1.51682500 4.23515800 2.01086700

C 1.16167900 5.80608000 -1.51234500

H 2.74075600 4.46194900 -2.10146300

C 0.33338900 6.23663600 -0.47391900

H -0.17269900 5.99561100 1.60985600

H 1.07317700 6.25000700 -2.50052800

H -0.40105900 7.01842100 -0.64775500

C 2.65811300 -0.92156000 -0.64980200

H 3.44474400 -0.41767800 -1.22524700

C 1.59873600 1.36262900 0.82299900

H 0.87085400 2.17412700 0.83712000

P 1.22366300 0.26972200 -0.65991700

C -0.27053200 -0.69160800 -0.22291000

C -0.66016900 -1.68750200 -1.14010500

C -1.05953400 -0.39361900 0.88906000

C -1.83586800 -2.40108600 -0.90142100

H -0.05131300 -1.87548100 -2.01852800

C -2.25340500 -1.10803700 1.09977600

H -0.78968600 0.36776300 1.61206800

C -2.64026200 -2.11316100 0.21685900

H -3.55524800 -2.67788200 0.34528400

O -2.29981900 -3.39265800 -1.70725700

O -2.97036600 -0.73410100 2.20114600

C -4.14195800 -1.47275900 2.52626200

H -3.91509600 -2.53793400 2.66493400

H -4.50269100 -1.05455500 3.46807400

H -4.91837100 -1.35762000 1.76021500

C -1.54859300 -3.70409300 -2.87993300

H -2.10511900 -4.49900800 -3.38027400

H -1.46358800 -2.83650000 -3.54414100

H -0.54229500 -4.05552800 -2.62754400

H 2.33738800 -1.77623700 -1.24631700

H 1.46782700 0.76626900 1.73219500

C -2.23098900 1.87305800 -2.28870100

H -2.56103300 2.11240000 -3.30896700

H -2.23870600 0.78090000 -2.20221100

C -3.21820500 2.47082700 -1.26291400

H -3.25349300 3.56263800 -1.39625800

H -2.84289300 2.28170500 -0.24971100

C -0.79531300 2.38560400 -2.06496600

H -0.77734000 3.48045700 -2.16521200

H -0.50254900 2.17663800 -1.03182300

C 1.22410300 0.97326500 -2.84671500

C 2.26083600 0.42141700 -3.57780700

C 0.18810600 1.79072100 -3.03239700

H 0.00397900 2.04338100 -4.07964300

H 3.12104800 1.02674300 -3.85030700

C 2.27069200 -0.96520200 -3.93309100

O 1.44774400 -1.83034400 -3.60612800

O 3.35105500 -1.28689700 -4.71863300

C 3.41148000 -2.64714500 -5.13906400

H 2.54662400 -2.91104100 -5.75711000

H 4.33011800 -2.73626300 -5.72315400

H 3.43996800 -3.32804100 -4.28184200

C -4.59792400 1.90405600 -1.40266900

H -5.14603200 2.12501100 -2.31874200

C -5.18801500 1.11171800 -0.49715800

H -4.69290400 0.84092500 0.43107900

C -6.53422700 0.55258000 -0.73731300

O -7.24514000 0.79068100 -1.69687500

O -6.88523400 -0.29432100 0.26888000

C -8.18433900 -0.93617300 0.15806500

H -8.04000700 -1.91748300 0.61623700

H -8.41818700 -1.05602700 -0.90143700

C -9.26132500 -0.15962900 0.87605500

C -9.93771600 0.88652500 0.23171400

C -9.59548600 -0.47096200 2.19978700

C -10.92763400 1.60464200 0.90292900

H -9.67444200 1.13511200 -0.79202000

C -10.58625200 0.24615700 2.87233500

H -9.07831800 -1.28351500 2.70575200

C -11.25431800 1.28635400 2.22359400

H -11.44733200 2.41192900 0.39353100

H -10.83777200 -0.00850100 3.89854900

H -12.02820400 1.84495100 2.74358900

**TS23**

C -3.12515500 -1.38341200 0.14882300

C -3.70764000 -0.61150400 -0.85427200

C -4.35793300 -1.23417000 -1.97101000

C -4.42137700 -2.66317900 -2.01918500

C -3.85824200 -3.41157400 -0.95828100

C -3.22357000 -2.81360000 0.11288000

H -4.85864900 0.58431500 -3.04619700

C -4.91363900 -0.49844200 -3.05621700

C -5.05121800 -3.29845400 -3.12385800

H -3.93885000 -4.49511800 -0.98469700

C -5.58650800 -2.55713000 -4.15146300

C -5.51064400 -1.14358000 -4.11650000

H -5.09541600 -4.38456900 -3.14265900

H -6.06226000 -3.05195500 -4.99354900

H -5.92546500 -0.56292500 -4.93585000

C -3.61079100 0.88481700 -0.79857400

C -4.77969000 1.66627500 -0.51826600

C -2.37776800 1.50046400 -1.01027400

C -6.04423000 1.08633700 -0.21571700

C -4.66727100 3.09295600 -0.50711900

C -2.28453000 2.93245600 -1.06092900

C -7.13447600 1.87760500 0.07130700

H -6.14198700 0.00638200 -0.20623800

C -5.81370800 3.88172700 -0.21674500

C -3.41679300 3.68365500 -0.80603100

C -7.02243800 3.28935200 0.06714400

H -8.08970100 1.41523500 0.30419400

H -5.71472800 4.96449500 -0.21940700

H -3.35366700 4.76747900 -0.85855100

H -7.89209100 3.90060300 0.29173500

C -1.03661400 3.65924600 -1.43849800

C -0.40507400 3.42309700 -2.67174700

C -0.51785300 4.66144200 -0.60333000

C 0.71427900 4.16308700 -3.05458700

H -0.81102000 2.67423100 -3.34718500

C 0.59931500 5.40427000 -0.98677600

H -0.98658700 4.84443700 0.35936300

C 1.21945700 5.15770800 -2.21374100

H 1.18337200 3.97039900 -4.01557700

H 0.98965600 6.17070300 -0.32292600

H 2.08777800 5.73770200 -2.51407800

C -2.71315300 -3.69755800 1.20512300

C -3.32898000 -3.69676800 2.46781100

C -1.66796600 -4.60222300 0.96310000

C -2.91328100 -4.58230400 3.46344100

H -4.15391000 -3.01459200 2.66014900

C -1.25271400 -5.48667200 1.96056200

H -1.15676700 -4.59279200 0.00500800

C -1.87476700 -5.48160200 3.21084500

H -3.40607600 -4.57464500 4.43214700

H -0.43514400 -6.17293900 1.75855000

H -1.55061200 -6.17192600 3.98489900

C -1.14474900 0.62375700 -1.11861400

H -1.30705900 -0.21796200 -1.79690200

C -2.31990200 -0.68235100 1.22775000

H -2.10675200 -1.34138800 2.06906200

P -0.69605100 -0.10603800 0.53165300

C -0.03778500 1.27681400 1.52131600

C 1.17071900 1.83874600 1.06787600

C -0.67220300 1.74757400 2.66785400

C 1.74002400 2.88929100 1.79298000

H 1.66857800 1.43874500 0.19338300

C -0.08412600 2.80217100 3.39108700

H -1.59745100 1.32605500 3.04211600

C 1.11111400 3.37045900 2.95727800

H 1.59285900 4.18073200 3.49012300

O 2.89450000 3.51468400 1.46626000

O -0.76654600 3.19526600 4.50122100

C -0.21474100 4.23151800 5.30113500

H -0.12924600 5.17089300 4.73941800

H -0.90933700 4.36860300 6.13150900

H 0.77190300 3.95322800 5.69280000

C 3.57524800 3.14011200 0.26322900

H 4.44561500 3.79626400 0.21216200

H 3.91020100 2.09821600 0.28010100

H 2.93752700 3.31125000 -0.61183500

H -0.27520300 1.17989000 -1.47048600

H -2.84899500 0.19961200 1.60096200

C 1.54250800 -2.30715800 3.83980900

H 1.31082300 -1.99710300 4.86623800

H 1.78262500 -3.37912600 3.87110300

C 2.71641700 -1.53183800 3.23779800

H 3.65681900 -1.75489700 3.75990800

H 2.54352800 -0.45141300 3.34493500

C 0.35813800 -2.07571600 2.88448000

H -0.46386600 -2.76955500 3.10273700

H -0.01566700 -1.05783300 3.05998400

C 0.44587700 -1.48073500 0.33787300

C 1.00705000 -1.59833400 -0.93221100

C 0.90048600 -2.23684800 1.47377300

H 1.08335200 -3.27083800 1.19655300

H 0.80757300 -0.87499600 -1.71260800

C 1.72662500 -2.78396100 -1.38825000

O 1.80709700 -3.86379200 -0.81610300

O 2.27621500 -2.56916200 -2.61663800

C 3.04695900 -3.66516700 -3.12684300

H 2.41619700 -4.54735300 -3.27780400

H 3.44546900 -3.31979100 -4.08313600

H 3.85491500 -3.90465900 -2.43371300

C 2.85055500 -1.88413100 1.75920800

H 3.19825200 -2.90305200 1.58740600

C 3.42127900 -0.95484800 0.86573000

H 3.26445600 0.10266300 1.04643500

C 4.28468200 -1.29041900 -0.21711000

O 4.69684900 -2.39893800 -0.56567700

O 4.69438600 -0.12687000 -0.88552300

C 5.56784300 -0.30884100 -2.00653100

H 5.24816100 0.42170000 -2.75787400

H 5.42176600 -1.31777700 -2.40030800

C 7.02143200 -0.08459300 -1.64800700

C 7.70712800 1.04701400 -2.10287700

C 7.70655100 -1.01596500 -0.85211700

C 9.05041100 1.25135500 -1.77656100

H 7.18499300 1.77470100 -2.72159000

C 9.04573200 -0.81210200 -0.52161700

H 7.17295000 -1.89464000 -0.50108200

C 9.72258100 0.32134100 -0.98290300

H 9.56897000 2.13507900 -2.14074300

H 9.56600300 -1.54167400 0.09428200

H 10.76765000 0.47619700 -0.72609000

**TS24**

C -2.74811600 -1.72465400 -0.08416700

C -3.21802700 -1.12484600 -1.25149200

C -3.53510200 -1.91605000 -2.40552700

C -3.40761700 -3.33850900 -2.32066200

C -3.00824500 -3.91725500 -1.09304500

C -2.68836100 -3.15569500 0.01468600

H -4.02092600 -0.27185800 -3.73627600

C -3.93674200 -1.34919800 -3.64835900

C -3.70330600 -4.13713500 -3.45870400

H -2.97442700 -5.00094400 -1.01577000

C -4.09522600 -3.55741000 -4.64304400

C -4.20717200 -2.14894900 -4.73670000

H -3.60750000 -5.21697900 -3.37542900

H -4.31424000 -4.17568000 -5.50919400

H -4.50659200 -1.69520300 -5.67747700

C -3.36941900 0.36573600 -1.31319200

C -4.68177300 0.94441600 -1.34852900

C -2.23350800 1.17244200 -1.30773900

C -5.87512200 0.17106400 -1.27603300

C -4.80165900 2.36830500 -1.42691000

C -2.36094300 2.59810400 -1.43093300

C -7.11293800 0.77508600 -1.29617300

H -5.80263900 -0.90802600 -1.19868300

C -6.09373300 2.96056900 -1.45559600

C -3.62559800 3.15320700 -1.48483400

C -7.22703000 2.18320300 -1.39265000

H -8.01079700 0.16602800 -1.23638400

H -6.16795000 4.04329500 -1.52339500

H -3.72487300 4.23031700 -1.59269600

H -8.21006400 2.64561600 -1.41187000

C -1.19098400 3.51877300 -1.54652500

C -0.26071200 3.38948300 -2.59081200

C -1.04392600 4.58798100 -0.64869400

C 0.78742100 4.29909000 -2.72954400

H -0.36893900 2.58119800 -3.30889400

C 0.00383800 5.49895500 -0.78684200

H -1.74674200 4.69054200 0.17329700

C 0.92301400 5.35785000 -1.82863700

H 1.49503700 4.18248900 -3.54568300

H 0.10576800 6.31410600 -0.07557400

H 1.73805100 6.06814300 -1.93914900

C -2.37068600 -3.87940700 1.28145600

C -3.18207000 -3.72766500 2.41864600

C -1.31513000 -4.80322600 1.33063500

C -2.93987600 -4.47297800 3.57354600

H -4.02360900 -3.03992200 2.38657000

C -1.07071000 -5.54675500 2.48627200

H -0.67753400 -4.92636500 0.45978300

C -1.88164500 -5.38377800 3.61156600

H -3.58379400 -4.34801500 4.44013200

H -0.24358700 -6.25112700 2.50718300

H -1.69187600 -5.96373900 4.51056400

C -0.87949900 0.52276000 -1.12288200

H -0.77251400 -0.37665900 -1.73701400

C -2.25754100 -0.83593100 1.04350100

H -2.14104700 -1.38842800 1.97328100

P -0.61763000 -0.03711200 0.62870100

C -0.43653500 1.46099100 1.66445900

C 0.75632000 2.18740200 1.49082800

C -1.39861800 1.85722000 2.59118500

C 0.95917600 3.33368200 2.26206100

H 1.47579900 1.86068600 0.74828300

C -1.16971100 3.00705300 3.36923400

H -2.31951100 1.31132700 2.75671800

C 0.00082400 3.74381900 3.20730100

H 0.21087400 4.63320000 3.78831300

O 2.05564900 4.12860100 2.17265300

O -2.15869200 3.31441400 4.25522600

C -1.98562300 4.44923600 5.09043300

H -1.90084800 5.37289100 4.50292200

H -2.87910100 4.49921300 5.71517900

H -1.09928400 4.34623400 5.72977200

C 3.07620200 3.76235200 1.24478700

H 3.84642600 4.52975200 1.33924500

H 3.50277300 2.78262000 1.49065100

H 2.69378800 3.74365600 0.21820400

H -0.04608400 1.17846700 -1.35891900

H -2.96268800 -0.01775500 1.21726300

C 1.05665500 -1.60581000 4.47105300

H 0.59738500 -1.10121000 5.32990300

H 1.36394600 -2.60429500 4.81154900

C 2.28306600 -0.86381200 3.92879100

H 3.08493000 -0.83044200 4.67993000

H 2.02689700 0.17605800 3.68359300

C 0.05847000 -1.73987700 3.29715000

H -0.67651000 -2.52750300 3.50261900

H -0.48766000 -0.79221700 3.23722400

C 0.70181900 -1.24781000 0.80075200

C 1.65970300 -1.44852500 -0.20905400

C 0.85061100 -1.99167200 2.02222400

H 1.11721000 -3.03489700 1.85661100

H 2.14187500 -2.42021900 -0.24818100

C 2.17616800 -0.47069800 -1.13192400

O 1.99169500 0.75507500 -1.11445600

O 2.99437400 -1.03770200 -2.06359900

C 3.71587100 -0.11609700 -2.88506200

H 4.36630000 0.51649100 -2.27528200

H 4.31781900 -0.73020400 -3.55673400

H 3.03227000 0.51733800 -3.45862400

C 2.73892200 -1.60539800 2.68698600

H 2.98884900 -2.64799400 2.89025300

C 3.55345500 -1.05082000 1.68798700

H 3.61268100 0.02078000 1.52569200

C 4.43191900 -1.89423000 0.91909300

O 4.46637100 -3.12268800 0.92180000

O 5.28368400 -1.15876700 0.11572500

C 6.15259200 -1.93882400 -0.70565800

H 5.56072400 -2.52771400 -1.41589900

H 6.69416200 -2.65806300 -0.07756800

C 7.11412300 -1.02663500 -1.43141800

C 7.50180100 0.20895200 -0.89809400

C 7.67296400 -1.44025200 -2.64787900

C 8.42891400 1.01038800 -1.56709200

H 7.06219100 0.53776700 0.03773600

C 8.60542000 -0.64378800 -3.31421900

H 7.37439000 -2.39499400 -3.07647400

C 8.98641800 0.58695800 -2.77548100

H 8.71737800 1.96869300 -1.14186000

H 9.02832300 -0.98130600 -4.25720600

H 9.70861100 1.21175300 -3.29471400

**TS25**

C -3.12586100 -1.35836000 0.22548900

C -3.73783900 -0.52261100 -0.70893800

C -4.55752800 -1.07774000 -1.74720500

C -4.80607400 -2.48752900 -1.74744200

C -4.24868000 -3.28511300 -0.71914800

C -3.40979800 -2.76561700 0.24863400

H -4.93145700 0.77402000 -2.81571300

C -5.12202500 -0.29337500 -2.79261700

C -5.61341700 -3.05389700 -2.77156700

H -4.47289000 -4.34855600 -0.70592600

C -6.14673600 -2.26642100 -3.76542900

C -5.89268300 -0.87331300 -3.77583100

H -5.79551200 -4.12566400 -2.75647400

H -6.75855300 -2.70974100 -4.54614000

H -6.30777000 -0.25743700 -4.56891000

C -3.59135800 0.97123100 -0.60478200

C -4.72852400 1.74908500 -0.19555000

C -2.37299800 1.59593300 -0.87142700

C -5.97256700 1.16733000 0.18117500

C -4.60257700 3.17348700 -0.12460000

C -2.26787800 3.02806200 -0.84744700

C -7.02946600 1.95233200 0.58622500

H -6.08360800 0.08958200 0.15511800

C -5.71560900 3.95664900 0.28576900

C -3.37090900 3.77293400 -0.47875200

C -6.90591400 3.36178000 0.63392900

H -7.96730000 1.48511900 0.87375100

H -5.60426900 5.03754900 0.32423200

H -3.30005200 4.85761700 -0.47569900

H -7.74984600 3.96837700 0.95044100

C -1.03043600 3.75773400 -1.25460300

C -0.48510000 3.60024000 -2.54052000

C -0.42514000 4.67316600 -0.37956500

C 0.63731800 4.32912800 -2.93490900

H -0.95802400 2.91829700 -3.24279100

C 0.69657000 5.40407500 -0.77362700

H -0.82732100 4.79419800 0.62217400

C 1.23249100 5.23357600 -2.05194100

H 1.04066100 4.19708700 -3.93518900

H 1.15676400 6.10029000 -0.07796300

H 2.10496600 5.80323400 -2.35991200

C -2.91414100 -3.68505200 1.32158900

C -3.84594000 -4.30140900 2.17456700

C -1.55463100 -3.99684500 1.47999700

C -3.42774900 -5.19527100 3.16133000

H -4.90220100 -4.06886000 2.06546100

C -1.13617400 -4.89180100 2.46601000

H -0.81371800 -3.55585500 0.82341300

C -2.07101400 -5.49227400 3.31105500

H -4.16340700 -5.65797300 3.81425500

H -0.07621900 -5.11370700 2.55388400

H -1.74664300 -6.18913600 4.07937500

C -1.15157900 0.73627200 -1.12201800

H -1.34051400 -0.01235500 -1.89074300

C -2.18094500 -0.73547000 1.22829300

H -1.89705400 -1.43315700 2.01877100

P -0.61020100 -0.14744300 0.42464800

C 0.09842700 1.15249900 1.49412800

C 1.29467600 1.75919400 1.06414300

C -0.51683200 1.54109300 2.68105200

C 1.86609200 2.76297800 1.84944700

H 1.79252000 1.41236900 0.16863600

C 0.06619200 2.55977600 3.45885300

H -1.42477600 1.08472200 3.05366000

C 1.24643200 3.17029500 3.04632000

H 1.72812900 3.94987000 3.62327300

O 3.01688600 3.40878400 1.55079100

O -0.60599800 2.86953700 4.60099800

C -0.06112500 3.86492200 5.45533300

H -0.00035900 4.83798700 4.95059500

H -0.74602400 3.93859600 6.30161300

H 0.93577800 3.58158200 5.81630000

C 3.73493300 3.02075700 0.37443700

H 4.63183500 3.64226600 0.36646500

H 4.02076100 1.96342700 0.39933100

H 3.14445200 3.22691800 -0.52660500

H -0.29107300 1.32561700 -1.44312700

H -2.64454800 0.14206300 1.68797200

C 0.26391500 -2.41136700 -3.40559300

H 0.69235400 -3.37684600 -3.70583800

H -0.44696100 -2.12348800 -4.18982600

C 1.40475000 -1.40146400 -3.23680700

H 1.00831100 -0.38310500 -3.10651900

H 2.04187500 -1.37551600 -4.13161300

C -0.42707800 -2.56186700 -2.02668300

H -1.25162200 -1.85014100 -1.94450100

H -0.89686100 -3.54987900 -1.94210600

C 0.56756900 -1.49260300 0.18921300

C 1.56776100 -1.50150300 1.16455000

C 0.62958900 -2.37268300 -0.94563700

H 1.09962000 -3.30582200 -0.65093000

H 1.67712300 -0.69012000 1.86987700

C 2.41138300 -2.65858400 1.45879400

O 2.27456100 -3.80694500 1.05355800

O 3.39611700 -2.31911900 2.33721800

C 4.31297600 -3.37648500 2.64485400

H 4.78381000 -3.74067700 1.72935000

H 5.05457000 -2.93355300 3.31272500

H 3.80121000 -4.20484300 3.14534900

C 2.22069000 -1.80353500 -2.01616500

H 2.66355200 -2.79598700 -2.11468600

C 3.07619700 -0.90010200 -1.35583500

H 2.85472200 0.16443700 -1.36001100

C 4.30305700 -1.29731100 -0.74043100

O 4.77685900 -2.43141300 -0.65131100

O 4.98690400 -0.18935700 -0.23200400

C 6.24989200 -0.45207300 0.39842500

H 6.24552600 -1.47835800 0.77281700

H 6.30964500 0.24164400 1.24327700

C 7.41545500 -0.23286500 -0.54221000

C 7.67053900 -1.14367600 -1.57929900

C 8.25701300 0.87552100 -0.39596200

C 8.74216100 -0.94196400 -2.44831500

H 7.01676700 -2.00384200 -1.69091500

C 9.33407700 1.07726900 -1.26294900

H 8.06924400 1.58642400 0.40670900

C 9.57787600 0.16827000 -2.29310100

H 8.92997800 -1.65537100 -3.24707600

H 9.97976000 1.94255600 -1.13359400

H 10.41480700 0.32141300 -2.96996100

**TS26**

C 2.28513000 -2.05788300 -0.49798800

C 3.44988700 -1.68451200 0.16917000

C 4.15145200 -2.62271100 0.99836300

C 3.65663300 -3.96277400 1.08940800

C 2.51079200 -4.32400900 0.34092200

C 1.82499900 -3.41728300 -0.44377300

H 5.67680400 -1.26055600 1.72692100

C 5.29852100 -2.27584200 1.76736600

C 4.33144900 -4.90290900 1.91488000

H 2.17138600 -5.35616200 0.37454500

C 5.44260500 -4.53640000 2.63854800

C 5.92492100 -3.20726000 2.56589500

H 3.94714900 -5.91872800 1.96667000

H 5.94887500 -5.26150300 3.26972800

H 6.79648500 -2.91922800 3.14736900

C 3.96196900 -0.27918400 0.05131200

C 5.17419700 -0.02373700 -0.67233300

C 3.25236600 0.76748900 0.63708300

C 5.88983200 -1.03927300 -1.36759900

C 5.67289100 1.31657300 -0.73023500

C 3.79400200 2.09879300 0.63474600

C 7.03808700 -0.74308800 -2.06821800

H 5.51625400 -2.05689100 -1.34834000

C 6.86638100 1.58795100 -1.45332100

C 4.97308300 2.33844900 -0.04513700

C 7.53689900 0.58154600 -2.10914700

H 7.56570600 -1.53247500 -2.59646400

H 7.23609000 2.61008300 -1.48180400

H 5.39177500 3.34147900 -0.03926400

H 8.44587000 0.79964000 -2.66293300

C 3.17569600 3.23261600 1.38530000

C 2.99003700 3.16106600 2.77680400

C 2.84173900 4.42646400 0.72638700

C 2.47556400 4.24656600 3.48628300

H 3.27428900 2.25636300 3.30859000

C 2.32750800 5.51194000 1.43704900

H 2.96996200 4.49273800 -0.35004900

C 2.14054000 5.42588500 2.81776100

H 2.34581100 4.17335400 4.56277500

H 2.06623600 6.42492800 0.90859100

H 1.73820900 6.27119500 3.36927500

C 0.66284500 -3.91314300 -1.23985000

C 0.67821000 -3.86857800 -2.64350300

C -0.43874500 -4.50182700 -0.59993300

C -0.38150900 -4.39252800 -3.38477500

H 1.53426100 -3.43765800 -3.15659900

C -1.50138400 -5.02081400 -1.34032200

H -0.46677400 -4.53366900 0.48558300

C -1.47580600 -4.96784200 -2.73501700

H -0.34743200 -4.35701400 -4.47055700

H -2.35356400 -5.45618400 -0.82628000

H -2.30357400 -5.37170900 -3.31140100

C 1.89788700 0.47922700 1.25232400

H 1.95060000 -0.38793000 1.91374000

C 1.48101400 -0.99759900 -1.21556000

H 0.72697500 -1.39936600 -1.88648500

P 0.57267900 0.09612400 -0.01037000

C 0.15534900 1.68926900 -0.80359400

C -1.11872800 2.24306600 -0.58790400

C 1.10409400 2.33727600 -1.59053300

C -1.43021100 3.46153700 -1.19908500

H -1.85565800 1.69856100 -0.01126800

C 0.78046200 3.57195900 -2.17668700

H 2.09200100 1.93321400 -1.77394000

C -0.47860400 4.13198100 -1.98837300

H -0.76838800 5.07541400 -2.43412000

O -2.62876800 4.09164000 -1.10589700

O 1.78139000 4.15080100 -2.90439300

C 1.49915800 5.36453300 -3.58527200

H 1.24209200 6.17001700 -2.88481000

H 2.41422400 5.62787800 -4.11879100

H 0.68041000 5.23903600 -4.30523500

C -3.65524600 3.50575200 -0.30746500

H -4.51602800 4.16950700 -0.40404800

H -3.92830600 2.50237300 -0.65107200

H -3.35311700 3.45770800 0.74605600

H 1.54472700 1.32893700 1.83356200

H 2.12261800 -0.33268900 -1.80149800

C -1.17530900 -0.32173700 4.50255500

H -1.46168900 -1.30287600 4.90692000

H -0.71203300 0.24287600 5.32081400

C -2.40536500 0.37515300 3.91870000

H -2.15223300 1.40756600 3.63670600

H -3.22207100 0.43663800 4.65018600

C -0.21228900 -0.50031900 3.31667700

H 0.18674900 0.49049900 3.08384700

H 0.63721100 -1.14230700 3.59340300

C -0.82678600 -0.79572500 0.73784100

C -1.85433300 -1.22766900 -0.10097700

C -1.02869800 -1.04486100 2.15536600

H -1.30692000 -2.08692900 2.31779600

H -2.62743100 -1.84851700 0.33448500

C -1.96933600 -1.02892000 -1.52875500

O -1.17526500 -0.42437000 -2.25480000

O -3.10414900 -1.58363300 -2.02544300

C -3.30731700 -1.40859600 -3.42934000

H -2.50760600 -1.89088000 -3.99979300

H -4.26676700 -1.88055100 -3.64667300

H -3.33604100 -0.34678700 -3.69154000

C -2.85673100 -0.40587900 2.69288000

H -3.25119800 -1.39015800 2.94992000

C -3.61192700 0.20596800 1.66989000

H -3.50017200 1.26538400 1.46418200

C -4.62586800 -0.50448000 0.95571300

O -4.91831600 -1.69892800 1.06933800

O -5.30187100 0.32495100 0.06841600

C -6.30564100 -0.30157500 -0.74810000

H -6.04825000 -1.35736000 -0.86211000

H -6.24243900 0.19634600 -1.72107100

C -7.69707400 -0.14460300 -0.17394500

C -8.08861400 -0.88000600 0.95564600

C -8.61722900 0.73184000 -0.76007400

C -9.37150300 -0.73453100 1.48256300

H -7.37233600 -1.55842900 1.40986500

C -9.90443700 0.87593800 -0.23631600

H -8.32384700 1.30635800 -1.63675000

C -10.28392000 0.14274000 0.88851900

H -9.66314900 -1.31029000 2.35766300

H -10.60730300 1.56020900 -0.70537200

H -11.28433600 0.25211900 1.29993800
